# Cryo-EM Structures of MDA5-dsRNA Filaments at Different Stages of ATP Hydrolysis

**DOI:** 10.1016/j.molcel.2018.10.012

**Published:** 2018-12-20

**Authors:** Qin Yu, Kun Qu, Yorgo Modis

**Affiliations:** 1 Department of Medicine, University of Cambridge, MRC Laboratory of Molecular Biology, Cambridge Biomedical Campus, Cambridge CB2 0QH, UK; 2 MRC Laboratory of Molecular Biology, Cambridge Biomedical Campus, Cambridge CB2 0QH, UK

**Keywords:** RIG-I-like receptor, RLR, nucleic acid sensing, innate immune pattern recognition, DExD/H-box RNA helicase, superfamily 2 helicases, SF2 helicases, cryoelectron microscopy, cryo-EM, helical reconstruction, ATPase, Aicardi-Goutières syndrome, AGS, Singleton-Merten syndrome, SMS

## Abstract

Double-stranded RNA (dsRNA) is a potent proinflammatory signature of viral infection. Long cytosolic dsRNA is recognized by MDA5. The cooperative assembly of MDA5 into helical filaments on dsRNA nucleates the assembly of a multiprotein type I interferon signaling platform. Here, we determined cryoelectron microscopy (cryo-EM) structures of MDA5-dsRNA filaments with different helical twists and bound nucleotide analogs at resolutions sufficient to build and refine atomic models. The structures identify the filament-forming interfaces, which encode the dsRNA binding cooperativity and length specificity of MDA5. The predominantly hydrophobic interface contacts confer flexibility, reflected in the variable helical twist within filaments. Mutation of filament-forming residues can result in loss or gain of signaling activity. Each MDA5 molecule spans 14 or 15 RNA base pairs, depending on the twist. Variations in twist also correlate with variations in the occupancy and type of nucleotide in the active site, providing insights on how ATP hydrolysis contributes to MDA5-dsRNA recognition.

## Highlights

• Cryo-EM structures of MDA5-dsRNA filaments determined for three catalytic states
• Filament forming interfaces are flexible and predominantly hydrophobic
• Mutation of filament-forming residues can cause loss or gain of IFN-β signaling
• ATPase cycle is coupled to changes in filament twist and size of the RNA footprint

## Graphical Abstract

**Figure:**
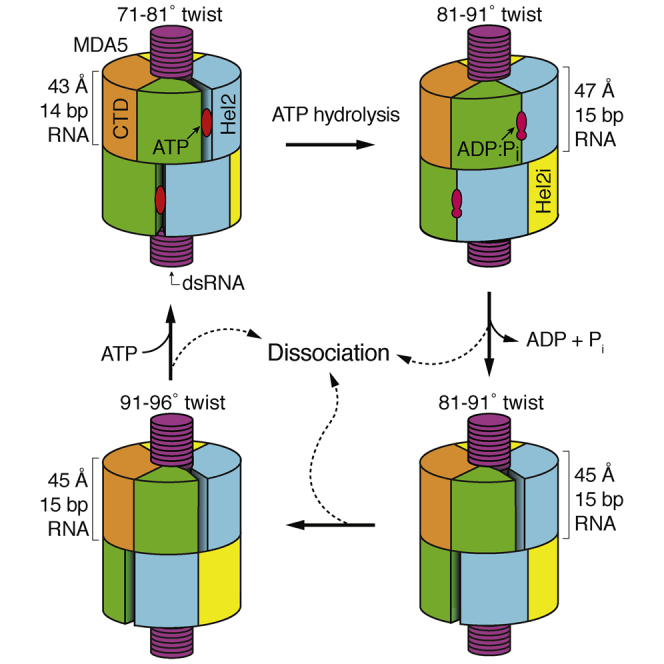

## Teaser

Cryo-EM structures determined by Yu et al. show how the innate immune sensor MDA5 forms helical filaments to recognize cytosolic double-stranded RNA. Mutations at the hydrophobic filament interface can cause loss or gain of signaling activity. ATP binding and hydrolysis cause structural changes, which are proposed to confer proofreading activity.

## Introduction

Recognition of viral nucleic acids by innate immune receptors is one of the most conserved and important mechanisms for sensing viral infection. Many viruses deliver or generate double-stranded RNA (dsRNA) in the cytosol of the host cell. RNA duplexes have the A-form double-helix structure distinct from the B-form structure of DNA (Pabit et al., 2016), and cytosolic double-stranded RNA (dsRNA) is a potent proinflammatory signal in vertebrates. Uninterrupted RNA duplexes longer than a few hundred base pairs are recognized in the cytosol by the innate immune receptor MDA5 (melanoma differentiation-associated gene-5; Kato et al., 2006, Kato et al., 2008). The cooperative assembly of MDA5 into ATP-sensitive filaments on dsRNA induces oligomerization of its tandem N-terminal caspase recruitment domains (CARDs) (Berke and Modis, 2012, Peisley et al., 2011). The MDA5 CARD oligomers nucleate the growth of microfibrils of the CARD from MAVS (mitochondrial antiviral signaling protein; Hou et al., 2011, Wu et al., 2014). The amyloid-like properties of MAVS CARD microfibrils initiate the assembly and growth of a multimeric signaling platform on the outer mitochondrial membrane, which includes proteins from the TRAF and TRIM families (Hou et al., 2011). The MAVS signalosome activates both type I interferon and nuclear factor κB (NF-κB) inflammatory responses (Hou et al., 2011, Kato et al., 2006, Kato et al., 2008). The assembly of MDA5 filaments on dsRNA also efficiently displaces viral proteins from the RNA while promoting dsRNA-binding and activation of protein kinase R (Yao et al., 2015), which leads to inhibition of protein translation and hence virus replication (Chung et al., 2018). This effector activity of MDA5 is ATP dependent but CARD independent (Yao et al., 2015).

Recently, it was shown that mRNA containing Alu repeats, endogenous retroelements of viral origin constituting 10% of the human genome, can hybridize into long RNA duplexes that must be deaminated by ADAR1 to avoid recognition by MDA5 (Ahmad et al., 2018). A-to-I deamination by ADAR1 destabilizes the of Alu:Alu duplex sufficiently to prevent MDA5 filament formation. Gain-of-function MDA5 mutations or ADAR1 deficiency can cause PKR-mediated translational shutdown and severe autoimmune disorders, including Aicardi-Goutières syndrome (AGS), Singleton-Merten syndrome (SMS), and other interferonopathies (Ahmad et al., 2018, Chung et al., 2018, Rice et al., 2014, Rutsch et al., 2015).

Crystal structures show that MDA5 binds dsRNA oligonucleotides with a modified DExD/H-box helicase core and a C-terminal domain (CTD) (Uchikawa et al., 2016, Wu et al., 2013). The helicase consists of two RecA-like domains, Hel1 and Hel2, and an insert domain, Hel2i, all of which form contacts with phosphate and ribose moieties of both RNA strands. The helicase and CTD, linked by a pair of α helices referred to as the pincer domain, form a closed ring around the RNA. The overall structure is similar to those of two other helicases in the same subfamily, RIG-I and LGP2 (Jiang et al., 2011, Kowalinski et al., 2011, Luo et al., 2011, Uchikawa et al., 2016). However, the CTDs of RIG-I and LGP2 bind dsRNA blunt ends, with 5′-di- or triphosphate caps and unphosphorylated, respectively, and both proteins have a much lower propensity than MDA5 to form filaments (Devarkar et al., 2016, Goubau et al., 2014, Luo et al., 2012). A structure of MDA5 bound to viral dsRNA determined by negative-stain electron microscopy at 22 Å resolution showed that MDA5 forms a polar, single-start helix on dsRNA and suggested that in MDA5 the CTD participates in filament formation rather than dsRNA blunt end recognition, but the resolution was insufficient to identify specific intermolecular interfaces (Berke et al., 2012).

The dsRNA binding cooperativity and length specificity of MDA5, which are critical for its signaling activity, are encoded by the filament-forming interfaces, but these remain unknown. Here, we determined the structures of the MDA5-dsRNA filament by cryoelectron microscopy (cryo-EM) in different helical twist states and nucleotide-binding states at local resolutions of up to 3.42 Å, allowing us to build and refine atomic models of the filaments. The structures reveal a predominantly hydrophobic pair of filament-forming interfaces with the requisite flexibility to accommodate the mechanical properties of dsRNA (Herrero-Galán et al., 2013). Structures bound to ATP, ground-state analog AMPPNP, transition-state analog ADP-AlF_4_, and no nucleotide show how the ATPase cycle is coupled to changes in helical twist and the mode of dsRNA binding of MDA5. This work shows how MDA5 recognizes long dsRNA ligands and provides a structural basis for its proposed proofreading activity.

## Results

### MDA5-dsRNA Filaments Have a Variable Helical Twist

**Figure 1:**
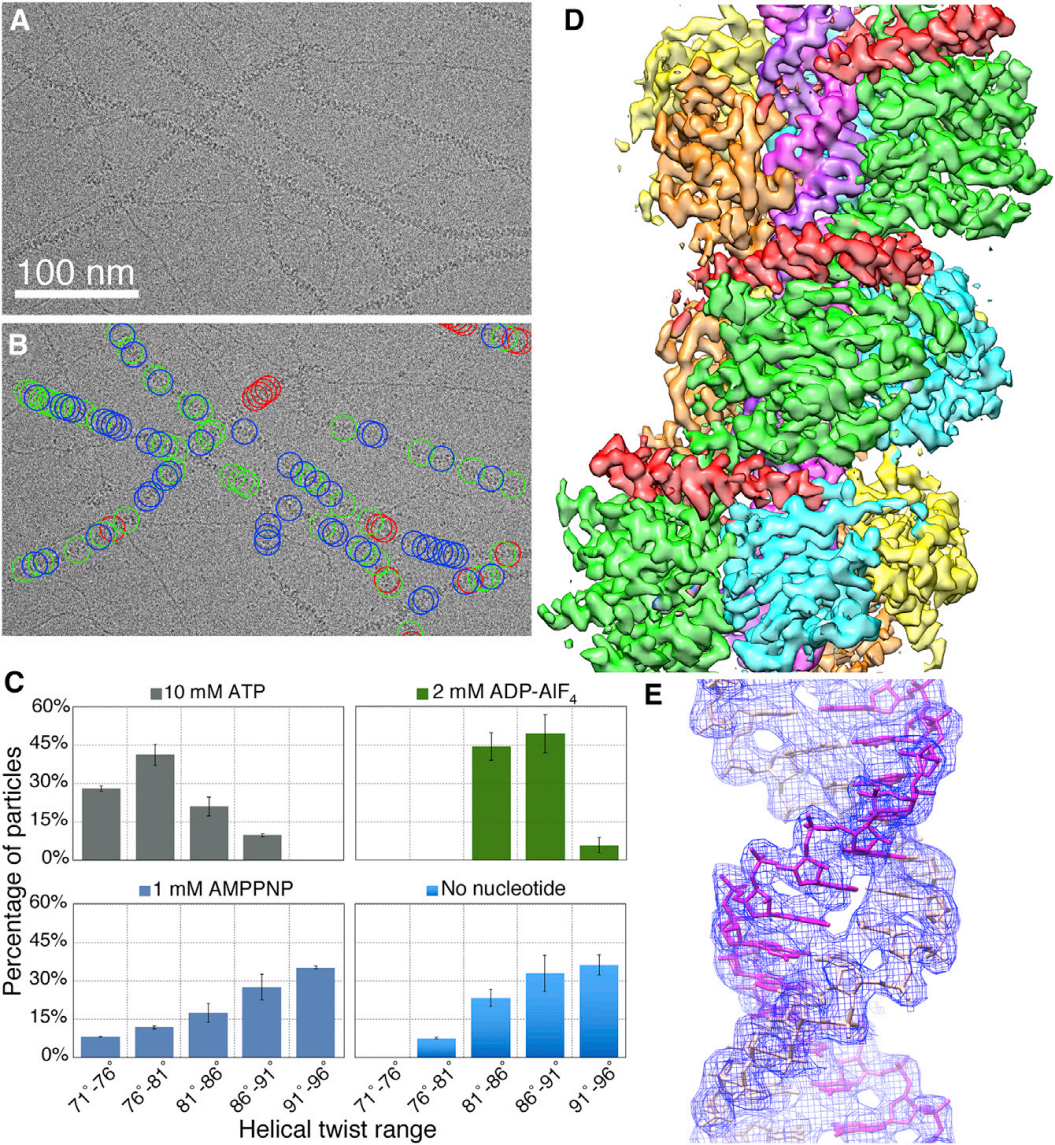
Cryo-EM Image Reconstruction of MDA5-dsRNA Filaments with Helical Symmetry Averaging (A) Representative cryo-EM micrograph of MDA5-dsRNA filaments. (B) Cryo-EM micrograph shown in (A) with circles drawn around the boxed filament segments that were used in the helical reconstructions. The circles are colored according to the 3D class that they contributed to. Segments that contributed to the Twist74, Twist87, and Twist91 structures are in red, green, and blue, respectively. (C) Histogram showing the distributions of filament segments as a function of helical twist for the ATP, ADP-AlF_4_, 1-mM AMPPNP, and nucleotide-free datasets. The distributions shown are from 3D classification performed with ten classes per dataset. Error bars represent SEM between replicate 3D classification calculations; n = 3. (D) 3D density map of the Twist74 MDA5-dsRNA filament at 3.68 Å overall resolution. The components are colored as follows: Hel1, green; Hel2, cyan; Hel2i, yellow; pincer domain, red; CTD, orange; and RNA, magenta. (E) The dsRNA density in the Twist74 filament (blue mesh) is shown with the fitted atomic model (magenta and pink). See also Figures S1 and S2.

MDA5-dsRNA filaments were assembled by incubating recombinant mouse MDA5 with dsRNA in the presence of either ATP, the nonhydrolyzable ATP (ground state) analog AMPPNP, or the transition state analog ADP-AlF_4_. A concentration range of 1–10 mM of nucleotide was selected for a favorable tradeoff between filament stabilization and vitreous ice formation, while remaining near the physiological range of cellular ATP concentration. Residues 646–663, in a flexible surface loop of Hel2i, were deleted to improve solubility, resulting in a 114-kDa polypeptide chain. This “ΔL2” deletion did not interfere with the ATPase, dsRNA binding or interferon signaling activities of MDA5 (Berke et al., 2012, Wu et al., 2013). Power spectra from raw cryo-EM images showed meridional reflections confirming the previously determined helical rise of ∼44 Å (Berke et al., 2012; Figure S1). The high average curvature and variability in helical twist of the filaments presented challenges for helical image reconstruction. A cylinder was used as the initial model for 3D image reconstruction, along with the experimentally determined helical symmetry parameters (STAR Methods; Berke et al., 2012). To deal with sample heterogeneity, particles were divided into several classes during 3D image reconstruction. Most of the variability between classes was in the helical twist. There was no evidence for discrete twist states, and the number of classes used was arbitrary. Individual filaments contained segments with different twists (Figure 1B). Segments of similar twist formed small clusters, indicating that the variability in twist was a local phenomenon. Some twist values occurred more frequently than others, and the twist distribution depended on type and occupancy the nucleotide bound (Figure 1C). Helical reconstruction of the filaments formed with 1 mM AMPPNP in RELION (He and Scheres, 2017) produced three maps with an overall resolution better than 4 Å (3.68–3.93 Å) (Table 1; Figures S2 and S3). The maps had distinct helical twists of 74°, 87°, and 91°, respectively, but similar rises (43–45 Å). With local resolutions up to 3.42 Å, the maps, referred to henceforth as Twist74, Twist87, and Twist91, were sufficiently detailed for atomic models to be built and refined into each map using the crystal structures of human and chicken MDA5 bound to dsRNA oligonucleotides as starting models (Uchikawa et al., 2016, Wu et al., 2013) (Figures 1D, 1E, and S3). Reconstructions of filaments formed with 2.5 mM AMPPNP, 10 mM ATP, 2 mM ADP-AlF_4_ and without nucleotide were subsequently obtained with resolutions of 3.87–4.06 Å. Filaments formed with ATP were frozen 7–8 min after addition of ATP to the sample, to prevent ATP hydrolysis from proceeding to completion. Most of the filaments formed with 10 mM ATP had low helical twist (71°–81°), and most of the filaments formed without nucleotide had high helical twist (91°–96°). The filaments formed with ADP-AlF_4_ had a narrower distribution of intermediate twists (81°–91°) (Figure 1C). Filaments formed with 2.5 mM AMPPNP had twists spanning a similarly broad range as with 1 mM AMPPNP (71°–96°).

**Table 1:** Cryo-EM Data Collection, Structure Determination, Model Building, and Refinement Parameters and Statistics

	Mouse MDA5 Bound to Phi6 dsRNA 1 mM AMPPNP			MDA5-dsRNA, 2.5 mM AMPPNP	MDA5-dsRNA, 10 mM ATP	MDA5-dsRNA, 2 mM ADP-AlF_4_	MDA5-dsRNA, No Nucleotide	
**Data collection and processing**								

Microscope	FEI Titan Krios			FEI Titan Krios	FEI Titan Krios	FEI Titan Krios	FEI Titan Krios	
Voltage (kV)	300			300	300	300	300	
Electron exposure (electrons Å^−2^)	29.85			29.33	30.24	29.85/30.50	27.0	
Exposure per frame (electrons Å^−2^)	0.398			0.391	0.403	0.398/0.272	1.00	
Defocus range (μm)	−1.8 to −2.7			−1.8 to −2.7	−1.7 to −3.1	−1.8 to −2.7	−1.8 to −2.7	
Pixel size (Å)	1.07			1.07	1.07	1.07/1.085	1.04	
No. of initial segment images	367,549			207,000	526,596	234,835	137,601	

**3D class average**	**Twist74**	**Twist87**	**Twist91**	**Twist77-2.5**	**Twist72-10**	**ADP-AlF**_**4**_	**Tw89-NoNt**	**Tw93-NoNt**

No. of final segment images	33,138	60,079	40,265	28,663	100,482	31,556	26,527	19,111
Map resolution range (Å)	240–3.68	240–3.93	240–3.93	240–4.06	240–3.87	240–4.06	240–4.02	240–4.16
Fourier shell correlation (FSC) threshold for resolution limit	0.143	0.143	0.143	0.143	0.143	0.143	0.143	0.143
Max. local resolution range	24.0–3.42	20.0–3.63	20.0–3.69	26.6–3.63	21.8–3.58	26.6–3.99	50.0–3.22	50.06–3.89

**Model refinement**								

Map sharpening B factor (Å^2^)	−175	−165	−175	−125	−175	−175	−150	−175
Helical symmetry imposed								
Twist (°)	74.302	87.369	90.921	76.776	72.817	87.832	89.000	93.060
Rise (Å)	42.844	44.510	44.970	43.106	43.062	46.511	44.242	44.366
Mask correlation coefficient	0.767	0.767	0.762	0.795	0.818	0.798	0.801	0.775

**Model composition**								

No. of non-hydrogen atoms	6,209	5,894	5,907	6,209	6,268	6,296	5,955	5,997
Protein residues	682	645	648	682	689	685	660	667
RNA nucleotides	28	30	30	28	28	30	30	30
Ligand	AMPPMP	–	–	AMPPMP	ATP	ADP-AlF_4_	–	–
Ions (Zn^2+^ or Zn^2+^ and Mg^2+^)	1	1	1	1	2	1	1	1

**RMSDs**								

Bond lengths (Å)	0.012	0.006	0.010	0.011	0.007	0.006	0.007	0.008
Bond angles (°)	1.093	1.000	1.242	1.050	0.896	0.938	0.820	0.872
Planarity (Å)	0.006	0.004	0.005	0.006	0.004	0.004	0.005	0.006

**B-factors and ADPs**								

Minimum	30.0	54.4	51.0	102	75.6	57.9	76.1	36.8
Maximum	100	170	156	263	187	159	198	145
Mean	56.0	103	100	167	115	95.4	136	78.8

**Validation**								

MolProbity overall score	1.71	1.79	1.83	1.77	1.65	1.74	1.71	1.76
MolProbity all-atom clash score	3.52	3.84	4.37	4.00	3.09	4.03	3.87	4.19
Rotamer outliers (%)	0	0	0	0	0.11	0	0.18	0.17
Ramachandran plot								
Favored (%)	89.4	87.3	87.3	88.8	90.1	90.0	90.5	89.5
Allowed (%)	10.3	12.7	12.7	10.9	9.7	10.0	9.5	10.3
Outliers (%)	0.3	0	0	0.3	0.2	0	0	0.2
PDB codes	PDB: 6G19	PDB: 6G1S	PDB: 6G1X	PDB: 6GJZ	PDB: 6GKM	PDB: 6GKH	PDB: 6H61	PDB: 6H66
EMDB codes	EMD-4338	EMD-4340	EMD-4341	EMD-0012	EMD-0024	EMD-0023	EMD-0143	EMD-0145
EMPIAR codes	10213	10213	10213	10209	10208	10211	10210	10210

See also Figures 1, 3, S2, and S3. ADPs, atomic displacement parameters.

### Overall Structure of the MDA5-dsRNA Filaments

**Figure 2:**
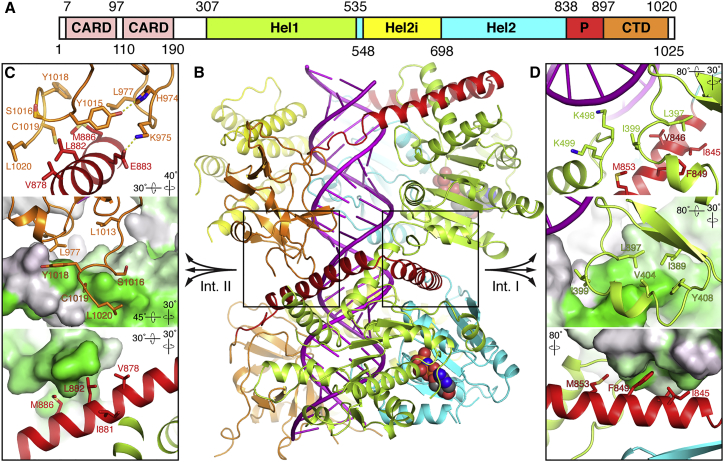
Atomic Model of the MDA5-dsRNA Filament (A) Domain structure of mouse MDA5. CARD, caspase recruitment domain; CTD, C-terminal domain; Hel1 and Hel2, first and second RecA-like helicase domains; Hel2i, Hel2 insert domain; P, pincer domain. The same color code and domain abbreviations are used in subsequent panels and in Figures 1, 7A, and 7D. (B) Overview of the refined atomic model of the MDA5-dsRNA filament. Two adjacent MDA5 subunits and 28 bp of dsRNA are shown from the Twist74 structure. RNA is in magenta. The bound AMPPNP molecules are shown in sphere representation. The two filament-forming interfaces are boxed. (C and D) Close-up views of filament interface II (C) and interface I (D). The top panels show side chains forming key contacts, with hydrogen bonds shown as yellow dashed lines. In the middle panels the lower protomer in (B) is shown in surface representation colored by hydrophobicity from gray to green, with green being the most hydrophobic. In the lower panels, the upper protomer in (B) is shown in surface representation colored by hydrophobicity. The orientation of the view relative to (B) is indicated for each panel. See also Figure S3 and Videos S1, S2, S3, S4, and S5.

**Figure 3:**
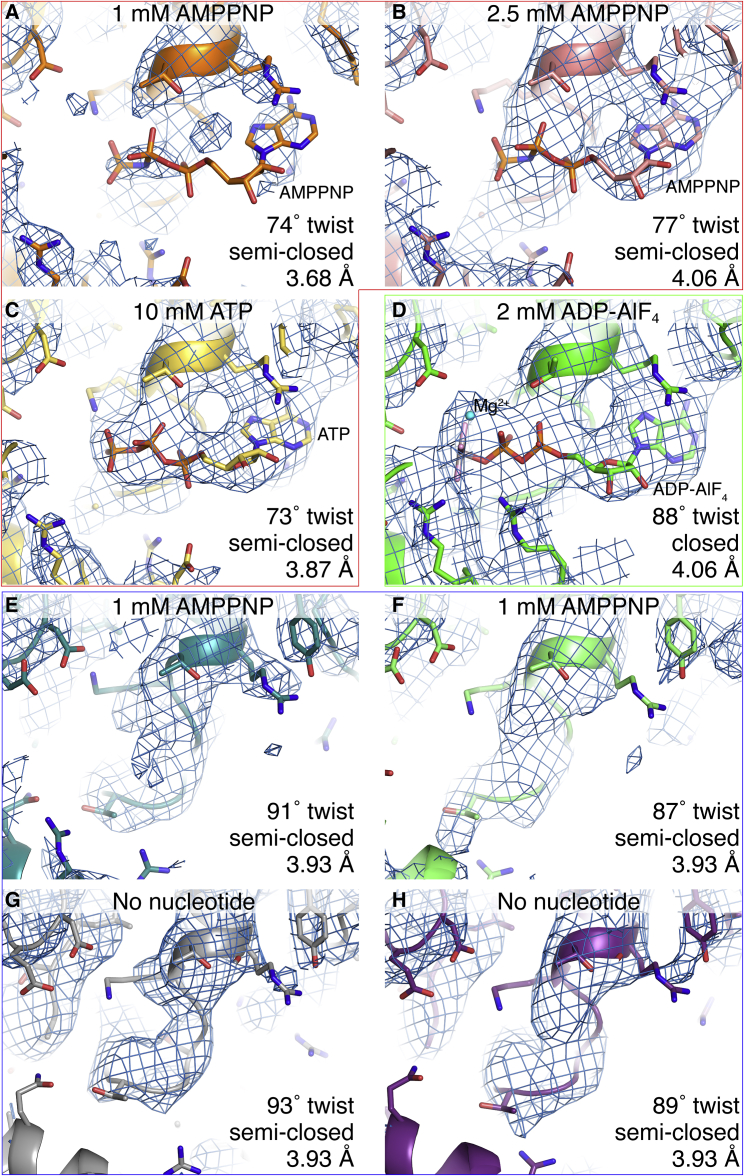
Close-up Views of the Cryo-EM Densities and Atomic Models around the ATP-Binding Site for Reconstructions with Different Helical Twists and Bound Nucleotides (A–C) Density consistent with a nucleotide triphosphate molecule is visible in the low-twist structures with 2.5 mM AMPPNP (B) and 10 mM ATP (C), but only weak density is visible in the low-twist (74°) 1 mM AMPPNP structure (A) (red outline). (D) With 2 mM ADP-AlF_4_, strong density is visible for the ADP and AlF_4_ moieties (green box). The AlF_4_ moiety shown in pink and gray and a coordinated Mg^2+^ ion in cyan. (E–H) With 1–2.5 mM AMPPNP (E and F) or no nucleotide (G and H), there is no nucleotide density in the catalytic site of the structures with mid- to high helical twist (81°–96°, blue box). A contour level of 4.5 σ in PyMol was used for all panels. The AMPPNP, ATP, and ADP-AlF_4_ molecules and selected protein side chains are shown in stick representation. See also Figure S3.

In the cryo-EM structures presented here, MDA5 forms a closed ring around the RNA (Figure 2; Video S1). The low-twist structures (71°–81°) contain 14 bp of dsRNA in the asymmetric unit and density for nucleotide in the ATP-binding site. The intermediate-twist (81°–91°) and high-twist (91°–96°) structures have 15 bp of dsRNA and no interpretable density in the catalytic site. The nucleotide density ranges from absent or weak in the nucleotide-free structures and the 1-mM AMPPNP low-twist structure (Twist74), respectively, to strong in the intermediate-twist ADP-AlF_4_ structure. The 2.5-mM AMPPNP and 10-mM ATP structures have nucleotide densities of intermediate amplitude (Figure 3). The nucleotide density in the 10-mM ATP structure is unambiguously more consistent with ATP than with ADP or ADP:Mg^2+^ (Figure 3C), suggesting that the low-twist filament segments used in the 10-mM ATP reconstruction predominantly contained ATP that remained unhydrolyzed when the sample was frozen. The Hel1 and Hel2 domains are in the semi-closed state, as defined by Uchikawa et al. (2016) in all structures except the ADP-AlF_4_-bound structure, which is in the closed state. Two out of six ATP-binding helicase motifs, motifs Q and I as defined by Jiang et al. (2011), are engaged with nucleotide in the low-twist semi-closed structures. All six ATP-binding helicase motifs (motifs Q, I, II, III, Va, and VI) are engaged with ADP-AlF_4_ in the closed structure.

**Figure 4:**
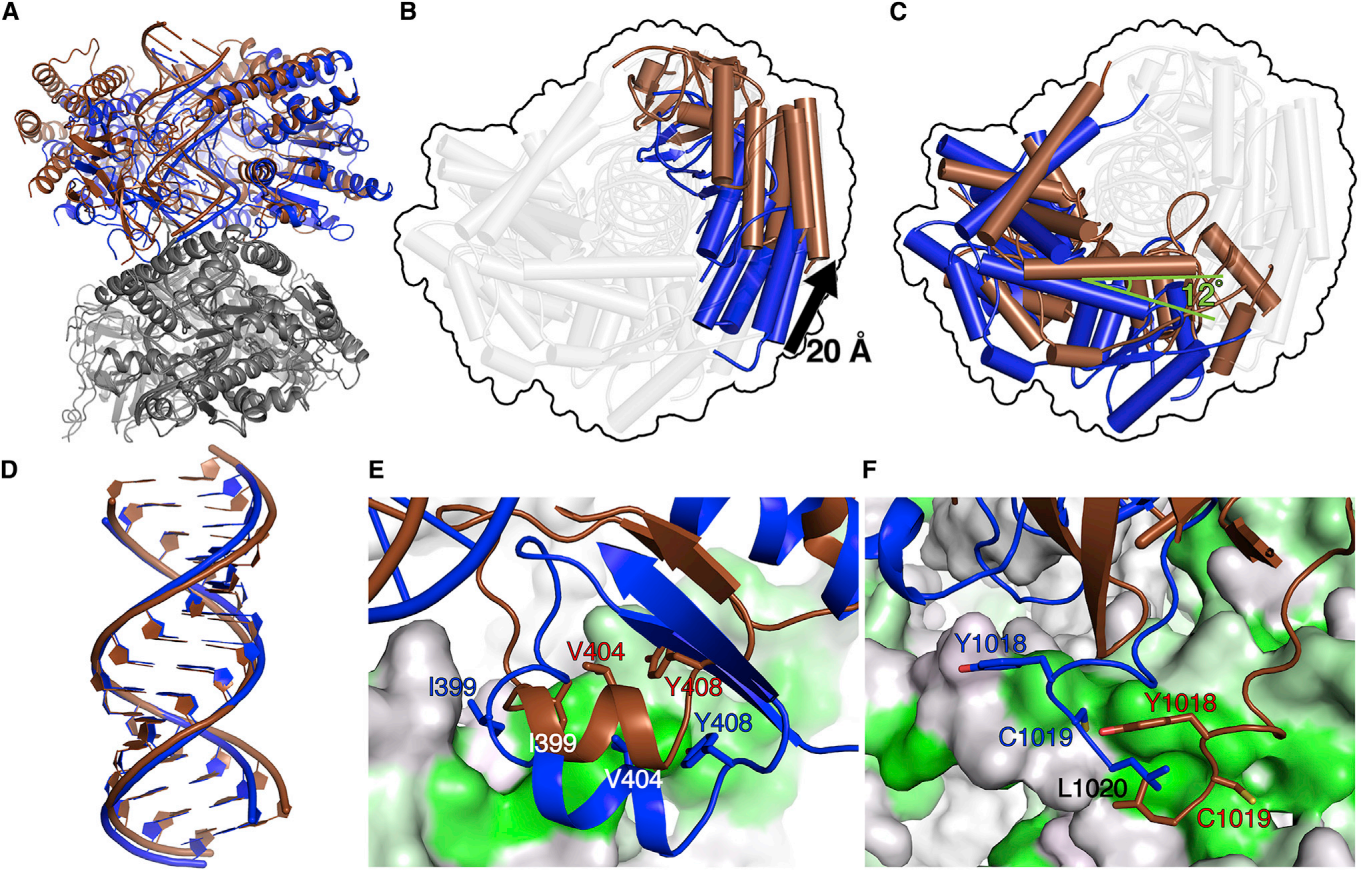
Differences in Relative Domain Positions and Filament Contacts in the Twist74 and Twist91 Structures (A) Overview of two protomers of the Twist74 and Twist91 structures superimposed using the pincer domain of the lower protomer (gray) as the reference. The upper protomer of Twist74 is in blue and that of Twist91 is in brown. (B and C) Top views along the helical axis of the upper protomer from (A) showing the shifts in the positions of Hel2i and CTD (B), and Hel1, Hel2, and pincer (C). The 20 Å translation in Hel2i and 12° rotation in the pincer domain are highlighted. The highlighted domains are colored as in the upper protomer in (A), and the remaining domains are shown in transparent gray for clarity. The outer contour of the superimposed structures is shown for reference as a black outline. (D) Close-up of the dsRNAs from the Twist74 and Twist91 structures from the structural alignment in (A). The RMSD of the atoms in the 14 superimposed RNA base pairs is 1.23 Å. (E and F) Close-up views of filament interface I (E) and interface II (F). The protomer of Twist74 shown in gray in (A) and used as the alignment reference is shown in surface representation colored by hydrophobicity as in Figure 2. Key interface residues in the adjacent protomer are shown with Twist74 in blue and Twist91 in brown. See also Figures S4–S6 and Videos S1, S2, S3, S4, and S5.

Video S1. Atomic Models of the MDA5-dsRNA Filaments in Four Different States, Related to Figures 2, 4, and 7 Morphing between overviews of the refined atomic models of the MDA5-dsRNA filaments in the four states shown in Figure 7D: low-twist AMPNPN-bound (Twist74), intermediate-twist ADP-AlF_4_-bound, intermediate-twist low nucleotide-occupancy (Twist87) and high-twist low nucleotide-occupancy (Twist91). Three adjacent MDA5 protomers and bound dsRNA are shown. RNA is in magenta. The bound AMPPNP molecules are shown in ball-and-stick representation. The domains of the central MDA5 protomer are colored as in Figures 1, 2, and 7: Hel1, light green; Hel2, cyan; Hel2i, yellow; pincer, red; C-terminal domain, orange. The two adjacent MDA5 protomers are shown in gray.

The asymmetric units of the three twist classes of filaments have a similar overall structure to the dsRNA-bound crystal structures of MDA5 (Figure S4). However, the cryo-EM structures contain several features not present the crystal structures, most notably the C-terminal tail of the CTD (residues 1,014–1,020), which extends toward an adjacent subunit and forms key filament contacts. The cryo-EM structures also contain a short acidic loop in Hel1 (residues 428–430), the linker between the pincer domain and the CTD (residues 894–899), and part of an extended loop within the central β sheet of the CTD (residues 945–949). Some of these features are present but have different conformations in the crystal and nuclear magnetic resonance (NMR) structures of the MDA5 CTD alone (PDB: 3GA3; Takahasi et al., 2009). The CARDs were not visible in any of the density maps after helical symmetry averaging, even at low contour levels, indicating that the CARDs do not share the helical symmetry of the MDA5-dsRNA filament. Since CARD oligomers could not be distinguished in the raw micrographs, we conclude that the oligomers are no larger than ∼100 kDa, the current size limit for cryo-EM single-particle imaging, which corresponds to the tandem CARDs of six to eight MDA5 molecules.

### Identification of the Filament-Forming Interfaces

The cryo-EM structures identify the filament-forming surfaces of MDA5. The majority of the contacts are hydrophobic and can be grouped into two interfaces, both involving the pincer domain (Figure 2). Interface I is formed by a loop in Hel1 (residues 395–408), which forms an extensive set of hydrophobic interactions with the first α helix of the pincer domain (residues 839–864) and an adjacent loop in Hel1 (residues 497–500) of the adjacent subunit (Figure 2D; Video S2). The most notable interactions are hydrophobic side-chain contacts between Leu397, Ile399, Ser400, Glu403, and Val404 in Hel1 and Glu842, Ile845, Val846, Phe849, and Met853 in the pincer helix. These side chains form the core of interface I, with a buried surface area of 452–570 Å^2^ in the semi-closed structures and 413 Å^2^ in the closed ADP-AlF_4_-bound structure.

Video S2. Comparison of Filament-Forming Interface I in Four Different States, Related to Figures 2, 4, and 7 Zoom from overview to close-up of filament forming interface I, with morphing between the refined atomic models of the Twist74, ADP-AlF_4_-bound, Twist87 and Twist91 structures in the close-up view. Three adjacent MDA5 protomers and bound dsRNA are shown and colored as in Video S1.

Interface II is formed by the C-terminal tail, which extends from the CTD to form hydrophobic contacts with the second pincer helix (residues 866–891) of the adjacent subunit (Figure 2C; Video S3). The core contacts of the interface are hydrophobic side-chain contacts between Asp1014, Tyr1015, Tyr1018, and Cys1019 in the C-terminal tail, along with Gly976 and Leu977 in the CTD and Gln879, Leu882, Glu883, and Met886 in the pincer domain. Glu883 also forms polar contacts with either Lys975 or Gly976, depending on the twist class. The C-terminal tail was not resolved in crystal structures of human and chicken MDA5 bound to dsRNA oligonucleotides, indicating that the structure of the C-terminal tail observed by cryo-EM is dependent on head-to-tail intersubunit filament contacts. The surface area buried by interface II is 413–433 Å^2^ in the semi-closed structures and 471 Å^2^ in the ADP-AlF_4_-bound structure. Thus, whereas interface I buries a larger area than interface II in the semi-closed structures, the opposite is true in the closed ADP-AlF_4_ structure.

Video S3. Comparison of Filament-Forming Interface II in Four Different States, Related to Figures 2, 4, and 7 Zoom from overview to close-up of filament forming interface II, with morphing between the refined atomic models of the Twist74, ADP-AlF_4_-bound, Twist87 and Twist91 structures in the close-up view. Three adjacent MDA5 protomers and bound dsRNA are shown and colored as in Videos S1 and S2.

The residues listed above as forming interfaces I and II are conserved or similar in MDA5, but not RIG-I sequences from vertebrate species (Figure S5). The following residues are strictly conserved across terrestrial vertebrates in MDA5, but not RIG-I: 397–403, 497–500, and 846 in interface I and 879, 883, 886, 976, 977, 1,015, and 1,019 in interface II.

**Figure 5:**
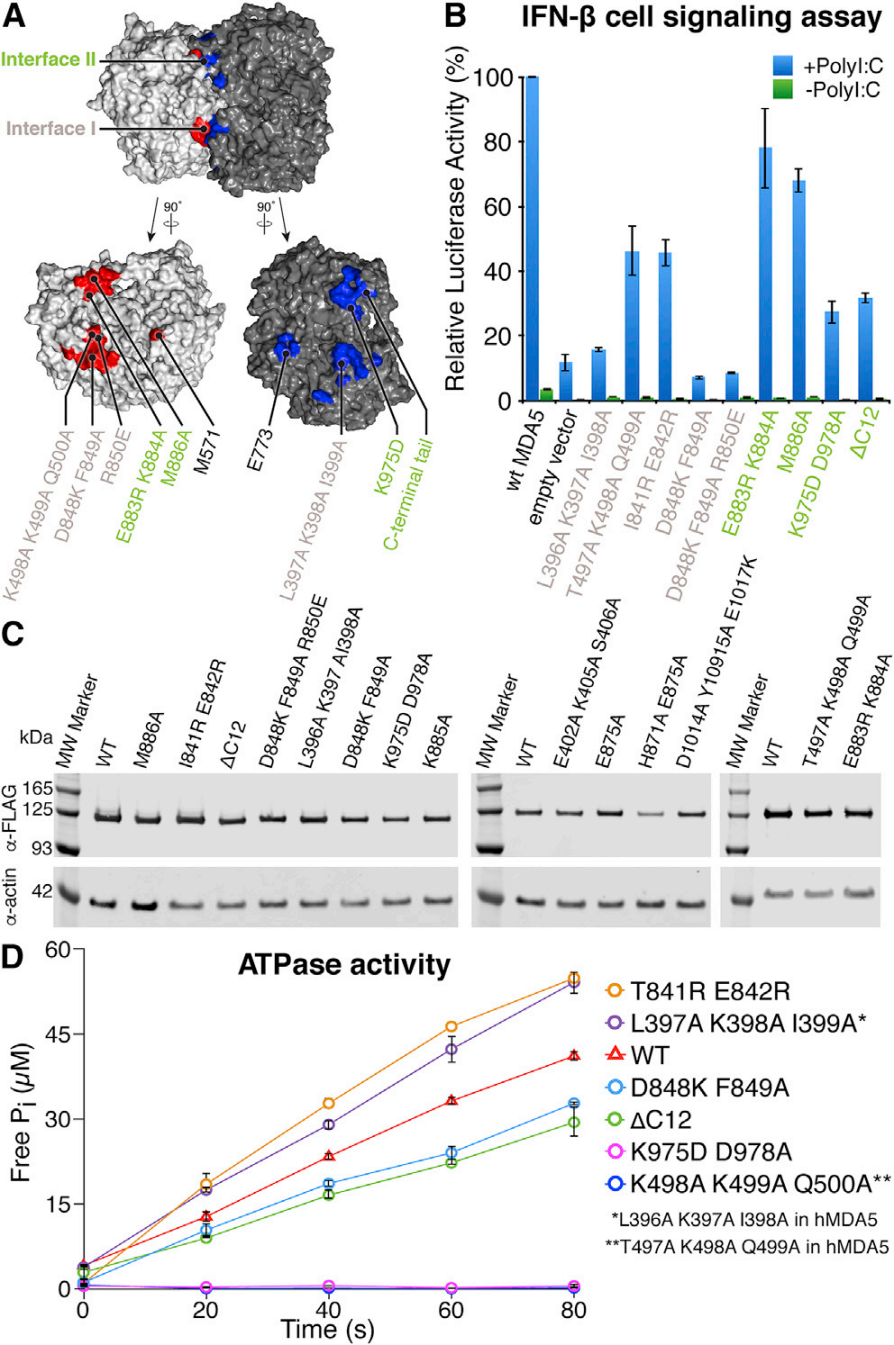
Mutations at the Filament-Forming Interfaces Abolish or Reduce Cell Signaling in Response to dsRNA (A) Location of the engineered filament interface mutations. Two filament protomers are shown in surface representation with the filament-forming surfaces of each protomer colored in red and blue, respectively. The protomers are shown assembled with the helical axis horizontal (top) and separately after being opened like a book with 90° rotations in opposite directions to show the interface surfaces (bottom). Residue labels are colored pink for interface I and green for interface II. Residue numbers refer to mouse MDA5. (B) IFN-β reporter cell signaling assay. Plasmids encoding human MDA5 mutants were co-transfected into HEK293 cells with plasmids encoding firefly luciferase under an IFN-β-inducible promoter and *Renilla* luciferase under a constitutive promoter. Cells were later transfected with poly(I:C) RNA (+PolyI:C) or DMEM (−PolyI:C). Relative luciferase activity was calculated as the ratio of firefly to *Renilla* luciferase luminescence. Residue numbers refer to human MDA5. Error bars represent SEM between measurements; n = 3. (C) Western blots showing the expression level of the human MDA5 mutants in HEK293T cells. The FLAG tag on each MDA5 variant was detected with an anti-FLAG antibody. (D) ATP hydrolysis assay. The ATPase activities of MDA5 mutants with reduced signaling activity were measured as release of inorganic phosphate (P_i_) on incubation with ATP and 1-kb dsRNA. Error bars represent SEM; n = 3. See also Figure S7.

A minor filament contact point is formed by Met571 in Hel2i, which forms hydrophobic side-chain contacts with Glu773 from the Hel2 domain of the adjacent subunit (Figure 5A). The contact area of this interface is small, 141 Å^2^ in Twist74, and only 42–65 Å^2^ in the higher-twist classes, representing 4%–13% of the total filament interface area. This interface is absent the ADP-AlF_4_-bound structure, and Met571 is not strictly conserved in mammalian MDA5 sequences.

### Flexible MDA5 Interfaces Lead to Variable Helical Symmetry

The extent to which the MDA5 filament-forming interfaces are predominantly hydrophobic in nature is striking. Hydrophobic interfaces can be intrinsically structurally flexible and allow intersubunit rotation in the absence of the chemical and geometric restraints imposed by polar hydrogen bonds or salt bridges (Li et al., 2005). Comparison of the filament-forming interfaces in the different twist classes shows that although the interfaces are broadly conserved, there are significant differences in how they form in each twist class. Superposition of the Twist74 and Twist91 structures using the Hel1 domain of a specific subunit as the reference (root mean square deviation [RMSD] 0.75 Å) highlights how variable and flexible the filament-forming interfaces are, with differences of up to 20 Å in the resulting positions of the domains of adjacent filament subunits (Figure 4). This large variability in the orientation of the adjacent subunits within the filament is possible specifically due to the structural versatility of the Hel1 loop component of interface I (residues 395–408) and of the C-terminal tail component of interface II (residues 1,014–1,020). These components have significantly different conformations in each twist class, each adapting their structure to maintain hydrophobic contacts with the apposed pincer domain helix from the adjacent subunit (Figures 4E, 4F, and S6). The interface components within the pincer domain, constrained by helical secondary structure, have the same local conformation in the different twist classes, though the pincer helices, which pack tightly onto the Hel1 and Hel2 domains, follow the larger shifts in domain positions mentioned above. The specific hydrophobic contacts formed in the different twist classes are similar, but not identical. For example, the C-terminal tail forms contacts two helical turns further down the second pincer helix in the Twist74 structure than in the Twist91 structure, and the Hel1 interface loop adopts a different conformation in Twist74 than in the other cryo-EM and crystal structures (Video S4). The net result is that the Hel1 interface loop functions as a flexible finger and the C-terminal tail as a flexible arm, allowing similar, but not identical, sets of hydrophobic contacts to be maintained between subunits. This bears similarity to the capsid proteins of some spherical viruses, which have flexible C-terminal arms and internal loops that allow the quasi-equivalent assembly of capsomeres in slightly different symmetry environments within the icosahedral lattices of viral capsids (Liddington et al., 1991).

Video S4. Comparison of Filament-Forming Interfaces in the Different Twist States of the 1-mM AMPPMP Structures with Side-Chain Detail, Related to Figures 2, 4, and 7 Zoom from overview to close-up of the two filament forming interfaces, with morphing between the refined atomic models of the Twist74, Twist87 and Twist91 structures in close-up views showing the key filament forming side chains. Three adjacent MDA5 protomers and bound dsRNA are shown and colored as in the other videos.

### Functional Importance of MDA5 Interfaces in Cell Signaling

The filament interfaces are critical to MDA5 function because they encode the dsRNA-binding cooperativity of MDA5 (Berke and Modis, 2012, Peisley et al., 2011). Hence, the filament interfaces encode the propensity of MDA5 to form long filaments on RNA and signal more actively from longer dsRNAs. To test the functional importance of the filament-forming interfaces revealed in our cryo-EM structures, we examined the effect of structure-based mutations targeting the interfaces in cell-signaling assays and filament-forming assays. A panel of 16 human MDA5 variants was generated, with each variant bearing one, two, or three structure-based point mutations at one of the filament-forming interfaces. Expression plasmids were individually co-transfected into HEK293 cells together with plasmids encoding firefly luciferase under control of the interferon β (IFN-β) promoter and *Renilla* luciferase under a constitutive promoter. After expression for 6 hr, cells were transfected with poly(I:C) RNA to induce MDA5 signaling. IFN-β-dependent induction of firefly luciferase was measured in cell lysates 24 hr post-induction, as firefly luciferase luminescence normalized against *Renilla* luciferase luminescence (Figure 5). The expression level of each MDA5 variant in HEK cells was assessed by western blotting (Figure 5C). With a few exceptions discussed below, the mutations significantly reduced or abolished luciferase signaling. The following mutations reduced luciferase activity down to background levels: L396A/K397A/I398A in Hel1 and D848A/F849A, targeting other interface I contacts listed above (some residue numbers are different in human and mouse MDA5; Figure S5). Deletion of the C-terminal tail (ΔC12) and CTD mutations K975D/D987A, targeting interface II contacts, resulted in a 4-fold reduction in signaling (Figure 5B). A triple point mutation in the C-terminal tail (D1014A/Y1015A/E1017K), also targeting interface II, caused a more modest 2- to 3-fold reduction in signaling (Figure S7B). T497A/K498A/Q499A, in interface I, resulted in a 2-fold reduction in luciferase activity. The M886A mutation caused a 30% reduction in signaling. The E883R/K884A and K885A mutations, targeting interface II contacts with the C-terminal tail and CTD, had no effect on signaling.

The mutant I841R/E842R was shown previously to partially inhibit filament formation of human MDA5 on 1-kb dsRNA and to reduce binding affinity for 112-bp, but not 15-bp, dsRNA (Wu et al., 2013). This pair of mutations maps to the periphery of the pincer helix component of interface I, with Glu842 forming hydrophobic contacts with Thr395 or Leu397 in the Hel1 interface loop. Ile841 does not form any interface contacts, however, and is not conserved in mouse and chicken MDA5. The I841R/E842R mutation resulted in a 2.5-fold reduction of signaling, consistent with a moderate role of these residues, most likely Glu842, in filament formation (Figure 5B). The variant E402A/K405A/S406A had 77% of the signaling activity of wild-type (WT) MDA5, suggesting that the C-terminal portion of the Hel1 interface loop (residues 395–408) plays only a minor role in filament formation (Figure S7B).

Two variants unexpectedly caused slight increases in signaling activity: E875A and H871A/E875A (Figure S7B). His871 and Glu875 are in the second pincer helix, and their side chains form an intramolecular hydrogen bond in the Twist74 and Twist91 structures. The two residues form interface II contacts only in the Twist87 and Twist91 structures, with the C-terminal tail of the adjacent subunit. Filaments formed by the E875A variant formed aggregates (Figure S7A). We speculate that the increase in signaling upon loss of this glutamate side chain, which is conserved in terrestrial vertebrates, may arise from the collapse of filaments into three-dimensional filamentous aggregates.

**Figure 6:**
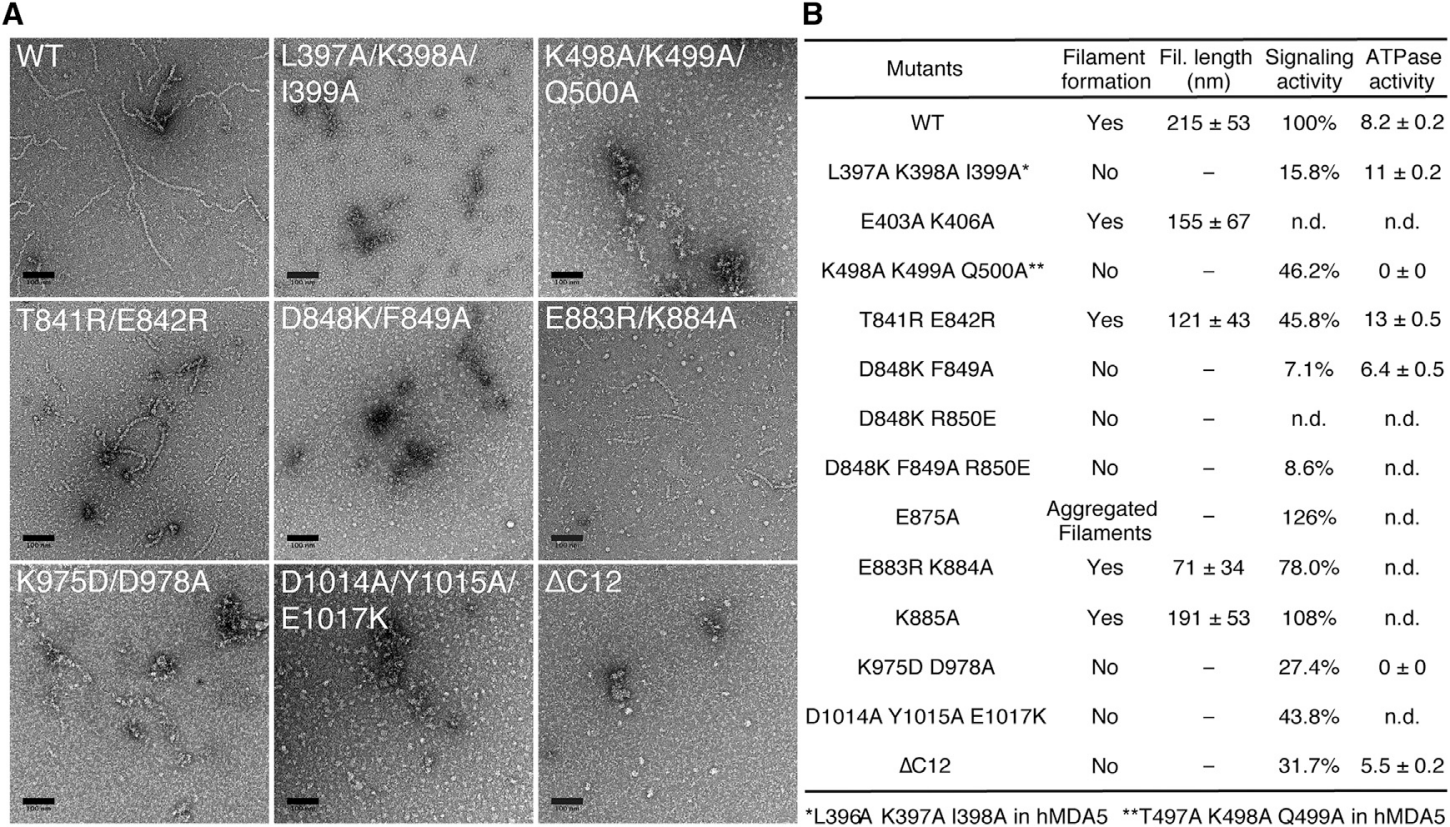
Interface Mutations that Impair Signaling Also Impair Filament Formation (A) Representative electron micrographs of MDA5 filament interface mutants in the presence of 1 kb dsRNA, 1 mM AMPPNP, and 5 mM MgCl_2_. Scale bars, 100 nm. Residue numbers refer to mouse MDA5. (B) Table summarizing the filament formation activity, filament length, cell-signaling activity, and ATPase activity of selected MDA5 mutants. ATPase activities were calculated from the initial slopes of the curves in Figure 5D and is expressed as moles of released phosphate per mole of MDA5 per second (M_Pi_ M_MDA5_^−1^ s^−1^). Residue numbers refer to mouse MDA5. For mutants with different residue numbers in human MDA5, the corresponding mutation is shown in human residue numbers at the bottom. n.d., not determined. See also Figures S5 and S7.

To determine whether mutations in the MDA5 filament interfaces affected ATPase activity, we assayed the ATP hydrolysis activity of the mutants with reduced signaling activities. Mouse MDA5 variants L397A/K398A/I399A, T841R/E842R, D848K/D849A, and ΔC12 had activities comparable to WT MDA5, ranging from 5.5 to 13 M_ATP_ M_MDA5_^−1^ s^−1^ (Figures 5D and 6B). K498A/K499A/Q500A and K975D/D978A had no ATPase activity. However, the mutations in these variants are distant from the ATP-binding site, and both mutants appeared to bind Mant-AMPPNP, a fluorescent ATP analog, with affinity comparable to WT MDA5 (Figure S7E), although the equilibrium binding constants could not be determined due to limitations in protein solubility. In contrast, mutations in the nucleotide-binding motifs reduce or abolish ATPase activity but increase signaling activity. For example, R337G, from a patient with elevated interferon and neuropathic symptoms, and G821S, which causes lupus-like autoimmune symptoms in mice, both constitutively activate MDA5 signaling in the absence of infection and abrogate ATPase activity (Funabiki et al., 2014, Rice et al., 2014). Similarly, R822Q, a common missense mutation in nucleotide-binding motif VI, increases constitutive and RNA-stimulated signaling and is associated with Singleton-Merten syndrome (Rutsch et al., 2015).

### Interface Mutations that Impair Signaling Hinder Polymerization

Cell signaling data show that mutation of MDA5 filament-forming residues predominantly results in loss of interferon-signaling activity. To determine whether these changes in signaling activity were due to corresponding changes in the efficiency of filament assembly, we purified 15 mouse MDA5 mutants selected from the panel of human MDA5 mutants assayed for cell signaling. All variants were expressed in quantities similar to WT MDA5 and had similar solubilities and hydrodynamic radii consistent with a monodisperse monomeric population (Figures S7C and S7D). We can therefore exclude the possibility that the loss of signaling activity observed in most interface mutants was due to gross destabilization of the protein fold. We then assessed the ability of each variant to assemble into filaments on 1 kb dsRNA in the presence of 1 mM AMPPNP, using filament formation in negatively stained electron micrographs as the readout (Figure 6A). We found a strong correlation between loss of signaling activity and loss of filament formation. Variants bearing mutations that completely abolished signaling or reduced signaling by at least 4-fold (including K498A/K499A/Q500A, D848A/F849A, L397A/K398A/I399A, K975D/D987A, and ΔC12) all failed to form filaments. D1014A/Y1015A/E1017A, which caused a 2.5-fold reduction in signaling, also failed to form filaments. T841R E842R, which also caused a 2.5-fold reduction in signaling, formed filaments half as long as WT MDA5 (Figure S7A). Among the least impaired variants, E403A/K406A formed filaments that were 27% shorter than WT MDA5, correlating well with the 23% reduction of signaling observed for the related mutant E402A/K405A/S406A.

To determine whether filament formation was strictly dependent on dsRNA, we imaged WT MDA5 and several of the mutants in the absence of dsRNA and with 1 mM AMPPNP. None of them formed filaments, including the hyperactive variant E875A, which remained monomeric in the absence of RNA (Figure S7C). In conclusion, mutation of MDA5 filament-forming residues predominantly results in loss of cellular MDA5 signaling activity. Most mutations inhibit MDA5 filament formation and abolish cellular interferon signaling activity without significantly affecting ATPase activity. Two variants (K498A/K499A/Q500A and K975D/D978A) also lack ATPase activity. One pair of mutations (H871A/E875A) appears to increase signaling activity by promoting the collapse of filaments into filamentous aggregates.

### MDA5-RNA Contacts and How They Vary across the Twist and Nucleotide States

The dsRNA-binding interfaces vary in the different twist classes. The protein-RNA contact area decreases as twist increases, with 2,315 Å^2^ for Twist74, 2,102 Å^2^ for Twist87, and 1,926 Å^2^ for Twist91. The ADP-AlF_4_-bound structure, which has a twist of 88°, has a protein-RNA interface of 2,152 Å^2^, similar to Twist87. The CTD forms stronger RNA contacts in the Twist74 structure than in the Twist91 structure (849 Å^2^ versus 471 Å^2^ contact areas), whereas the opposite is true for the Hel2i domain (293 Å^2^ versus 321 Å^2^). The intermediate-twist ADP-AlF_4_-bound structure is more similar to Twist74 in how its CTD and Hel2i domain bind RNA, with contact areas of 751 Å^2^ and 231 Å^2^, respectively. Within Hel2i, Gln581 is positioned in the Twist87 and Twist91 structures to form hydrogen bonds with either or both bases of an RNA base pair. Within the CTD, Ile923, Glu924, and Met926 form hydrophobic contacts with the RNA backbone in the Twist74 structure; the side chain of His927 forms hydrogen bonds with the O2_-_ or N2 atom of a pyrimidine base and with the ribose hydroxyl group (the latter is present in all three twist classes). The CTD capping loop (residues 944–953), so called because it binds to RNA blunt ends in RIG-I and LGP2 (Li et al., 2009, Pippig et al., 2009, Wang et al., 2010), is disordered in the Twist87 and Twist91 structures and in the MDA5-dsRNA crystal structures but partially ordered in the Twist74 structure, in which it forms contacts with the RNA backbone at the minor groove, as had been predicted (Uchikawa et al., 2016). By contrast, in structures of the MDA5 CTD alone (PDB: 3GA3; Takahasi et al., 2009), the capping loop and flanking residues 947–953 extend the central β sheet of the CTD, forming an additional pair of β strands. The resulting conformation is incompatible with dsRNA binding, suggesting that dsRNA binding causes the capping loop to peel off from the central β sheet.

**Figure 7:**
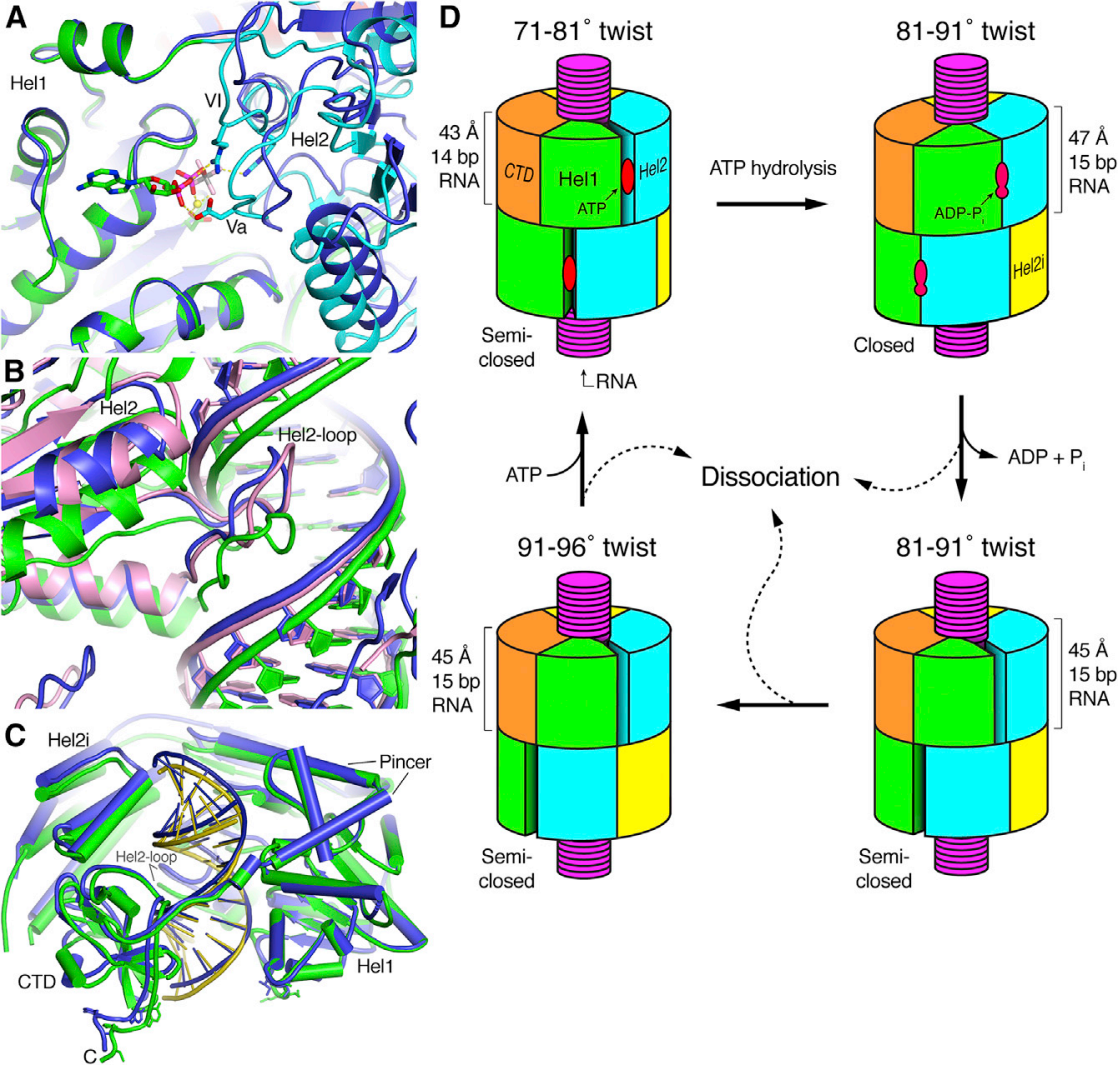
Comparison of the Closed ADP-AlF_4_-Bound Structure with the Semi-open Structures and Schematic Model of the ATPase Cycle and Proofreading Mechanism of MDA5 For a Figure360 author presentation of Figure 7, see https://doi.org/10.1016/j.molcel.2018.10.012. (A) Close-up view of the nucleotide-binding site and Hel1-Hel2 domain interface. The Twist74 AMPPNP-bound structure (blue) was superimposed on the ADP-AlF_4_-bound structure (colored by domain as in Figure 2) using the Hel1 domain as the reference. Nucleotide-binding motifs Va and VI are labeled. Only the ADP-AlF_4_ nucleotide is shown for clarity. (B) Close-up view of the Hel2-loop and its interactions with the dsRNA. The Twist74 (blue) and Twist87 (pink) AMPPNP-bound structures are superimposed onto the ADP-AlF_4_-bound structure (green) using Hel1 as the reference. (C) Overview of Twist74 (blue) superimposed on the ADP-AlF_4_-bound structure (green) using Hel1 as the reference. (D) Model of the ATPase cycle and proofreading mechanism. Only two filament protomers are shown for clarity. The low-twist (71°–81°) structures correspond to the ATP-bound catalytic ground state, the intermediate-twist (81°–91°) ADP-AlF_4_-bound structure is the transition state, and the intermediate- and high-twist (91°–96°) states represent nucleotide-free states. The four panels relate to the panels in Figures 3C–3F. See also Videos S1, S2, S3, S4, and S5. Figure360: An Author Presentation of Figure 7

There is less variation across the cryo-EM structures in the number of RNA contacts formed by the helicase motifs. Eight out of the ten RNA-binding helicase motifs are engaged with the RNA (motifs Ia, Ib, Ic, IIa, IV, IVa, V, and Vc, as defined by Jiang et al. [2011], with IVb and Vb not engaged). Hel2 forms a more extensive set of contacts than Hel1, with contact areas of 565–660 Å^2^ for Hel2 versus 492–502 Å^2^ for Hel1. Many of the Hel2-RNA contacts are formed by the Hel2 loop (residues 758–767, adjacent to motif IVa), identified previously as a key element for dsRNA stem recognition by inserting into the major groove (Wu et al., 2013). This loop has a similar conformation in the cryo-EM and crystal structures (Uchikawa et al., 2016, Wu et al., 2013), and insertion of the loop into the RNA major groove causes a similar widening of the groove from 12 Å to 16 Å. The groove is widened further (to 18 Å) in the ADP-AlF_4_-bound structure. Moreover, in the Twist74 and ADP-AlF_4_-bound structures, His759, at the apex of the Hel2 loop, is positioned so that it could form a hydrogen bond with the O4 or N4_-_ atom of a pyrimidine base. The analogous residue in chicken MDA5 (His733) forms a hydrogen bond with an RNA base, albeit with a purine (G), in a crystal structure in complex with ADP:Mg^2+^ (Uchikawa et al., 2016). In contrast, in the Twist87 and Twist91 structures the Hel2 loop does not form any base contacts and is less firmly engaged with the RNA major groove. Previous work has shown that the Hel2 loop is required for dsRNA-dependent ATP hydrolysis by MDA5 (Wu et al., 2013). Hence, our data support the hypothesis that ATP binding and progression of catalysis to the transition state promote progressive insertion of the Hel2 loop into the RNA major groove, causing a widening of the groove (Figure 7B; Video S5). Consistent with this, the dsRNA is slightly stretched along its helical axis in the cryo-EM structures relative to free dsRNA (Pabit et al., 2016), and the increase in the rise per bp over the asymmetric unit versus free dsRNA is 13% in the ADP-AlF_4_-bound structure versus only 8% in the Twist87 structure, which has very low nucleotide occupancy in the active site.

Video S5. Video Highlighting the Domain Motions that Accompany the Transition from the ATP-Bound Ground State to the Nucleotide-free State via the ADP-AlF_4_-Bound Transition-State Intermediate, Related to Figures 2, 4, and 7 Close-ups of the Hel1 and Hel2 domains (green and cyan, respectively), with morphing between the refined atomic models of the Twist74 (ATP-bound), ADP-AlF_4_-bound, and Twist87 (nucleotide-free) structures. Two orthogonal views are shown. The first view highlights the transition to and from the closed Hel1-Hel2 conformation, in the ADP-AlF_4_-bound structure. The second view highlights the motion of the Hel2-loop, and the distortions of the dsRNA duplex that accompany the transition to and from the closed Hel1-Hel2 conformation, in the ADP-AlF_4_-bound structure. MDA5 protomers and bound dsRNA are colored as in the other videos.

### ATP Hydrolysis Causes Hel1-Hel2 Rotation, Increasing the RNA Footprint and Filament Twist

The structure of MDA5-dsRNA filaments bound to the catalytic transition state analog ADP-AlF_4_ captures a key intermediate in the ATPase cycle. The Hel1 and Hel2 domains are in the fully closed state, with all six nucleotide-binding motifs correctly positioned for catalysis. The configuration of the helicase and pincer domains is similar to those in the closed structures of RIG-I and LGP2 bound to ADP-AlF_4_ (Kowalinski et al., 2011, Uchikawa et al., 2016). Superposition of the ATP- and ADP-AlF_4_-bound MDA5 structures via Hel1 shows that, as in RIG-I and LGP2, the transition from the semi-closed state to the closed state involves a 6°–7° rotation of Hel2 relative to Hel1, which brings nucleotide binding motifs Va and VI in Hel2 into position for catalysis (Figure 7A; Figure360; Video S5). This rotation of Hel2 relative to Hel1 is transduced by the pincer domain and is part of the conserved allosteric mechanism coupling ATP hydrolysis to dsRNA binding in RLRs (Rawling et al., 2014). As in LGP2 (Uchikawa et al., 2016), the transition from the semi-closed ATP-bound state to the closed ADP-AlF_4_-bound state is accompanied by a shift in the interactions with the RNA backbone made by Hel2 (via motifs IVa and V) by one phosphate along the backbone in the 3′ direction, whereas the interactions of Hel1 with the RNA (via motifs Ia, Ib, Ic, and IIa) are unchanged. The net effect of this shift in Hel2-RNA backbone interactions is a 1-bp increase in the overall footprint of MDA5, from 14 bp in the ATP-bound state to 15 bp in the transition state. Hence, progression from the catalytic ground state (semi-closed, ATP-bound) to the transition state (closed, ADP-AlF_4_-bound) induces a rotation of Hel2 relative to Hel1 that increases the RNA-binding footprint by 1 bp. We conclude that ATP hydrolysis by MDA5 is directly coupled to a 1-bp increase in RNA-binding footprint.

Along with the increase in RNA-binding footprint, the rotation of Hel2 relative to Hel1 upon ATP hydrolysis causes a shift in the position of the RNA backbone at the Hel2 contact site in the direction of the Hel2 rotation. As noted above, the Hel2 loop forms many of the Hel2-RNA contacts, inserts into the RNA major groove, and causes widening of the groove. The extent of the widening is greater in the transition state, which contributes to the shift in RNA backbone position relative to the ground state. The net shift of the RNA backbone in the transition state suggests that ATP hydrolysis causes a local distortion of the RNA at the Hel2 loop contact site (Figure 7B; Videos S1, S2, S3, S4, and S5). Consistent with this, in the mid- and high-twist semi-closed structures (Twist87 and Twist91), the Hel2 loop is less firmly engaged with the RNA major groove, the RNA backbone is in a similar position as in the ground state (Figure 7B), and the overall helical rise is reduced by 2 Å relative to the transition state (Table 1). Hence the RNA is less distorted in the low-nucleotide-occupancy Twist87 structure than in the transition state, which has the same twist. This suggests that the strain introduced in the RNA in the transition state dissipates upon dissociation of the nucleotide and relaxation of the Hel1-Hel2 interface back to the semi-closed state. Notably, the Hel2-RNA backbone interactions of the Twist87 and Twist91 structures are in the same register as in the ground state structure (Twist74) despite having the same 15-bp footprint as the transition state structure. This suggests that the increases in RNA-binding footprint and helical twist gained upon ATP hydrolysis are maintained after dissociation of the nucleotide.

In contrast to LGP2 and RIG-I, the conformational change from the ATP-bound ground state to the ADP-AlF_4_-bound transition state is coupled to shifts in both the Hel2i and CTD domains such that the transition state structure wraps more tightly around the dsRNA, reducing the gap between Hel1 and the CTD by ∼5 Å (Figure 7C). This slightly tighter winding of MDA5 around the RNA could generate overwinding of the RNA, which could explain the increase in helical twist that occurs in going from the ground state to the transition state.

## Discussion

The dsRNA-binding cooperativity and length specificity of MDA5, which are critical for its signaling activity, are encoded by the filament-forming interfaces. The cryo-EM structures of MDA5-dsRNA filaments determined in this study identify two filament-forming surfaces, providing an essential missing link to understanding the polymerization-dependent recognition of dsRNA by MDA5 at the molecular level. The predominantly hydrophobic nature of the filament-forming contacts provides the flexibility necessary to support cooperative filament assembly on inherently flexible dsRNA. We have shown that mutation of filament-forming residues results in loss of filament formation and MDA5-dependent signaling, with the exception of a pair of mutations, which moderately enhances signaling. In a clinical setting, increased interferon signaling from mutations that stabilize the filament-forming interfaces has potential to be pathogenic. Indeed, many gain-of-function mutations in MDA5 cause severe autoimmune disease (Ahmad et al., 2018, Rice et al., 2014, Rutsch et al., 2015), although the disease-associated mutations reported so far map to the RNA and ATP binding sites. Hence the identification of the filament-forming interface has predictive value for researchers and also for clinicians who are likely to encounter patients with SNPs in the filament-forming regions of MDA5.

### Structural Changes in MDA5-dsRNA Filaments Coupled with ATP Binding and Hydrolysis

A comparative analysis of cryo-EM structures determined in the presence of different ATP nucleotide analogs reveals clear correlations between the type and occupancy of the nucleotide bound in the catalytic site, the length of the RNA-binding footprint, and the helical twist of the filaments. Filaments formed with transition state analog ADP-AlF_4_ had a narrow distribution of intermediate twists (81°–91°) and a 15-bp footprint, whereas filaments formed with a high concentration of ATP (10 mM) had mostly low helical twist (71°–81°) and a 14-bp footprint (Figure 1C). In both of these cases, the nucleotide occupancy in the active site was high (Figure 3). Filaments formed with lower concentrations of the nonhydrolyzable ATP analog AMPPNP (1–2.5 mM) had a broad range of twists (71°–96°) and 15-bp footprints. Within these populations, the low-twist filaments had relatively high AMPPNP occupancy in the active site, but the filaments with intermediate and high twists had very little (if any) nucleotide in the active site. The lack of AMPPNP density in these twist classes is not surprising, as the AMPPNP concentration present in the samples was of the same order of magnitude as the K_M_ for ATP reported for chicken MDA5 bound to a 24-bp dsRNA (2.2 ± 0.47 mM; Uchikawa et al., 2016). Although the K_M_(ATP) is likely to be smaller for MDA5 filaments on long dsRNAs, if we assume a K_d_(AMPPNP) value of 1 mM, the occupancy of AMPPNP in the catalytic site can be expected to be 30%–50% given the concentration of MDA5 in the imaged samples (3.45 μM). This would imply that less than half of the imaged MDA5 molecules contained AMPPNP in their active site. Consistent with this, more than half the filaments segments formed with 1–2.5 mM AMPPNP had intermediate or high twist, lacked AMPPNP density in the active site, and had essentially identical structures as segments formed without nucleotide (Figure 1). We conclude that ATP binding and hydrolysis are coupled to increases in the helical twist and RNA-binding footprint of the filament.

Together, our structural data support the hypothesis that our cryo-EM reconstructions have captured three distinct intermediates in the ATPase cycle. The low-twist structures correspond to the ATP-bound catalytic ground state, the intermediate-twist ADP-AlF_4_-bound structure is the transition state, and intermediate- and high-twist states represent nucleotide-free states (Figure 7D). The coincidence of all three twist states on the same filament (Figure 1B) therefore suggests that multiple nucleotide-binding states can coexist on one filament. The RecA-like domains Hel1 and Hel2 are in the closed conformation in the transition state and in the semi-closed conformation in the other states. The RNA-binding footprint is 14 bp in the ground state and expands to 15 bp in the transition and nucleotide-free states. Notably, the increased twist and RNA footprint are maintained in the low-nucleotide-occupancy states, even though the helicase domains return to the semi-closed state. Despite the increase in RNA footprint, the protein-RNA contact area decreases as the catalytic cycle proceeds, from 2,300 Å^2^ in the ground state to 2,100 Å^2^ in the intermediate-twist states and 1,900 Å^2^ in the high-twist nucleotide-free state. This implies that dissociation of ADP and Pi following hydrolysis causes MDA5 to loosen its grip on the RNA. Indeed, Uchikawa et al. (2016) reached the same conclusion based on the crystal structures of MDA5 and LGP2 bound to dsRNA and different nucleotide analogs. Consistent with this, MDA5 forms longer, more continuous filaments in the presence of nonhydrolyzable ATP analogs than without nucleotide (Berke et al., 2012, Peisley et al., 2012, Wu et al., 2013). In contrast, binding of ATP or transition-state analogs to RIG-I reduces its affinity for RNA (Rawling et al., 2015).

### Potential Role of ATP Hydrolysis in MDA5 Function

The structural snapshots of the ATPase cycle we have obtained provide clues on the potential role of ATP binding and hydrolysis in MDA5 signaling, though open questions remain. The ATPase cycle of RIG-I and MDA5 has been proposed to perform a proofreading function in discrimination of self- versus non-self dsRNA by increasing the rate of dissociation of the protein from shorter endogenous RNAs (Lässig et al., 2015, Peisley et al., 2012, Rawling et al., 2015). In the case of RIG-I, ATP binding (Rawling et al., 2015) and hydrolysis (Lässig et al., 2015) have both been reported to promote dissociation from non-cognate RNA ligands. In the case of MDA5, ATP hydrolysis was found to enhance the binding specificity for long dsRNAs and promote formation of more continuous and stable filaments while promoting dissociation from shorter dsRNAs (Peisley et al., 2012). As noted above, our structures show that binding of MDA5 induces significant distortions in the dsRNA backbone. The extent and location of these distortions vary in the different intermediates of the ATPase cycle (Video S5). A possible interpretation is that ATP hydrolysis by MDA5 tests the physical properties of the RNA—specifically resistance to twisting and bending—such that MDA5 is more likely to remain associated with cognate ligands (exogenous long continuous RNA duplexes) and more likely to dissociate from non-cognate ligands (deaminated Alu repeats and short endogenous RNAs) due to the different way each type of ligand responds to the ATP-dependent conformational changes in MDA5, including changes in helical twist. This would provide a mechanical proofreading mechanism dependent on ATP hydrolysis. Consistent with this hypothesis, biochemical studies suggest that ATP binding contributes to proofreading by RIG-I by challenging the interaction with RNA and promoting dissociation (Rawling et al., 2015).

Our structural data indicate that ATP hydrolysis is coupled with a 1-bp expansion in the dsRNA-binding footprint through ratchet-like movements of Hel2 relative to Hel1 (Figure 7B). This could in principle result in translocation of dsRNA. However, translocation would require cooperative binding of ATP to adjacent protomers and sequential hydrolysis in one direction along the filament. It appears more likely that expansion of the RNA-binding footprint is a local phenomenon. Local expansion of the MDA5 footprint would provide a possible explanation for the reported repair of MDA5 filament discontinuities through ATP hydrolysis (Peisley et al., 2012). Expansion of the binding footprint of a long continuous filament could also explain how MDA5 can displace viral proteins from dsRNA in an ATP-dependent, CARD-independent manner (Yao et al., 2015). Further work is required to determine more specifically how ATP hydrolysis propagates structural changes through MDA5-dsRNA filaments and how these changes may contribute to the proofreading and antiviral effector functions of MDA5.

## STAR★Methods

### Key Resources Table

**Table:** 

REAGENT or RESOURCE	SOURCE	IDENTIFIER
**Antibodies**		

Anti-FLAG primary antibody	Sigma-Aldrich	RRID:AB_262044; F1804
Anti-actin primary antibody	Abcam	RRID:AB_297660; AC-40 (ab11003)
Anti-mouse IgG (H+L) DyLight 800 4X PEG Conjugate	Cell Signaling	RRID:AB_10693543; 5257

**Bacterial and Virus Strains**		

*Escherichia coli* BL21(DE3) cells	Merck	69450

**Biological Samples**		

Bacteriophage Φ6 genomic dsRNA	ThermoFisher	F630 (Discontinued)

**Chemicals, Peptides, and Recombinant Proteins**		

384-well plate, black, clear flat bottom	Corning	3540
AlCl_3_	Alfa Aesar	14552.14
ADP	Sigma-Aldrich	A2754
AMPPNP	Sigma-Aldrich	10102547001
ATP	Sigma-Aldrich	A6419-1G
DTT	Thermo Scientific	R0862
HEPES	Sigma-Aldrich	H3375
HiTrapQ 5-ml column	GE Healthcare	17115401
KCl	Fisher Chemical	P/4280/53
Mant-AppNHp (Mant-AMPPNP)	Jena Bioscience	NU-214L
MgCl_2_	Sigma-Aldrich	M8266
NaF	Sigma-Aldrich	67414-1ML-F
Ni-NTA agarose	QIAGEN	30210
Poly(I:C)	Tocris Bioscience	4287
Polyethylenimine	Polysciences	24765
Ribonucleotide Solution Mix	New England Biolabs	N0466
Superdex 200 Increase 10/300 GL column	GE Healthcare	28990944
T7 RNA Polymerase	New England Biolabs	M0251L

**Critical Commercial Assays**		

ATPase/GTPase Activity Assay Kit	Sigma-Aldrich	MAK113-1KT
Dual-Luciferase Reporter Assay System	Promega	E1910
PureLink RNA Mini Kit	ThermoFisher	12183018A

**Deposited Data**		

Full resolution original images used in figures and supplemental figures	Mendeley Data	10.17632/djfd3yhtn5.1
MDA5-dsRNA 1-mM AMPPNP low-twist atomic coordinates	Protein Data Bank	PDB: 6G19
MDA5-dsRNA 1-mM AMPPNP intermediate-twist atomic coordinates	Protein Data Bank	PDB: 6G1S
MDA5-dsRNA 1-mM AMPPNP high-twist atomic coordinates	Protein Data Bank	PDB: 6G1X
MDA5-dsRNA 2.5-mM AMPPNP low-twist atomic coordinates	Protein Data Bank	PDB: 6GJZ
MDA5-dsRNA 2-mM ADP-AlF_4_ low-twist atomic coordinates	Protein Data Bank	PDB: 6GKH
MDA5-dsRNA 10-mM ATP low-twist atomic coordinates	Protein Data Bank	PDB: 6GKM
MDA5-dsRNA no nucleotide intermediate-twist atomic coordinates	Protein Data Bank	PDB: 6H61
MDA5-dsRNA no nucleotide high-twist atomic coordinates	Protein Data Bank	PDB: 6H66
MDA5-dsRNA 1-mM AMPPNP low-twist EM map	EM Data bank	EMD-4338
MDA5-dsRNA intermediate-twist EM map	EM Data bank	EMD-4340
MDA5-dsRNA high-twist EM map	EM Data bank	EMD-4341
MDA5-dsRNA 2.5-mM AMPPNP low-twist EM map	EM Data bank	EMD-0012
MDA5-dsRNA 2-mM ADP-AlF_4_ low-twist EM map	EM Data bank	EMD-0023
MDA5-dsRNA 10-mM ATP low-twist EM map	EM Data bank	EMD-0024
MDA5-dsRNA no nucleotide intermediate-twist EM map	EM Data bank	EMD-0143
MDA5-dsRNA no nucleotide high-twist EM map	EM Data bank	EMD-0145
MDA5-dsRNA 1-mM AMPPNP cryoEM dataset	EMPIAR	10213
MDA5-dsRNA 2.5-mM AMPPNP cryoEM dataset	EMPIAR	10209
MDA5-dsRNA 2-mM ADP-AlF_4_ cryoEM dataset	EMPIAR	10211
MDA5-dsRNA 10-mM ATP cryoEM dataset	EMPIAR	10208
MDA5-dsRNA no nucleotide cryoEM dataset	EMPIAR	10210

**Experimental Models: Cell Lines**		

Human embryonic kidney (HEK) 293T cells		N/A

**Recombinant DNA**		

pET28a	Merck	69864
pCold-TF	TAKARA	N/A
pIFN-Luc	Promega	N/A
pRL-TK	Promega	N/A
pLEXm	Aricescu et al., 2006	N/A

**Software and Algorithms**		

ImageJ	NIH	https://imagej.nih.gov/ij/
EPU Automated Data Acquisition Software for Single Particle Analysis v1.9.1	ThermoFisher	https://www.fei.com/software/epu/
RELION v2.1.0	Scheres, 2012	https://www2.mrc-lmb.cam.ac.uk/relion/index.php?title=Main_Page
MOTIONCOR2	Zheng et al., 2017	http://msg.ucsf.edu/em/software/motioncor2.html
Gctf v1.06	Zhang, 2016	N/A
Coot v0.8.9	Emsley and Cowtan, 2004	https://www2.mrc-lmb.cam.ac.uk/personal/pemsley/coot/
PHENIX v1.13	Adams et al., 2010	http://www.phenix-online.org
UCSF Chimera	UCSF Resource for Biocomputing, Visualization and Informatics	https://www.cgl.ucsf.edu/chimera/

**Other**		

*In vitro*-transcribed 1-kb dsRNA	This paper	N/A
QUANTIFOIL R1.2/1.3 grids	Quantifoil Micro Tools	R1.2/1.3

### Contact for Reagent and Resource Sharing

Further information and requests for resources and reagents should be directed to and will be fulfilled by the Lead Contact, Yorgo Modis (ymodis@mrc-lmb.cam.ac.uk).

### Experimental Model Details

#### Cell lines

HEK293T cells were a kind gift from Yiquan Tang in William Schafer’s group (MRC Laboratory of Molecular Biology).

#### Microbe strains

All proteins were expressed in *Escherichia coli* BL21(DE3) cells (Merck).

### Method Details

#### Protein expression and purification of MDA5

Genes encoding mouse *MDA5 (IFIH1),* UniProt: Q8R5F7, were cloned in frame with the N-terminal histidine purification tag of a pET28a vector in which the thrombin cleavage site was replaced with a tobacco etch virus (TEV) protease cleavage site. Wild-type (WT) mouse *MDA5* with residues 646–663 deleted, MDA5-ΔL2, was expressed from a previously generated pET28a expression plasmid (Berke and Modis, 2012). The ΔL2 loop deletion is in a solvent exposed loop of Hel2i. The deleted sequence is not conserved in other vertebrate *MDA5* genes (Figure S5). Its deletion does not affect the dsRNA binding, ATPase or interferon signaling activities of MDA5 (see (Berke et al., 2012, Wu et al., 2013), in supplementary information). Mouse MDA5-ΔL2 mutants were cloned into the pCold vector with an N-terminal extension comprising a hexahistidine tag, maltose binding protein (MBP) and human rhinovirus (HRV) 3C protease cleavage site or pET28a vector in which the thrombin cleavage site was replaced with a TEV protease cleavage site.

*Escherichia coli* BL21(DE3) cells were transformed with an *MDA5* construct and grown to OD_600_ 0.4-0.5 at 37°C. After cooling for 15 min at 16°C, expression was induced overnight with 0.4 mM isopropyl-β-D-1-thiogalactopyranoside (IPTG). Harvested cells were resuspended in SPG buffer pH 6.0 (49 mM NaH_2_PO_4_, 49 mM glycine, 14 mM succinic acid), 0.4 M KCl, 5 mM β-mercaptoethanol, 5% glycerol, 20 mM imidazole. Roche Complete Protease Inhibitor (without EDTA), 0.2 mM phenylmethanesulfonylfluoride (PMSF) and 1 μg mL^−1^ pepstatin A were added immediately prior to cell lysis.

MDA5-ΔL2 protein was purified with three liquid chromatography steps: nickel-affinity with Ni-NTA agarose (QIAGEN), anion-exchange with a HiTrapQ or ResourceQ column (GE Healthcare), and size-exclusion with a Superdex 200 Increase 10/300 GL column (GE Healthcare). The protein used for nucleotide-free MDA5-dsRNA data collection was treated with 0.5 mM EDTA for 1 h to remove nucleotide before loading on a Superdex 200 Increase 10/300 GL column. The MBP-tagged mutants were purified by with the same procedure except that on-column cleavage with HRV 3C protease in a HEPES pH 7.0 buffer was used to elute the proteins from the Ni-NTA agarose beads.

#### Extraction and *in vitro* transcription of double-stranded RNA

Bacteriophage Φ6 genomic dsRNA was purchased from ThermoFisher. The 1 kb dsRNA was prepared by T7 *in vitro* transcription using T7 RNA Polymerase (New England Biolabs) following to the manufacturer’s instructions. Two complementary strands were co-transcribed. The transcripts were treated with DNase and purified by column-based purification (ThermoFisher PureLink RNA Mini Kit). After purification transcripts were heated at 95°C for 5 min then annealing by cooling to room temperature.

#### Negative stain EM

For the filament assembly assay, 26 μg mL^−1^ MDA5-ΔL2 or MDA5-ΔL2 mutants were incubated with 3.24 μg mL^−1^ of 1 kb dsRNA in buffer containing 20 mM HEPES pH 7.7, 0.1 M KCl, 5 mM MgCl_2_, 1 mM AMPPNP on ice for 30 min. Samples were applied to glow-discharged carbon-coated grids, negatively stained with uranyl acetate [2% (wt/vol)], and imaged with an FEI Tecnai 12 transmission electron microscope at an accelerating voltage of 120 kV. Images were taken at 3-4 μm defocus, 26000 × magnification and with 4 Å per pixel. The length of filaments was measured manually with ImageJ.

#### CryoEM sample preparation and data collection

WT MDA5-ΔL2 at a concentration of 0.8 g L^−1^ was incubated with 0.03 g L^−1^ bacteriophage *Φ6* dsRNA (ThermoFisher) in buffer containing 20 mM HEPES pH 7.7, 0.1 M KCl, 5 mM MgCl_2_, 2 mM DTT and 1 mM AMPPNP on ice for 30 min. CryoEM grids were prepared with a Vitrobot (ThermoFisher) at 4°C at 100% humidity. Filament samples were diluted twofold with buffer (20 mM HEPES pH 7.7, 0.1 M KCl, 5 mM MgCl_2_, 2 mM DTT) and a 3.5 μl aliquot of the sample was immediately applied onto a glow-discharged 300-mesh gold Quantifoil R1.2/1.3 grid (Quantifoil Micro Tools). For the 2.5 mM AMPPNP filament sample, 1 g L^−1^ protein was incubated with 0.05 g L^−1^ 1-kb dsRNA and 2.5 mM AMPPNP on ice for 5–10 min. For the 10 mM ATP filament sample, 1 g L^−1^ protein was incubated with 0.05 g L^−1^ 1-kb dsRNA and 10 mM ATP for 7.5 min. For the 2 mM ADP-AlF_4_ filament sample, 1 g L^−1^ protein was incubated with 0.05 g L^−1^ 1-kb dsRNA, 2 mM ADP, 4 mM AlCl_3_ and 40 mM NaF for 2 h. For the nucleotide-free MDA5-dsRNA filament sample, 1 g L^−1^ protein (EDTA-treated during purification) was incubated with 0.05 g L^−1^ 1-kb dsRNA. The grids were blotted for 4 s and plunge-frozen in liquid ethane cooled by liquid nitrogen in the Vitrobot. CryoEM data collection was performed on a Titan Krios microscope operated at 300 kV equipped with Falcon III direct electron detector in counting mode (ThermoFisher). For the 1-mM AMPPNP dataset, a total of 1,563 micrographs from 3 independent sessions were recorded at a calibrated magnification of 75000 × leading to a magnified pixel size of 1.07 Å. Each movie comprises 75 sub frames with a total dose of 29.85 e^−^ Å^−2^, exposure time 60 s and a dose rate 0.57 e^−^ pixel^−1^ second^−2^ on the detector. Data acquisition was performed with EPU Automated Data Acquisition Software for Single Particle Analysis (ThermoFisher) with one shot per hole at −1.8 μm to −2.7 μm defocus. For the 2.5-mM AMPPNP, 2-mM ADP-AlF_4_ 10-mM ATP, and nucleotide-free samples, the datasets were collected as described for the 1-mM AMPPNP sample except with minor variation of dose and pixel size due to the use of different microscopes.

#### Image processing and helical reconstruction

All movies were motion-corrected and dose-weighted with MOTIONCOR2 (Zheng et al., 2017). Aligned, non-dose-weighted micrographs were then used to estimate the contrast transfer function (CTF) with GCTF (Zhang, 2016). All subsequent image processing steps were performed using helical reconstruction in RELION 2.1.0 (He and Scheres, 2017, Scheres, 2012). Approximately 4,100 segments were manually picked in RELION. One round of reference-free two-dimensional (2D) classification was performed to produce templates for reference-dependent auto-picking. A limited resolution E-step (low-pass filter) of 15 Å was applied to prevent overfitting. Using the resulting 2D classes as templates, overlapping helical filament segments were automatically picked with an inter-box distance of 44 Å, to coincide with the helical rise measured from the power spectra.

With the first 891-movie dataset collected on the Krios microscope, reconstructions with a box size of 400, 320, 256, 224 or 192 pixels were compared. A cylinder with a 12 nm diameter was used as the initial model. A spherical mask with a diameter equal to 90% of the box size was applied. Selected 2D classes were used with the cylindrical initial model to perform 3D auto-refinement in RELION using the helical rise measured from the power spectra (44 Å) and the twist derived from the negative-stain EM structure (Berke et al., 2012) (74° - 93°). RELION reduces blurring effects from variability of helical symmetry between filament segments by only using the central part of an intermediate asymmetrical reconstruction for real-space helical symmetrization (He and Scheres, 2017). The corresponding helical_z_percentage parameter in RELION was set to 45%. The output volume was used as the reference model for 3D classification. 3D classification with different box sizes, from 192 to 400 pixels, resulted in 3D classes with the same range of twists, from 72° to 96° for the 1-mM AMPPNP dataset. The 224-pixel box produced the 2D class averages with the most clearly discernible secondary structure features and the 3D reconstruction of the highest resolution. A box size of 224 pixels were therefore selected for the full reconstruction.

The segments were extracted from dose-weighted micrographs in 224-pixel boxes with a 44 Å inter-box distance. For the 1 mM AMPPNP dataset, a total of 367,549 segments were extracted from the full dataset. Five rounds of reference-free 2D classification were performed to remove low-quality filaments, yielding 255,437 particles.

The particles were subjected to 3D auto-refinement. Beam-induced motion of individual filament segments was corrected with movie refinement. *B*-factor weights were applied to each frame in particle polishing to compensate for radiation damage (Scheres, 2014). One round of 2D classification was performed to discard low-quality classes, yielding 245,826 particles. The particles were divided into six classes in 3D classification with a local search of helical symmetry (twist 73°- 94° and rise 42.5 - 45.5 Å). A class with a 74.9° twist and 33,138 particles was selected and 3D auto-refined to 4.7 Å resolution. Further refinement with a soft mask around the filament produced a volume with 3.87 Å resolution and final twist 74.3°. Helical symmetry was imposed on the unfiltered half-maps with relion_helix_toolbox (He and Scheres, 2017), which improved the resolution to 3.68 Å after post-processing with a soft mask applied (Table 1). The 3D reconstruction was then sharpened with a *B*-factor of −175 Å^−2^. A second 3D class with twist 90.8° and 39,987 particles processed following the same procedure, yielding a map with an overall resolution of 3.93 Å with a twist of 90.9°. The overall map resolutions reported in Table 1 were derived from Fourier shell correlation (FSC) calculations between reconstructions from two independently refined half-maps (FSCmap2map), and reported resolutions are based on the gold-standard (FSC = 0.143 Å) criterion (Figures S2A–S2C). Local resolution was estimated with RELION.

In a parallel alternative processing workflow, all 255,437 particles from initial 2D classification were subjected to two more rounds of 2D classification, yielding 215,722 particles. The particles were divided into four classes in 3D classification, with local search of helical symmetry (twist 74° - 94° and rise 44 - 46 Å). A class with 66,565 particles (twist 86.5° and rise 44.4 Å) was selected and 3D auto-refined to 5.7 Å resolution. After particle polishing, additional 3D auto-refinement with a soft mask produced a density map at 4.13 Å resolution. Further 3D classifications did not improve the density quality. Helical symmetry was imposed on the unfiltered half-maps, and a *B-*factor of −165 Å^−2^ was applied to the reconstruction, which had a resolution of 3.93 Å. We note that although the overall resolutions for the Twist87 and Twist91 structures were the same, the Twist87 density had a greater local resolution range than Twist91, with slightly higher resolution near the helical axis, and lower resolution away from the axis.

The 2.5-mM AMPPNP, 10-mM ATP and 2-mM ADP-AlF_4_ datasets were processed using the same procedure as for the 1-mM AMPPNP dataset, except that the 2-mM ADP-AlF_4_ dataset was generated from merging data collected in separate sessions with different pixel sizes, of 1.085 Å and 1.07 Å, respectively. The dataset with 1.085 Å pixel size was rescaled to 1.07 Å pixel size and then merged with the 1.07 Å-pixel-size dataset.

#### Model building and refinement

The crystal structure of human MDA5 bound to a 12-bp dsRNA oligonucleotide, PDB: 4GL2 (Wu et al., 2013) was used as the starting atomic model for all three twist classes. The model was docked as a rigid body into the density for the central subunit in each reconstruction with UCSF Chimera (Pettersen et al., 2004). Initial docking was performed manually and was followed by real space fitting with the Fit in Map function. The positions of individual protein secondary structure elements and domain fragments were then sequentially refined using the Jiggle Fit script (Brown et al., 2015) in COOT (Emsley and Cowtan, 2004). Each model was then manually rebuilt in COOT to optimize the fit to the density, using the crystal structure of chicken MDA5 bound to a 10-bp dsRNA and ADP-Mg^2+^, PDB: 5JCF (Uchikawa et al., 2016) as a guide for rebuilding in regions where the crystal structure of human MDA5 was disordered or had a more divergent conformation. The sequence of the dsRNA was randomly selected from the bacteriophage Φ6 genome and is purely representative since any RNA sequence-specific information was lost during helical symmetry averaging. The two adjacent subunits in the filament were then generated by applying the helical symmetry for each reconstruction from RELION to the respective rebuilt atomic model. The resulting models, containing three MDA5 subunits each, allowed the filament forming interfaces to be refined in subsequent real space refinement. The bases in the dsRNAs bound to each subunit were renumbered so they would be treated as a single 42/45-bp dsRNA in subsequent steps. Real space refinement was performed on the three-subunit models, in PHENIX 1.13 (Adams et al., 2010), using the final helically averaged volumes from RELION as the maps for refinement. The global minimization and atomic displacement parameter (ADP) refinement options were selected in PHENIX. The following restraints were used in real space refinement: secondary structure restraints, non-crystallographic symmetry (NCS) restraints between the protein subunits, side chain rotamer restraints, and Ramachandran restraints. Key refinement statistics are listed in Table 1.

#### Model validation and analysis

The FSC curve between the final model and full map after post-processing in RELION, FSC(model2map), is shown in Figures S2A–S2C. Cross-validation against overfitting was performed as described by Amunts et al. (Amunts et al., 2014). The atoms in each final atomic model were displaced by 0.25 Å in random directions. The shifted coordinates were then refined against one of the half-maps generated in RELION, the “work set.” This test refinement was performed with PHENIX using the same procedure as for the refinement of final models (see above). The other half-map, the “test set” was not used in refinement for cross-validation. FSC curves of the refined shifted model against the work set, FSCwork, and against the test set, FSCfree, are shown in Figures 2D–2F. The FSCwork and FSCfree curves are not significantly different, consistent with the absence of overfitting in our final models.

The quality of the atomic models, including basic protein and RNA geometry, Ramachandran plots, and clash analysis, was assessed and validated with MolProbity (Chen et al., 2010) as implemented in PHENIX, and with the Worldwide PDB (wwPDB) OneDep System (https://deposit-pdbe.wwpdb.org/deposition).

To determine which conformational state the helicase modules of the cryoEM structures were in, each model was superimposed onto the fully closed structure of LGP2 (PDB: 5JAJ (Uchikawa et al., 2016)) using conserved core secondary structure elements of Hel1 as the reference. The rotation angle relating the Hel2 domains of the aligned structures was found to be in 8.8°- 9.5° for the filaments formed in the presence of ATP or AMPPNP and 3° for the ADP-AlF_4_-bound structure. The Hel1-Hel2 conformations are defined by (Uchikawa et al., 2016) as follows: 0° - 3° for the closed state, 7° - 13° for the semi-closed state, ∼40° for the semi-open state, and 50° - 60° for the open state.

#### Luciferase reporter cell signaling assay

HEK293T cells in 12-well plates were transfected with 400 ng mL^−1^ of firefly luciferase under control of the IFN-β promoter (pIFN-Luc, Promega), 40 ng mL^−1^ of *Renilla* luciferase under a constitutive promoter (pRL-TK, Promega), and 40 ng mL^−1^ of pLEXm vector (Aricescu et al., 2006) containing either no insert, WT human MDA5ΔL2 (pLEXm MDA5-Δ644-663), or human MDA5-ΔL2 with mutations generated by overlap PCR from WT MDA5-ΔL2. All transfections were performed with polyethylenimine (Sigma-Aldrich). After expression for 6 h, cells were transfected with poly(I:C) (Tocris Bioscience). After 24 h, cell lysates were prepared, and luciferase activity was measured using Promega assay kits according to the manufacturer’s instructions. Firefly luciferase activity was normalized against the co-transfected *Renilla* luciferase.

#### ATPase assay

Mouse MDA5 protein (WT or signaling-defective mutant) was diluted to a concentration of 75 nM in a solution containing 2.25 nM 1-kb dsRNA, 1 mM ATP, 20mM HEPES pH 7.8, 0.15 M KCl, 1.5 mM MgCl_2_ and 1 mM DTT and incubated at 37°C. Samples of the reaction were extracted and quenched with 20 mM EDTA at 20 s intervals. The concentration of inorganic phosphate released by hydrolysis of ATP was measured by tracking absorbance at 620 nm of malachite green binding to phosphate ions using the ATPase/GTPase activity colorimetric assay (Sigma-Aldrich).

#### Fluorescence polarization AMPPNP binding assay

The buffer used for this assay was 20 mM HEPES pH 7.8, 0.15 M KCl, 1.5 mM MgCl_2_ and 1 mM DTT. Concentrations of 0.2 μM, 2 μM 10 μM and 20 μM Mant-AMPPNP (Jena Bioscience) were tested for fluorescent signal. 10 μM Mant-AMPPNP was selected for the assay as it was the minimum concentration required for detection of fluorescent signal relative to a blank containing 10 μM (1.14 g l^−1^) MDA5 and 0.303 g l^−1^ poly(I:C) RNA but no Mant-AMPPNP. Mouse MDA5 protein (WT or ATPase-defective mutant) was titrated from 0 to 40 μM into a solution containing 10 μM Mant-AMPPNP and poly(I:C) RNA at a 1:3 molar ratio of MDA5 to RNA binding sites, assuming 15 bp per binding site. The mixture was incubated for 1 h at room temperature in a black, flat-bottomed 384-well plate (Corning). Fluorescence polarization was measured with a Clariostar plate reader (BMG LABTECH) with an excitation wavelength of 355 nm and an emission wavelength of 448 nm.

### Quantification and Statistical Analysis

No statistical methods were used to predetermine sample size, experiments were not randomized, and the investigators were not blinded to experimental outcomes. Luciferase-reporter cell signaling data are represented as the mean ± standard error of the mean of three replicates conducted in a single independent experiment. Data are representative of at least three independent experiments.

### Data Availability

The accession numbers for the atomic coordinates reported in this paper are PDB: 6G19, 6G1S, 6G1X, 6GJZ, 6GKH, 6GKM, 6H61, 6H66. The accession numbers for the cryo-EM densities reported in this paper are EMDB: EMD-4338, EMD-4340, EMD-4341, EMD-0012, EMD-0023, EMD-0024, EMD-0143 and EMD-0145. The accession numbers for the raw electron micrographs reported in this paper are EMPIAR: 10208, 10209, 10210, 10211 and 10213. Full resolution original experimental images used in the figures and supplemental figures have been uploaded to Mendeley Data, https://doi.org/10.17632/djfd3yhtn5.1. Other data are available from the Lead Contact upon reasonable request.

## Supplemental Information

Document S1. Figures S1–S7

Document S2. Full wwPDB/EMDataBank EM Map/Model Validation Report

Document S3. Article plus Supplemental Information

## References

[bib1] Adams P.D., Afonine P.V., Bunkóczi G., Chen V.B., Davis I.W., Echols N., Headd J.J., Hung L.W., Kapral G.J., Grosse-Kunstleve R.W. (2010). PHENIX: a comprehensive Python-based system for macromolecular structure solution. Acta Crystallogr. D Biol. Crystallogr..

[bib2] Ahmad S., Mu X., Yang F., Greenwald E., Park J.W., Jacob E., Zhang C.Z., Hur S. (2018). Breaching self-tolerance to Alu duplex RNA underlies MDA5-mediated inflammation. Cell.

[bib3] Amunts A., Brown A., Bai X.C., Llácer J.L., Hussain T., Emsley P., Long F., Murshudov G., Scheres S.H.W., Ramakrishnan V. (2014). Structure of the yeast mitochondrial large ribosomal subunit. Science.

[bib4] Aricescu A.R., Lu W., Jones E.Y. (2006). A time- and cost-efficient system for high-level protein production in mammalian cells. Acta Crystallogr. D Biol. Crystallogr..

[bib5] Berke I.C., Modis Y. (2012). MDA5 cooperatively forms dimers and ATP-sensitive filaments upon binding double-stranded RNA. EMBO J..

[bib6] Berke I.C., Yu X., Modis Y., Egelman E.H. (2012). MDA5 assembles into a polar helical filament on dsRNA. Proc. Natl. Acad. Sci. USA.

[bib7] Brown A., Long F., Nicholls R.A., Toots J., Emsley P., Murshudov G. (2015). Tools for macromolecular model building and refinement into electron cryo-microscopy reconstructions. Acta Crystallogr. D Biol. Crystallogr..

[bib8] Chen V.B., Arendall W.B., Headd J.J., Keedy D.A., Immormino R.M., Kapral G.J., Murray L.W., Richardson J.S., Richardson D.C. (2010). MolProbity: all-atom structure validation for macromolecular crystallography. Acta Crystallogr. D Biol. Crystallogr..

[bib9] Chung H., Calis J.J.A., Wu X., Sun T., Yu Y., Sarbanes S.L., Dao Thi V.L., Shilvock A.R., Hoffmann H.H., Rosenberg B.R. (2018). Human ADAR1 prevents endogenous RNA from triggering translational shutdown. Cell.

[bib10] Devarkar S.C., Wang C., Miller M.T., Ramanathan A., Jiang F., Khan A.G., Patel S.S., Marcotrigiano J. (2016). Structural basis for m7G recognition and 2′-O-methyl discrimination in capped RNAs by the innate immune receptor RIG-I. Proc. Natl. Acad. Sci. USA.

[bib11] Emsley P., Cowtan K. (2004). Coot: model-building tools for molecular graphics. Acta Crystallogr. D Biol. Crystallogr..

[bib12] Funabiki M., Kato H., Miyachi Y., Toki H., Motegi H., Inoue M., Minowa O., Yoshida A., Deguchi K., Sato H. (2014). Autoimmune disorders associated with gain of function of the intracellular sensor MDA5. Immunity.

[bib13] Goubau D., Schlee M., Deddouche S., Pruijssers A.J., Zillinger T., Goldeck M., Schuberth C., Van der Veen A.G., Fujimura T., Rehwinkel J. (2014). Antiviral immunity via RIG-I-mediated recognition of RNA bearing 5′-diphosphates. Nature.

[bib14] He S., Scheres S.H.W. (2017). Helical reconstruction in RELION. J. Struct. Biol..

[bib15] Herrero-Galán E., Fuentes-Perez M.E., Carrasco C., Valpuesta J.M., Carrascosa J.L., Moreno-Herrero F., Arias-Gonzalez J.R. (2013). Mechanical identities of RNA and DNA double helices unveiled at the single-molecule level. J. Am. Chem. Soc..

[bib16] Hou F., Sun L., Zheng H., Skaug B., Jiang Q.X., Chen Z.J. (2011). MAVS forms functional prion-like aggregates to activate and propagate antiviral innate immune response. Cell.

[bib17] Jiang F., Ramanathan A., Miller M.T., Tang G.Q., Gale M., Patel S.S., Marcotrigiano J. (2011). Structural basis of RNA recognition and activation by innate immune receptor RIG-I. Nature.

[bib18] Kato H., Takeuchi O., Sato S., Yoneyama M., Yamamoto M., Matsui K., Uematsu S., Jung A., Kawai T., Ishii K.J. (2006). Differential roles of MDA5 and RIG-I helicases in the recognition of RNA viruses. Nature.

[bib19] Kato H., Takeuchi O., Mikamo-Satoh E., Hirai R., Kawai T., Matsushita K., Hiiragi A., Dermody T.S., Fujita T., Akira S. (2008). Length-dependent recognition of double-stranded ribonucleic acids by retinoic acid-inducible gene-I and melanoma differentiation-associated gene 5. J. Exp. Med..

[bib20] Kowalinski E., Lunardi T., McCarthy A.A., Louber J., Brunel J., Grigorov B., Gerlier D., Cusack S. (2011). Structural basis for the activation of innate immune pattern-recognition receptor RIG-I by viral RNA. Cell.

[bib21] Lässig C., Matheisl S., Sparrer K.M., de Oliveira Mann C.C., Moldt M., Patel J.R., Goldeck M., Hartmann G., García-Sastre A., Hornung V. (2015). ATP hydrolysis by the viral RNA sensor RIG-I prevents unintentional recognition of self-RNA. eLife.

[bib22] Li W., Kamtekar S., Xiong Y., Sarkis G.J., Grindley N.D., Steitz T.A. (2005). Structure of a synaptic gammadelta resolvase tetramer covalently linked to two cleaved DNAs. Science.

[bib23] Li X., Ranjith-Kumar C.T., Brooks M.T., Dharmaiah S., Herr A.B., Kao C., Li P. (2009). The RIG-I-like receptor LGP2 recognizes the termini of double-stranded RNA. J. Biol. Chem..

[bib24] Liddington R.C., Yan Y., Moulai J., Sahli R., Benjamin T.L., Harrison S.C. (1991). Structure of simian virus 40 at 3.8-A resolution. Nature.

[bib25] Luo D., Ding S.C., Vela A., Kohlway A., Lindenbach B.D., Pyle A.M. (2011). Structural insights into RNA recognition by RIG-I. Cell.

[bib26] Luo D., Kohlway A., Vela A., Pyle A.M. (2012). Visualizing the determinants of viral RNA recognition by innate immune sensor RIG-I. Structure.

[bib27] Pabit S.A., Katz A.M., Tolokh I.S., Drozdetski A., Baker N., Onufriev A.V., Pollack L. (2016). Understanding nucleic acid structural changes by comparing wide-angle x-ray scattering (WAXS) experiments to molecular dynamics simulations. J. Chem. Phys..

[bib28] Peisley A., Lin C., Wu B., Orme-Johnson M., Liu M., Walz T., Hur S. (2011). Cooperative assembly and dynamic disassembly of MDA5 filaments for viral dsRNA recognition. Proc. Natl. Acad. Sci. USA.

[bib29] Peisley A., Jo M.H., Lin C., Wu B., Orme-Johnson M., Walz T., Hohng S., Hur S. (2012). Kinetic mechanism for viral dsRNA length discrimination by MDA5 filaments. Proc. Natl. Acad. Sci. USA.

[bib30] Pettersen E.F., Goddard T.D., Huang C.C., Couch G.S., Greenblatt D.M., Meng E.C., Ferrin T.E. (2004). UCSF Chimera--a visualization system for exploratory research and analysis. J. Comput. Chem..

[bib31] Pippig D.A., Hellmuth J.C., Cui S., Kirchhofer A., Lammens K., Lammens A., Schmidt A., Rothenfusser S., Hopfner K.P. (2009). The regulatory domain of the RIG-I family ATPase LGP2 senses double-stranded RNA. Nucleic Acids Res..

[bib32] Rawling D.C., Kohlway A.S., Luo D., Ding S.C., Pyle A.M. (2014). The RIG-I ATPase core has evolved a functional requirement for allosteric stabilization by the Pincer domain. Nucleic Acids Res..

[bib33] Rawling D.C., Fitzgerald M.E., Pyle A.M. (2015). Establishing the role of ATP for the function of the RIG-I innate immune sensor. eLife.

[bib34] Rice G.I., Del Toro Duany Y., Jenkinson E.M., Forte G.M., Anderson B.H., Ariaudo G., Bader-Meunier B., Baildam E.M., Battini R., Beresford M.W. (2014). Gain-of-function mutations in IFIH1 cause a spectrum of human disease phenotypes associated with upregulated type I interferon signaling. Nat. Genet..

[bib35] Rutsch F., MacDougall M., Lu C., Buers I., Mamaeva O., Nitschke Y., Rice G.I., Erlandsen H., Kehl H.G., Thiele H. (2015). A specific IFIH1 gain-of-function mutation causes Singleton-Merten syndrome. Am. J. Hum. Genet..

[bib36] Scheres S.H. (2012). RELION: implementation of a Bayesian approach to cryo-EM structure determination. J. Struct. Biol..

[bib37] Scheres S.H. (2014). Beam-induced motion correction for sub-megadalton cryo-EM particles. eLife.

[bib38] Takahasi K., Kumeta H., Tsuduki N., Narita R., Shigemoto T., Hirai R., Yoneyama M., Horiuchi M., Ogura K., Fujita T., Inagaki F. (2009). Solution structures of cytosolic RNA sensor MDA5 and LGP2 C-terminal domains: identification of the RNA recognition loop in RIG-I-like receptors. J. Biol. Chem..

[bib39] Uchikawa E., Lethier M., Malet H., Brunel J., Gerlier D., Cusack S. (2016). Structural Analysis of dsRNA Binding to Anti-viral Pattern Recognition Receptors LGP2 and MDA5. Mol. Cell.

[bib40] Wang Y., Ludwig J., Schuberth C., Goldeck M., Schlee M., Li H., Juranek S., Sheng G., Micura R., Tuschl T. (2010). Structural and functional insights into 5′-ppp RNA pattern recognition by the innate immune receptor RIG-I. Nat. Struct. Mol. Biol..

[bib41] Wu B., Peisley A., Richards C., Yao H., Zeng X., Lin C., Chu F., Walz T., Hur S. (2013). Structural basis for dsRNA recognition, filament formation, and antiviral signal activation by MDA5. Cell.

[bib42] Wu B., Peisley A., Tetrault D., Li Z., Egelman E.H., Magor K.E., Walz T., Penczek P.A., Hur S. (2014). Molecular imprinting as a signal-activation mechanism of the viral RNA sensor RIG-I. Mol. Cell.

[bib43] Yao H., Dittmann M., Peisley A., Hoffmann H.H., Gilmore R.H., Schmidt T., Schmidt-Burgk J., Hornung V., Rice C.M., Hur S. (2015). ATP-dependent effector-like functions of RIG-I-like receptors. Mol. Cell.

[bib44] Zhang K. (2016). Gctf: Real-time CTF determination and correction. J. Struct. Biol..

[bib45] Zheng S.Q., Palovcak E., Armache J.P., Verba K.A., Cheng Y., Agard D.A. (2017). MotionCor2: anisotropic correction of beam-induced motion for improved cryo-electron microscopy. Nat. Methods.

