# Supplementary material for: Cryo-EM Structures of MDA5-dsRNA Filaments at Different Stages of ATP Hydrolysis
Source: Mol Cell. 2018 Dec 20;72(6):999–1012.e6. doi: 10.1016/j.molcel.2018.10.012 (PMC6310684; doi:10.1016/j.molcel.2018.10.012)
Supplement: Document S1. Figures S1–S7 [file mmc1.pdf]

Molecular Cell, Volume *72*

Supplemental Information

Cryo-EM Structures of MDA5-dsRNA Filaments at Different Stages of ATP Hydrolysis

Qin Yu, Kun Qu, and Yorgo Modis

CryoEM structures of MDA5-dsRNA filaments at different stages of ATP hydrolysis

Qin Yu[1], Kun Qu[2] and Yorgo Modis[1,*]

[1]Department of Medicine, University of Cambridge, MRC Laboratory of Molecular Biology, Cambridge Biomedical Campus, Cambridge, CB2 0QH, UK

[2]MRC Laboratory of Molecular Biology, Cambridge Biomedical Campus, Cambridge, CB2 0QH, UK

*Correspondence: ymodis@mrc-lmb.cam.ac.uk (Y.M.)

Supplemental Information

Supplemental Figure Legends

Supplemental Figure Legends

Figure S1. CryoEM image processing of mouse MDA5-dsRNA helical filaments, Related to **Figure 1**.

(**A**) Raw cryo-electron micrograph of MDA5-dsRNA filaments imaged in the presence of 1 mM AMPPNP. Genomic bacteriophage Φ6 genomic RNA was used as the dsRNA. The scale bar is 100 nm. The filament segment used for the Fourier transform in *(B)* is marked with a black rectangle.

(**B**) Fast Fourier transform (power spectrum) of the boxed region in *(A)* showing the helical layer lines. Red lines mark the meridian (n = 0) and first meridional reflection (n = 1). This distance between the meridian and the n = 1 layer line corresponds to the helical rise of the one-start helix.

(**C**) A selected 2D class average of MDA5-dsRNA filaments. The box size is 719 Å.

(**D**) Fast Fourier transform (power spectrum) of the 2D class average in *(C).* Red lines mark the meridian (n = 0) and the n = 1 layer line to indicate the helical rise.

(**E**) 2D class averages of MDA5-dsRNA filaments used for cryoEM reconstruction. The box size is 240 Å. This was the box size that was used in image reconstruction.

Figure S2. Data and model quality assessment by Fourier shell correlation analysis, Related to **Figures 1 and S3** and the **STAR Methods**.

(**A-C**) Fourier Shell Correlations (FSC) of reconstructions of filaments with 1 mM AMPPNP from two independently refined half-maps (map2map, in blue); and of the reconstruction from the whole dataset versus a map calculated from the refined atomic model (model2map, in red). The gold-standard cutoff (FSC = 0.143) and the FSC = 0.5 level are marked with dashed lines. The resolution values of each curve at these levels are indicated. (**A**), FSC plots for the Twist74 structure; (**B**), Twist87; (**C**), Twist91.

(**D-F**) FSC plots for cross-validation as described by Amunts et al. (Amunts et al., 2014). FSCwork (red), FSC of refined test model versus work set (half-map used in test refinement). FSCfree (blue), FSC of refined test model versus test set (half-map not used in test refinement). The FSC = 0.5 level is indicated by a dashed line. (**D**), FSCwork and FSCfree plots for the Twist74 structure; (**E**), Twist87; (**F**), Twist91.

(**G-J**) Map2map and model2map FSC plots of reconstructions from filaments in the presence of 2.5 mM AMPPNP (77˚ twist) (**G**), 10 mM ATP (73˚ twist) (**H**), 2 mM ADP-AlF_4 (88˚ twist) (**I**),

or without any added nucleotide (**J**). The gold-standard cutoff (FSC = 0.143) and the FSC = 0.5 level are marked with dashed lines. The resolution values of each curve at these levels are indicated. (**K**) Flow chart showing the workflow pipeline for cryoEM image processing, classification and model refinement, as described in the Methods.

Figure S3. Local resolution estimation for the three cryoEM reconstructions and samples of local cryoEM density, Related to **Figures 1-3 and S2**.

(**A**) Local resolution estimation for the cryoEM volumes obtained from samples in the presence of 1 mM AMPPNP, 2.5 mM AMPPNP, 10 mM ATP, 2 mM ADP-AlF_4, or without added nucleotide. The maps and resolution values were calculated in RELION 2.1 (Scheres, 2012). (**B**) Representative samples of local cryoEM density from the Twist74 structure (1 mM AMPPNP) with fitted and refined atomic models. A contour level (sdLevel) of 1.0 in USCF Chimera (Pettersen et al., 2004) was used for all panels.

Figure S4. Structural alignment of the cryoEM and crystal structures of MDA5 bound to dsRNA, Related to **Figures 4 and S5**.

The Twist74 cryoEM structure (blue) was used as the alignment reference. Twist87 is in pink, Twist91 in brown, chicken MDA5 (PDB code 5JCF) in grey and human MDA5 (PDB code 4GL2) in white. The root mean square deviations between the three cryoEM structures and the crystal structures of hMDA5 and chMDA5 range from 1.09 Å to 1.63 Å (main chain atoms), with Twist74 and chicken MDA5 bound to a 10-bp dsRNA and ADP-Mg^{2+} being the most similar.

(**A**) Side view, with the helical axis of dsRNA vertical. The regions that would form filament contacts with the pincer domain of an adjacent filament protomer are boxed. Close-ups of the boxed regions are shown in *(C)* and *(D)* as labeled in the panel.

(**B**) Top view, with the helical axis perpendicular to the image plane.

(**C**) Close-up view of the C-terminal tail, which forms most of the contacts with the second pincer helix in filament-forming Interface II in the cryoEM structures. The C-terminus of each atomic model is labeled with “C”.

(**D**) Close-up view of Hel1 interface loop, which forms most of the contact with the first pincer helix in filament-forming Interface I in the cryoEM structures.

Figure S5. Amino acid sequence alignment of MDA5 from mouse, human and chicken, Related to **Figures 4 and S4**.

The CARDs and linker between the CARDs and Hel1 are missing from all dsRNA-bound structures and are not shown in the alignment for clarity. Amino acids that were present in the experimental sample but unstructured in the crystal structures (PDB codes 4GL2 and 5JCF) or cryoEM structures of MDA5 bound to dsRNA are shown in grey typeface. Amino acids that were not present in the crystallized or imaged proteins are shown in outline font. The residues in a surface loop in Hel2i that were deleted to improve solubility (646–663 in mouse MDA5 and 644-663 in human MDA5) are highlighted in yellow. These sequences are not conserved in vertebrate MDA5 genes and their deletion does not affect the dsRNA binding, ATPase or interferon signaling activities of MDA5 (see (Berke et al., 2012; Wu et al., 2013), in supplementary information). The secondary structure of mouse MDA5 is shown above the sequences. This figure was generated in part with ESPript (Robert and Gouet, 2014).

Figure S6. Differences in the relative domain positions and filament contacts in the cryoEM structures with different helical twists, Related to **Figure 4**.

(**A**) Overview of the Twist74 structure for reference, viewed along the helical axis with the pincer domain in the foreground (long helices on the left).

(**B-F**) Protomers from the Twist74, Twist87 and Twist91 structures (not shown) were superimposed on each other using the pincer domain the reference. The adjacent protomers from each twist class are shown to highlight the differences in relative domain orientations. (**B**), Hel1 domains; (**C**), Hel2 domains; (**D**), Hel2i domains; (**E**), pincer domains; (**F**) CTDs. The highlighted domains are colored as follows: Twist74, blue; Twist87, pink; Twist91, brown. The remaining domains are shown in transparent grey representation for clarity. The outer contour of the superimposed structures is shown for reference as a black outline.

Figure S7. Filament formation and cell signaling activities of additional MDA5 filament formation interface mutants, Related to **Figures 5 and 6**.

(**A**) Representative electron micrographs of WT MDA5 and filament interface mutants not shown in **Figure 6** in the presence of 1 kb dsRNA, 1 mM AMPPNP and 5 mM $MgCl_2$. Scale bars are 100 nm. Residue numbers refer to the mouse MDA5 sequence.

(**B**) IFN-β reporter cell signaling assays of WT MDA5 and mutants not shown in **Figure 6**. The relative luciferase activity for each variant was measured as in **Figure 6**. Residue numbers

refer to the human MDA5 sequence. Error bars represent standard error of the mean between measurements; n = 3. *P < 0.05; **P < 0.01; one tailed and unpaired t-test compared to wild-type data, n = 3.

(**C-D**) MDA5 filament forming interface mutants are purified as monomers and do not form filaments or oligomers in the absence of dsRNA. (**C**) Representative electron micrographs of selected WT MDA5 and filament interface mutants in the absence of dsRNA and in the presence of 1 mM AMPPNP and 5 mM $MgCl_2$. Scale bars are 100 nm. Residue numbers refer to the mouse MDA5 sequence. The insets show a 4x magnification of an area within each respective micrograph. (**D**) Size-exclusion chromatograms for WT, D848K/F849A, E875A, E883R/K884A and D1014A/Y1015A/E1017K. Elution volumes are consistent with monomeric protein. Chromatography was performed with a Superdex 200 Increase (10/300) column (GE Healthcare).

(**E**) Fluorescence polarization assay of MDA5 binding to Mant-AMPPNP. WT MDA5 and the two mutants defective in ATPase activity (see **Figure 5**) were titrated into a solution containing poly(I:C) RNA and 10 μM Mant-AMPPNP. Binding of Mant-AMPPNP to MDA5 was measured as fluorescence polarization (λ_{ex} = 355 nm, λ_{em} = 448 nm). Error bars represent standard error of the mean between measurements in different wells; n = 3.

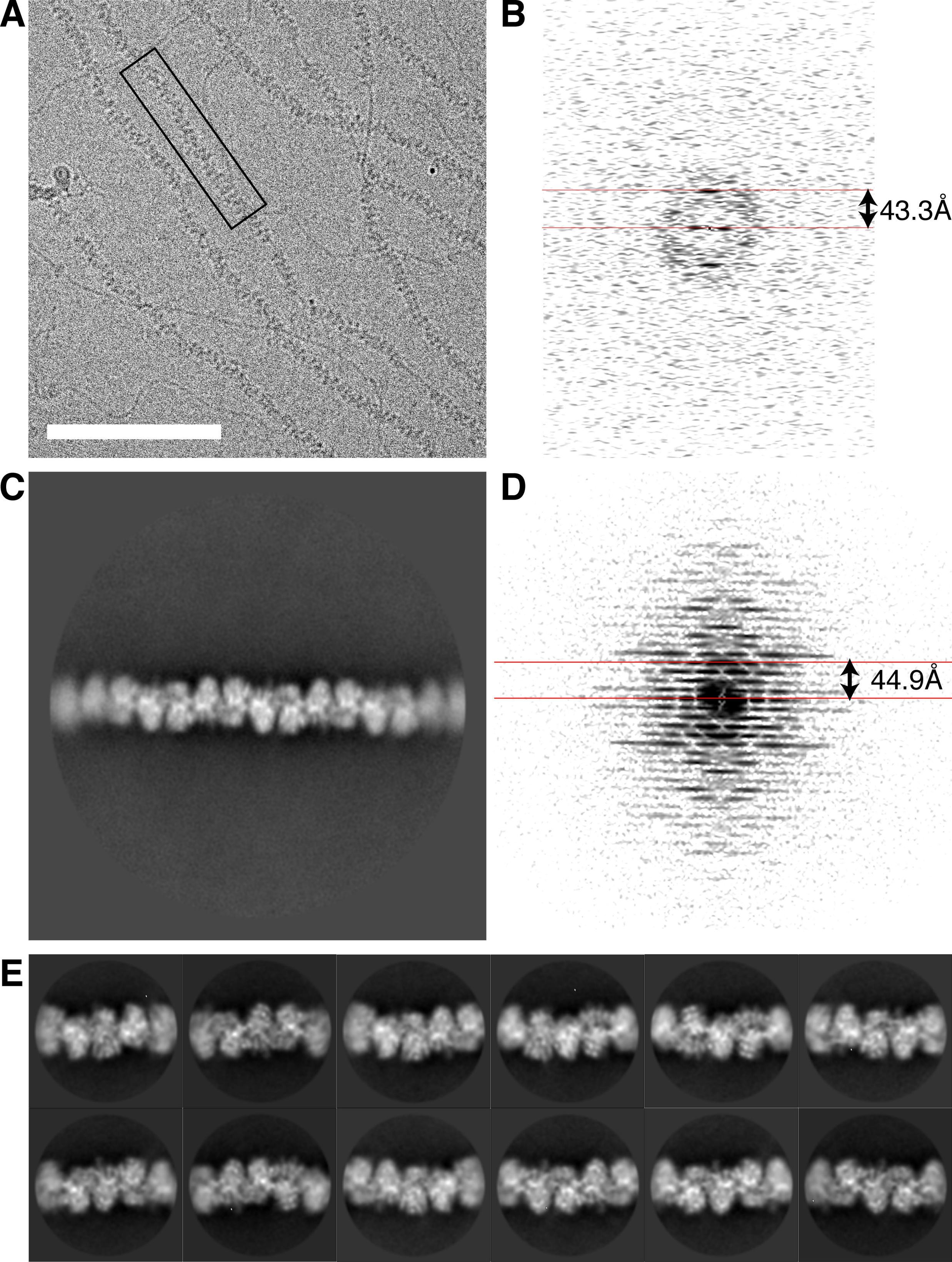

A
B
43.3Å
C
D
44.9Å
E

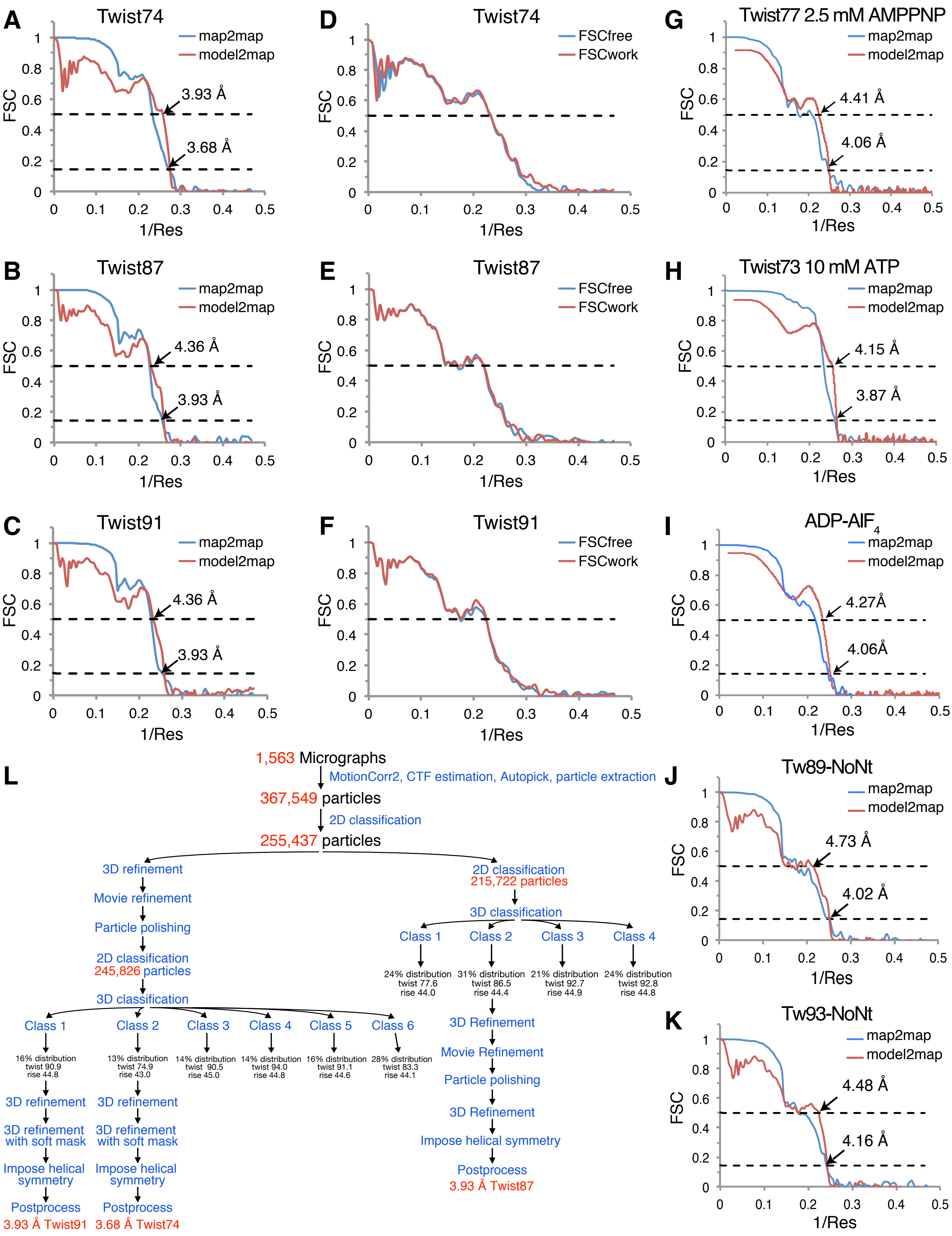
A
Twist74
map2map
model2map
3.93 Å
3.68 Å
FSC
1/Res
D
Twist74
FSCfree
FSCwork
G
Twist77 2.5 mM AMPPNP
map2map
model2map
4.41 Å
4.06 Å
B
Twist87
map2map
model2map
4.36 Å
3.93 Å
E
Twist87
FSCfree
FSCwork
H
Twist73 10 mM ATP
map2map
model2map
4.15 Å
3.87 Å
C
Twist91
map2map
model2map
4.36 Å
3.93 Å
F
Twist91
FSCfree
FSCwork
I
ADP-AlF4
map2map
model2map
4.27Å
4.06Å
L
1,563 Micrographs
MotionCorr2, CTF estimation, Autopick, particle extraction
367,549 particles
2D classification
255,437 particles
3D refinement
Movie refinement
Particle polishing
2D classification
245,826 particles
3D classification
Class 1
Class 2
Class 3
Class 4
Class 5
Class 6
16% distribution twist 90.9 rise 44.8
13% distribution twist 74.9 rise 43.0
14% distribution twist 90.5 rise 45.0
14% distribution twist 94.0 rise 44.8
16% distribution twist 91.1 rise 44.6
28% distribution twist 83.3 rise 44.1
3D refinement
3D refinement with soft mask
Impose helical symmetry
Postprocess
3.93 Å Twist91
3.68 Å Twist74
2D classification
215,722 particles
3D classification
Class 1
Class 2
Class 3
Class 4
24% distribution twist 77.6 rise 44.0
31% distribution twist 86.5 rise 44.4
21% distribution twist 92.7 rise 44.9
24% distribution twist 92.8 rise 44.8
3D Refinement
Movie Refinement
Particle polishing
3D Refinement
Impose helical symmetry
Postprocess
3.93 Å Twist87
J
Tw89-NoNt
map2map
model2map
4.73 Å
4.02 Å
K
Tw93-NoNt
map2map
model2map
4.48 Å
4.16 Å

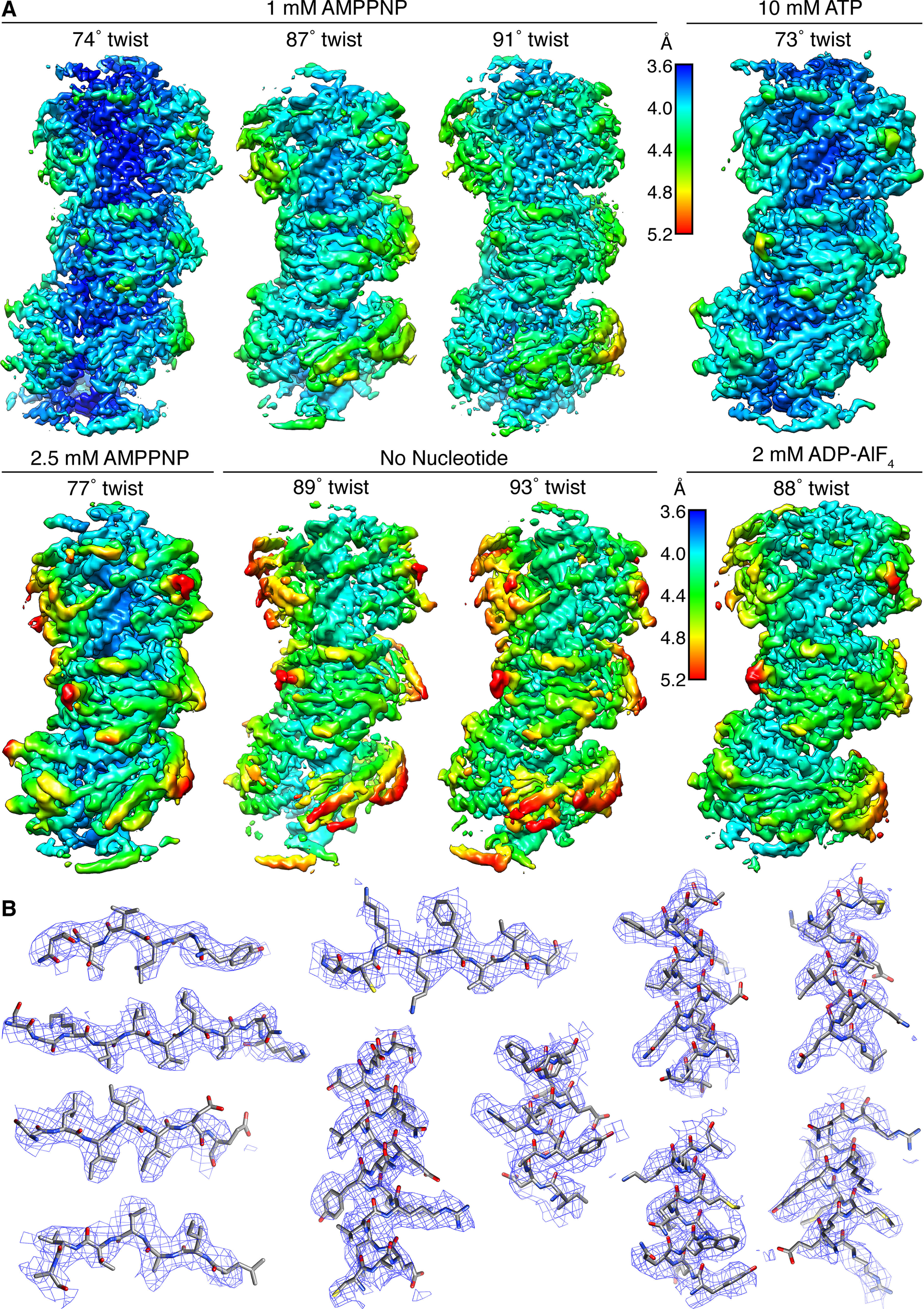

A
1 mM AMPPNP
10 mM ATP
74° twist
87° twist
91° twist
73° twist
Å
3.6
4.0
4.4
4.8
5.2
2.5 mM AMPPNP
No Nucleotide
2 mM ADP-AlF4
77° twist
89° twist
93° twist
88° twist
Å
3.6
4.0
4.4
4.8
5.2
B

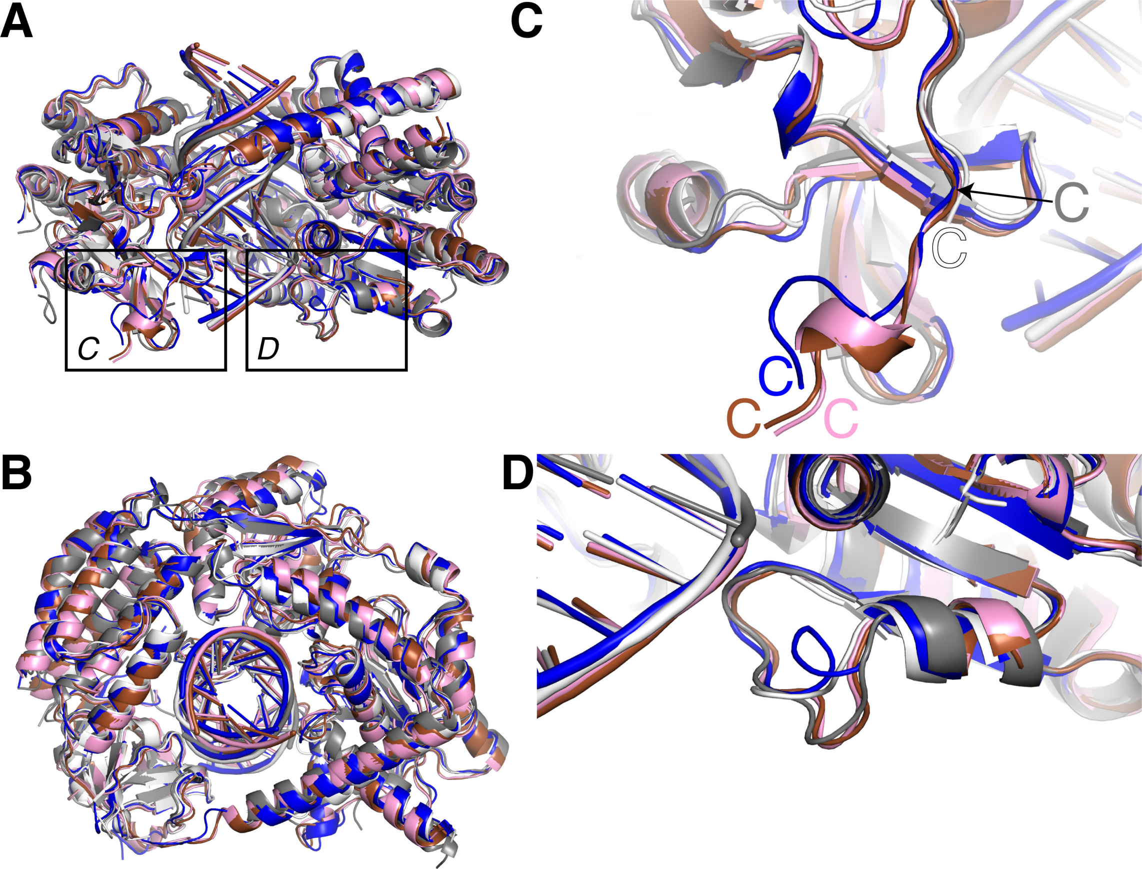
A
C
D
B
C
C
C
C
C
C
D

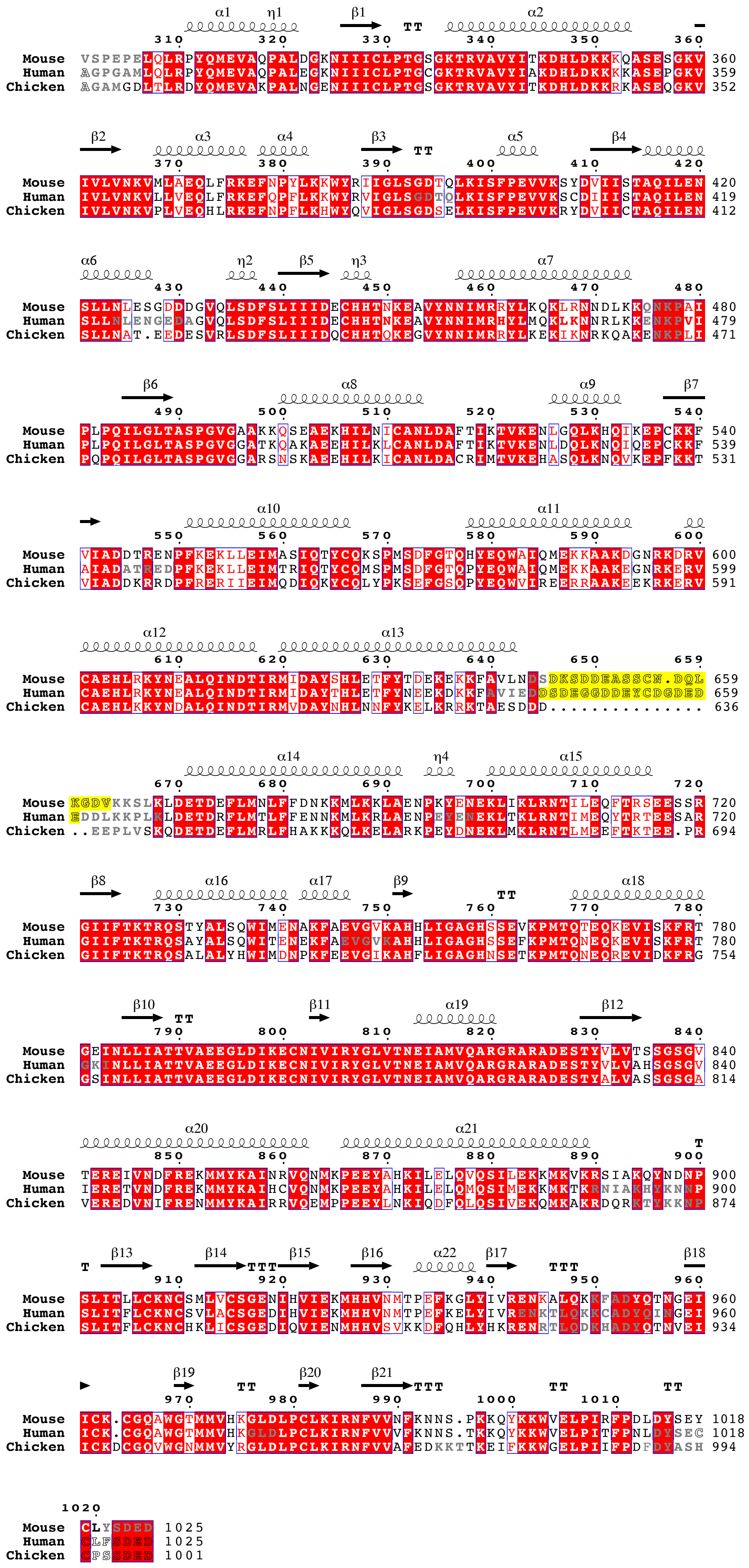

Mouse VSPEPELQLRPYQMEVAQPALDGKNIIICLPTGSGKTRVAVYITKDHLDKKKQASESGKV 360
Human AGPGAMLQLRPYQMEVAQPALEGKNIIICLPTGCGKTRVAVYIAKDHLDKKKKASEPGKV 359
Chicken AGAMGDLTLRDYQMEVAKPALNGENIIICLPTGSGKTRVAVYITKDHLDKKRKASEQGKV 352
Mouse IVLVNKVMLAEQLFRKEFNPYLKKWYRIIGLSGDTQLKISFPEVVKSYDVIISTAQILEN 420
Human IVLVNKVLLVEQLFRKEFQPFLKKWYRVIGLSGDTQLKISFPEVVKSCDIIISTAQILEN 419
Chicken IVLVNKVPLVEQHLRKEFNPFLKHWYQVIGLSGDSELKISFPEVVKRYDVIICTAQILEN 412
Mouse SLLNLESGDDDGVQLSDFSLIIIDECHHTNKEAVYNNIMRRYLKQKLRNNDLKKQNKPAI 480
Human SLLNLENGEDAGVQLSDFSLIIIDECHHTNKEAVYNNIMRHYLMQKLKNNRLKKENKPVI 479
Chicken SLLNAT.EEDESVRLSDFSLIIIDQCHHTQKEGVYNNIMRRYLKEKIKNRKQAKENKPLI 471
Mouse PLPQILGLTASPGVGAAKKQSEAEKHILNICANLDAFTIKTVKENLGQLKHQIKEPCKKF 540
Human PLPQILGLTASPGVGGATKQAKAEEHILKLCANLDAFTIKTVKENLDQLKNQIQEPCKKF 539
Chicken PQPQILGLTASPGVGGARSNSKAEEHILKICANLDACRIMTVKEHASQLKNQVKEPFKKT 531
Mouse VIADDTRENPFKEKLLEIMASIQTYCQKSPMSDFGTQHYEQWAIQMEKKAAKDGNRKDRV 600
Human AIADATREDPFKEKLLEIMTRIQTYCQMSPMSDFGTQPYEQWAIQMEKKAAKEGNRKERV 599
Chicken VIADDKRRDPFRERIIEIMQDIQKYCQLYPKSEFGSQPYEQWVIREERRAAKEEKRKERV 591
Mouse CAEHLRKYNEALQINDTIRMIDAYSHLETFYTDEKEKKFAVLNDSDKSDDEASSCN.DQL 659
Human CAEHLRKYNEALQINDTIRMIDAYTHLETFYNEEKDKKFAVIEDDSDEGGDDEYCDGDED 659
Chicken CAEHLKKYNDALQINDTIRMVDAYNHLNNFYKELKRRKTAESDDD.............. 636
Mouse KGDVKKSLKLDETDEFLMNLFFDNKKMLKKLAENPKYENEKLIKLRNTILEQFTRSEESSR 720
Human EDDLKKPLKLDETDRFLMTLFFENNKMLKKLAENPEYENEKLTKLRNTIMEQYTRTEESAR 720
Chicken ..EEPLVSKQDETDEFLMRLFHAKKKQLKELARKPEYDNEKLMKLRNTLMEEFTKTEE.PR 694
Mouse GIIFTKTRQSTYALSQWIMENAKFAEVGVKAHHLIGAGHSSEVKPMTQTEQKEVISKFRT 780
Human GIIFTKTRQSAYALSQWITENEKFAEVGVKAHHLIGAGHSSEFKPMTQNEQKEVISKFRT 780
Chicken GIIFTKTRQSALALYHWIMDNPKFEEVGIKAHFLIGAGHNSETKPMTQNEQREVIDKFRG 754
Mouse GEINLLIATTVAEEGLDIKECNIVIRYGLVTNEIAMVQARGRARADESTYVLVTSSGSGV 840
Human GKINLLIATTVAEEGLDIKECNIVIRYGLVTNEIAMVQARGRARADESTYVLVAHSGSGV 840
Chicken GSINLLIATTVAEEGLDIKECNIVIRYGLVTNEIAMVQARGRARADESTYALVASSGSGA 814
Mouse TEREIVNDFREKMMYKAINRVQNMKPEEYAHKILELQVQSILEKKMKVKRSIAKQYNDNP 900
Human IERETVNDFREKMMYKAIHCVQNMKPEEYAHKILELQMQSIMEKKMKTKRNIAKHYKNNP 900
Chicken VEREDVNIFRENMMYKAIRRVQEMPPEEYLNKIQDFQLQSIVEKQMKAKRDQRKTYKKNP 874
Mouse SLITLLCKNCSMLVCSGENIHVIEKMHHVNMTPEFKGLYIVRENKALQKKFADYQTNGEI 960
Human SLITFLCKNCSVLACSGEDIHVIEKMHHVNMTPEFKELYIVRENKTLQKKCADYQINGEI 960
Chicken SLITFLCKNCHKLICSGEDIQVIENMHHVSVKKDFQHLYHKRENRTLQDKHADYQTNVEI 934
Mouse ICK.CGQAWGTMMVHKGLDLPCLKIRNFVVNFKNNS.PKKQYKKWVELPIRFPDLDYSEY 1018
Human ICK.CGQAWGTMMVHKGLDLPCLKIRNFVVVFKNNS.TKKQYKKWVELPITFPNLDYSEC 1018
Chicken ICKDCGQVWGNMMVYRGLDLPCLKIRNFVVAFEDKKTTKEIFKKWGELPIIFPDFDYASH 994
Mouse CLYSDED 1025
Human CLFSDED 1025
Chicken CPSSDED 1001

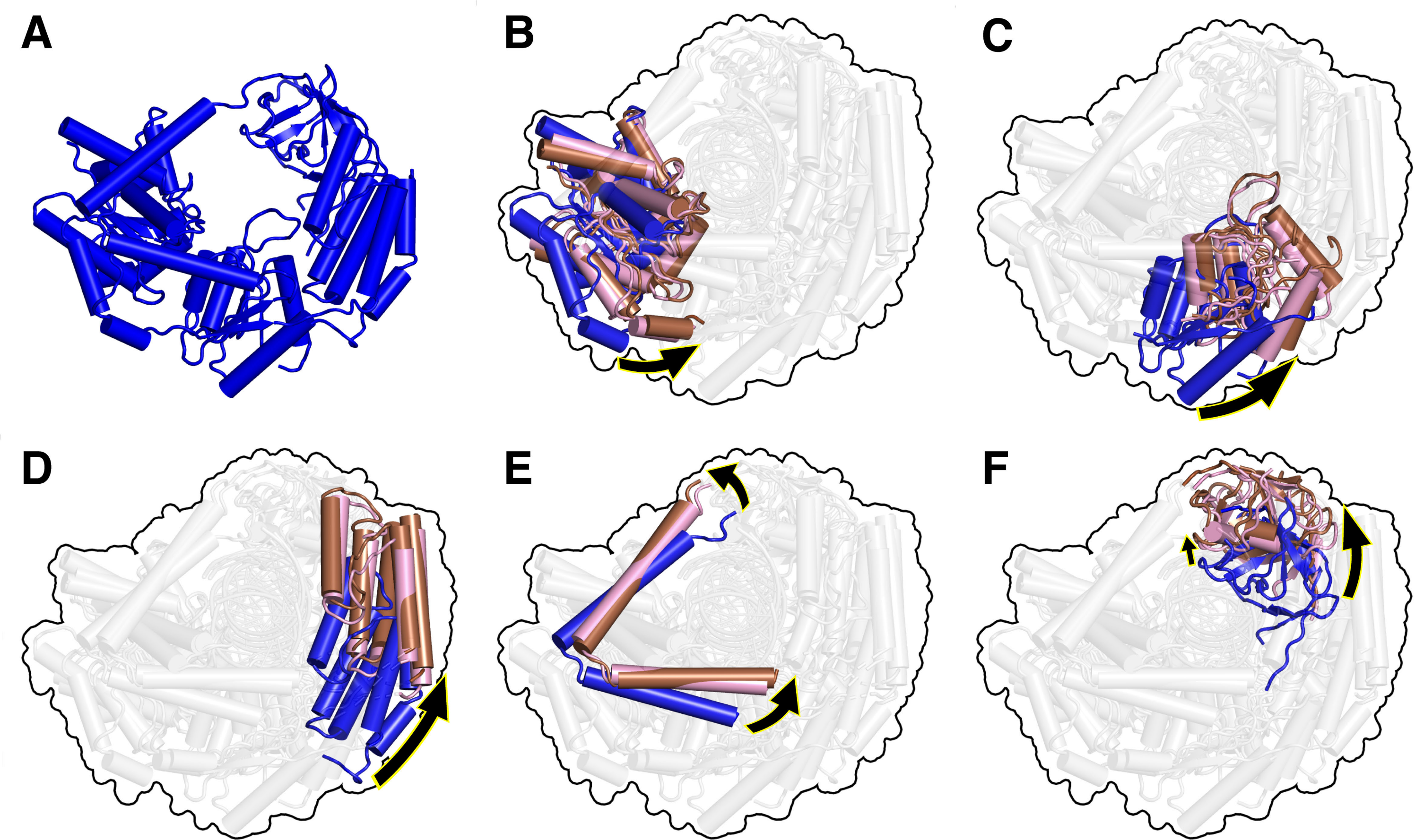

A
B
C
D
E
F

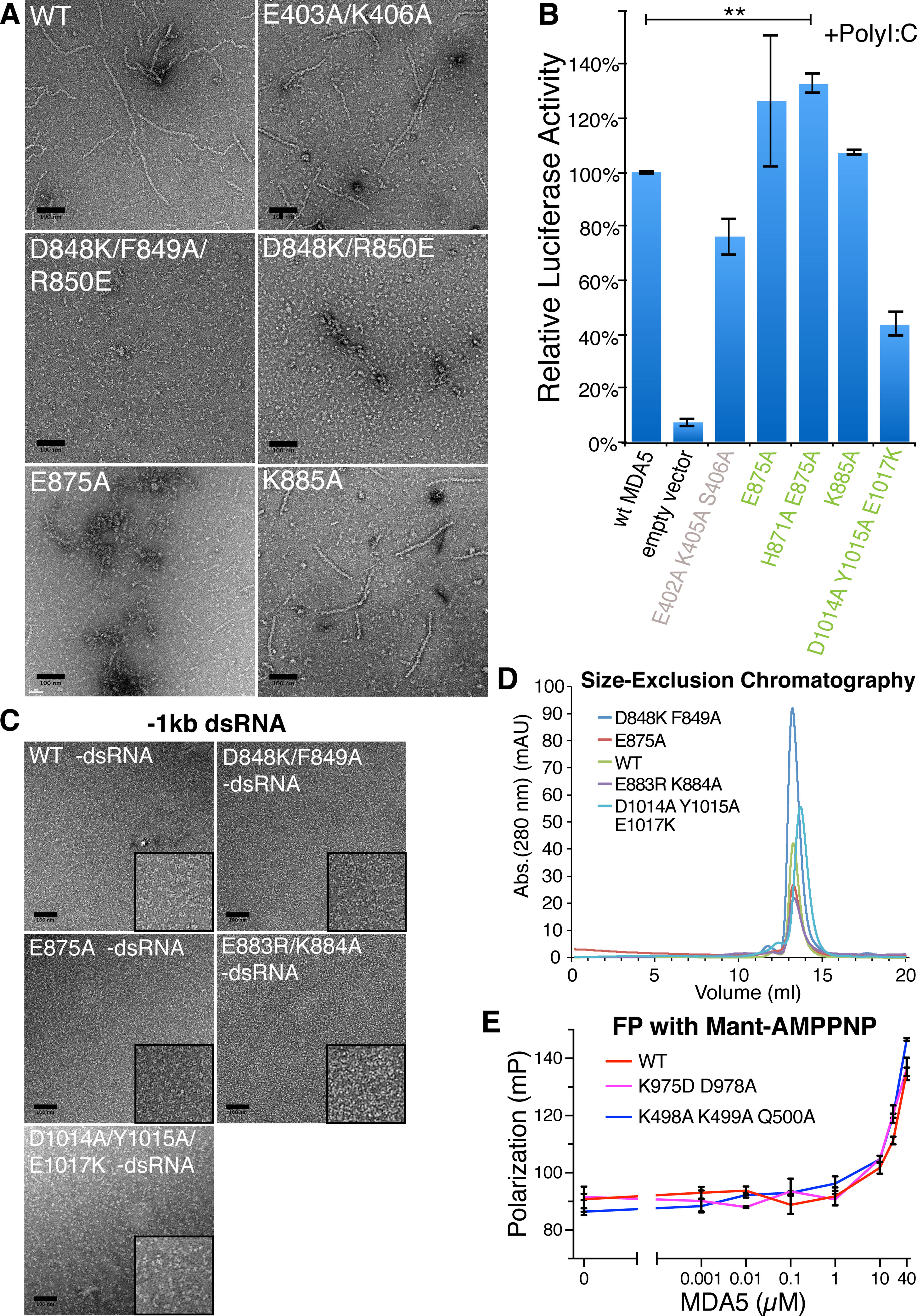
A
WT
E403A/K406A
D848K/F849A/ R850E
D848K/R850E
E875A
K885A
100 nm
B
**
+PolyI:C
Relative Luciferase Activity
0%
20%
40%
60%
80%
100%
120%
140%
wt MDA5
empty vector
E402A K405A S406A
E875A
H871A E875A
K885A
D1014A Y1015A E1017K
C
-1kb dsRNA
WT -dsRNA
D848K/F849A -dsRNA
E875A -dsRNA
E883R/K884A -dsRNA
D1014A/Y1015A/ E1017K -dsRNA
D
Size-Exclusion Chromatography
D848K F849A
E875A
WT
E883R K884A
D1014A Y1015A E1017K
Abs.(280 nm) (mAU)
Volume (ml)
0
5
10
15
20
E
FP with Mant-AMPPNP
WT
K975D D978A
K498A K499A Q500A
Polarization (mP)
80
100
120
140
MDA5 (μM)
0
0.001
0.01
0.1
1
10
40
