# Supplementary material for: Cryo-EM Structures of MDA5-dsRNA Filaments at Different Stages of ATP Hydrolysis
Source: Mol Cell. 2018 Dec 20;72(6):999–1012.e6. doi: 10.1016/j.molcel.2018.10.012 (PMC6310684; doi:10.1016/j.molcel.2018.10.012)
Supplement: Document S2. Full wwPDB/EMDataBank EM Map/Model Validation Report [file mmc2.pdf]

Click here to access/download;PDB Validation Report;D_1200009238_val-report-full-annotate_P1.pdf

CONFIDENTIAL VALIDATION REPORT

Full wwPDB/EMDataBank EM Map/Model Validation Report (i)

Apr 3, 2018 – 09:41 AM BST

PDB ID	:	6G19
EMDB ID:	:	EMD-4338
Title	:	CryoEM structure of the MDA5-dsRNA filament with 74-degree helical twist
Deposited on	:	2018-03-20
Resolution	:	3.68 Å(reported)

This is a Full wwPDB/EMDataBank EM Map/Model Validation Report.

This report is produced by the wwPDB biocuration pipeline after annotation of the structure.

We welcome your comments at *validation@mail.wwpdb.org*
A user guide is available at
https://www.wwpdb.org/validation/2017/EMValidationReportHelp
with specific help available everywhere you see the (i) symbol.

MolProbity	:	4.02b-467
Mogul	:	1.7.3 (157068), CSD as539be (2018)
Percentile statistics	:	20171227.v01 (using entries in the PDB archive December 27th 2017)
Ideal geometry (proteins)	:	Engh & Huber (2001)
Ideal geometry (DNA, RNA)	:	Parkinson et. al. (1996)
Validation Pipeline (wwPDB-VP)	:	rb-20031021

1 Overall quality at a glance (i)

The following experimental techniques were used to determine the structure:
ELECTRON MICROSCOPY

The reported resolution of this entry is 3.68 Å.

Percentile scores (ranging between 0-100) for global validation metrics of the entry are shown in the following graphic. The table shows the number of entries on which the scores are based.

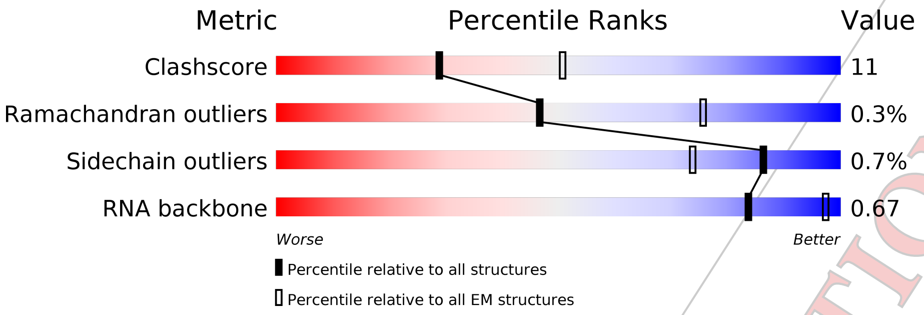

Metric	Whole archive (#Entries)	EM structures (#Entries)
Clashscore	136327	1886
Ramachandran outliers	132723	1663
Sidechain outliers	132532	1531
RNA backbone	3747	458

The table below summarises the geometric issues observed across the polymeric chains. The red, orange, yellow and green segments on the bar indicate the fraction of residues that contain outliers for >=3, 2, 1 and 0 types of geometric quality criteria. A grey segment represents the fraction of residues that are not modelled. The numeric value for each fraction is indicated below the corresponding segment, with a dot representing fractions <=5%

Mol	Chain	Length	Quality of chain
1	A	696	79% 18% • •
2	X	14	7% 93%
3	Y	14	100%

CONFIDENTIAL VALIDATION REPORT

2 Entry composition (i)

There are 5 unique types of molecules in this entry. The entry contains 10503 atoms, of which 4402 are hydrogens and 0 are deuteriums.

In the tables below, the AltConf column contains the number of residues with at least one atom in alternate conformation and the Trace column contains the number of residues modelled with at most 2 atoms.

- Molecule 1 is a protein called Interferon-induced helicase C domain-containing protein 1.

Mol	Chain	Residues	Atoms						AltConf	Trace
			Total	C	H	N	O	S		
1	A	682	9874	3468	4402	950	1019	35	0	0

There are 18 discrepancies between the modelled and reference sequences:

Chain	Residue	Modelled	Actual	Comment	Reference
A	?	-	ASP	deletion	UNP Q8R5F7
A	?	-	LYS	deletion	UNP Q8R5F7
A	?	-	SER	deletion	UNP Q8R5F7
A	?	-	ASP	deletion	UNP Q8R5F7
A	?	-	ASP	deletion	UNP Q8R5F7
A	?	-	GLU	deletion	UNP Q8R5F7
A	?	-	ALA	deletion	UNP Q8R5F7
A	?	-	SER	deletion	UNP Q8R5F7
A	?	-	SER	deletion	UNP Q8R5F7
A	?	-	CYS	deletion	UNP Q8R5F7
A	?	-	ASN	deletion	UNP Q8R5F7
A	?	-	ASP	deletion	UNP Q8R5F7
A	?	-	GLN	deletion	UNP Q8R5F7
A	?	-	LEU	deletion	UNP Q8R5F7
A	?	-	LYS	deletion	UNP Q8R5F7
A	?	-	GLY	deletion	UNP Q8R5F7
A	?	-	ASP	deletion	UNP Q8R5F7
A	?	-	VAL	deletion	UNP Q8R5F7

- Molecule 2 is a RNA chain called RNA (5'-R(P*CP*AP*AP*GP*CP*CP*GP*AP*GP*GP *AP*GP*AP*G)-3').

Mol	Chain	Residues	Atoms					AltConf	Trace
			Total	C	N	O	P		
2	X	14	308	137	64	93	14	0	0

- Molecule 3 is a RNA chain called RNA (5'-R(P*CP*UP*CP*UP*CP*CP*UP*CP*GP*GP

CONFIDENTIAL VALIDATION REPORT

*CP*UP*UP*G)-3').

Mol	Chain	Residues	Atoms					AltConf	Trace
			Total	C	N	O	P		
3	Y	14	289	129	43	103	14	0	0

- Molecule 4 is ZINC ION (three-letter code: ZN) (formula: Zn).

Mol	Chain	Residues	Atoms		AltConf
			Total	Zn	
4	A	1	1	1	0

- Molecule 5 is PHOSPHOAMINOPHOSPHONIC ACID-ADENYLATE ESTER (three-letter code: ANP) (formula: $C_{10}H_{17}N_6O_{12}P_3$).

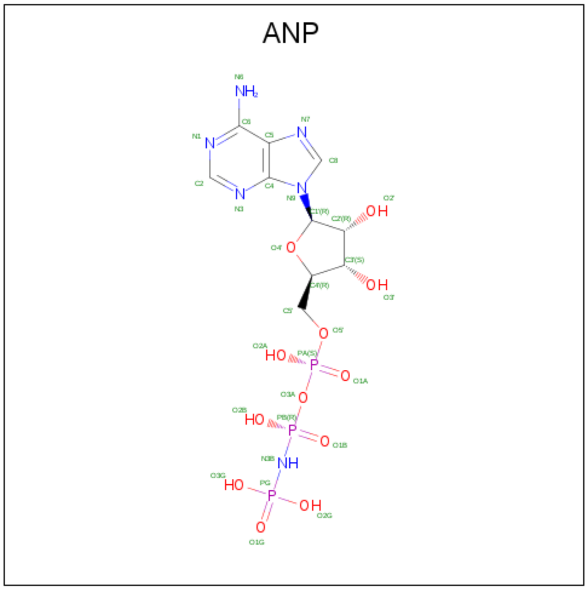

Mol	Chain	Residues	Atoms					AltConf
			Total	C	N	O	P	
5	A	1	31	10	6	12	3	0

CONFIDENTIAL VALIDATION REPORT

3 Residue-property plots ⓘ

These plots are drawn for all protein, RNA and DNA chains in the entry. The first graphic for a chain summarises the proportions of the various outlier classes displayed in the second graphic. The second graphic shows the sequence view annotated by issues in geometry. Residues are color-coded according to the number of geometric quality criteria for which they contain at least one outlier: green = 0, yellow = 1, orange = 2 and red = 3 or more. Stretches of 2 or more consecutive residues without any outlier are shown as a green connector. Residues present in the sample, but not in the model, are shown in grey.

- Molecule 1: Interferon-induced helicase C domain-containing protein 1

Chain A:

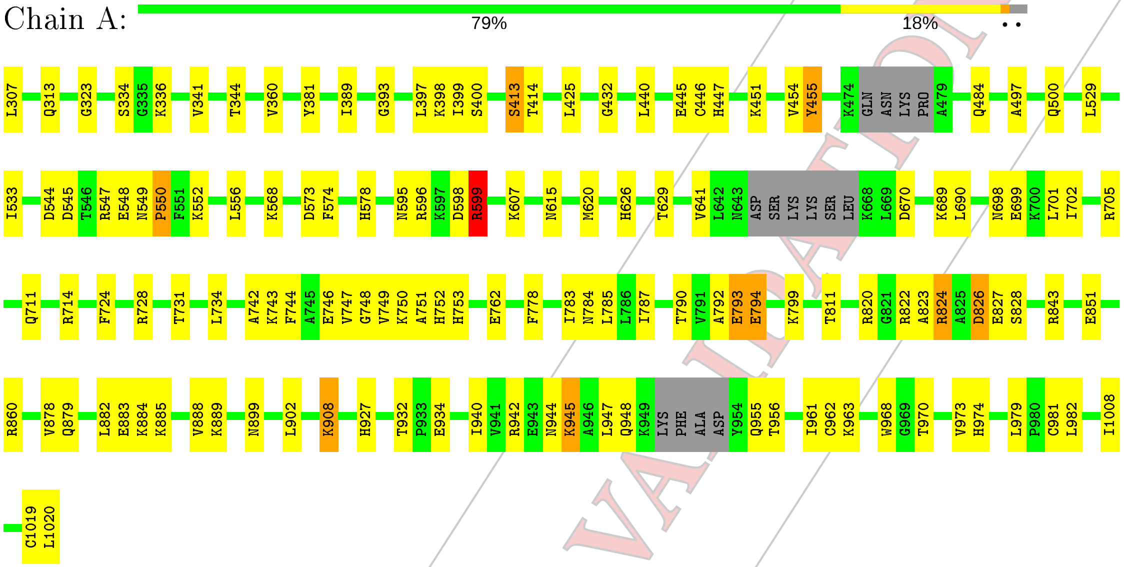

- Molecule 2: RNA (5'-R(P*CP*AP*AP*GP*CP*CP*GP*AP*GP*GP*AP*GP*AP*G)-3')

Chain X:

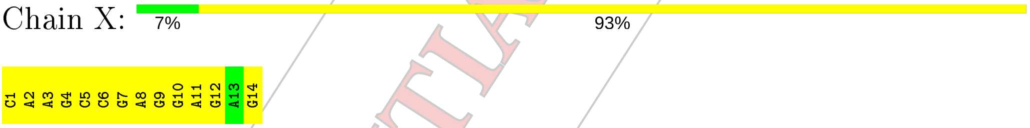

- Molecule 3: RNA (5'-R(P*CP*UP*CP*UP*CP*CP*UP*CP*GP*GP*CP*UP*UP*G)-3')

Chain Y:

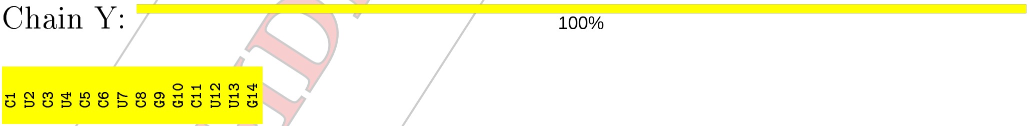

CONFIDENTIAL VALIDATION REPORT

4 Experimental information (i)

**Property**	**Value**	**Source**
Reconstruction method	HELICAL	Depositor
Imposed symmetry	HELICAL, twist=74.3022°, rise=42.8438 Å, axial sym=C1	Depositor
Number of segments used	33138	Depositor
Resolution determination method	FSC 0.143 CUT-OFF	Depositor
CTF correction method	PHASE FLIPPING AND AMPLITUDE CORRECTION	Depositor
Microscope	FEI TITAN KRIOS	Depositor
Voltage (kV)	300	Depositor
Electron dose $(e^{-}/\text{Å}^{2})$	29.85	Depositor
Minimum defocus (nm)	1800	Depositor
Maximum defocus (nm)	2700	Depositor
Magnification	75000	Depositor
Image detector	FEI FALCON III (4k x 4k)	Depositor

CONFIDENTIAL VALIDATION REPORT

5 Model quality (i)

5.1 Standard geometry (i)

Bond lengths and bond angles in the following residue types are not validated in this section: ZN, ANP

The Z score for a bond length (or angle) is the number of standard deviations the observed value is removed from the expected value. A bond length (or angle) with $|Z| > 5$ is considered an outlier worth inspection. RMSZ is the root-mean-square of all Z scores of the bond lengths (or angles).

Mol	Chain	Bond lengths		Bond angles	
		RMSZ	$\#\|Z\| >2$	RMSZ	$\#\|Z\| >2$
1	A	0.57	3/5558 (0.1%)	0.69	9/7469 (0.1%)
2	X	0.77	0/346	0.88	0/539
3	Y	0.79	0/319	1.01	1/493 (0.2%)
All	All	0.60	3/6223 (0.0%)	0.73	10/8501 (0.1%)

Chiral center outliers are detected by calculating the chiral volume of a chiral center and verifying if the center is modelled as a planar moiety or with the opposite hand.A planarity outlier is detected by checking planarity of atoms in a peptide group, atoms in a mainchain group or atoms of a sidechain that are expected to be planar.

Mol	Chain	#Chirality outliers	#Planarity outliers
1	A	0	5

All (3) bond length outliers are listed below:

Mol	Chain	Res	Type	Atoms	Z	Observed(Å)	Ideal(Å)
1	A	455	TYR	CD2-CE2	-6.62	1.29	1.39
1	A	455	TYR	CB-CG	-5.17	1.43	1.51
1	A	455	TYR	CD1-CE1	-5.12	1.31	1.39

All (10) bond angle outliers are listed below:

Mol	Chain	Res	Type	Atoms	Z	Observed(^{o})	Ideal(^{o})
1	A	599	ARG	NE-CZ-NH2	-13.92	113.34	120.30
1	A	599	ARG	NE-CZ-NH1	9.87	125.23	120.30
1	A	824	ARG	NE-CZ-NH2	-8.71	115.94	120.30
1	A	497	ALA	C-N-CA	8.60	143.19	121.70
1	A	794	GLU	C-N-CA	7.89	138.87	122.30
1	A	793	GLU	C-N-CA	6.26	137.35	121.70
1	A	599	ARG	CG-CD-NE	-5.62	100.00	111.80

Continued on next page...

CONFIDENTIAL VALIDATION REPORT

Continued from previous page...

Mol	Chain	Res	Type	Atoms	Z	Observed(^{o})	Ideal(^{o})
3	Y	12	U	C5-C6-N1	-5.34	120.03	122.70
1	A	451	LYS	C-N-CA	5.26	134.86	121.70
1	A	908	LYS	CD-CE-NZ	5.19	123.63	111.70

There are no chirality outliers.

All (5) planarity outliers are listed below:

Mol	Chain	Res	Type	Group
1	A	413	SER	Peptide
1	A	454	VAL	Peptide
1	A	548	GLU	Peptide
1	A	550	PRO	Peptide
1	A	793	GLU	Peptide

5.2 Too-close contacts (i)

In the following table, the Non-H and H(model) columns list the number of non-hydrogen atoms and hydrogen atoms in the chain respectively. The H(added) column lists the number of hydrogen atoms added and optimized by MolProbity. The Clashes column lists the number of clashes within the asymmetric unit, whereas Symm-Clashes lists symmetry related clashes.

Mol	Chain	Non-H	H(model)	H(added)	Clashes	Symm-Clashes
1	A	5472	4402	5596	103	0
2	X	308	0	155	22	0
3	Y	289	0	150	19	0
4	A	1	0	0	0	0
5	A	31	0	13	2	0
All	All	6101	4402	5914	135	0

The all-atom clashscore is defined as the number of clashes found per 1000 atoms (including hydrogen atoms). The all-atom clashscore for this structure is 11.

All (135) close contacts within the same asymmetric unit are listed below, sorted by their clash magnitude.

Atom-1	Atom-2	Interatomic distance (Å)	Clash overlap (Å)
1:A:641:VAL:HG11	1:A:963:LYS:O	1.74	0.88
1:A:823:ALA:HB1	1:A:828:SER:HB2	1.67	0.74
1:A:843:ARG:CZ	2:X:12:G:H5'	2.19	0.72
1:A:547:ARG:NH2	1:A:699:GLU:OE2	2.23	0.72

Continued on next page...

CONFIDENTIAL VALIDATION REPORT

Continued from previous page...

Atom-1	Atom-2	Interatomic distance (Å)	Clash overlap (Å)
1:A:944:ASN:H	1:A:955:GLN:HG2	1.54	0.72
1:A:932:THR:OG1	1:A:934:GLU:OE1	2.10	0.69
1:A:701:LEU:O	1:A:705:ARG:HG3	1.95	0.66
2:X:1:C:H2'	2:X:2:A:H8	1.61	0.66
1:A:336:LYS:NZ	5:A:1102:ANP:O3G	2.30	0.65
1:A:341:VAL:O	1:A:344:THR:OG1	2.15	0.64
1:A:389:ILE:HD11	1:A:397:LEU:HD13	1.80	0.64
1:A:413:SER:OG	1:A:414:THR:O	2.09	0.64
1:A:724:PHE:HE1	1:A:792:ALA:HB3	1.62	0.64
1:A:744:PHE:O	1:A:749:VAL:HB	1.98	0.63
1:A:974:HIS:HB3	1:A:979:LEU:HD21	1.81	0.63
1:A:607:LYS:NZ	1:A:762:GLU:OE2	2.33	0.62
1:A:792:ALA:O	1:A:822:ARG:NH2	2.32	0.61
1:A:970:THR:HG21	2:X:3:A:H4'	1.82	0.61
1:A:824:ARG:N	1:A:824:ARG:HD2	2.16	0.61
1:A:981:CYS:SG	1:A:982:LEU:N	2.74	0.61
1:A:962:CYS:SG	1:A:963:LYS:N	2.73	0.61
3:Y:2:U:H2'	3:Y:3:C:C6	2.36	0.61
1:A:549:ASN:HB2	1:A:550:PRO:HD3	1.81	0.61
1:A:843:ARG:NH2	2:X:11:A:O3'	2.33	0.61
1:A:899:ASN:HB3	1:A:902:LEU:HD13	1.82	0.60
1:A:945:LYS:O	1:A:948:GLN:HG3	2.03	0.58
1:A:749:VAL:HG13	1:A:785:LEU:HD22	1.86	0.58
1:A:885:LYS:O	1:A:889:LYS:HG2	2.03	0.58
3:Y:1:C:H2'	3:Y:2:U:H6	1.68	0.58
1:A:945:LYS:H	1:A:945:LYS:HD3	1.69	0.57
1:A:947:LEU:HD13	1:A:956:THR:H	1.70	0.57
1:A:899:ASN:HB2	1:A:1008:ILE:CG2	2.33	0.57
1:A:1019:CYS:SG	1:A:1020:LEU:N	2.75	0.57
3:Y:1:C:H2'	3:Y:2:U:C6	2.38	0.57
1:A:751:ALA:HB2	1:A:785:LEU:HD21	1.86	0.57
1:A:724:PHE:CE1	1:A:792:ALA:HB3	2.40	0.57
1:A:927:HIS:HE1	2:X:4:G:H1'	1.70	0.57
1:A:945:LYS:HA	1:A:948:GLN:HG3	1.87	0.57
2:X:11:A:H2'	2:X:12:G:C8	2.40	0.56
3:Y:7:U:O2'	3:Y:8:C:H5'	2.05	0.56
2:X:11:A:H2'	2:X:12:G:H8	1.70	0.56
1:A:979:LEU:HD23	1:A:979:LEU:H	1.71	0.55
1:A:445:GLU:OE1	1:A:447:HIS:NE2	2.40	0.55
1:A:313:GLN:NE2	1:A:334:SER:O	2.38	0.55
1:A:927:HIS:CE1	2:X:4:G:H1'	2.41	0.55

Continued on next page...

CONFIDENTIAL VALIDATION REPORT

Continued from previous page...

Atom-1	Atom-2	Interatomic distance (Å)	Clash overlap (Å)
1:A:752:HIS:ND1	1:A:753:HIS:O	2.39	0.55
1:A:752:HIS:HB2	1:A:783:ILE:HG12	1.89	0.54
1:A:641:VAL:HA	1:A:940:ILE:CD1	2.37	0.54
5:A:1102:ANP:N3B	5:A:1102:ANP:O1A	2.38	0.54
1:A:307:LEU:HB3	1:A:381:TYR:CD2	2.42	0.54
1:A:578:HIS:ND1	2:X:14:G:H4'	2.23	0.53
1:A:547:ARG:HB3	1:A:549:ASN:OD1	2.08	0.53
1:A:568:LYS:HE2	1:A:598:ASP:OD1	2.08	0.53
1:A:573:ASP:OD1	1:A:574:PHE:N	2.40	0.53
1:A:778:PHE:CE2	1:A:799:LYS:HB3	2.44	0.53
1:A:552:LYS:HG2	1:A:556:LEU:CD1	2.39	0.52
1:A:728:ARG:O	1:A:731:THR:OG1	2.19	0.52
2:X:1:C:H2'	2:X:2:A:C8	2.43	0.52
1:A:945:LYS:HA	1:A:948:GLN:CG	2.39	0.52
1:A:822:ARG:O	1:A:824:ARG:HD2	2.10	0.52
1:A:899:ASN:HB2	1:A:1008:ILE:HG22	1.92	0.51
1:A:731:THR:HG22	1:A:787:ILE:HG22	1.92	0.51
1:A:711:GLN:OE1	1:A:714:ARG:NH2	2.43	0.50
3:Y:2:U:H2'	3:Y:3:C:H6	1.75	0.50
1:A:790:THR:HG1	3:Y:6:C:HO2'	1.60	0.50
2:X:9:G:O2'	2:X:10:G:H5'	2.12	0.49
2:X:7:G:O2'	2:X:8:A:H5'	2.12	0.49
1:A:596:ARG:NH1	1:A:670:ASP:OD2	2.43	0.49
1:A:974:HIS:CB	1:A:979:LEU:HD21	2.43	0.49
1:A:545:ASP:HB3	1:A:699:GLU:CD	2.33	0.48
3:Y:13:U:H2'	3:Y:14:G:O4'	2.14	0.48
1:A:778:PHE:HE2	1:A:799:LYS:HB3	1.80	0.47
3:Y:5:C:O2'	3:Y:6:C:H5'	2.14	0.47
2:X:5:C:O2'	2:X:6:C:H5'	2.15	0.47
1:A:811:THR:O	2:X:11:A:H4'	2.15	0.47
1:A:743:LYS:O	1:A:747:VAL:HG23	2.13	0.47
1:A:824:ARG:H	1:A:824:ARG:HD2	1.81	0.46
1:A:962:CYS:HB2	1:A:968:TRP:CE2	2.51	0.46
1:A:393:GLY:HA2	1:A:398:LYS:HD3	1.98	0.46
1:A:820:ARG:NH2	1:A:851:GLU:OE2	2.48	0.46
1:A:878:VAL:O	1:A:882:LEU:HD13	2.15	0.46
1:A:947:LEU:HD13	1:A:955:GLN:HA	1.97	0.46
1:A:749:VAL:HG13	1:A:785:LEU:CD2	2.46	0.46
1:A:550:PRO:HB2	1:A:690:LEU:HD23	1.97	0.46
1:A:879:GLN:O	1:A:883:GLU:HG3	2.15	0.45
1:A:399:ILE:HG13	1:A:400:SER:N	2.31	0.45

Continued on next page...

CONFIDENTIAL VALIDATION REPORT

Continued from previous page...

Atom-1	Atom-2	Interatomic distance (Å)	Clash overlap (Å)
1:A:942:ARG:HD2	1:A:961:ILE:HG21	1.99	0.45
1:A:529:LEU:O	1:A:533:ILE:N	2.45	0.44
1:A:750:LYS:O	1:A:784:ASN:N	2.48	0.44
2:X:3:A:H2’	2:X:4:G:O4’	2.17	0.44
1:A:544:ASP:OD1	1:A:545:ASP:N	2.50	0.44
3:Y:10:G:O2’	3:Y:11:C:H5’	2.18	0.44
2:X:6:C:H2’	2:X:7:G:H8	1.83	0.44
3:Y:3:C:O2’	3:Y:4:U:H5’	2.17	0.44
1:A:545:ASP:OD1	1:A:545:ASP:N	2.50	0.44
1:A:899:ASN:HB2	1:A:1008:ILE:HG21	1.97	0.44
1:A:626:HIS:O	1:A:629:THR:OG1	2.32	0.43
2:X:8:A:O2’	2:X:9:G:H5’	2.18	0.43
1:A:599:ARG:NH2	1:A:908:LYS:HG2	2.34	0.43
2:X:2:A:O2’	2:X:3:A:H5’	2.18	0.43
1:A:620:MET:SD	1:A:620:MET:N	2.91	0.43
3:Y:4:U:H2’	3:Y:5:C:H6	1.84	0.43
2:X:8:A:H2’	2:X:9:G:C8	2.54	0.42
3:Y:4:U:H2’	3:Y:5:C:C6	2.54	0.42
1:A:728:ARG:NH2	3:Y:7:U:OP2	2.50	0.42
1:A:446:CYS:HA	1:A:455:TYR:OH	2.19	0.42
1:A:500:GLN:NE2	1:A:860:ARG:HD2	2.35	0.42
1:A:944:ASN:H	1:A:955:GLN:CG	2.28	0.42
1:A:973:VAL:O	3:Y:14:G:H5’	2.19	0.42
1:A:360:VAL:HG12	1:A:440:LEU:HB3	2.01	0.42
3:Y:4:U:O2’	3:Y:5:C:H5’	2.19	0.42
2:X:6:C:H2’	2:X:7:G:C8	2.55	0.42
1:A:698:ASN:OD1	1:A:699:GLU:N	2.53	0.42
1:A:734:LEU:HB2	1:A:787:ILE:HD13	2.02	0.42
1:A:552:LYS:HG2	1:A:556:LEU:HD13	2.02	0.41
1:A:742:ALA:O	1:A:746:GLU:HG3	2.20	0.41
1:A:714:ARG:NH2	1:A:827:GLU:O	2.52	0.41
1:A:425:LEU:HB2	1:A:432:GLY:HA3	2.03	0.41
1:A:689:LYS:HA	1:A:689:LYS:HE2	2.01	0.41
1:A:702:ILE:HA	1:A:705:ARG:HD3	2.02	0.41
1:A:826:ASP:O	1:A:827:GLU:HB2	2.19	0.41
1:A:947:LEU:CD1	1:A:956:THR:H	2.32	0.41
1:A:323:GLY:O	1:A:484:GLN:NE2	2.53	0.41
1:A:826:ASP:OD1	1:A:827:GLU:N	2.49	0.41
1:A:884:LYS:O	1:A:888:VAL:HG23	2.20	0.41
1:A:751:ALA:HB2	1:A:785:LEU:CD2	2.51	0.41
3:Y:8:C:O2’	3:Y:9:G:H5’	2.20	0.41

Continued on next page...

CONFIDENTIAL VALIDATION REPORT

Continued from previous page...

Atom-1	Atom-2	Interatomic distance (Å)	Clash overlap (Å)
2:X:4:G:O2'	2:X:5:C:H5'	2.21	0.41
1:A:751:ALA:HA	1:A:783:ILE:HG23	2.04	0.41
2:X:1:C:O2'	2:X:2:A:H5'	2.21	0.40
3:Y:3:C:H2'	3:Y:4:U:C6	2.57	0.40
1:A:748:GLY:O	1:A:784:ASN:ND2	2.50	0.40
1:A:962:CYS:HB2	1:A:968:TRP:CZ2	2.56	0.40
3:Y:10:G:C2'	3:Y:11:C:H5'	2.52	0.40
3:Y:9:G:H2'	3:Y:10:G:C8	2.56	0.40

There are no symmetry-related clashes.

5.3 Torsion angles (i)

5.3.1 Protein backbone (i)

In the following table, the Percentiles column shows the percent Ramachandran outliers of the chain as a percentile score with respect to all PDB entries followed by that with respect to all EM entries.

The Analysed column shows the number of residues for which the backbone conformation was analysed, and the total number of residues.

Mol	Chain	Analysed	Favoured	Allowed	Outliers	Percentiles	
1	A	674/696 (97%)	603 (90%)	69 (10%)	2 (0%)	43	78

All (2) Ramachandran outliers are listed below:

Mol	Chain	Res	Type
1	A	794	GLU
1	A	826	ASP

5.3.2 Protein sidechains (i)

In the following table, the Percentiles column shows the percent sidechain outliers of the chain as a percentile score with respect to all PDB entries followed by that with respect to all EM entries.

The Analysed column shows the number of residues for which the sidechain conformation was analysed, and the total number of residues.

CONFIDENTIAL VALIDATION REPORT

Mol	Chain	Analysed	Rotameric	Outliers	Percentiles	
1	A	608/625 (97%)	604 (99%)	4 (1%)	85	93

All (4) residues with a non-rotameric sidechain are listed below:

Mol	Chain	Res	Type
1	A	595	ASN
1	A	599	ARG
1	A	615	ASN
1	A	945	LYS

Some sidechains can be flipped to improve hydrogen bonding and reduce clashes. All (4) such sidechains are listed below:

Mol	Chain	Res	Type
1	A	563	GLN
1	A	595	ASN
1	A	678	ASN
1	A	957	ASN

5.3.3 RNA (i)

Mol	Chain	Analysed	Backbone Outliers	Pucker Outliers
2	X	13/14 (92%)	0	0
3	Y	13/14 (92%)	0	0
All	All	26/28 (92%)	0	0

There are no RNA backbone outliers to report.

There are no RNA pucker outliers to report.

5.4 Non-standard residues in protein, DNA, RNA chains (i)

There are no non-standard protein/DNA/RNA residues in this entry.

5.5 Carbohydrates (i)

There are no carbohydrates in this entry.

CONFIDENTIAL VALIDATION REPORT

5.6 Ligand geometry (i)

Of 2 ligands modelled in this entry, 1 is monoatomic - leaving 1 for Mogul analysis.

In the following table, the Counts columns list the number of bonds (or angles) for which Mogul statistics could be retrieved, the number of bonds (or angles) that are observed in the model and the number of bonds (or angles) that are defined in the Chemical Component Dictionary. The Link column lists molecule types, if any, to which the group is linked. The Z score for a bond length (or angle) is the number of standard deviations the observed value is removed from the expected value. A bond length (or angle) with $|Z| > 2$ is considered an outlier worth inspection. RMSZ is the root-mean-square of all Z scores of the bond lengths (or angles).

Mol	Type	Chain	Res	Link	Bond lengths			Bond angles		
					Counts	RMSZ	$\#\|Z\| > 2$	Counts	RMSZ	$\#\|Z\| > 2$
5	ANP	A	1102	-	29,33,33	2.71	6 (20%)	29,52,52	1.10	1 (3%)

In the following table, the Chirals column lists the number of chiral outliers, the number of chiral centers analysed, the number of these observed in the model and the number defined in the Chemical Component Dictionary. Similar counts are reported in the Torsion and Rings columns. '-' means no outliers of that kind were identified.

Mol	Type	Chain	Res	Link	Chirals	Torsions	Rings
5	ANP	A	1102	-	-	1/13/38/38	0/3/3/3

All (6) bond length outliers are listed below:

Mol	Chain	Res	Type	Atoms	Z	Observed(Å)	Ideal(Å)
5	A	1102	ANP	PB-O2B	-2.58	1.49	1.56
5	A	1102	ANP	PG-O2G	-2.52	1.49	1.56
5	A	1102	ANP	PB-O3A	-2.41	1.56	1.59
5	A	1102	ANP	PG-N3B	2.25	1.69	1.63
5	A	1102	ANP	PB-O1B	8.69	1.55	1.46
5	A	1102	ANP	PG-O1G	10.15	1.57	1.46

All (1) bond angle outliers are listed below:

Mol	Chain	Res	Type	Atoms	Z	Observed(^{o})	Ideal(^{o})
5	A	1102	ANP	PA-O3A-PB	-3.86	118.87	132.40

There are no chirality outliers.

All (1) torsion outliers are listed below:

Mol	Chain	Res	Type	Atoms
5	A	1102	ANP	O1B-PB-N3B-PG

CONFIDENTIAL VALIDATION REPORT

There are no ring outliers.

1 monomer is involved in 2 short contacts:

Mol	Chain	Res	Type	Clashes	Symm-Clashes
5	A	1102	ANP	2	0

5.7 Other polymers (i)

There are no such residues in this entry.

5.8 Polymer linkage issues (i)

There are no chain breaks in this entry.

CONFIDENTIAL VALIDATION REPORT

Click here to access/download;PDB Validation Report;D_1200009290_val-report-full-annotate_P1.pdf

Full wwPDB/EMDataBank EM Map/Model Validation Report (i)

CONFIDENTIAL VALIDATION REPORT

Apr 4, 2018 – 10:25 AM BST

PDB ID	:	6G1S
EMDB ID:	:	EMD-4340
Title	:	CryoEM structure of the MDA5-dsRNA filament with 87-degree helical twist
Deposited on	:	2018-03-21
Resolution	:	3.93 Å(reported)

This is a Full wwPDB/EMDataBank EM Map/Model Validation Report.

This report is produced by the wwPDB biocuration pipeline after annotation of the structure.

We welcome your comments at *validation@mail.wwpdb.org*
A user guide is available at
https://www.wwpdb.org/validation/2017/EMValidationReportHelp
with specific help available everywhere you see the (i) symbol.

MolProbity	:	4.02b-467
Percentile statistics	:	20171227.v01 (using entries in the PDB archive December 27th 2017)
Ideal geometry (proteins)	:	Engh & Huber (2001)
Ideal geometry (DNA, RNA)	:	Parkinson et. al. (1996)
Validation Pipeline (wwPDB-VP)	:	rb-20031021

1 Overall quality at a glance (i)

The following experimental techniques were used to determine the structure:
ELECTRON MICROSCOPY

The reported resolution of this entry is 3.93 Å.

Percentile scores (ranging between 0-100) for global validation metrics of the entry are shown in the following graphic. The table shows the number of entries on which the scores are based.

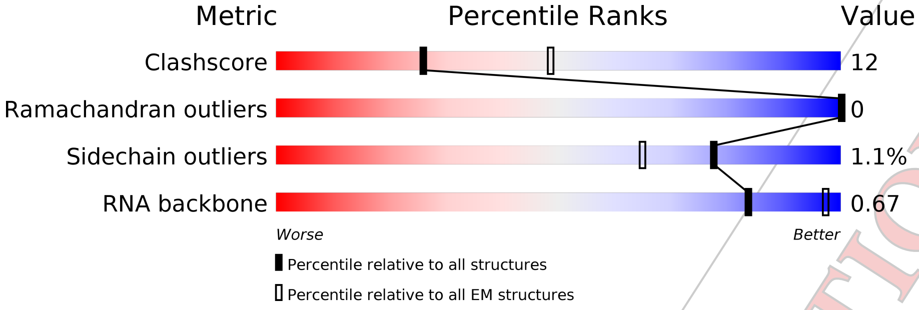

Metric	Whole archive (#Entries)	EM structures (#Entries)
Clashscore	136327	1886
Ramachandran outliers	132723	1663
Sidechain outliers	132532	1531
RNA backbone	3747	458

The table below summarises the geometric issues observed across the polymeric chains. The red, orange, yellow and green segments on the bar indicate the fraction of residues that contain outliers for >=3, 2, 1 and 0 types of geometric quality criteria. A grey segment represents the fraction of residues that are not modelled. The numeric value for each fraction is indicated below the corresponding segment, with a dot representing fractions <=5%

Mol	Chain	Length	Quality of chain
1	A	693	72% 21% 7%
2	X	15	7% 93%
3	Y	15	7% 93%

CONFIDENTIAL VALIDATION REPORT

2 Entry composition (i)

There are 4 unique types of molecules in this entry. The entry contains 9891 atoms, of which 4096 are hydrogens and 0 are deuteriums.

In the tables below, the AltConf column contains the number of residues with at least one atom in alternate conformation and the Trace column contains the number of residues modelled with at most 2 atoms.

- Molecule 1 is a protein called Interferon-induced helicase C domain-containing protein 1.

Mol	Chain	Residues	Atoms						AltConf	Trace
			Total	C	H	N	O	S		
1	A	645	9251	3279	4096	893	949	34	0	0

There are 18 discrepancies between the modelled and reference sequences:

Chain	Residue	Modelled	Actual	Comment	Reference
A	?	-	ASP	deletion	UNP Q8R5F7
A	?	-	LYS	deletion	UNP Q8R5F7
A	?	-	SER	deletion	UNP Q8R5F7
A	?	-	ASP	deletion	UNP Q8R5F7
A	?	-	ASP	deletion	UNP Q8R5F7
A	?	-	GLU	deletion	UNP Q8R5F7
A	?	-	ALA	deletion	UNP Q8R5F7
A	?	-	SER	deletion	UNP Q8R5F7
A	?	-	SER	deletion	UNP Q8R5F7
A	?	-	CYS	deletion	UNP Q8R5F7
A	?	-	ASN	deletion	UNP Q8R5F7
A	?	-	ASP	deletion	UNP Q8R5F7
A	?	-	GLN	deletion	UNP Q8R5F7
A	?	-	LEU	deletion	UNP Q8R5F7
A	?	-	LYS	deletion	UNP Q8R5F7
A	?	-	GLY	deletion	UNP Q8R5F7
A	?	-	ASP	deletion	UNP Q8R5F7
A	?	-	VAL	deletion	UNP Q8R5F7

- Molecule 2 is a RNA chain called RNA (5'-R(P*UP*CP*CP*AP*UP*GP*CP*GP*CP*AP *UP*GP*AP*CP*G)-3').

Mol	Chain	Residues	Atoms					AltConf	Trace
			Total	C	N	O	P		
2	X	15	318	142	56	105	15	0	0

- Molecule 3 is a RNA chain called RNA (5'-R(P*CP*GP*UP*CP*AP*UP*GP*CP*GP*CP

CONFIDENTIAL VALIDATION REPORT

*AP*UP*GP*GP*A)-3').

Mol	Chain	Residues	Atoms					AltConf	Trace
			Total	C	N	O	P		
3	Y	15	321	143	58	105	15	0	0

- Molecule 4 is ZINC ION (three-letter code: ZN) (formula: Zn).

Mol	Chain	Residues	Atoms		AltConf
			Total	Zn	
4	A	1	1	1	0

CONFIDENTIAL VALIDATION REPORT

3 Residue-property plots ⓘ

These plots are drawn for all protein, RNA and DNA chains in the entry. The first graphic for a chain summarises the proportions of the various outlier classes displayed in the second graphic. The second graphic shows the sequence view annotated by issues in geometry. Residues are color-coded according to the number of geometric quality criteria for which they contain at least one outlier: green = 0, yellow = 1, orange = 2 and red = 3 or more. Stretches of 2 or more consecutive residues without any outlier are shown as a green connector. Residues present in the sample, but not in the model, are shown in grey.

• Molecule 1: Interferon-induced helicase C domain-containing protein 1

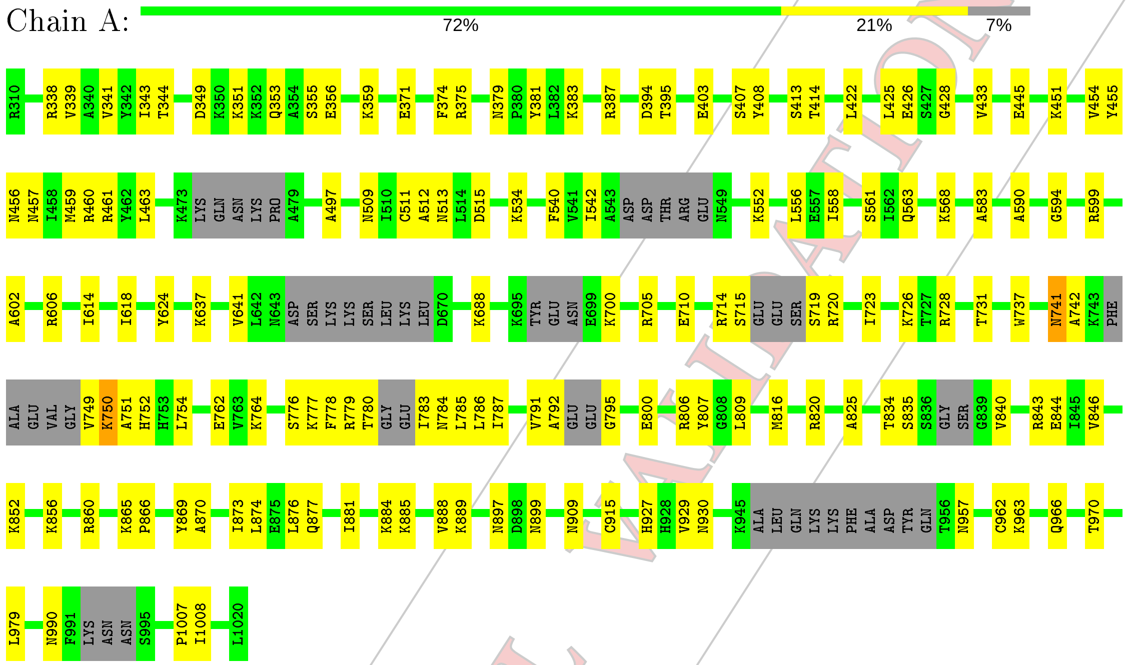

• Molecule 2: RNA (5'-R(P*UP*CP*CP*AP*UP*GP*CP*GP*CP*AP*UP*GP*AP*CP*G)-3')

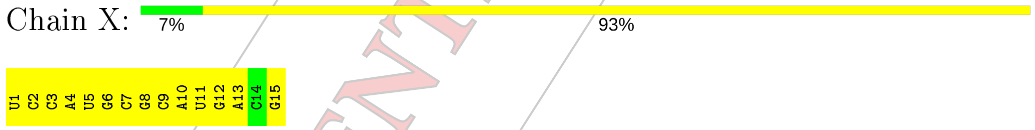

• Molecule 3: RNA (5'-R(P*CP*GP*UP*CP*AP*UP*GP*CP*GP*CP*AP*UP*GP*GP*A)-3')

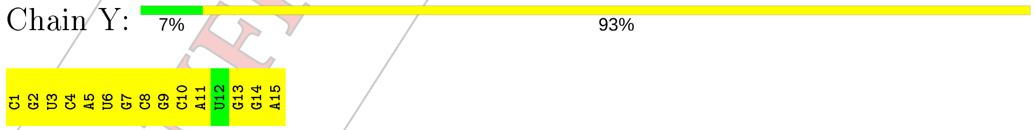

CONFIDENTIAL VALIDATION REPORT

4 Experimental information (i)

Property	Value	Source
Reconstruction method	HELICAL	Depositor
Imposed symmetry	HELICAL, twist=87.3689°, rise=44.5102 Å, axial sym=C1	Depositor
Number of segments used	60079	Depositor
Resolution determination method	FSC 0.143 CUT-OFF	Depositor
CTF correction method	PHASE FLIPPING AND AMPLITUDE CORRECTION	Depositor
Microscope	FEI TITAN KRIOS	Depositor
Voltage (kV)	300	Depositor
Electron dose ($e^-/\text{Å}^2$)	29.85	Depositor
Minimum defocus (nm)	1800	Depositor
Maximum defocus (nm)	2700	Depositor
Magnification	75000	Depositor
Image detector	FEI FALCON III (4k x 4k)	Depositor

CONFIDENTIAL VALIDATION REPORT

5 Model quality ⓘ

5.1 Standard geometry ⓘ

Bond lengths and bond angles in the following residue types are not validated in this section: ZN

The Z score for a bond length (or angle) is the number of standard deviations the observed value is removed from the expected value. A bond length (or angle) with $|Z| > 5$ is considered an outlier worth inspection. RMSZ is the root-mean-square of all Z scores of the bond lengths (or angles).

Mol	Chain	Bond lengths		Bond angles	
		RMSZ	$\#|Z| >2$	RMSZ	$\#|Z| >2$
1	A	0.39	0/5231	0.57	1/7025 (0.0%)
2	X	0.57	0/354	0.87	0/549
3	Y	0.58	0/358	0.85	0/556
All	All	0.42	0/5943	0.62	1/8130 (0.0%)

Chiral center outliers are detected by calculating the chiral volume of a chiral center and verifying if the center is modelled as a planar moiety or with the opposite hand.A planarity outlier is detected by checking planarity of atoms in a peptide group, atoms in a mainchain group or atoms of a sidechain that are expected to be planar.

Mol	Chain	#Chirality outliers	#Planarity outliers
1	A	0	3

There are no bond length outliers.

All (1) bond angle outliers are listed below:

Mol	Chain	Res	Type	Atoms	Z	Observed(^{o})	Ideal(^{o})
1	A	497	ALA	C-N-CA	9.22	144.74	121.70

There are no chirality outliers.

All (3) planarity outliers are listed below:

Mol	Chain	Res	Type	Group
1	A	428	GLY	Peptide
1	A	454	VAL	Peptide
1	A	762	GLU	Peptide

CONFIDENTIAL VALIDATION REPORT

5.2 Too-close contacts (i)

In the following table, the Non-H and H(model) columns list the number of non-hydrogen atoms and hydrogen atoms in the chain respectively. The H(added) column lists the number of hydrogen atoms added and optimized by MolProbity. The Clashes column lists the number of clashes within the asymmetric unit, whereas Symm-Clashes lists symmetry related clashes.

Mol	Chain	Non-H	H(model)	H(added)	Clashes	Symm-Clashes
1	A	5155	4096	5272	102	0
2	X	318	0	163	21	0
3	Y	321	0	163	22	0
4	A	1	0	0	0	0
All	All	5795	4096	5598	139	0

The all-atom clashscore is defined as the number of clashes found per 1000 atoms (including hydrogen atoms). The all-atom clashscore for this structure is 12.

All (139) close contacts within the same asymmetric unit are listed below, sorted by their clash magnitude.

Atom-1	Atom-2	Interatomic distance (Å)	Clash overlap (Å)
1:A:777:LYS:O	1:A:780:THR:OG1	2.02	0.78
1:A:749:VAL:HA	1:A:784:ASN:HD22	1.51	0.75
1:A:515:ASP:OD1	1:A:877:GLN:NE2	2.19	0.74
1:A:791:VAL:HG23	3:Y:7:G:H5”	1.72	0.71
1:A:599:ARG:NH1	1:A:909:ASN:OD1	2.22	0.71
1:A:806:ARG:HD2	1:A:809:LEU:HD23	1.73	0.71
1:A:374:PHE:O	1:A:379:ASN:ND2	2.24	0.70
1:A:445:GLU:O	1:A:455:TYR:OH	2.09	0.69
1:A:715:SER:HG	1:A:719:SER:N	1.90	0.69
1:A:457:ASN:OD1	1:A:460:ARG:NH2	2.26	0.69
2:X:5:U:H2’	2:X:6:G:H8	1.59	0.68
3:Y:1:C:H2’	3:Y:2:G:C8	2.29	0.67
1:A:792:ALA:O	1:A:795:GLY:N	2.29	0.66
1:A:341:VAL:O	1:A:344:THR:OG1	2.15	0.65
3:Y:4:C:H2’	3:Y:5:A:C8	2.32	0.65
1:A:865:LYS:HD2	1:A:866:PRO:HD2	1.78	0.64
3:Y:3:U:H2’	3:Y:4:C:H6	1.62	0.64
1:A:351:LYS:O	1:A:355:SER:N	2.30	0.64
2:X:5:U:H2’	2:X:6:G:C8	2.32	0.64
1:A:590:ALA:HB3	1:A:599:ARG:HB3	1.80	0.64
1:A:909:ASN:ND2	1:A:966:GLN:OE1	2.31	0.64
3:Y:3:U:H2’	3:Y:4:C:C6	2.33	0.63
2:X:9:C:H2’	2:X:10:A:C8	2.34	0.62

Continued on next page...

CONFIDENTIAL VALIDATION REPORT

Continued from previous page...

Atom-1	Atom-2	Interatomic distance (Å)	Clash overlap (Å)
1:A:558:ILE:O	1:A:561:SER:OG	2.14	0.62
2:X:3:C:H2’	2:X:4:A:C8	2.35	0.62
1:A:511:CYS:O	1:A:515:ASP:N	2.33	0.62
3:Y:4:C:H2’	3:Y:5:A:H8	1.64	0.62
3:Y:2:G:O2’	3:Y:3:U:H5’	2.00	0.61
1:A:624:TYR:CZ	1:A:688:LYS:HB2	2.36	0.61
1:A:957:ASN:HB3	1:A:970:THR:CG2	2.31	0.61
1:A:728:ARG:O	1:A:731:THR:OG1	2.11	0.61
2:X:4:A:O2’	2:X:5:U:H5’	2.02	0.60
2:X:12:G:H2’	2:X:13:A:H8	1.67	0.60
1:A:371:GLU:OE2	1:A:375:ARG:NH2	2.34	0.60
1:A:927:HIS:NE2	2:X:5:U:O2’	2.29	0.59
1:A:413:SER:OG	1:A:414:THR:N	2.34	0.59
3:Y:1:C:H2’	3:Y:2:G:H8	1.68	0.58
1:A:962:CYS:SG	1:A:963:LYS:N	2.77	0.58
1:A:387:ARG:NH1	1:A:407:SER:OG	2.37	0.57
2:X:12:G:H2’	2:X:13:A:C8	2.39	0.57
1:A:816:MET:O	1:A:820:ARG:HG3	2.03	0.57
1:A:800:GLU:HG2	1:A:825:ALA:HB2	1.86	0.56
1:A:870:ALA:HA	1:A:873:ILE:HG12	1.85	0.56
1:A:752:HIS:CB	1:A:783:ILE:HG12	2.35	0.56
1:A:751:ALA:HB2	1:A:785:LEU:HD21	1.88	0.56
1:A:749:VAL:HG12	1:A:785:LEU:HD22	1.88	0.55
1:A:614:ILE:O	1:A:618:ILE:N	2.39	0.55
3:Y:10:C:O2’	3:Y:11:A:H5’	2.06	0.54
3:Y:14:G:H2’	3:Y:15:A:H8	1.72	0.54
2:X:3:C:H2’	2:X:4:A:H8	1.71	0.54
1:A:425:LEU:HB2	1:A:433:VAL:H	1.71	0.54
1:A:459:MET:O	1:A:463:LEU:HG	2.08	0.54
2:X:9:C:H2’	2:X:10:A:H8	1.72	0.54
3:Y:7:G:O2’	3:Y:8:C:H5’	2.08	0.53
1:A:637:LYS:O	1:A:641:VAL:HG23	2.09	0.53
2:X:2:C:H2’	2:X:3:C:H6	1.73	0.53
1:A:339:VAL:O	1:A:343:ILE:HD12	2.08	0.53
1:A:728:ARG:NH2	3:Y:7:G:OP2	2.41	0.53
1:A:731:THR:HG22	1:A:787:ILE:HG22	1.92	0.52
3:Y:5:A:O2’	3:Y:6:U:H5’	2.08	0.52
1:A:426:GLU:OE1	1:A:461:ARG:NH1	2.41	0.52
1:A:710:GLU:O	1:A:714:ARG:HG3	2.09	0.52
1:A:749:VAL:HA	1:A:784:ASN:ND2	2.23	0.52
1:A:349:ASP:O	1:A:353:GLN:HG2	2.10	0.52

Continued on next page...

CONFIDENTIAL VALIDATION REPORT

Continued from previous page...

Atom-1	Atom-2	Interatomic distance (Å)	Clash overlap (Å)
1:A:351:LYS:HD2	1:A:356:GLU:HB2	1.92	0.51
2:X:1:U:H2'	2:X:2:C:C6	2.45	0.51
1:A:754:LEU:N	1:A:787:ILE:O	2.44	0.51
1:A:899:ASN:HB2	1:A:1008:ILE:CG2	2.41	0.50
1:A:451:LYS:HB2	2:X:10:A:H5"	1.94	0.50
3:Y:3:U:O2'	3:Y:4:C:H5'	2.12	0.50
2:X:6:G:O2'	2:X:7:C:H5'	2.11	0.50
1:A:852:LYS:O	1:A:856:LYS:HG2	2.12	0.50
1:A:897:ASN:HD22	1:A:1007:PRO:CG	2.25	0.49
1:A:915:CYS:SG	1:A:930:ASN:ND2	2.83	0.49
1:A:929:VAL:HG21	1:A:979:LEU:HD22	1.95	0.49
1:A:752:HIS:HB2	1:A:783:ILE:HG12	1.95	0.49
2:X:2:C:H2'	2:X:3:C:C6	2.48	0.49
1:A:422:LEU:O	1:A:425:LEU:HB3	2.13	0.48
1:A:403:GLU:O	1:A:407:SER:N	2.46	0.48
2:X:7:C:H2'	2:X:8:G:C8	2.48	0.48
2:X:1:U:H2'	2:X:2:C:H6	1.77	0.48
3:Y:14:G:H2'	3:Y:15:A:C8	2.48	0.48
3:Y:6:U:H2'	3:Y:7:G:C8	2.48	0.48
1:A:752:HIS:HB3	1:A:783:ILE:HG12	1.95	0.48
1:A:540:PHE:HE1	1:A:844:GLU:HG2	1.79	0.48
1:A:563:GLN:NE2	1:A:568:LYS:O	2.46	0.48
1:A:776:SER:O	1:A:779:ARG:HB2	2.14	0.47
1:A:843:ARG:HA	1:A:846:VAL:HG12	1.96	0.47
1:A:425:LEU:HD22	1:A:433:VAL:C	2.35	0.47
1:A:750:LYS:HD2	1:A:784:ASN:ND2	2.29	0.47
1:A:509:ASN:HD21	1:A:876:LEU:HD22	1.80	0.47
1:A:720:ARG:NH1	1:A:778:PHE:O	2.43	0.47
1:A:512:ALA:HB1	1:A:877:GLN:HG3	1.96	0.47
2:X:8:G:O2'	2:X:9:C:H5'	2.14	0.47
2:X:10:A:O2'	2:X:11:U:H5'	2.15	0.47
1:A:873:ILE:O	1:A:877:GLN:HG3	2.14	0.46
2:X:2:C:O2'	2:X:3:C:H5'	2.16	0.46
1:A:542:ILE:HG21	1:A:835:SER:HB3	1.96	0.46
1:A:540:PHE:CE1	1:A:844:GLU:HG2	2.50	0.46
1:A:834:THR:HG21	1:A:840:VAL:HG21	1.96	0.46
1:A:962:CYS:HB3	1:A:966:GLN:H	1.81	0.46
3:Y:6:U:H2'	3:Y:7:G:H8	1.81	0.45
1:A:425:LEU:HD22	1:A:433:VAL:O	2.17	0.45
1:A:359:LYS:NZ	1:A:407:SER:O	2.50	0.44
1:A:542:ILE:CG2	1:A:835:SER:HB3	2.47	0.44

Continued on next page...

CONFIDENTIAL VALIDATION REPORT

Continued from previous page...

Atom-1	Atom-2	Interatomic distance (Å)	Clash overlap (Å)
1:A:869:TYR:O	1:A:873:ILE:HG23	2.17	0.44
1:A:407:SER:OG	1:A:408:TYR:N	2.50	0.44
1:A:509:ASN:ND2	1:A:876:LEU:HD22	2.31	0.44
1:A:705:ARG:HD3	1:A:737:TRP:CH2	2.52	0.44
3:Y:13:G:O2'	3:Y:14:G:H5'	2.18	0.44
3:Y:8:C:H2'	3:Y:9:G:C8	2.53	0.44
1:A:929:VAL:HG21	1:A:979:LEU:HB3	2.00	0.44
1:A:778:PHE:CG	1:A:786:LEU:HD11	2.54	0.43
1:A:777:LYS:HB3	1:A:783:ILE:HD12	2.01	0.43
3:Y:14:G:O2'	3:Y:15:A:H5'	2.19	0.43
1:A:552:LYS:HG2	1:A:556:LEU:HD13	2.00	0.43
1:A:897:ASN:HD22	1:A:1007:PRO:HG2	1.83	0.43
1:A:590:ALA:O	1:A:594:GLY:N	2.52	0.43
1:A:338:ARG:NH1	1:A:381:TYR:OH	2.52	0.42
1:A:741:ASN:HD22	1:A:742:ALA:H	1.67	0.42
1:A:856:LYS:O	1:A:860:ARG:HG3	2.19	0.42
1:A:884:LYS:O	1:A:888:VAL:HG23	2.18	0.42
1:A:509:ASN:O	1:A:513:ASN:ND2	2.53	0.42
1:A:749:VAL:CG1	1:A:785:LEU:HD22	2.48	0.42
1:A:723:ILE:HD12	1:A:787:ILE:HG12	2.00	0.42
1:A:791:VAL:HG22	3:Y:7:G:O3'	2.20	0.42
1:A:379:ASN:O	1:A:383:LYS:N	2.45	0.42
1:A:463:LEU:HB3	1:A:881:ILE:HD11	2.02	0.42
2:X:7:C:H2'	2:X:8:G:H8	1.85	0.42
1:A:583:ALA:O	1:A:602:ALA:HB1	2.19	0.41
1:A:700:LYS:HB3	1:A:807:TYR:HE1	1.84	0.41
1:A:726:LYS:O	3:Y:6:U:H4'	2.21	0.41
1:A:885:LYS:O	1:A:889:LYS:HG2	2.20	0.41
1:A:751:ALA:HA	1:A:783:ILE:HG23	2.02	0.41
1:A:870:ALA:O	1:A:874:LEU:HG	2.21	0.41
1:A:750:LYS:O	1:A:785:LEU:HD23	2.21	0.41
1:A:764:LYS:O	1:A:764:LYS:HD3	2.20	0.40
1:A:899:ASN:H	1:A:1008:ILE:HA	1.86	0.40
1:A:394:ASP:OD1	1:A:395:THR:N	2.55	0.40

There are no symmetry-related clashes.

CONFIDENTIAL VALIDATION REPORT

5.3 Torsion angles (i)

5.3.1 Protein backbone (i)

In the following table, the Percentiles column shows the percent Ramachandran outliers of the chain as a percentile score with respect to all PDB entries followed by that with respect to all EM entries.

The Analysed column shows the number of residues for which the backbone conformation was analysed, and the total number of residues.

Mol	Chain	Analysed	Favoured	Allowed	Outliers	Percentiles	
1	A	621/693 (90%)	542 (87%)	79 (13%)	0	100	100

There are no Ramachandran outliers to report.

5.3.2 Protein sidechains (i)

In the following table, the Percentiles column shows the percent sidechain outliers of the chain as a percentile score with respect to all PDB entries followed by that with respect to all EM entries.

The Analysed column shows the number of residues for which the sidechain conformation was analysed, and the total number of residues.

Mol	Chain	Analysed	Rotameric	Outliers	Percentiles	
1	A	571/622 (92%)	565 (99%)	6 (1%)	76	88

All (6) residues with a non-rotameric sidechain are listed below:

Mol	Chain	Res	Type
1	A	456	ASN
1	A	534	LYS
1	A	606	ARG
1	A	741	ASN
1	A	750	LYS
1	A	990	ASN

Some sidechains can be flipped to improve hydrogen bonding and reduce clashes. All (7) such sidechains are listed below:

Mol	Chain	Res	Type
1	A	513	ASN
1	A	683	ASN

Continued on next page...

CONFIDENTIAL VALIDATION REPORT

Continued from previous page...

Mol	Chain	Res	Type
1	A	741	ASN
1	A	784	ASN
1	A	897	ASN
1	A	899	ASN
1	A	990	ASN

5.3.3 RNA (i)

Mol	Chain	Analysed	Backbone Outliers	Pucker Outliers
2	X	14/15 (93%)	1 (7%)	0
3	Y	14/15 (93%)	0	0
All	All	28/30 (93%)	1 (3%)	0

All (1) RNA backbone outliers are listed below:

Mol	Chain	Res	Type
2	X	15	G

There are no RNA pucker outliers to report.

5.4 Non-standard residues in protein, DNA, RNA chains (i)

There are no non-standard protein/DNA/RNA residues in this entry.

5.5 Carbohydrates (i)

There are no carbohydrates in this entry.

5.6 Ligand geometry (i)

Of 1 ligands modelled in this entry, 1 is monoatomic - leaving 0 for Mogul analysis.

There are no bond length outliers.

There are no bond angle outliers.

There are no chirality outliers.

There are no torsion outliers.

There are no ring outliers.

CONFIDENTIAL VALIDATION REPORT

No monomer is involved in short contacts.

5.7 Other polymers (i)

There are no such residues in this entry.

5.8 Polymer linkage issues (i)

There are no chain breaks in this entry.

CONFIDENTIAL VALIDATION REPORT

Click here to access/download;PDB Validation Report;D_1200009295_val-report-full-annotate_P1.pdf

Full wwPDB/EMDataBank EM Map/Model Validation Report (i)

Apr 4, 2018 – 10:25 AM BST

PDB ID	:	6G1X
EMDB ID:	:	EMD-4341
Title	:	CryoEM structure of the MDA5-dsRNA filament with 91-degree helical twist
Deposited on	:	2018-03-22
Resolution	:	3.93 Å(reported)

CONFIDENTIAL VALIDATION REPORT

This is a Full wwPDB/EMDataBank EM Map/Model Validation Report.

This report is produced by the wwPDB biocuration pipeline after annotation of the structure.

We welcome your comments at *validation@mail.wwpdb.org*
A user guide is available at
https://www.wwpdb.org/validation/2017/EMValidationReportHelp
with specific help available everywhere you see the (i) symbol.

MolProbity	:	4.02b-467
Percentile statistics	:	20171227.v01 (using entries in the PDB archive December 27th 2017)
Ideal geometry (proteins)	:	Engh & Huber (2001)
Ideal geometry (DNA, RNA)	:	Parkinson et. al. (1996)
Validation Pipeline (wwPDB-VP)	:	rb-20031021

1 Overall quality at a glance (i)

The following experimental techniques were used to determine the structure:
ELECTRON MICROSCOPY

The reported resolution of this entry is 3.93 Å.

Percentile scores (ranging between 0-100) for global validation metrics of the entry are shown in the following graphic. The table shows the number of entries on which the scores are based.

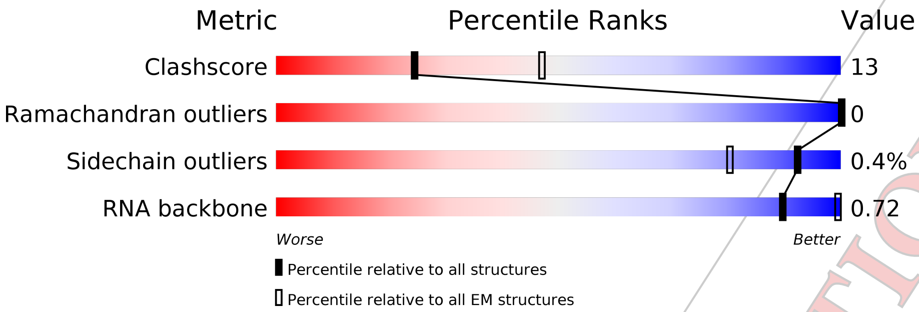

Metric	Whole archive (#Entries)	EM structures (#Entries)
Clashscore	136327	1886
Ramachandran outliers	132723	1663
Sidechain outliers	132532	1531
RNA backbone	3747	458

The table below summarises the geometric issues observed across the polymeric chains. The red, orange, yellow and green segments on the bar indicate the fraction of residues that contain outliers for >=3, 2, 1 and 0 types of geometric quality criteria. A grey segment represents the fraction of residues that are not modelled. The numeric value for each fraction is indicated below the corresponding segment, with a dot representing fractions <=5%

Mol	Chain	Length	Quality of chain
1	A	696	71% 22% • 7%
2	X	15	93% 7%
3	Y	15	100%

CONFIDENTIAL VALIDATION REPORT

2 Entry composition (i)

There are 4 unique types of molecules in this entry. The entry contains 9989 atoms, of which 4182 are hydrogens and 0 are deuteriums.

In the tables below, the AltConf column contains the number of residues with at least one atom in alternate conformation and the Trace column contains the number of residues modelled with at most 2 atoms.

- Molecule 1 is a protein called Interferon-induced helicase C domain-containing protein 1.

Mol	Chain	Residues	Atoms						AltConf	Trace
			Total	C	H	N	O	S		
1	A	648	9349	3287	4182	896	949	35	0	0

There are 18 discrepancies between the modelled and reference sequences:

Chain	Residue	Modelled	Actual	Comment	Reference
A	?	-	ASP	deletion	UNP Q8R5F7
A	?	-	LYS	deletion	UNP Q8R5F7
A	?	-	SER	deletion	UNP Q8R5F7
A	?	-	ASP	deletion	UNP Q8R5F7
A	?	-	ASP	deletion	UNP Q8R5F7
A	?	-	GLU	deletion	UNP Q8R5F7
A	?	-	ALA	deletion	UNP Q8R5F7
A	?	-	SER	deletion	UNP Q8R5F7
A	?	-	SER	deletion	UNP Q8R5F7
A	?	-	CYS	deletion	UNP Q8R5F7
A	?	-	ASN	deletion	UNP Q8R5F7
A	?	-	ASP	deletion	UNP Q8R5F7
A	?	-	GLN	deletion	UNP Q8R5F7
A	?	-	LEU	deletion	UNP Q8R5F7
A	?	-	LYS	deletion	UNP Q8R5F7
A	?	-	GLY	deletion	UNP Q8R5F7
A	?	-	ASP	deletion	UNP Q8R5F7
A	?	-	VAL	deletion	UNP Q8R5F7

- Molecule 2 is a RNA chain called RNA (5'-R(P*UP*CP*CP*AP*UP*GP*CP*GP*CP*AP *UP*GP*AP*CP*G)-3').

Mol	Chain	Residues	Atoms					AltConf	Trace
			Total	C	N	O	P		
2	X	15	318	142	56	105	15	0	0

- Molecule 3 is a RNA chain called RNA (5'-R(P*CP*GP*UP*CP*AP*UP*GP*CP*GP*CP

CONFIDENTIAL VALIDATION REPORT

*AP*UP*GP*GP*A)-3').

Mol	Chain	Residues	Atoms					AltConf	Trace
			Total	C	N	O	P		
3	Y	15	321	143	58	105	15	0	0

- Molecule 4 is ZINC ION (three-letter code: ZN) (formula: Zn).

Mol	Chain	Residues	Atoms		AltConf
			Total	Zn	
4	A	1	1	1	0

CONFIDENTIAL VALIDATION REPORT

3 Residue-property plots

These plots are drawn for all protein, RNA and DNA chains in the entry. The first graphic for a chain summarises the proportions of the various outlier classes displayed in the second graphic. The second graphic shows the sequence view annotated by issues in geometry. Residues are color-coded according to the number of geometric quality criteria for which they contain at least one outlier: green = 0, yellow = 1, orange = 2 and red = 3 or more. Stretches of 2 or more consecutive residues without any outlier are shown as a green connector. Residues present in the sample, but not in the model, are shown in grey.

- Molecule 1: Interferon-induced helicase C domain-containing protein 1

Chain A:

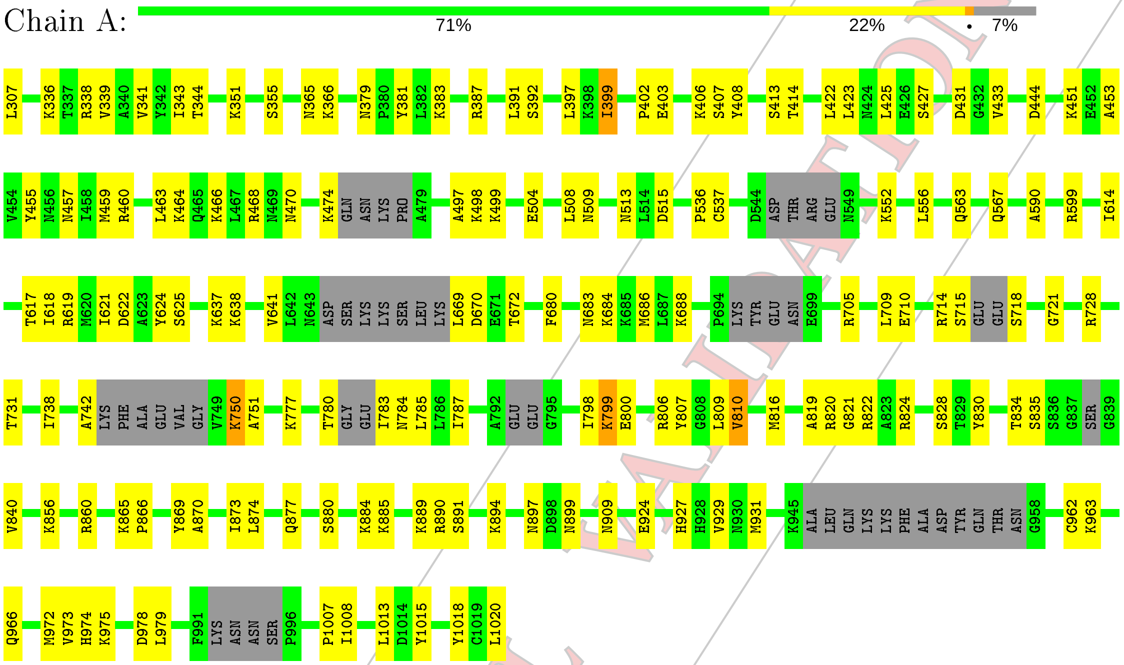

- Molecule 2: RNA (5'-R(P*UP*CP*CP*AP*UP*GP*CP*GP*CP*AP*UP*GP*AP*CP*G)-3')

Chain X:

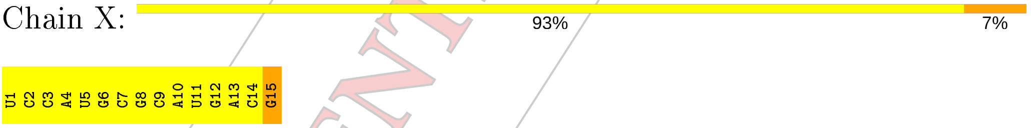

- Molecule 3: RNA (5'-R(P*CP*GP*UP*CP*AP*UP*GP*CP*GP*CP*AP*UP*GP*GP*A)-3')

Chain Y:

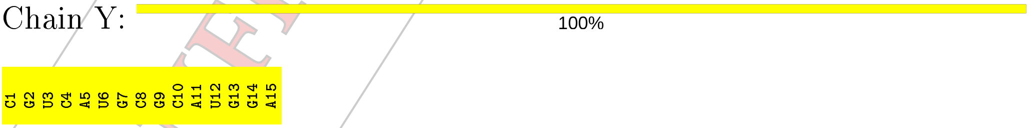

CONFIDENTIAL VALIDATION REPORT

4 Experimental information (i)

Property	Value	Source
Reconstruction method	HELICAL	Depositor
Imposed symmetry	HELICAL, twist=90.9214°, rise=44.9703 Å, axial sym=C1	Depositor
Number of segments used	39987	Depositor
Resolution determination method	FSC 0.143 CUT-OFF	Depositor
CTF correction method	PHASE FLIPPING AND AMPLITUDE CORRECTION	Depositor
Microscope	FEI TITAN KRIOS	Depositor
Voltage (kV)	300	Depositor
Electron dose ($e^-/\text{Å}^2$)	29.85	Depositor
Minimum defocus (nm)	1800	Depositor
Maximum defocus (nm)	2700	Depositor
Magnification	75000	Depositor
Image detector	FEI FALCON III (4k x 4k)	Depositor

CONFIDENTIAL VALIDATION REPORT

5 Model quality (i)

5.1 Standard geometry (i)

Bond lengths and bond angles in the following residue types are not validated in this section: ZN

The Z score for a bond length (or angle) is the number of standard deviations the observed value is removed from the expected value. A bond length (or angle) with $|Z| > 5$ is considered an outlier worth inspection. RMSZ is the root-mean-square of all Z scores of the bond lengths (or angles).

Mol	Chain	Bond lengths		Bond angles	
		RMSZ	#$|Z|$ >2	RMSZ	#$|Z|$ >2
1	A	0.55	0/5242	0.62	2/7039 (0.0%)
2	X	0.95	0/354	0.99	0/549
3	Y	0.94	0/358	0.88	0/556
All	All	0.61	0/5954	0.67	2/8144 (0.0%)

Chiral center outliers are detected by calculating the chiral volume of a chiral center and verifying if the center is modelled as a planar moiety or with the opposite hand.A planarity outlier is detected by checking planarity of atoms in a peptide group, atoms in a mainchain group or atoms of a sidechain that are expected to be planar.

Mol	Chain	#Chirality outliers	#Planarity outliers
1	A	0	1

There are no bond length outliers.

All (2) bond angle outliers are listed below:

Mol	Chain	Res	Type	Atoms	Z	Observed(^{o})	Ideal(^{o})
1	A	497	ALA	C-N-CA	6.12	137.00	121.70
1	A	399	ILE	C-N-CA	5.75	136.06	121.70

There are no chirality outliers.

All (1) planarity outliers are listed below:

Mol	Chain	Res	Type	Group
1	A	810	VAL	Peptide

CONFIDENTIAL VALIDATION REPORT

5.2 Too-close contacts (i)

In the following table, the Non-H and H(model) columns list the number of non-hydrogen atoms and hydrogen atoms in the chain respectively. The H(added) column lists the number of hydrogen atoms added and optimized by MolProbity. The Clashes column lists the number of clashes within the asymmetric unit, whereas Symm-Clashes lists symmetry related clashes.

Mol	Chain	Non-H	H(model)	H(added)	Clashes	Symm-Clashes
1	A	5167	4182	5287	107	0
2	X	318	0	163	23	0
3	Y	321	0	163	22	0
4	A	1	0	0	0	0
All	All	5807	4182	5613	146	0

The all-atom clashscore is defined as the number of clashes found per 1000 atoms (including hydrogen atoms). The all-atom clashscore for this structure is 13.

All (146) close contacts within the same asymmetric unit are listed below, sorted by their clash magnitude.

Atom-1	Atom-2	Interatomic distance (Å)	Clash overlap (Å)
2:X:3:C:H2'	2:X:4:A:H8	1.42	0.83
1:A:444:ASP:O	1:A:455:TYR:OH	2.01	0.78
1:A:599:ARG:NH1	1:A:909:ASN:OD1	2.18	0.77
1:A:777:LYS:O	1:A:780:THR:OG1	2.05	0.74
1:A:909:ASN:ND2	1:A:966:GLN:OE1	2.20	0.73
1:A:974:HIS:HB3	1:A:975:LYS:HD2	1.71	0.73
1:A:821:GLY:O	1:A:824:ARG:NE	2.17	0.72
1:A:351:LYS:O	1:A:355:SER:N	2.22	0.72
2:X:9:C:H2'	2:X:10:A:H8	1.54	0.71
1:A:423:LEU:O	1:A:427:SER:N	2.20	0.70
2:X:9:C:H2'	2:X:10:A:C8	2.27	0.69
1:A:715:SER:O	1:A:718:SER:N	2.24	0.69
2:X:5:U:H2'	2:X:6:G:H8	1.57	0.69
1:A:341:VAL:O	1:A:344:THR:OG1	2.11	0.68
3:Y:1:C:H2'	3:Y:2:G:H8	1.58	0.67
1:A:751:ALA:HB2	1:A:785:LEU:HD21	1.76	0.67
1:A:810:VAL:HG21	2:X:13:A:H5'	1.77	0.67
2:X:3:C:H2'	2:X:4:A:C8	2.29	0.67
1:A:824:ARG:O	1:A:828:SER:OG	2.14	0.66
3:Y:1:C:H2'	3:Y:2:G:C8	2.30	0.66
1:A:891:SER:HA	1:A:894:LYS:HG2	1.76	0.66
3:Y:3:U:H2'	3:Y:4:C:H6	1.61	0.65
2:X:5:U:H2'	2:X:6:G:C8	2.32	0.65

Continued on next page...

CONFIDENTIAL VALIDATION REPORT

Continued from previous page...

Atom-1	Atom-2	Interatomic distance (Å)	Clash overlap (Å)
3:Y:4:C:H2’	3:Y:5:A:C8	2.31	0.65
1:A:336:LYS:NZ	1:A:444:ASP:OD1	2.30	0.64
3:Y:8:C:H2’	3:Y:9:G:H8	1.62	0.64
3:Y:4:C:H2’	3:Y:5:A:H8	1.63	0.64
1:A:927:HIS:NE2	2:X:5:U:O2’	2.28	0.63
1:A:614:ILE:O	1:A:618:ILE:N	2.30	0.63
1:A:617:THR:O	1:A:807:TYR:OH	2.16	0.63
1:A:806:ARG:HD2	1:A:809:LEU:CD2	2.29	0.62
1:A:897:ASN:HB2	1:A:1007:PRO:HG2	1.80	0.62
1:A:453:ALA:HB2	2:X:10:A:H5’	1.81	0.62
3:Y:10:C:O2’	3:Y:11:A:H5’	2.00	0.62
1:A:379:ASN:O	1:A:383:LYS:N	2.32	0.62
1:A:464:LYS:HE2	1:A:468:ARG:HH21	1.66	0.61
1:A:834:THR:HG21	1:A:840:VAL:HG21	1.82	0.61
1:A:865:LYS:HD2	1:A:866:PRO:HD2	1.83	0.61
1:A:425:LEU:HB2	1:A:433:VAL:H	1.66	0.60
1:A:339:VAL:O	1:A:343:ILE:HD12	2.00	0.60
3:Y:8:C:H2’	3:Y:9:G:C8	2.37	0.60
1:A:806:ARG:HD2	1:A:809:LEU:HD23	1.83	0.59
3:Y:3:U:H2’	3:Y:4:C:C6	2.38	0.59
1:A:590:ALA:HB3	1:A:599:ARG:HB3	1.85	0.59
1:A:619:ARG:HG3	1:A:621:ILE:HG22	1.85	0.59
2:X:8:G:O2’	2:X:9:C:H5’	2.03	0.58
2:X:7:C:H2’	2:X:8:G:H8	1.68	0.58
2:X:12:G:H2’	2:X:13:A:H8	1.70	0.57
1:A:451:LYS:HD2	1:A:880:SER:HA	1.87	0.57
1:A:683:ASN:O	1:A:686:MET:HG2	2.05	0.56
1:A:307:LEU:HB3	1:A:381:TYR:CD2	2.40	0.56
1:A:402:PRO:HG2	1:A:431:ASP:HB3	1.88	0.56
3:Y:7:G:O2’	3:Y:8:C:H5’	2.06	0.56
2:X:7:C:H2’	2:X:8:G:C8	2.40	0.55
2:X:12:G:H2’	2:X:13:A:C8	2.42	0.55
1:A:798:ILE:HB	1:A:822:ARG:HD2	1.87	0.55
1:A:890:ARG:O	1:A:894:LYS:HG2	2.07	0.55
1:A:459:MET:O	1:A:463:LEU:HG	2.07	0.55
1:A:1018:TYR:O	1:A:1020:LEU:HD12	2.07	0.55
1:A:891:SER:HA	1:A:894:LYS:CG	2.37	0.55
1:A:710:GLU:O	1:A:714:ARG:HG3	2.06	0.55
1:A:413:SER:OG	1:A:414:THR:N	2.40	0.54
2:X:4:A:O2’	2:X:5:U:H5’	2.07	0.54
1:A:536:PRO:HB2	1:A:828:SER:HB2	1.88	0.54

Continued on next page...

CONFIDENTIAL VALIDATION REPORT

Continued from previous page...

Atom-1	Atom-2	Interatomic distance (Å)	Clash overlap (Å)
1:A:515:ASP:OD1	1:A:877:GLN:NE2	2.40	0.54
1:A:622:ASP:O	1:A:625:SER:OG	2.21	0.53
3:Y:14:G:H2'	3:Y:15:A:H8	1.71	0.53
1:A:962:CYS:HB3	1:A:966:GLN:N	2.23	0.53
1:A:728:ARG:NH2	3:Y:7:G:OP2	2.41	0.53
1:A:975:LYS:HG2	1:A:1015:TYR:OH	2.08	0.53
1:A:509:ASN:O	1:A:513:ASN:ND2	2.42	0.53
1:A:624:TYR:CE2	1:A:688:LYS:HD2	2.44	0.53
1:A:806:ARG:HG3	1:A:830:TYR:OH	2.09	0.53
1:A:399:ILE:HD11	1:A:403:GLU:HG3	1.91	0.52
3:Y:3:U:O2'	3:Y:4:C:H5'	2.10	0.52
1:A:552:LYS:HG2	1:A:556:LEU:CD1	2.40	0.52
1:A:391:LEU:O	1:A:392:SER:OG	2.25	0.51
1:A:731:THR:HG22	1:A:787:ILE:HG22	1.91	0.51
2:X:6:G:O2'	2:X:7:C:H5'	2.10	0.51
1:A:637:LYS:O	1:A:641:VAL:HG23	2.11	0.51
1:A:422:LEU:O	1:A:425:LEU:HB3	2.11	0.51
1:A:738:ILE:HG23	1:A:742:ALA:HB3	1.93	0.50
1:A:924:GLU:OE1	3:Y:12:U:O2'	2.22	0.50
3:Y:14:G:H2'	3:Y:15:A:C8	2.47	0.50
1:A:929:VAL:HG21	1:A:979:LEU:HB3	1.93	0.50
1:A:563:GLN:O	1:A:567:GLN:N	2.44	0.50
1:A:870:ALA:O	1:A:874:LEU:HG	2.12	0.49
1:A:962:CYS:SG	1:A:963:LYS:N	2.85	0.49
2:X:1:U:H2'	2:X:2:C:H6	1.77	0.49
3:Y:6:U:H2'	3:Y:7:G:C8	2.48	0.49
1:A:721:GLY:N	1:A:784:ASN:O	2.46	0.48
3:Y:14:G:O2'	3:Y:15:A:H5'	2.13	0.48
3:Y:2:G:O2'	3:Y:3:U:H5'	2.14	0.48
3:Y:6:U:H2'	3:Y:7:G:H8	1.78	0.48
1:A:464:LYS:HE2	1:A:468:ARG:NH2	2.29	0.48
1:A:466:LYS:NZ	1:A:515:ASP:OD2	2.35	0.47
1:A:619:ARG:CG	1:A:621:ILE:HG22	2.45	0.47
1:A:624:TYR:CZ	1:A:688:LYS:HD2	2.50	0.47
3:Y:13:G:O2'	3:Y:14:G:H5'	2.14	0.47
1:A:457:ASN:OD1	1:A:460:ARG:NH2	2.48	0.47
1:A:670:ASP:CG	1:A:672:THR:HG1	2.16	0.47
1:A:810:VAL:HG21	2:X:12:G:O2'	2.15	0.47
2:X:3:C:O2'	2:X:4:A:H5'	2.15	0.47
1:A:972:MET:HG2	1:A:974:HIS:HD2	1.81	0.46
2:X:1:U:H2'	2:X:2:C:C6	2.50	0.46

Continued on next page...

CONFIDENTIAL VALIDATION REPORT

Continued from previous page...

Atom-1	Atom-2	Interatomic distance (Å)	Clash overlap (Å)
1:A:470:ASN:O	1:A:474:LYS:N	2.44	0.46
2:X:11:U:H2'	2:X:12:G:C8	2.50	0.46
1:A:504:GLU:O	1:A:508:LEU:HD23	2.16	0.46
3:Y:9:G:H2'	3:Y:10:C:H6	1.79	0.46
1:A:387:ARG:NH1	1:A:407:SER:OG	2.48	0.46
1:A:799:LYS:HD2	1:A:800:GLU:HB2	1.97	0.46
1:A:537:CYS:O	1:A:820:ARG:NH2	2.49	0.45
2:X:14:C:H2'	2:X:15:G:C8	2.50	0.45
1:A:705:ARG:HG2	1:A:709:LEU:HD12	1.97	0.45
3:Y:5:A:O2'	3:Y:6:U:H5'	2.15	0.45
1:A:391:LEU:HD13	1:A:397:LEU:HD22	1.99	0.45
1:A:365:ASN:OD1	1:A:366:LYS:N	2.50	0.45
1:A:816:MET:O	1:A:820:ARG:HG2	2.17	0.45
1:A:973:VAL:HG22	1:A:978:ASP:OD1	2.17	0.45
1:A:425:LEU:HD22	1:A:433:VAL:O	2.17	0.44
1:A:777:LYS:CB	1:A:783:ILE:HD12	2.48	0.44
1:A:899:ASN:H	1:A:1008:ILE:HA	1.81	0.44
1:A:806:ARG:HD2	1:A:809:LEU:HD22	1.99	0.44
1:A:931:MET:CE	1:A:1013:LEU:HD11	2.48	0.44
1:A:728:ARG:O	1:A:731:THR:OG1	2.21	0.43
1:A:856:LYS:O	1:A:860:ARG:HG3	2.19	0.43
1:A:869:TYR:O	1:A:873:ILE:HG12	2.19	0.43
1:A:403:GLU:O	1:A:407:SER:N	2.52	0.42
1:A:806:ARG:HH21	1:A:819:ALA:CB	2.32	0.42
1:A:638:LYS:HE2	1:A:669:LEU:HD23	2.01	0.42
1:A:750:LYS:HD2	1:A:750:LYS:H	1.85	0.42
1:A:806:ARG:HH22	1:A:816:MET:HA	1.84	0.42
2:X:10:A:O2'	2:X:11:U:H5'	2.19	0.42
1:A:407:SER:OG	1:A:408:TYR:N	2.53	0.42
1:A:498:LYS:HG3	1:A:499:LYS:HG3	2.02	0.42
1:A:338:ARG:HD2	1:A:381:TYR:HE2	1.85	0.41
1:A:899:ASN:HB2	1:A:1008:ILE:CG2	2.50	0.41
1:A:873:ILE:O	1:A:877:GLN:HG3	2.19	0.41
1:A:460:ARG:HE	1:A:884:LYS:HD2	1.84	0.41
1:A:834:THR:HG22	1:A:835:SER:O	2.21	0.41
1:A:785:LEU:HD23	1:A:785:LEU:H	1.85	0.41
1:A:402:PRO:O	1:A:406:LYS:HG3	2.21	0.40
1:A:751:ALA:CB	1:A:785:LEU:HD21	2.46	0.40
1:A:777:LYS:HB3	1:A:783:ILE:HD12	2.03	0.40
1:A:885:LYS:O	1:A:889:LYS:HG2	2.21	0.40
1:A:680:PHE:HE2	1:A:684:LYS:HD3	1.87	0.40

CONFIDENTIAL VALIDATION REPORT

There are no symmetry-related clashes.

5.3 Torsion angles (i)

5.3.1 Protein backbone (i)

In the following table, the Percentiles column shows the percent Ramachandran outliers of the chain as a percentile score with respect to all PDB entries followed by that with respect to all EM entries.

The Analysed column shows the number of residues for which the backbone conformation was analysed, and the total number of residues.

Mol	Chain	Analysed	Favoured	Allowed	Outliers	Percentiles	
1	A	624/696 (90%)	545 (87%)	79 (13%)	0	100	100

There are no Ramachandran outliers to report.

5.3.2 Protein sidechains (i)

In the following table, the Percentiles column shows the percent sidechain outliers of the chain as a percentile score with respect to all PDB entries followed by that with respect to all EM entries.

The Analysed column shows the number of residues for which the sidechain conformation was analysed, and the total number of residues.

Mol	Chain	Analysed	Rotameric	Outliers	Percentiles	
1	A	571/625 (91%)	569 (100%)	2 (0%)	92	96

All (2) residues with a non-rotameric sidechain are listed below:

Mol	Chain	Res	Type
1	A	750	LYS
1	A	799	LYS

Some sidechains can be flipped to improve hydrogen bonding and reduce clashes. All (5) such sidechains are listed below:

Mol	Chain	Res	Type
1	A	379	ASN
1	A	513	ASN
1	A	711	GLN

Continued on next page...

CONFIDENTIAL VALIDATION REPORT

Continued from previous page...

Mol	Chain	Res	Type
1	A	859	ASN
1	A	974	HIS

5.3.3 RNA (i)

Mol	Chain	Analysed	Backbone Outliers	Pucker Outliers
2	X	14/15 (93%)	1 (7%)	0
3	Y	14/15 (93%)	0	0
All	All	28/30 (93%)	1 (3%)	0

All (1) RNA backbone outliers are listed below:

Mol	Chain	Res	Type
2	X	15	G

There are no RNA pucker outliers to report.

5.4 Non-standard residues in protein, DNA, RNA chains (i)

There are no non-standard protein/DNA/RNA residues in this entry.

5.5 Carbohydrates (i)

There are no carbohydrates in this entry.

5.6 Ligand geometry (i)

Of 1 ligands modelled in this entry, 1 is monoatomic - leaving 0 for Mogul analysis.

There are no bond length outliers.

There are no bond angle outliers.

There are no chirality outliers.

There are no torsion outliers.

There are no ring outliers.

No monomer is involved in short contacts.

CONFIDENTIAL VALIDATION REPORT

5.7 Other polymers (i)

There are no such residues in this entry.

5.8 Polymer linkage issues (i)

There are no chain breaks in this entry.

CONFIDENTIAL VALIDATION REPORT

Click here to access/download;PDB Validation Report;D_1200010087_val-report-full-annotate_P1.pdf

Full wwPDB/EMDataBank EM Map/Model Validation Report (i)

May 17, 2018 – 08:51 PM BST

PDB ID	:	6GJZ
EMDB ID:	:	EMD-0012
Title	:	CryoEM structure of the MDA5-dsRNA filament in complex with AMPPNP
Deposited on	:	2018-05-17
Resolution	:	4.06 Å(reported)

This is a Full wwPDB/EMDataBank EM Map/Model Validation Report.

This report is produced by the wwPDB biocuration pipeline after annotation of the structure.

We welcome your comments at *validation@mail.wwpdb.org*
A user guide is available at
https://www.wwpdb.org/validation/2017/EMValidationReportHelp
with specific help available everywhere you see the (i) symbol.

CONFIDENTIAL VALIDATION REPORT

MolProbity	:	4.02b-467
Mogul	:	1.7.3 (157068), CSD as539be (2018)
Percentile statistics	:	20171227.v01 (using entries in the PDB archive December 27th 2017)
Ideal geometry (proteins)	:	Engh & Huber (2001)
Ideal geometry (DNA, RNA)	:	Parkinson et. al. (1996)
Validation Pipeline (wwPDB-VP)	:	rb-20031021

1 Overall quality at a glance (i)

The following experimental techniques were used to determine the structure:
ELECTRON MICROSCOPY

The reported resolution of this entry is 4.06 Å.

Percentile scores (ranging between 0-100) for global validation metrics of the entry are shown in the following graphic. The table shows the number of entries on which the scores are based.

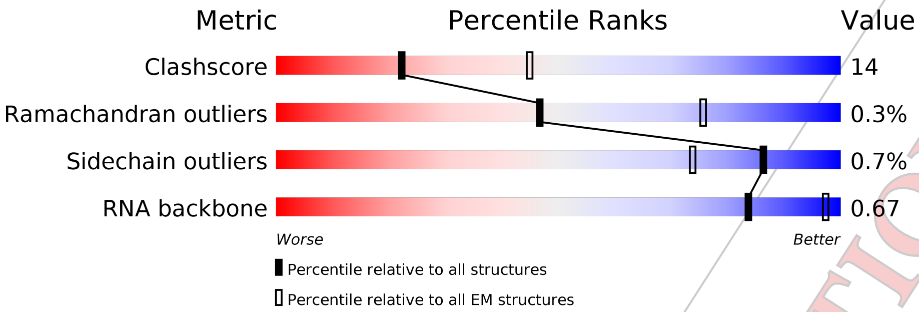

Metric	Whole archive (#Entries)	EM structures (#Entries)
Clashscore	136327	1886
Ramachandran outliers	132723	1663
Sidechain outliers	132532	1531
RNA backbone	3747	458

The table below summarises the geometric issues observed across the polymeric chains. The red, orange, yellow and green segments on the bar indicate the fraction of residues that contain outliers for >=3, 2, 1 and 0 types of geometric quality criteria. A grey segment represents the fraction of residues that are not modelled. The numeric value for each fraction is indicated below the corresponding segment, with a dot representing fractions <=5%

Mol	Chain	Length	Quality of chain
1	A	1007	52% 14% • 32%
2	X	14	100%
3	Y	14	7% 93%

CONFIDENTIAL VALIDATION REPORT

2 Entry composition (i)

There are 5 unique types of molecules in this entry. The entry contains 10503 atoms, of which 4402 are hydrogens and 0 are deuteriums.

In the tables below, the AltConf column contains the number of residues with at least one atom in alternate conformation and the Trace column contains the number of residues modelled with at most 2 atoms.

- Molecule 1 is a protein called Interferon-induced helicase C domain-containing protein 1.

Mol	Chain	Residues	Atoms						AltConf	Trace
			Total	C	H	N	O	S		
1	A	682	9874	3468	4402	950	1019	35	0	0

There are 18 discrepancies between the modelled and reference sequences:

Chain	Residue	Modelled	Actual	Comment	Reference
A	?	-	ASP	deletion	UNP Q8R5F7
A	?	-	LYS	deletion	UNP Q8R5F7
A	?	-	SER	deletion	UNP Q8R5F7
A	?	-	ASP	deletion	UNP Q8R5F7
A	?	-	ASP	deletion	UNP Q8R5F7
A	?	-	GLU	deletion	UNP Q8R5F7
A	?	-	ALA	deletion	UNP Q8R5F7
A	?	-	SER	deletion	UNP Q8R5F7
A	?	-	SER	deletion	UNP Q8R5F7
A	?	-	CYS	deletion	UNP Q8R5F7
A	?	-	ASN	deletion	UNP Q8R5F7
A	?	-	ASP	deletion	UNP Q8R5F7
A	?	-	GLN	deletion	UNP Q8R5F7
A	?	-	LEU	deletion	UNP Q8R5F7
A	?	-	LYS	deletion	UNP Q8R5F7
A	?	-	GLY	deletion	UNP Q8R5F7
A	?	-	ASP	deletion	UNP Q8R5F7
A	?	-	VAL	deletion	UNP Q8R5F7

- Molecule 2 is a RNA chain called RNA (5'-R(P*CP*AP*AP*GP*CP*CP*GP*AP*GP*GP*AP*GP*AP*G)-3').

Mol	Chain	Residues	Atoms					AltConf	Trace
			Total	C	N	O	P		
2	X	14	308	137	64	93	14	0	0

- Molecule 3 is a RNA chain called RNA (5'-R(P*CP*UP*CP*UP*CP*CP*UP*CP*GP*GP

CONFIDENTIAL VALIDATION REPORT

*CP*UP*UP*G)-3').

Mol	Chain	Residues	Atoms					AltConf	Trace
			Total	C	N	O	P		
3	Y	14	289	129	43	103	14	0	0

- Molecule 4 is ZINC ION (three-letter code: ZN) (formula: Zn).

Mol	Chain	Residues	Atoms		AltConf
			Total	Zn	
4	A	1	1	1	0

- Molecule 5 is PHOSPHOAMINOPHOSPHONIC ACID-ADENYLATE ESTER (three-letter code: ANP) (formula: $C_{10}H_{17}N_6O_{12}P_3$).

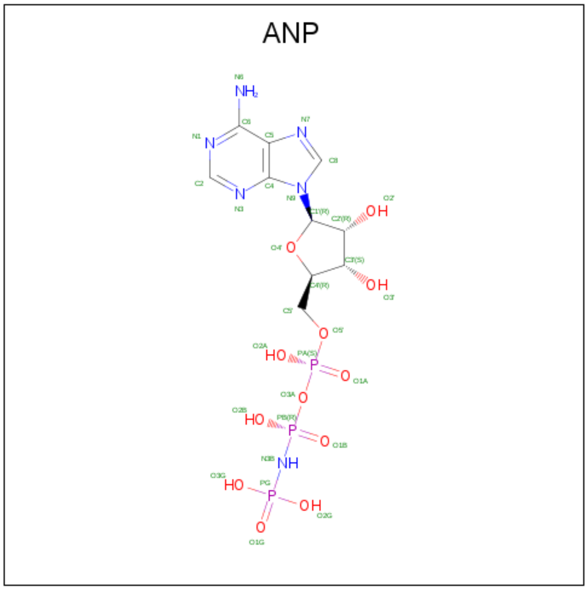

Mol	Chain	Residues	Atoms					AltConf
			Total	C	N	O	P	
5	A	1	31	10	6	12	3	0

CONFIDENTIAL VALIDATION REPORT

3 Residue-property plots (i)

These plots are drawn for all protein, RNA and DNA chains in the entry. The first graphic for a chain summarises the proportions of the various outlier classes displayed in the second graphic. The second graphic shows the sequence view annotated by issues in geometry. Residues are color-coded according to the number of geometric quality criteria for which they contain at least one outlier: green = 0, yellow = 1, orange = 2 and red = 3 or more. Stretches of 2 or more consecutive residues without any outlier are shown as a green connector. Residues present in the sample, but not in the model, are shown in grey.

• Molecule 1: Interferon-induced helicase C domain-containing protein 1

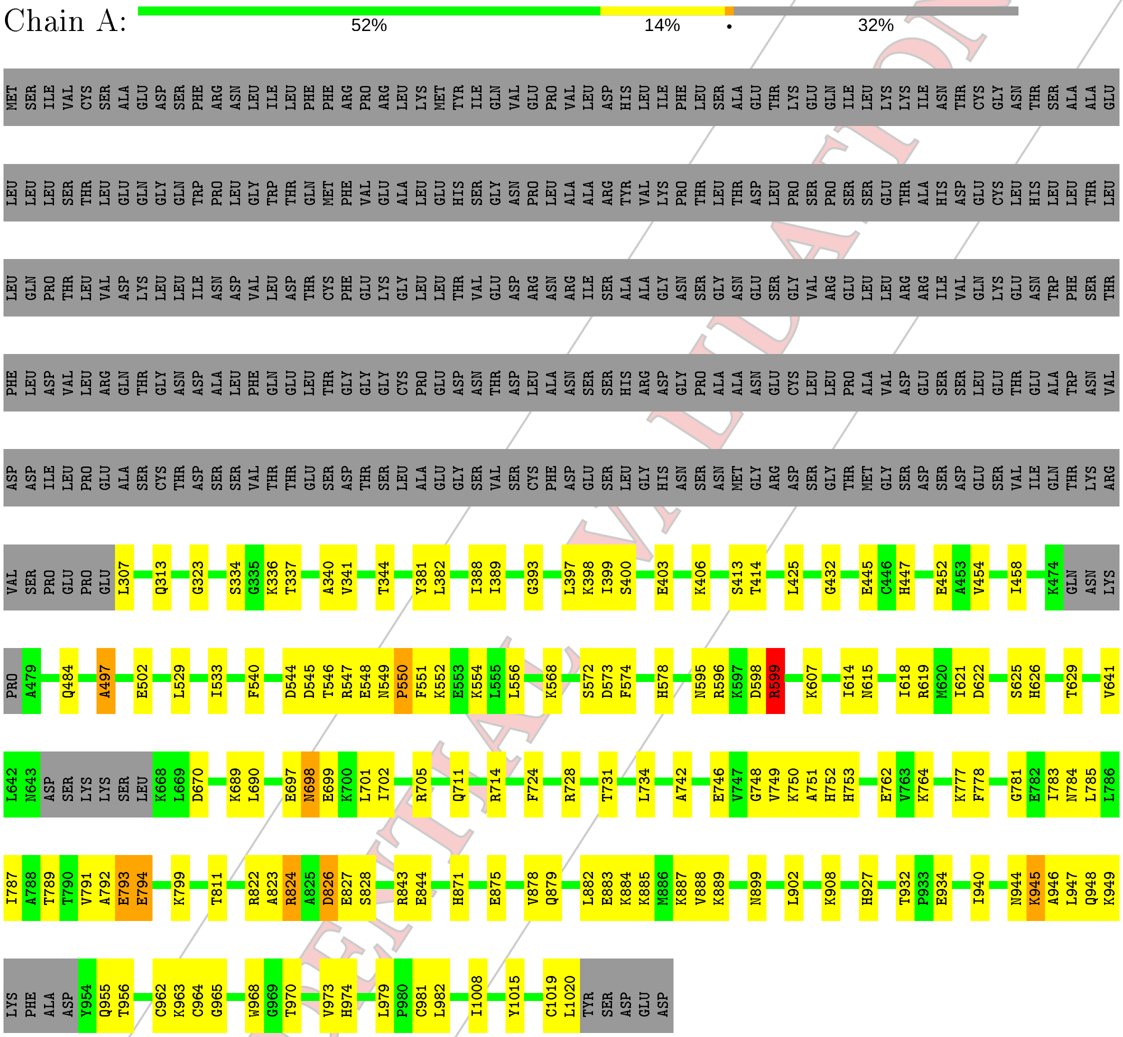

• Molecule 2: RNA (5'-R(P*CP*AP*AP*GP*CP*CP*GP*AP*GP*GP*AP*GP*AP*G)-3')

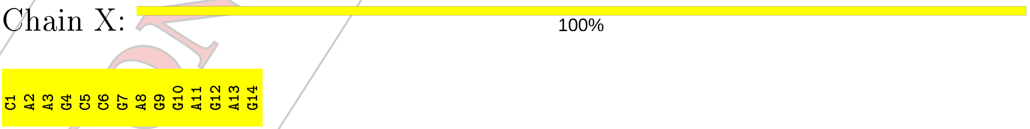

CONFIDENTIAL VALIDATION REPORT

• Molecule 3: RNA (5'-R(P*CP*UP*CP*UP*CP*CP*UP*CP*GP*GP*CP*UP*UP*G)-3')

Chain Y: 7% 93%

C1 U2 C3 U4 C5 C6 U7 C8 G9 G10 C11 U12 U13 G14

CONFIDENTIAL VALIDATION REPORT

4 Experimental information (i)

Property	Value	Source
Reconstruction method	HELICAL	Depositor
Imposed symmetry	HELICAL, twist=76.7759°, rise=43.1057 Å, axial sym=C1	Depositor
Number of segments used	28663	Depositor
Resolution determination method	FSC 0.143 CUT-OFF	Depositor
CTF correction method	PHASE FLIPPING AND AMPLITUDE CORRECTION	Depositor
Microscope	FEI TITAN KRIOS	Depositor
Voltage (kV)	300	Depositor
Electron dose ($e^-/\text{Å}^2$)	29.33	Depositor
Minimum defocus (nm)	-1800	Depositor
Maximum defocus (nm)	-2700	Depositor
Magnification	75000	Depositor
Image detector	FEI FALCON III (4k x 4k)	Depositor

CONFIDENTIAL VALIDATION REPORT

5 Model quality (i)

5.1 Standard geometry (i)

Bond lengths and bond angles in the following residue types are not validated in this section: ZN, ANP

The Z score for a bond length (or angle) is the number of standard deviations the observed value is removed from the expected value. A bond length (or angle) with $|Z| > 5$ is considered an outlier worth inspection. RMSZ is the root-mean-square of all Z scores of the bond lengths (or angles).

Mol	Chain	Bond lengths		Bond angles	
		RMSZ	#$\|Z\|$ >2	RMSZ	#$\|Z\|$ >2
1	A	0.55	0/5558	0.65	7/7469 (0.1%)
2	X	1.00	0/346	1.13	0/539
3	Y	1.02	0/319	1.21	0/493
All	All	0.62	0/6223	0.73	7/8501 (0.1%)

Chiral center outliers are detected by calculating the chiral volume of a chiral center and verifying if the center is modelled as a planar moiety or with the opposite hand.A planarity outlier is detected by checking planarity of atoms in a peptide group, atoms in a mainchain group or atoms of a sidechain that are expected to be planar.

Mol	Chain	#Chirality outliers	#Planarity outliers
1	A	0	3

There are no bond length outliers.

All (7) bond angle outliers are listed below:

Mol	Chain	Res	Type	Atoms	Z	Observed(^{o})	Ideal(^{o})
1	A	599	ARG	NE-CZ-NH1	8.81	124.70	120.30
1	A	497	ALA	C-N-CA	8.37	142.62	121.70
1	A	824	ARG	NE-CZ-NH2	-8.18	116.21	120.30
1	A	794	GLU	C-N-CA	7.86	138.81	122.30
1	A	599	ARG	NE-CZ-NH2	-7.59	116.51	120.30
1	A	793	GLU	C-N-CA	6.80	138.69	121.70
1	A	887	LYS	C-N-CA	5.90	136.46	121.70

There are no chirality outliers.

All (3) planarity outliers are listed below:

CONFIDENTIAL VALIDATION REPORT

Mol	Chain	Res	Type	Group
1	A	454	VAL	Peptide
1	A	548	GLU	Peptide
1	A	550	PRO	Peptide

5.2 Too-close contacts (i)

In the following table, the Non-H and H(model) columns list the number of non-hydrogen atoms and hydrogen atoms in the chain respectively. The H(added) column lists the number of hydrogen atoms added and optimized by MolProbity. The Clashes column lists the number of clashes within the asymmetric unit, whereas Symm-Clashes lists symmetry related clashes.

Mol	Chain	Non-H	H(model)	H(added)	Clashes	Symm-Clashes
1	A	5472	4402	5597	126	0
2	X	308	0	155	29	0
3	Y	289	0	150	26	0
4	A	1	0	0	0	0
5	A	31	0	13	2	0
All	All	6101	4402	5915	166	0

The all-atom clashscore is defined as the number of clashes found per 1000 atoms (including hydrogen atoms). The all-atom clashscore for this structure is 14.

All (166) close contacts within the same asymmetric unit are listed below, sorted by their clash magnitude.

Atom-1	Atom-2	Interatomic distance (Å)	Clash overlap (Å)
1:A:823:ALA:HB1	1:A:828:SER:HB2	1.65	0.79
1:A:341:VAL:O	1:A:344:THR:OG1	2.01	0.78
1:A:748:GLY:O	1:A:784:ASN:ND2	2.16	0.77
1:A:932:THR:OG1	1:A:934:GLU:OE1	2.02	0.77
1:A:751:ALA:HB2	1:A:785:LEU:HD21	1.67	0.75
1:A:843:ARG:CZ	2:X:12:G:H5'	2.17	0.75
1:A:962:CYS:SG	1:A:963:LYS:N	2.60	0.75
1:A:336:LYS:NZ	5:A:1102:ANP:O3G	2.21	0.73
1:A:547:ARG:NH2	1:A:699:GLU:OE2	2.22	0.72
1:A:724:PHE:HE1	1:A:792:ALA:HB3	1.54	0.72
2:X:1:C:H2'	2:X:2:A:H8	1.56	0.70
1:A:549:ASN:HB2	1:A:550:PRO:HD3	1.78	0.66
3:Y:2:U:H2'	3:Y:3:C:C6	2.30	0.65
1:A:607:LYS:NZ	1:A:762:GLU:OE2	2.31	0.64
1:A:791:VAL:CG2	3:Y:7:U:H5"	2.27	0.64
1:A:701:LEU:O	1:A:705:ARG:HG3	1.97	0.64

Continued on next page...

CONFIDENTIAL VALIDATION REPORT

Continued from previous page...

Atom-1	Atom-2	Interatomic distance (Å)	Clash overlap (Å)
2:X:11:A:H2'	2:X:12:G:C8	2.33	0.63
1:A:641:VAL:HG11	1:A:963:LYS:O	1.99	0.63
2:X:11:A:H2'	2:X:12:G:H8	1.62	0.63
3:Y:9:G:H2'	3:Y:10:G:C8	2.33	0.63
1:A:742:ALA:O	1:A:746:GLU:HG3	1.99	0.62
3:Y:2:U:H2'	3:Y:3:C:H6	1.64	0.62
5:A:1102:ANP:N3B	5:A:1102:ANP:O1A	2.30	0.62
1:A:1019:CYS:SG	1:A:1020:LEU:N	2.73	0.61
1:A:578:HIS:ND1	2:X:14:G:H4'	2.16	0.61
1:A:724:PHE:CE1	1:A:792:ALA:HB3	2.34	0.60
1:A:843:ARG:NH2	2:X:11:A:O3'	2.34	0.60
3:Y:1:C:H2'	3:Y:2:U:H6	1.66	0.60
1:A:824:ARG:N	1:A:824:ARG:HD2	2.16	0.60
2:X:8:A:H2'	2:X:9:G:H8	1.66	0.60
1:A:728:ARG:O	1:A:731:THR:OG1	2.15	0.60
2:X:8:A:H2'	2:X:9:G:C8	2.37	0.60
1:A:313:GLN:NE2	1:A:334:SER:O	2.34	0.60
1:A:389:ILE:HD11	1:A:397:LEU:HD13	1.84	0.60
3:Y:13:U:H2'	3:Y:14:G:O4'	2.01	0.59
1:A:445:GLU:OE1	1:A:447:HIS:NE2	2.35	0.59
1:A:944:ASN:ND2	1:A:947:LEU:HG	2.17	0.59
1:A:974:HIS:HB3	1:A:979:LEU:HD21	1.83	0.59
1:A:899:ASN:HB2	1:A:1008:ILE:CG2	2.33	0.58
3:Y:1:C:H2'	3:Y:2:U:C6	2.37	0.58
1:A:413:SER:OG	1:A:414:THR:O	2.19	0.58
1:A:899:ASN:HB3	1:A:902:LEU:HD13	1.85	0.58
1:A:981:CYS:SG	1:A:982:LEU:N	2.76	0.58
3:Y:3:C:H2'	3:Y:4:U:H6	1.69	0.58
3:Y:7:U:O2'	3:Y:8:C:H5'	2.04	0.58
2:X:1:C:H2'	2:X:2:A:C8	2.39	0.57
1:A:944:ASN:H	1:A:955:GLN:HG2	1.68	0.57
1:A:979:LEU:H	1:A:979:LEU:HD23	1.68	0.57
2:X:6:C:H2'	2:X:7:G:H8	1.69	0.57
1:A:964:CYS:SG	1:A:965:GLY:N	2.78	0.57
1:A:734:LEU:HB2	1:A:787:ILE:HD13	1.87	0.57
3:Y:4:U:H2'	3:Y:5:C:H6	1.70	0.56
1:A:750:LYS:O	1:A:785:LEU:HD23	2.05	0.56
1:A:945:LYS:H	1:A:945:LYS:HD3	1.70	0.56
1:A:307:LEU:HB3	1:A:381:TYR:CD2	2.41	0.56
1:A:641:VAL:HA	1:A:940:ILE:CD1	2.36	0.56
1:A:791:VAL:HG23	3:Y:7:U:H5"	1.88	0.55

Continued on next page...

CONFIDENTIAL VALIDATION REPORT

Continued from previous page...

Atom-1	Atom-2	Interatomic distance (Å)	Clash overlap (Å)
1:A:927:HIS:CE1	2:X:4:G:H1'	2.42	0.55
1:A:945:LYS:O	1:A:948:GLN:HG3	2.06	0.55
1:A:970:THR:HG21	2:X:3:A:H4'	1.89	0.55
2:X:6:C:H2'	2:X:7:G:C8	2.42	0.55
1:A:573:ASP:OD1	1:A:574:PHE:N	2.40	0.55
1:A:927:HIS:HE1	2:X:4:G:H1'	1.71	0.55
3:Y:3:C:H2'	3:Y:4:U:C6	2.42	0.55
1:A:752:HIS:HB2	1:A:783:ILE:HG12	1.89	0.54
1:A:614:ILE:O	1:A:618:ILE:N	2.39	0.53
3:Y:4:U:H2'	3:Y:5:C:C6	2.44	0.53
1:A:944:ASN:HD22	1:A:947:LEU:HG	1.74	0.53
1:A:711:GLN:OE1	1:A:714:ARG:NH2	2.42	0.52
1:A:752:HIS:ND1	1:A:753:HIS:O	2.42	0.52
1:A:425:LEU:HB2	1:A:432:GLY:HA3	1.92	0.51
1:A:547:ARG:HB3	1:A:549:ASN:OD1	2.09	0.51
2:X:7:G:O2'	2:X:8:A:H5'	2.10	0.51
1:A:885:LYS:O	1:A:889:LYS:HG2	2.10	0.51
1:A:843:ARG:NH1	2:X:12:G:H5'	2.26	0.51
1:A:885:LYS:O	1:A:888:VAL:HB	2.11	0.51
3:Y:5:C:O2'	3:Y:6:C:H5'	2.10	0.50
1:A:702:ILE:HA	1:A:705:ARG:HD3	1.93	0.50
1:A:323:GLY:O	1:A:484:GLN:NE2	2.45	0.50
2:X:9:G:O2'	2:X:10:G:H5'	2.12	0.50
2:X:9:G:H2'	2:X:10:G:H8	1.76	0.50
3:Y:10:G:O2'	3:Y:11:C:H5'	2.12	0.49
1:A:947:LEU:CD1	1:A:956:THR:H	2.25	0.49
1:A:749:VAL:HG13	1:A:785:LEU:HD22	1.94	0.49
2:X:5:C:H2'	2:X:6:C:C6	2.48	0.49
1:A:698:ASN:OD1	1:A:699:GLU:N	2.46	0.48
1:A:899:ASN:HB2	1:A:1008:ILE:HG21	1.95	0.48
1:A:899:ASN:HB2	1:A:1008:ILE:HG22	1.95	0.48
1:A:393:GLY:HA2	1:A:398:LYS:HD3	1.96	0.48
1:A:399:ILE:HG13	1:A:400:SER:N	2.29	0.48
1:A:879:GLN:O	1:A:883:GLU:HG3	2.13	0.48
1:A:596:ARG:NH1	1:A:670:ASP:OD2	2.45	0.48
1:A:626:HIS:O	1:A:629:THR:OG1	2.28	0.48
2:X:2:A:O2'	2:X:3:A:H5'	2.14	0.47
1:A:750:LYS:O	1:A:784:ASN:N	2.42	0.47
1:A:791:VAL:HG22	3:Y:7:U:H5"	1.95	0.47
1:A:697:GLU:O	1:A:698:ASN:HB2	2.15	0.47
1:A:750:LYS:H	1:A:784:ASN:HD22	1.62	0.47

Continued on next page...

CONFIDENTIAL VALIDATION REPORT

Continued from previous page...

Atom-1	Atom-2	Interatomic distance (Å)	Clash overlap (Å)
2:X:5:C:O2'	2:X:6:C:H5'	2.15	0.47
1:A:337:THR:O	1:A:340:ALA:HB3	2.14	0.47
2:X:8:A:O2'	2:X:9:G:H5'	2.15	0.47
1:A:714:ARG:NH2	1:A:827:GLU:O	2.47	0.46
1:A:731:THR:HG22	1:A:787:ILE:HG22	1.97	0.46
3:Y:3:C:O2'	3:Y:4:U:H5'	2.15	0.46
1:A:552:LYS:HG2	1:A:556:LEU:CD1	2.46	0.46
1:A:702:ILE:O	1:A:705:ARG:HB2	2.16	0.46
1:A:826:ASP:OD1	1:A:827:GLU:N	2.49	0.46
1:A:962:CYS:HB2	1:A:968:TRP:CE2	2.51	0.45
2:X:13:A:H2'	2:X:14:G:C8	2.51	0.45
1:A:811:THR:O	2:X:11:A:H4'	2.15	0.45
3:Y:8:C:H2'	3:Y:9:G:C8	2.51	0.45
1:A:546:THR:C	1:A:547:ARG:HD3	2.37	0.45
2:X:13:A:H2'	2:X:14:G:H8	1.82	0.45
1:A:403:GLU:OE1	1:A:406:LYS:HD2	2.17	0.45
1:A:540:PHE:CZ	1:A:844:GLU:HG2	2.52	0.45
2:X:3:A:H2'	2:X:4:G:O4'	2.17	0.44
1:A:878:VAL:O	1:A:882:LEU:HD13	2.17	0.44
1:A:393:GLY:HA2	1:A:398:LYS:CE	2.47	0.44
3:Y:9:G:H2'	3:Y:10:G:H8	1.78	0.44
1:A:884:LYS:O	1:A:888:VAL:HG23	2.17	0.44
1:A:778:PHE:CE2	1:A:799:LYS:HB3	2.53	0.44
1:A:793:GLU:O	1:A:794:GLU:HG3	2.17	0.44
3:Y:6:C:O2'	3:Y:7:U:H5'	2.18	0.43
1:A:1015:TYR:O	1:A:1019:CYS:HB3	2.17	0.43
1:A:947:LEU:HD13	1:A:955:GLN:HA	2.00	0.43
1:A:822:ARG:O	1:A:824:ARG:HD2	2.18	0.43
3:Y:10:G:C2'	3:Y:11:C:H5'	2.48	0.43
1:A:785:LEU:HD23	1:A:785:LEU:H	1.84	0.43
1:A:944:ASN:HD21	1:A:946:ALA:HB3	1.83	0.43
1:A:550:PRO:HB2	1:A:690:LEU:HD23	2.00	0.43
1:A:689:LYS:HA	1:A:689:LYS:HE2	1.99	0.43
3:Y:8:C:O2'	3:Y:9:G:H5'	2.19	0.43
1:A:551:PHE:O	1:A:554:LYS:HB3	2.18	0.43
3:Y:4:U:O2'	3:Y:5:C:H5'	2.18	0.43
1:A:568:LYS:HE2	1:A:598:ASP:OD1	2.18	0.43
1:A:545:ASP:OD1	1:A:545:ASP:N	2.52	0.42
1:A:871:HIS:NE2	1:A:875:GLU:OE2	2.52	0.42
1:A:974:HIS:CB	1:A:979:LEU:HD21	2.47	0.42
1:A:619:ARG:HE	1:A:621:ILE:CG2	2.33	0.42

Continued on next page...

CONFIDENTIAL VALIDATION REPORT

Continued from previous page...

Atom-1	Atom-2	Interatomic distance (Å)	Clash overlap (Å)
1:A:458:ILE:H	1:A:458:ILE:HD12	1.83	0.42
1:A:826:ASP:O	1:A:827:GLU:HB2	2.19	0.42
1:A:393:GLY:HA2	1:A:398:LYS:HE2	2.01	0.42
1:A:552:LYS:HG2	1:A:556:LEU:HD13	2.02	0.42
1:A:973:VAL:HG12	3:Y:14:G:H4'	2.00	0.42
1:A:497:ALA:HB1	1:A:502:GLU:HB2	2.00	0.42
1:A:452:GLU:O	2:X:8:A:H4'	2.20	0.42
1:A:792:ALA:O	1:A:822:ARG:NH2	2.53	0.42
1:A:973:VAL:O	3:Y:14:G:H5'	2.20	0.41
1:A:714:ARG:NH2	1:A:827:GLU:HB3	2.36	0.41
1:A:764:LYS:HE2	1:A:949:LYS:HG3	2.02	0.41
1:A:545:ASP:HB3	1:A:699:GLU:CD	2.41	0.41
1:A:751:ALA:HB2	1:A:785:LEU:CD2	2.46	0.41
1:A:944:ASN:OD1	1:A:945:LYS:N	2.54	0.41
1:A:777:LYS:O	1:A:781:GLY:N	2.52	0.41
1:A:572:SER:OG	1:A:573:ASP:N	2.54	0.41
1:A:622:ASP:O	1:A:625:SER:OG	2.32	0.41
1:A:927:HIS:CE1	2:X:4:G:HO2'	2.39	0.41
1:A:399:ILE:HG13	1:A:400:SER:H	1.86	0.41
1:A:789:THR:OG1	1:A:791:VAL:HG23	2.21	0.41
1:A:529:LEU:O	1:A:533:ILE:N	2.42	0.40
1:A:544:ASP:OD1	1:A:545:ASP:N	2.53	0.40
1:A:973:VAL:CG1	3:Y:14:G:H4'	2.52	0.40
1:A:382:LEU:HB2	1:A:388:ILE:HD11	2.03	0.40
1:A:599:ARG:HH21	1:A:908:LYS:HG2	1.86	0.40
1:A:731:THR:HA	1:A:787:ILE:HG21	2.03	0.40
2:X:13:A:O2'	2:X:14:G:H5'	2.21	0.40

There are no symmetry-related clashes.

5.3 Torsion angles (i)

5.3.1 Protein backbone (i)

In the following table, the Percentiles column shows the percent Ramachandran outliers of the chain as a percentile score with respect to all PDB entries followed by that with respect to all EM entries.

The Analysed column shows the number of residues for which the backbone conformation was analysed, and the total number of residues.

CONFIDENTIAL VALIDATION REPORT

Mol	Chain	Analysed	Favoured	Allowed	Outliers	Percentiles	
1	A	674/1007 (67%)	599 (89%)	73 (11%)	2 (0%)	43	80

All (2) Ramachandran outliers are listed below:

Mol	Chain	Res	Type
1	A	698	ASN
1	A	826	ASP

5.3.2 Protein sidechains (i)

In the following table, the Percentiles column shows the percent sidechain outliers of the chain as a percentile score with respect to all PDB entries followed by that with respect to all EM entries.

The Analysed column shows the number of residues for which the sidechain conformation was analysed, and the total number of residues.

Mol	Chain	Analysed	Rotameric	Outliers	Percentiles	
1	A	608/900 (68%)	604 (99%)	4 (1%)	85	93

All (4) residues with a non-rotameric sidechain are listed below:

Mol	Chain	Res	Type
1	A	595	ASN
1	A	599	ARG
1	A	615	ASN
1	A	945	LYS

Some sidechains can be flipped to improve hydrogen bonding and reduce clashes. All (7) such sidechains are listed below:

Mol	Chain	Res	Type
1	A	372	GLN
1	A	420	ASN
1	A	563	GLN
1	A	595	ASN
1	A	678	ASN
1	A	784	ASN
1	A	957	ASN

5.3.3 RNA (i)

CONFIDENTIAL VALIDATION REPORT

Mol	Chain	Analysed	Backbone Outliers	Pucker Outliers
2	X	13/14 (92%)	0	0
3	Y	13/14 (92%)	0	0
All	All	26/28 (92%)	0	0

There are no RNA backbone outliers to report.

There are no RNA pucker outliers to report.

5.4 Non-standard residues in protein, DNA, RNA chains (i)

There are no non-standard protein/DNA/RNA residues in this entry.

5.5 Carbohydrates (i)

There are no carbohydrates in this entry.

5.6 Ligand geometry (i)

Of 2 ligands modelled in this entry, 1 is monoatomic - leaving 1 for Mogul analysis.

In the following table, the Counts columns list the number of bonds (or angles) for which Mogul statistics could be retrieved, the number of bonds (or angles) that are observed in the model and the number of bonds (or angles) that are defined in the Chemical Component Dictionary. The Link column lists molecule types, if any, to which the group is linked. The Z score for a bond length (or angle) is the number of standard deviations the observed value is removed from the expected value. A bond length (or angle) with $|Z| > 2$ is considered an outlier worth inspection. RMSZ is the root-mean-square of all Z scores of the bond lengths (or angles).

Mol	Type	Chain	Res	Link	Bond lengths			Bond angles		
					Counts	RMSZ	$\#\|Z\| > 2$	Counts	RMSZ	$\#\|Z\| > 2$
5	ANP	A	1102	-	29,33,33	2.76	6 (20%)	29,52,52	1.33	3 (10%)

In the following table, the Chirals column lists the number of chiral outliers, the number of chiral centers analysed, the number of these observed in the model and the number defined in the Chemical Component Dictionary. Similar counts are reported in the Torsion and Rings columns. '-' means no outliers of that kind were identified.

Mol	Type	Chain	Res	Link	Chirals	Torsions	Rings
5	ANP	A	1102	-	-	0/13/38/38	0/3/3/3

All (6) bond length outliers are listed below:

CONFIDENTIAL VALIDATION REPORT

Mol	Chain	Res	Type	Atoms	Z	Observed(Å)	Ideal(Å)
5	A	1102	ANP	PB-O3A	-2.72	1.55	1.59
5	A	1102	ANP	PB-O2B	-2.49	1.49	1.56
5	A	1102	ANP	PG-O2G	-2.39	1.50	1.56
5	A	1102	ANP	PG-N3B	2.13	1.69	1.63
5	A	1102	ANP	PB-O1B	8.65	1.55	1.46
5	A	1102	ANP	PG-O1G	10.51	1.58	1.46

All (3) bond angle outliers are listed below:

Mol	Chain	Res	Type	Atoms	Z	Observed(^{o})	Ideal(^{o})
5	A	1102	ANP	PA-O3A-PB	-4.57	116.36	132.40
5	A	1102	ANP	O1B-PB-N3B	-2.93	107.40	111.79
5	A	1102	ANP	O1G-PG-N3B	-2.31	108.33	111.79

There are no chirality outliers.

There are no torsion outliers.

There are no ring outliers.

1 monomer is involved in 2 short contacts:

Mol	Chain	Res	Type	Clashes	Symm-Clashes
5	A	1102	ANP	2	0

5.7 Other polymers (i)

There are no such residues in this entry.

5.8 Polymer linkage issues (i)

There are no chain breaks in this entry.

CONFIDENTIAL VALIDATION REPORT

Click here to access/download;PDB Validation Report;D_1200010091_val-report-full-annotate_P1_ADPAlF4.pdf

WORLDWIDE PDB PROTEIN DATA BANK

EMDataBank Unified Data Resource for 3DEM

Full wwPDB/EMDataBank EM Map/Model Validation Report (i)

May 21, 2018 – 03:39 PM BST

PDB ID	:	6GKH
EMDB ID:	:	4338
Title	:	CryoEM structure of the MDA5-dsRNA filament in complex with ADP-AlF4
Deposited on	:	2018-05-21
Resolution	:	4.06 Å(reported)

This is a Full wwPDB/EMDataBank EM Map/Model Validation Report.

This report is produced by the wwPDB biocuration pipeline after annotation of the structure.

We welcome your comments at *validation@mail.wwpdb.org*
A user guide is available at
https://www.wwpdb.org/validation/2017/EMValidationReportHelp
with specific help available everywhere you see the (i) symbol.

MolProbity	:	4.02b-467
Mogul	:	1.7.3 (157068), CSD as539be (2018)
Percentile statistics	:	20171227.v01 (using entries in the PDB archive December 27th 2017)
Ideal geometry (proteins)	:	Engh & Huber (2001)
Ideal geometry (DNA, RNA)	:	Parkinson et. al. (1996)
Validation Pipeline (wwPDB-VP)	:	rb-20031021

CONFIDENTIAL VALIDATION REPORT

1 Overall quality at a glance (i)

The following experimental techniques were used to determine the structure:
ELECTRON MICROSCOPY

The reported resolution of this entry is 4.06 Å.

Percentile scores (ranging between 0-100) for global validation metrics of the entry are shown in the following graphic. The table shows the number of entries on which the scores are based.

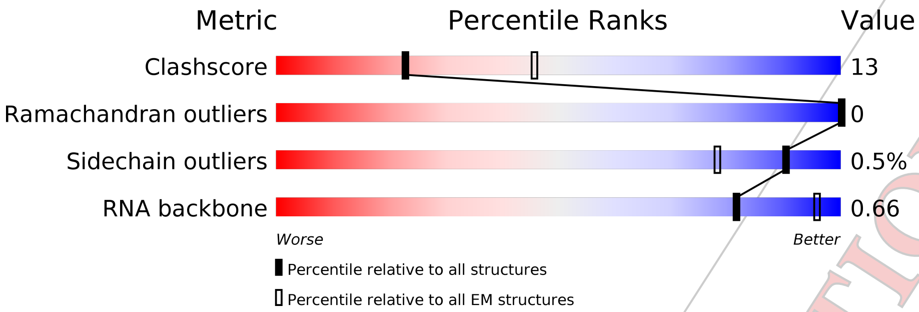

Metric	Whole archive (#Entries)	EM structures (#Entries)
Clashscore	136327	1886
Ramachandran outliers	132723	1663
Sidechain outliers	132532	1531
RNA backbone	3747	458

The table below summarises the geometric issues observed across the polymeric chains. The red, orange, yellow and green segments on the bar indicate the fraction of residues that contain outliers for >=3, 2, 1 and 0 types of geometric quality criteria. A grey segment represents the fraction of residues that are not modelled. The numeric value for each fraction is indicated below the corresponding segment, with a dot representing fractions <=5%

Mol	Chain	Length	Quality of chain
1	A	1007	51% 16% • 32%
2	X	15	13% 87%
3	Y	15	7% 87% 7%

CONFIDENTIAL VALIDATION REPORT

2 Entry composition (i)

There are 7 unique types of molecules in this entry. The entry contains 10599 atoms, of which 4411 are hydrogens and 0 are deuteriums.

In the tables below, the AltConf column contains the number of residues with at least one atom in alternate conformation and the Trace column contains the number of residues modelled with at most 2 atoms.

- Molecule 1 is a protein called Interferon-induced helicase C domain-containing protein 1.

Mol	Chain	Residues	Atoms						AltConf	Trace
			Total	C	H	N	O	S		
1	A	685	9914	3494	4399	956	1030	35	0	0

There are 18 discrepancies between the modelled and reference sequences:

Chain	Residue	Modelled	Actual	Comment	Reference
A	?	-	ASP	deletion	UNP Q8R5F7
A	?	-	LYS	deletion	UNP Q8R5F7
A	?	-	SER	deletion	UNP Q8R5F7
A	?	-	ASP	deletion	UNP Q8R5F7
A	?	-	ASP	deletion	UNP Q8R5F7
A	?	-	GLU	deletion	UNP Q8R5F7
A	?	-	ALA	deletion	UNP Q8R5F7
A	?	-	SER	deletion	UNP Q8R5F7
A	?	-	SER	deletion	UNP Q8R5F7
A	?	-	CYS	deletion	UNP Q8R5F7
A	?	-	ASN	deletion	UNP Q8R5F7
A	?	-	ASP	deletion	UNP Q8R5F7
A	?	-	GLN	deletion	UNP Q8R5F7
A	?	-	LEU	deletion	UNP Q8R5F7
A	?	-	LYS	deletion	UNP Q8R5F7
A	?	-	GLY	deletion	UNP Q8R5F7
A	?	-	ASP	deletion	UNP Q8R5F7
A	?	-	VAL	deletion	UNP Q8R5F7

- Molecule 2 is a RNA chain called RNA (5'-R(P*GP*UP*CP*AP*AP*GP*CP*CP*GP*AP *GP*GP*AP*GP*A)-3').

Mol	Chain	Residues	Atoms					AltConf	Trace
			Total	C	N	O	P		
2	X	15	328	146	66	101	15	0	0

- Molecule 3 is a RNA chain called RNA (5'-R(P*UP*CP*UP*CP*CP*UP*CP*GP*GP*CP

CONFIDENTIAL VALIDATION REPORT

*UP*UP*GP*AP*C)-3').

Mol	Chain	Residues	Atoms					AltConf	Trace
			Total	C	N	O	P		
3	Y	15	311	139	48	109	15	0	0

- Molecule 4 is ADENOSINE-5'-DIPHOSPHATE (three-letter code: ADP) (formula: $C_{10}H_{15}N_5O_{10}P_2$).

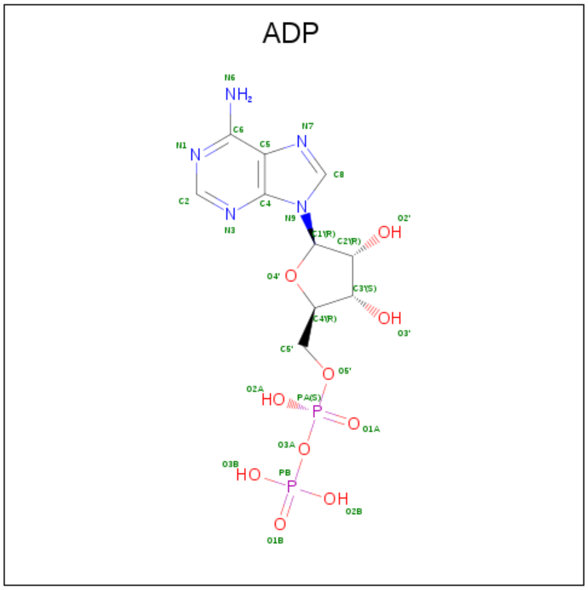

Mol	Chain	Residues	Atoms						AltConf
			Total	C	H	N	O	P	
4	A	1	39	10	12	5	10	2	0

- Molecule 5 is TETRAFLUOROALUMINATE ION (three-letter code: ALF) (formula: AlF_4).

CONFIDENTIAL VALIDATION REPORT

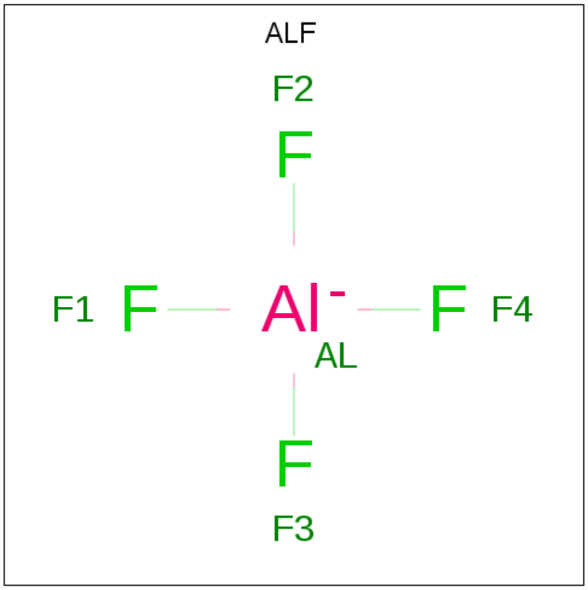

Mol	Chain	Residues	Atoms	AltConf
5	A	1	Total Al F<br>5 1 4	0

- Molecule 6 is MAGNESIUM ION (three-letter code: MG) (formula: Mg).

Mol	Chain	Residues	Atoms	AltConf
6	A	1	Total Mg<br>1 1	0

- Molecule 7 is ZINC ION (three-letter code: ZN) (formula: Zn).

Mol	Chain	Residues	Atoms	AltConf
7	A	1	Total Zn<br>1 1	0

CONFIDENTIAL VALIDATION REPORT

3 Residue-property plots ⓘ

These plots are drawn for all protein, RNA and DNA chains in the entry. The first graphic for a chain summarises the proportions of the various outlier classes displayed in the second graphic. The second graphic shows the sequence view annotated by issues in geometry. Residues are color-coded according to the number of geometric quality criteria for which they contain at least one outlier: green = 0, yellow = 1, orange = 2 and red = 3 or more. Stretches of 2 or more consecutive residues without any outlier are shown as a green connector. Residues present in the sample, but not in the model, are shown in grey.

• Molecule 1: Interferon-induced helicase C domain-containing protein 1

Chain A:

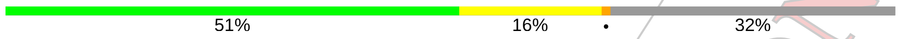

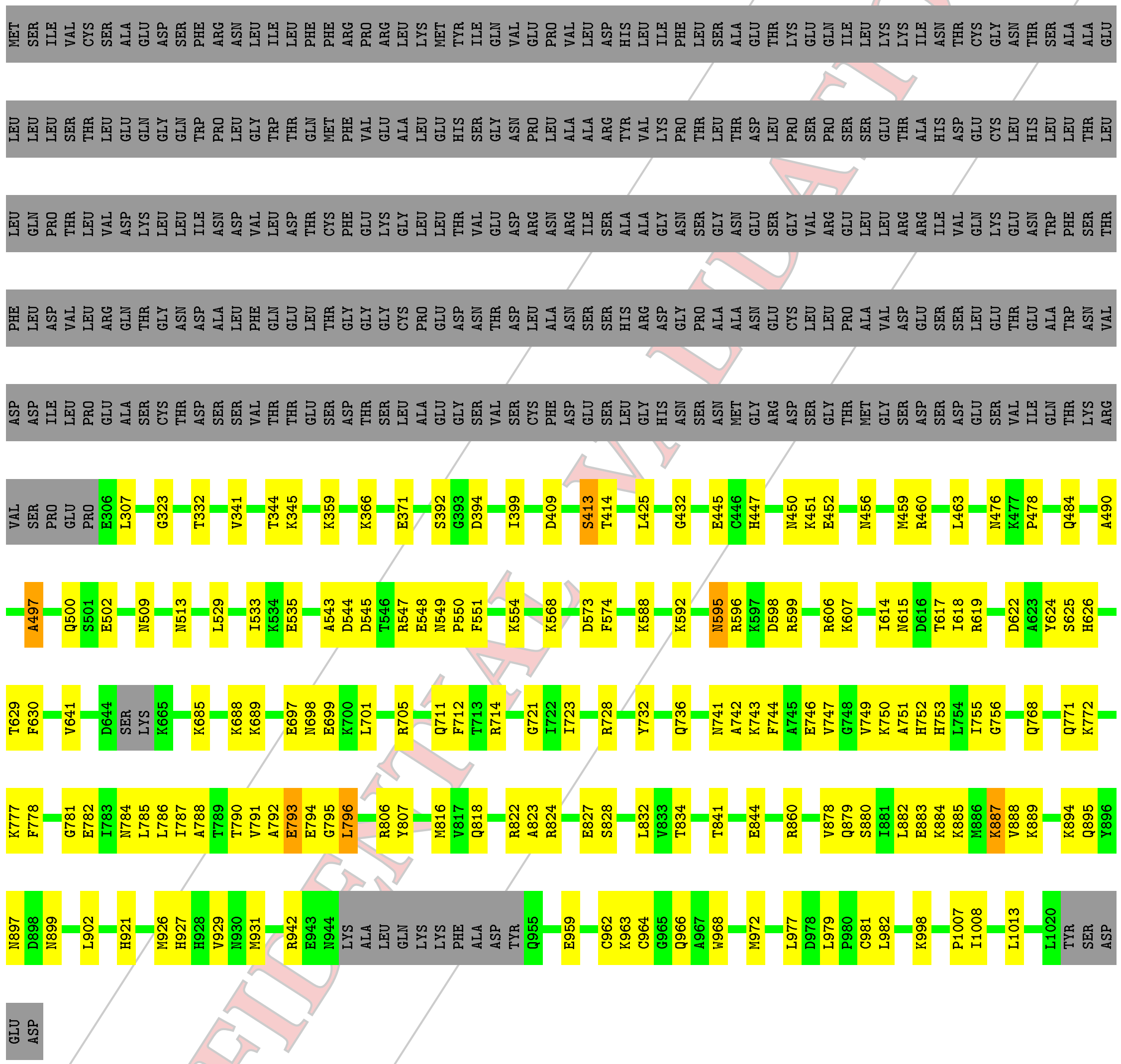

• Molecule 2: RNA (5’-R(P*GP*UP*CP*AP*AP*GP*CP*CP*GP*AP*GP*GP*AP*GP*A)-3’)

Chain X:

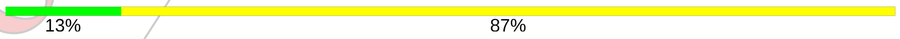

G1 U2 C3 A4 A5 G6 C7 C8 G9 A10 G11 G12 A13 G14 A15

• Molecule 3: RNA (5'-R(P*UP*CP*UP*CP*CP*UP*CP*GP*GP*CP*UP*UP*GP*AP*C)-3'
)

Chain Y: 7% 87% 7%

U1 C2 U3 C4 C5 U6 C7 G8 G9 C10 U11 U12 G13 A14 C15

CONFIDENTIAL VALIDATION REPORT

4 Experimental information (i)

Property	Value	Source
Reconstruction method	HELICAL	Depositor
Imposed symmetry	HELICAL, twist=87.8315°, rise=46.5105 Å, axial sym=C1	Depositor
Number of segments used	31556	Depositor
Resolution determination method	FSC 0.143 CUT-OFF	Depositor
CTF correction method	PHASE FLIPPING AND AMPLITUDE CORRECTION	Depositor
Microscope	FEI TITAN KRIOS	Depositor
Voltage (kV)	300	Depositor
Electron dose ($e^-/\text{Å}^2$)	29.85	Depositor
Minimum defocus (nm)	-1800	Depositor
Maximum defocus (nm)	-2700	Depositor
Magnification	75000	Depositor
Image detector	FEI FALCON III (4k x 4k)	Depositor

CONFIDENTIAL VALIDATION REPORT

5 Model quality (i)

5.1 Standard geometry (i)

Bond lengths and bond angles in the following residue types are not validated in this section: ALF, ZN, MG, ADP

The Z score for a bond length (or angle) is the number of standard deviations the observed value is removed from the expected value. A bond length (or angle) with $|Z| > 5$ is considered an outlier worth inspection. RMSZ is the root-mean-square of all Z scores of the bond lengths (or angles).

Mol	Chain	Bond lengths		Bond angles	
		RMSZ	#$|Z|$ >2	RMSZ	#$|Z|$ >2
1	A	0.33	0/5604	0.58	7/7532 (0.1%)
2	X	0.42	0/368	0.88	0/573
3	Y	0.37	0/344	0.85	0/532
All	All	0.33	0/6316	0.62	7/8637 (0.1%)

Chiral center outliers are detected by calculating the chiral volume of a chiral center and verifying if the center is modelled as a planar moiety or with the opposite hand.A planarity outlier is detected by checking planarity of atoms in a peptide group, atoms in a mainchain group or atoms of a sidechain that are expected to be planar.

Mol	Chain	#Chirality outliers	#Planarity outliers
1	A	0	1

There are no bond length outliers.

All (7) bond angle outliers are listed below:

Mol	Chain	Res	Type	Atoms	Z	Observed(^{o})	Ideal(^{o})
1	A	497	ALA	C-N-CA	10.74	148.54	121.70
1	A	399	ILE	C-N-CA	7.61	140.72	121.70
1	A	827	GLU	C-N-CA	6.48	137.89	121.70
1	A	887	LYS	C-N-CA	5.57	135.62	121.70
1	A	451	LYS	C-N-CA	5.47	135.37	121.70
1	A	793	GLU	C-N-CA	5.02	134.25	121.70
1	A	796	LEU	CA-CB-CG	5.01	126.82	115.30

There are no chirality outliers.

All (1) planarity outliers are listed below:

CONFIDENTIAL VALIDATION REPORT

Mol	Chain	Res	Type	Group
1	A	413	SER	Peptide

5.2 Too-close contacts (i)

In the following table, the Non-H and H(model) columns list the number of non-hydrogen atoms and hydrogen atoms in the chain respectively. The H(added) column lists the number of hydrogen atoms added and optimized by MolProbity. The Clashes column lists the number of clashes within the asymmetric unit, whereas Symm-Clashes lists symmetry related clashes.

Mol	Chain	Non-H	H(model)	H(added)	Clashes	Symm-Clashes
1	A	5515	4399	5641	122	0
2	X	328	0	165	24	0
3	Y	311	0	161	26	0
4	A	27	12	12	1	0
5	A	5	0	0	0	0
6	A	1	0	0	0	0
7	A	1	0	0	0	0
All	All	6188	4411	5979	164	0

The all-atom clashscore is defined as the number of clashes found per 1000 atoms (including hydrogen atoms). The all-atom clashscore for this structure is 13.

All (164) close contacts within the same asymmetric unit are listed below, sorted by their clash magnitude.

Atom-1	Atom-2	Interatomic distance (Å)	Clash overlap (Å)
1:A:543:ALA:HB1	1:A:699:GLU:HB3	1.52	0.92
1:A:749:VAL:HG12	1:A:785:LEU:HD22	1.59	0.82
1:A:790:THR:HG1	3:Y:6:U:HO2'	1.14	0.81
1:A:749:VAL:HA	1:A:784:ASN:HD22	1.47	0.79
1:A:751:ALA:HB2	1:A:785:LEU:HD21	1.67	0.75
1:A:359:LYS:NZ	1:A:409:ASP:OD1	2.20	0.74
1:A:392:SER:OG	1:A:394:ASP:OD1	2.06	0.73
1:A:705:ARG:HH21	1:A:743:LYS:HD2	1.54	0.73
1:A:341:VAL:O	1:A:344:THR:OG1	2.09	0.70
1:A:962:CYS:SG	1:A:963:LYS:N	2.65	0.70
1:A:476:ASN:HB3	1:A:478:PRO:HD2	1.75	0.69
1:A:791:VAL:HG22	3:Y:7:C:H5"	1.75	0.68
2:X:8:C:H2'	2:X:9:G:C8	2.29	0.67
1:A:529:LEU:O	1:A:533:ILE:N	2.27	0.67
2:X:8:C:H2'	2:X:9:G:H8	1.60	0.66
1:A:824:ARG:NH2	4:A:1101:ADP:O2B	2.28	0.66

Continued on next page...

CONFIDENTIAL VALIDATION REPORT

Continued from previous page...

Atom-1	Atom-2	Interatomic distance (Å)	Clash overlap (Å)
1:A:595:ASN:ND2	1:A:596:ARG:O	2.30	0.64
1:A:460:ARG:NH1	1:A:880:SER:O	2.32	0.62
2:X:10:A:H2'	2:X:11:G:H8	1.63	0.62
2:X:10:A:H2'	2:X:11:G:C8	2.34	0.62
3:Y:3:U:H2'	3:Y:4:C:C6	2.36	0.61
1:A:607:LYS:HE3	1:A:630:PHE:CE1	2.35	0.61
1:A:931:MET:HE3	1:A:977:LEU:HB3	1.82	0.61
1:A:979:LEU:HD23	1:A:979:LEU:H	1.66	0.61
1:A:624:TYR:CZ	1:A:688:LYS:HB2	2.36	0.60
3:Y:2:C:H2'	3:Y:3:U:C6	2.37	0.60
3:Y:2:C:H2'	3:Y:3:U:H6	1.67	0.58
1:A:701:LEU:O	1:A:705:ARG:HG3	2.04	0.58
2:X:5:A:H2'	2:X:6:G:C8	2.38	0.58
1:A:841:THR:O	1:A:844:GLU:HB3	2.04	0.58
1:A:323:GLY:O	1:A:484:GLN:NE2	2.37	0.58
1:A:614:ILE:O	1:A:618:ILE:N	2.37	0.58
1:A:962:CYS:HB3	1:A:966:GLN:H	1.69	0.57
1:A:791:VAL:CG2	3:Y:7:C:H5"	2.34	0.57
3:Y:9:G:O2'	3:Y:10:C:H5'	2.04	0.57
1:A:697:GLU:HG3	1:A:698:ASN:H	1.69	0.57
1:A:617:THR:O	1:A:807:TYR:OH	2.23	0.56
2:X:11:G:H2'	2:X:12:G:H8	1.70	0.56
1:A:592:LYS:HE2	1:A:998:LYS:HA	1.87	0.56
2:X:12:G:O2'	2:X:13:A:H5'	2.05	0.56
1:A:795:GLY:O	1:A:822:ARG:NH1	2.38	0.56
1:A:926:MET:CE	2:X:7:C:H1'	2.35	0.56
3:Y:1:U:O2'	3:Y:2:C:H5'	2.06	0.56
1:A:897:ASN:HB3	1:A:899:ASN:ND2	2.21	0.56
3:Y:3:U:H2'	3:Y:4:C:H6	1.70	0.56
1:A:723:ILE:HD12	1:A:785:LEU:HD12	1.87	0.55
1:A:885:LYS:O	1:A:889:LYS:HG2	2.07	0.55
1:A:549:ASN:OD1	1:A:550:PRO:HD3	2.06	0.55
1:A:705:ARG:HG2	1:A:744:PHE:HZ	1.71	0.55
1:A:622:ASP:O	1:A:625:SER:OG	2.20	0.54
1:A:568:LYS:HE2	1:A:598:ASP:OD1	2.06	0.54
1:A:425:LEU:HB2	1:A:432:GLY:HA3	1.89	0.54
2:X:4:A:O2'	2:X:5:A:H5'	2.07	0.54
3:Y:7:C:H2'	3:Y:8:G:C8	2.43	0.53
1:A:619:ARG:HD3	1:A:697:GLU:HB2	1.91	0.53
1:A:929:VAL:HG21	1:A:979:LEU:HB2	1.91	0.53
2:X:11:G:H2'	2:X:12:G:C8	2.44	0.53

Continued on next page...

CONFIDENTIAL VALIDATION REPORT

Continued from previous page...

Atom-1	Atom-2	Interatomic distance (Å)	Clash overlap (Å)
1:A:777:LYS:O	1:A:782:GLU:N	2.42	0.52
1:A:750:LYS:NZ	1:A:782:GLU:O	2.22	0.52
1:A:705:ARG:HG2	1:A:744:PHE:CZ	2.45	0.52
3:Y:4:C:H2'	3:Y:5:C:C6	2.44	0.52
1:A:606:ARG:NH2	3:Y:4:C:OP1	2.43	0.52
1:A:962:CYS:N	1:A:966:GLN:O	2.42	0.52
1:A:509:ASN:OD1	1:A:513:ASN:ND2	2.42	0.51
3:Y:12:U:H2'	3:Y:13:G:O4'	2.10	0.51
3:Y:3:U:O2'	3:Y:4:C:H5'	2.10	0.51
1:A:366:LYS:NZ	1:A:794:GLU:OE1	2.43	0.51
1:A:497:ALA:HB1	1:A:502:GLU:HB2	1.92	0.51
1:A:711:GLN:OE1	1:A:714:ARG:NH2	2.44	0.51
1:A:413:SER:OG	1:A:414:THR:O	2.22	0.51
1:A:926:MET:HE2	2:X:7:C:H1'	1.92	0.51
1:A:544:ASP:O	1:A:699:GLU:HG2	2.12	0.50
1:A:550:PRO:O	1:A:554:LYS:HB2	2.11	0.50
1:A:721:GLY:N	1:A:784:ASN:O	2.44	0.50
1:A:588:LYS:O	1:A:592:LYS:HG3	2.13	0.49
1:A:732:TYR:O	1:A:736:GLN:HG2	2.12	0.49
1:A:755:ILE:O	1:A:771:GLN:NE2	2.46	0.49
3:Y:4:C:H2'	3:Y:5:C:H6	1.76	0.49
1:A:899:ASN:HB2	1:A:1008:ILE:CG2	2.43	0.48
1:A:899:ASN:HB3	1:A:902:LEU:HD13	1.94	0.48
3:Y:5:C:O2'	3:Y:6:U:H5'	2.13	0.48
1:A:366:LYS:NZ	1:A:794:GLU:HB2	2.28	0.48
1:A:927:HIS:HE1	2:X:6:G:H1'	1.79	0.48
1:A:535:GLU:OE1	1:A:535:GLU:N	2.47	0.48
1:A:626:HIS:O	1:A:629:THR:OG1	2.22	0.47
1:A:806:ARG:HH12	1:A:816:MET:HA	1.79	0.47
1:A:942:ARG:NH2	1:A:959:GLU:OE1	2.43	0.47
1:A:790:THR:HG22	1:A:793:GLU:OE2	2.14	0.47
2:X:3:C:O2'	2:X:4:A:H5'	2.15	0.47
1:A:884:LYS:O	1:A:888:VAL:HG23	2.13	0.47
1:A:878:VAL:O	1:A:882:LEU:HD13	2.14	0.47
1:A:962:CYS:HB3	1:A:966:GLN:N	2.29	0.47
1:A:929:VAL:HG21	1:A:979:LEU:HD12	1.97	0.47
3:Y:7:C:H2'	3:Y:8:G:H8	1.79	0.47
1:A:972:MET:O	1:A:979:LEU:HD23	2.14	0.46
2:X:7:C:O2'	2:X:8:C:H5'	2.15	0.46
1:A:394:ASP:HB3	1:A:768:GLN:HG3	1.97	0.46
1:A:544:ASP:OD1	1:A:545:ASP:N	2.49	0.46

Continued on next page...

CONFIDENTIAL VALIDATION REPORT

Continued from previous page...

Atom-1	Atom-2	Interatomic distance (Å)	Clash overlap (Å)
2:X:13:A:H2'	2:X:14:G:C8	2.51	0.46
1:A:712:PHE:HB3	1:A:747:VAL:HG11	1.97	0.46
1:A:749:VAL:CG1	1:A:785:LEU:HD22	2.39	0.45
1:A:879:GLN:O	1:A:883:GLU:HG3	2.16	0.45
3:Y:13:G:H2'	3:Y:14:A:H8	1.81	0.45
3:Y:2:C:O2'	3:Y:3:U:H5'	2.15	0.45
1:A:545:ASP:HA	1:A:699:GLU:OE2	2.17	0.45
1:A:752:HIS:ND1	1:A:753:HIS:O	2.46	0.45
2:X:5:A:H2'	2:X:6:G:H8	1.77	0.45
1:A:832:LEU:HG	1:A:834:THR:HG23	1.99	0.45
2:X:6:G:O2'	2:X:7:C:H5'	2.16	0.45
1:A:753:HIS:HA	1:A:787:ILE:HB	1.99	0.45
3:Y:10:C:H2'	3:Y:11:U:C6	2.52	0.45
3:Y:13:G:H2'	3:Y:14:A:C8	2.51	0.45
1:A:728:ARG:NH2	1:A:756:GLY:O	2.50	0.45
1:A:547:ARG:O	1:A:548:GLU:HB2	2.17	0.45
1:A:750:LYS:O	1:A:785:LEU:HD23	2.16	0.45
1:A:445:GLU:OE1	1:A:447:HIS:NE2	2.48	0.45
2:X:13:A:O2'	2:X:14:G:H5'	2.17	0.44
1:A:307:LEU:O	1:A:307:LEU:HD12	2.17	0.44
1:A:883:GLU:O	1:A:887:LYS:HG2	2.18	0.44
1:A:792:ALA:HB1	1:A:796:LEU:HD21	1.99	0.44
3:Y:4:C:O2'	3:Y:5:C:H5'	2.17	0.44
1:A:742:ALA:O	1:A:746:GLU:HG3	2.18	0.44
1:A:823:ALA:HB3	1:A:828:SER:HB3	2.00	0.44
3:Y:1:U:H2'	3:Y:2:C:H6	1.82	0.44
1:A:778:PHE:CD1	1:A:786:LEU:HG	2.53	0.44
1:A:450:ASN:O	1:A:456:ASN:ND2	2.51	0.43
1:A:509:ASN:O	1:A:513:ASN:ND2	2.51	0.43
1:A:962:CYS:HB2	1:A:968:TRP:NE1	2.33	0.43
2:X:13:A:H2'	2:X:14:G:H8	1.83	0.43
1:A:962:CYS:HB2	1:A:968:TRP:CE2	2.54	0.43
1:A:981:CYS:SG	1:A:982:LEU:N	2.92	0.43
1:A:899:ASN:HB2	1:A:1008:ILE:HG22	2.01	0.43
2:X:7:C:H2'	2:X:8:C:H6	1.84	0.43
3:Y:10:C:H2'	3:Y:11:U:H6	1.83	0.43
1:A:792:ALA:HB1	1:A:796:LEU:CD2	2.48	0.43
1:A:452:GLU:O	2:X:10:A:H4'	2.19	0.42
1:A:685:LYS:O	1:A:689:LYS:HG2	2.18	0.42
1:A:793:GLU:O	1:A:822:ARG:NH2	2.51	0.42
1:A:500:GLN:NE2	1:A:860:ARG:HD2	2.34	0.42

Continued on next page...

CONFIDENTIAL VALIDATION REPORT

Continued from previous page...

Atom-1	Atom-2	Interatomic distance (Å)	Clash overlap (Å)
1:A:307:LEU:HD11	1:A:345:LYS:HZ1	1.84	0.42
1:A:551:PHE:O	1:A:554:LYS:HB3	2.20	0.42
1:A:778:PHE:O	1:A:781:GLY:N	2.51	0.42
1:A:697:GLU:HG3	1:A:698:ASN:N	2.34	0.42
1:A:962:CYS:SG	1:A:964:CYS:N	2.89	0.42
1:A:705:ARG:NH2	1:A:743:LYS:HD2	2.27	0.42
3:Y:6:U:H2'	3:Y:7:C:H6	1.84	0.42
1:A:490:ALA:HB3	1:A:818:GLN:OE1	2.19	0.42
1:A:796:LEU:O	1:A:796:LEU:HD12	2.19	0.42
1:A:371:GLU:OE1	1:A:772:LYS:NZ	2.42	0.42
1:A:749:VAL:HG12	1:A:785:LEU:CD2	2.42	0.42
2:X:2:U:O2'	2:X:3:C:H5'	2.20	0.42
1:A:723:ILE:CD1	1:A:785:LEU:HD12	2.50	0.41
3:Y:6:U:H2'	3:Y:7:C:C6	2.55	0.41
2:X:9:G:O2'	2:X:10:A:H5'	2.20	0.41
1:A:573:ASP:OD1	1:A:574:PHE:N	2.53	0.41
2:X:7:C:H2'	2:X:8:C:C6	2.55	0.41
1:A:741:ASN:OD1	1:A:742:ALA:N	2.54	0.41
1:A:788:ALA:HB1	1:A:792:ALA:HB3	2.02	0.41
1:A:459:MET:O	1:A:463:LEU:HG	2.20	0.41
1:A:641:VAL:HG11	1:A:963:LYS:O	2.21	0.41
1:A:895:GLN:HB3	1:A:1007:PRO:HB3	2.03	0.40
1:A:332:THR:HB	1:A:824:ARG:HH21	1.86	0.40
1:A:921:HIS:HB3	1:A:1013:LEU:HB2	2.04	0.40
1:A:894:LYS:O	1:A:895:GLN:HG3	2.22	0.40

There are no symmetry-related clashes.

5.3 Torsion angles (i)

5.3.1 Protein backbone (i)

In the following table, the Percentiles column shows the percent Ramachandran outliers of the chain as a percentile score with respect to all PDB entries followed by that with respect to all EM entries.

The Analysed column shows the number of residues for which the backbone conformation was analysed, and the total number of residues.

Mol	Chain	Analysed	Favoured	Allowed	Outliers	Percentiles	
1	A	679/1007 (67%)	611 (90%)	68 (10%)	0	100	100

CONFIDENTIAL VALIDATION REPORT

There are no Ramachandran outliers to report.

5.3.2 Protein sidechains (i)

In the following table, the Percentiles column shows the percent sidechain outliers of the chain as a percentile score with respect to all PDB entries followed by that with respect to all EM entries.

The Analysed column shows the number of residues for which the sidechain conformation was analysed, and the total number of residues.

Mol	Chain	Analysed	Rotameric	Outliers	Percentiles	
1	A	616/900 (68%)	613 (100%)	3 (0%)	90	95

All (3) residues with a non-rotameric sidechain are listed below:

Mol	Chain	Res	Type
1	A	595	ASN
1	A	599	ARG
1	A	615	ASN

Some sidechains can be flipped to improve hydrogen bonding and reduce clashes. All (6) such sidechains are listed below:

Mol	Chain	Res	Type
1	A	448	HIS
1	A	595	ASN
1	A	768	GLN
1	A	784	ASN
1	A	899	ASN
1	A	927	HIS

5.3.3 RNA (i)

Mol	Chain	Analysed	Backbone Outliers	Pucker Outliers
2	X	14/15 (93%)	0	0
3	Y	14/15 (93%)	1 (7%)	0
All	All	28/30 (93%)	1 (3%)	0

All (1) RNA backbone outliers are listed below:

Mol	Chain	Res	Type
3	Y	11	U

CONFIDENTIAL VALIDATION REPORT

There are no RNA pucker outliers to report.

5.4 Non-standard residues in protein, DNA, RNA chains (i)

There are no non-standard protein/DNA/RNA residues in this entry.

5.5 Carbohydrates (i)

There are no carbohydrates in this entry.

5.6 Ligand geometry (i)

Of 4 ligands modelled in this entry, 2 are monoatomic - leaving 2 for Mogul analysis.

In the following table, the Counts columns list the number of bonds (or angles) for which Mogul statistics could be retrieved, the number of bonds (or angles) that are observed in the model and the number of bonds (or angles) that are defined in the Chemical Component Dictionary. The Link column lists molecule types, if any, to which the group is linked. The Z score for a bond length (or angle) is the number of standard deviations the observed value is removed from the expected value. A bond length (or angle) with $|Z| > 2$ is considered an outlier worth inspection. RMSZ is the root-mean-square of all Z scores of the bond lengths (or angles).

Mol	Type	Chain	Res	Link	Bond lengths			Bond angles		
					Counts	RMSZ	$\#\|Z\| > 2$	Counts	RMSZ	$\#\|Z\| > 2$
4	ADP	A	1101	6	25,29,29	1.02	2 (8%)	25,45,45	1.89	4 (16%)
5	ALF	A	1102	-	0,4,4	0.00	-	0,6,6	0.00	-

In the following table, the Chirals column lists the number of chiral outliers, the number of chiral centers analysed, the number of these observed in the model and the number defined in the Chemical Component Dictionary. Similar counts are reported in the Torsion and Rings columns. '-' means no outliers of that kind were identified.

Mol	Type	Chain	Res	Link	Chirals	Torsions	Rings
4	ADP	A	1101	6	-	0/12/32/32	0/3/3/3
5	ALF	A	1102	-	-	0/0/0/0	0/0/0/0

All (2) bond length outliers are listed below:

Mol	Chain	Res	Type	Atoms	Z	Observed(Å)	Ideal(Å)
4	A	1101	ADP	C8-N9	-2.07	1.34	1.36
4	A	1101	ADP	C5-C4	2.85	1.46	1.40

All (4) bond angle outliers are listed below:

CONFIDENTIAL VALIDATION REPORT

Mol	Chain	Res	Type	Atoms	Z	Observed(^{o})	Ideal(^{o})
4	A	1101	ADP	N3-C2-N1	-6.61	123.21	128.86
4	A	1101	ADP	PA-O3A-PB	-4.04	119.05	132.63
4	A	1101	ADP	C4-C5-N7	-2.67	106.83	109.41
4	A	1101	ADP	O3B-PB-O2B	2.00	115.51	107.59

There are no chirality outliers.

There are no torsion outliers.

There are no ring outliers.

1 monomer is involved in 1 short contact:

Mol	Chain	Res	Type	Clashes	Symm-Clashes
4	A	1101	ADP	1	0

5.7 Other polymers (i)

There are no such residues in this entry.

5.8 Polymer linkage issues (i)

There are no chain breaks in this entry.

CONFIDENTIAL VALIDATION REPORT

Click here to access/download;PDB Validation Report;D_1200010127_val-report-full-annotate_P1.pdf

Full wwPDB/EMDataBank EM Map/Model Validation Report (i)

May 21, 2018 – 05:11 PM BST

PDB ID	:	6GKM
Title	:	CryoEM structure of the MDA5-dsRNA filament in complex with ATP (10 mM)
Deposited on	:	2018-05-21
Resolution	:	3.87 Å(reported)

This is a Full wwPDB/EMDataBank EM Map/Model Validation Report.

This report is produced by the wwPDB biocuration pipeline after annotation of the structure.

We welcome your comments at *validation@mail.wwpdb.org*
A user guide is available at
https://www.wwpdb.org/validation/2017/EMValidationReportHelp
with specific help available everywhere you see the (i) symbol.

MolProbity	:	4.02b-467
Mogul	:	1.7.3 (157068), CSD as539be (2018)
Percentile statistics	:	20171227.v01 (using entries in the PDB archive December 27th 2017)
Ideal geometry (proteins)	:	Engh & Huber (2001)
Ideal geometry (DNA, RNA)	:	Parkinson et. al. (1996)
Validation Pipeline (wwPDB-VP)	:	rb-20031021

CONFIDENTIAL VALIDATION REPORT

1 Overall quality at a glance (i)

The following experimental techniques were used to determine the structure:
ELECTRON MICROSCOPY

The reported resolution of this entry is 3.87 Å.

Percentile scores (ranging between 0-100) for global validation metrics of the entry are shown in the following graphic. The table shows the number of entries on which the scores are based.

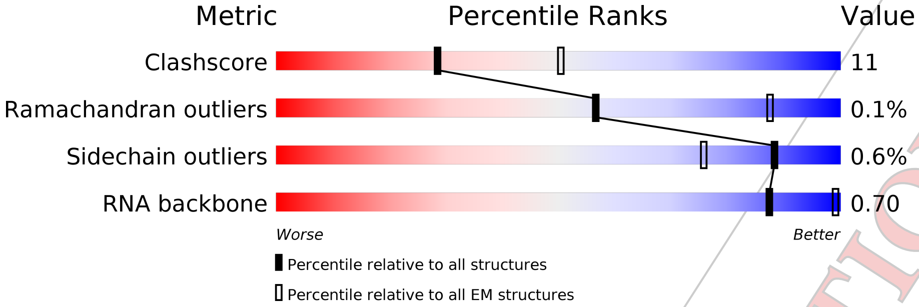

Metric	Whole archive (#Entries)	EM structures (#Entries)
Clashscore	136327	1886
Ramachandran outliers	132723	1663
Sidechain outliers	132532	1531
RNA backbone	3747	458

The table below summarises the geometric issues observed across the polymeric chains. The red, orange, yellow and green segments on the bar indicate the fraction of residues that contain outliers for >=3, 2, 1 and 0 types of geometric quality criteria. A grey segment represents the fraction of residues that are not modelled. The numeric value for each fraction is indicated below the corresponding segment, with a dot representing fractions <=5%

Mol	Chain	Length	Quality of chain
1	A	1007	55% 13% 32%
2	X	14	100%
3	Y	14	7% 86% 7%

CONFIDENTIAL VALIDATION REPORT

2 Entry composition (i)

There are 5 unique types of molecules in this entry. The entry contains 10562 atoms, of which 4402 are hydrogens and 0 are deuteriums.

In the tables below, the AltConf column contains the number of residues with at least one atom in alternate conformation and the Trace column contains the number of residues modelled with at most 2 atoms.

- Molecule 1 is a protein called Interferon-induced helicase C domain-containing protein 1.

Mol	Chain	Residues	Atoms						AltConf	Trace
			Total	C	H	N	O	S		
1	A	689	9933	3502	4402	960	1034	35	0	0

There are 18 discrepancies between the modelled and reference sequences:

Chain	Residue	Modelled	Actual	Comment	Reference
A	?	-	ASP	deletion	UNP Q8R5F7
A	?	-	LYS	deletion	UNP Q8R5F7
A	?	-	SER	deletion	UNP Q8R5F7
A	?	-	ASP	deletion	UNP Q8R5F7
A	?	-	ASP	deletion	UNP Q8R5F7
A	?	-	GLU	deletion	UNP Q8R5F7
A	?	-	ALA	deletion	UNP Q8R5F7
A	?	-	SER	deletion	UNP Q8R5F7
A	?	-	SER	deletion	UNP Q8R5F7
A	?	-	CYS	deletion	UNP Q8R5F7
A	?	-	ASN	deletion	UNP Q8R5F7
A	?	-	ASP	deletion	UNP Q8R5F7
A	?	-	GLN	deletion	UNP Q8R5F7
A	?	-	LEU	deletion	UNP Q8R5F7
A	?	-	LYS	deletion	UNP Q8R5F7
A	?	-	GLY	deletion	UNP Q8R5F7
A	?	-	ASP	deletion	UNP Q8R5F7
A	?	-	VAL	deletion	UNP Q8R5F7

- Molecule 2 is a RNA chain called RNA (5'-R(P*CP*AP*AP*GP*CP*CP*GP*AP*GP*GP *AP*GP*AP*G)-3').

Mol	Chain	Residues	Atoms					AltConf	Trace
			Total	C	N	O	P		
2	X	14	308	137	64	93	14	0	0

- Molecule 3 is a RNA chain called RNA (5'-R(P*CP*UP*CP*UP*CP*CP*UP*CP*GP*GP

CONFIDENTIAL VALIDATION REPORT

*CP*UP*UP*G)-3').

Mol	Chain	Residues	Atoms					AltConf	Trace
			Total	C	N	O	P		
3	Y	14	289	129	43	103	14	0	0

- Molecule 4 is ZINC ION (three-letter code: ZN) (formula: Zn).

Mol	Chain	Residues	Atoms		AltConf
			Total	Zn	
4	A	1	1	1	0

- Molecule 5 is ADENOSINE-5'-TRIPHOSPHATE (three-letter code: ATP) (formula: $C_{10}H_{16}N_5O_{13}P_3$).

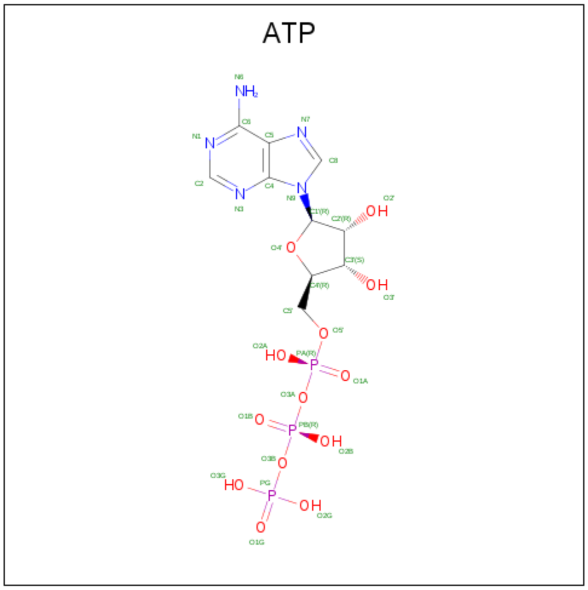

Mol	Chain	Residues	Atoms					AltConf
			Total	C	N	O	P	
5	A	1	31	10	5	13	3	0

CONFIDENTIAL VALIDATION REPORT

3 Residue-property plots (i)

These plots are drawn for all protein, RNA and DNA chains in the entry. The first graphic for a chain summarises the proportions of the various outlier classes displayed in the second graphic. The second graphic shows the sequence view annotated by issues in geometry. Residues are color-coded according to the number of geometric quality criteria for which they contain at least one outlier: green = 0, yellow = 1, orange = 2 and red = 3 or more. Stretches of 2 or more consecutive residues without any outlier are shown as a green connector. Residues present in the sample, but not in the model, are shown in grey.

- Molecule 1: Interferon-induced helicase C domain-containing protein 1

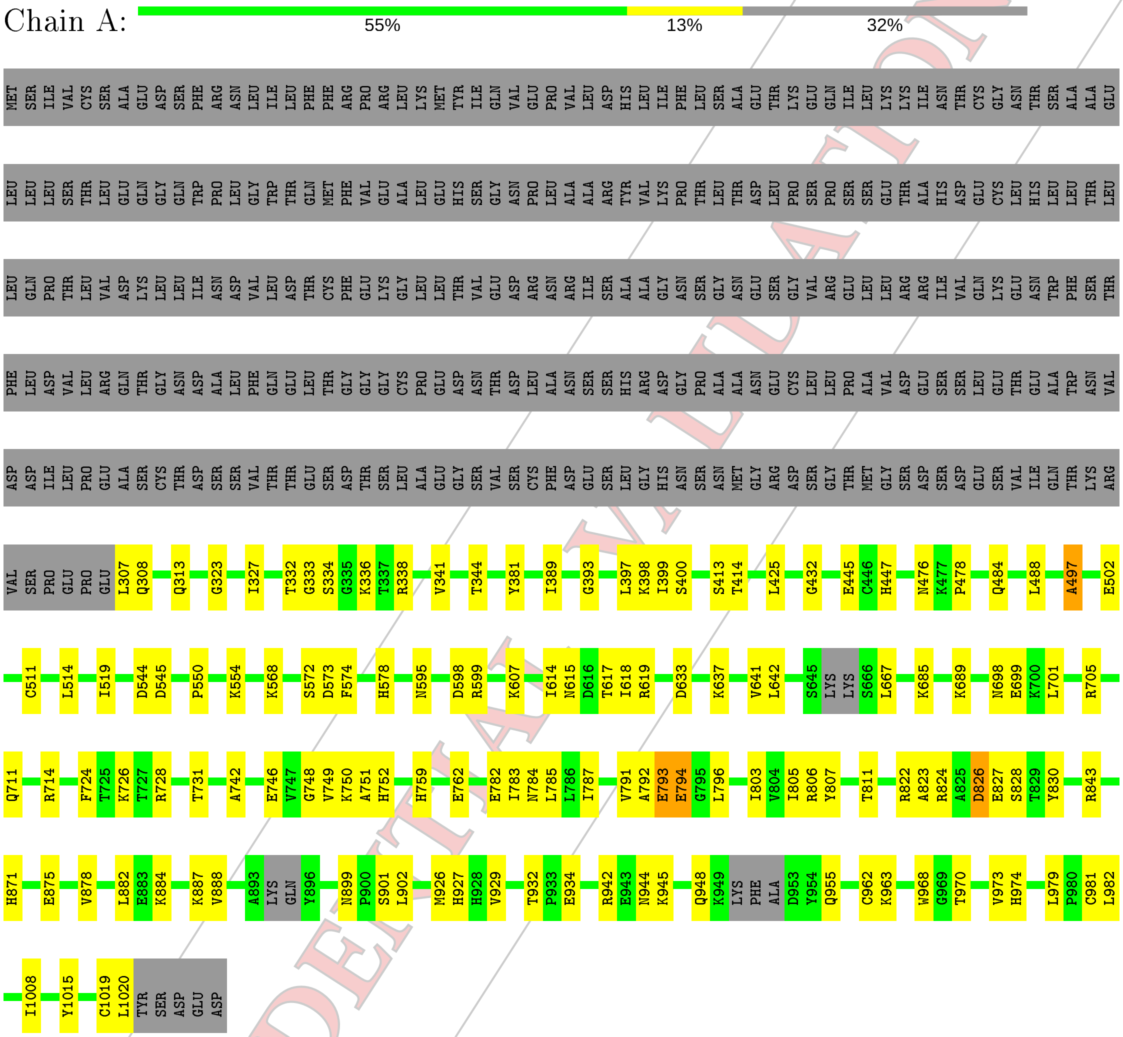

- Molecule 2: RNA (5'-R(P*CP*AP*AP*GP*CP*CP*GP*AP*GP*GP*AP*GP*AP*G)-3')

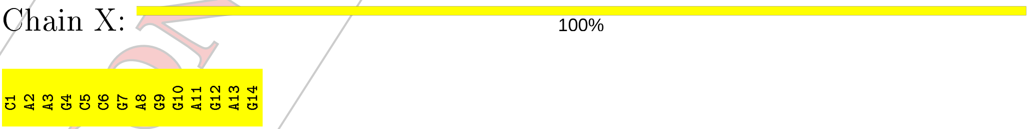

CONFIDENTIAL VALIDATION REPORT

• Molecule 3: RNA (5’-R(P*CP*UP*CP*UP*CP*CP*UP*CP*GP*GP*CP*UP*UP*G)-3’)

Chain Y:
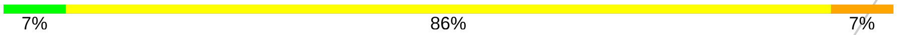

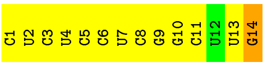

CONFIDENTIAL VALIDATION REPORT

4 Experimental information (i)

Property	Value	Source
Reconstruction method	HELICAL	Depositor
Imposed symmetry	HELICAL, twist=72.8171°, rise=43.0617 Å, axial sym=C1	Depositor
Number of segments used	100482	Depositor
Resolution determination method	FSC 0.143 CUT-OFF	Depositor
CTF correction method	PHASE FLIPPING AND AMPLITUDE CORRECTION	Depositor
Microscope	FEI TITAN KRIOS	Depositor
Voltage (kV)	300	Depositor
Electron dose ($e^-/\text{Å}^2$)	30.24	Depositor
Minimum defocus (nm)	-1700	Depositor
Maximum defocus (nm)	-3100	Depositor
Magnification	75000	Depositor
Image detector	FEI FALCON III (4k x 4k)	Depositor

CONFIDENTIAL VALIDATION REPORT

5 Model quality (i)

5.1 Standard geometry (i)

Bond lengths and bond angles in the following residue types are not validated in this section: ZN, ATP

The Z score for a bond length (or angle) is the number of standard deviations the observed value is removed from the expected value. A bond length (or angle) with $|Z| > 5$ is considered an outlier worth inspection. RMSZ is the root-mean-square of all Z scores of the bond lengths (or angles).

Mol	Chain	Bond lengths		Bond angles	
		RMSZ	$\#\|Z\| > 2$	RMSZ	$\#\|Z\| > 2$
1	A	0.38	0/5618	0.57	3/7550 (0.0%)
2	X	0.51	0/346	0.84	0/539
3	Y	0.53	0/319	0.88	0/493
All	All	0.39	0/6283	0.61	3/8582 (0.0%)

There are no bond length outliers.

All (3) bond angle outliers are listed below:

Mol	Chain	Res	Type	Atoms	Z	Observed(^{o})	Ideal(^{o})
1	A	794	GLU	C-N-CA	8.74	140.65	122.30
1	A	497	ALA	C-N-CA	8.58	143.16	121.70
1	A	793	GLU	C-N-CA	6.97	139.12	121.70

There are no chirality outliers.

There are no planarity outliers.

5.2 Too-close contacts (i)

In the following table, the Non-H and H(model) columns list the number of non-hydrogen atoms and hydrogen atoms in the chain respectively. The H(added) column lists the number of hydrogen atoms added and optimized by MolProbity. The Clashes column lists the number of clashes within the asymmetric unit, whereas Symm-Clashes lists symmetry related clashes.

Mol	Chain	Non-H	H(model)	H(added)	Clashes	Symm-Clashes
1	A	5531	4402	5655	106	0
2	X	308	0	155	25	0
3	Y	289	0	150	23	0
4	A	1	0	0	0	0
5	A	31	0	12	1	0

Continued on next page...

CONFIDENTIAL VALIDATION REPORT

Continued from previous page...

Mol	Chain	Non-H	H(model)	H(added)	Clashes	Symm-Clashes
All	All	6160	4402	5972	137	0

The all-atom clashscore is defined as the number of clashes found per 1000 atoms (including hydrogen atoms). The all-atom clashscore for this structure is 11.

All (137) close contacts within the same asymmetric unit are listed below, sorted by their clash magnitude.

Atom-1	Atom-2	Interatomic distance (Å)	Clash overlap (Å)
1:A:823:ALA:HB1	1:A:828:SER:HB2	1.62	0.82
1:A:476:ASN:HB3	1:A:478:PRO:HD2	1.61	0.82
1:A:899:ASN:HB3	1:A:902:LEU:HD13	1.62	0.80
1:A:642:LEU:HD22	1:A:667:LEU:HD22	1.68	0.73
1:A:336:LYS:NZ	5:A:1102:ATP:O3G	2.22	0.72
1:A:724:PHE:HE1	1:A:792:ALA:HB3	1.56	0.71
1:A:751:ALA:HB2	1:A:785:LEU:HD21	1.72	0.71
1:A:393:GLY:HA2	1:A:398:LYS:HD3	1.72	0.71
1:A:748:GLY:O	1:A:784:ASN:ND2	2.22	0.71
1:A:970:THR:HG21	2:X:3:A:H4’	1.74	0.68
1:A:843:ARG:CZ	2:X:12:G:H5’	2.22	0.68
1:A:843:ARG:NH2	2:X:11:A:O3’	2.26	0.67
1:A:1019:CYS:SG	1:A:1020:LEU:N	2.66	0.67
1:A:962:CYS:SG	1:A:963:LYS:N	2.69	0.66
1:A:607:LYS:NZ	1:A:762:GLU:OE2	2.31	0.63
1:A:792:ALA:O	1:A:822:ARG:NH2	2.32	0.62
1:A:728:ARG:O	1:A:731:THR:OG1	2.13	0.62
1:A:929:VAL:HG21	1:A:979:LEU:HD12	1.80	0.62
3:Y:13:U:H2’	3:Y:14:G:O4’	2.00	0.62
1:A:413:SER:OG	1:A:414:THR:O	2.14	0.62
3:Y:3:C:H2’	3:Y:4:U:H6	1.65	0.61
1:A:871:HIS:NE2	1:A:875:GLU:OE2	2.33	0.61
1:A:974:HIS:HB3	1:A:979:LEU:HD21	1.83	0.61
3:Y:2:U:H2’	3:Y:3:C:C6	2.36	0.60
1:A:701:LEU:O	1:A:705:ARG:HG3	2.01	0.60
1:A:927:HIS:CE1	2:X:4:G:H1’	2.37	0.60
1:A:884:LYS:O	1:A:888:VAL:HG23	2.02	0.60
1:A:341:VAL:O	1:A:344:THR:OG1	2.17	0.60
1:A:932:THR:OG1	1:A:934:GLU:OE1	2.20	0.59
1:A:642:LEU:CD2	1:A:667:LEU:HD22	2.33	0.59
1:A:323:GLY:O	1:A:484:GLN:NE2	2.36	0.59
3:Y:3:C:H2’	3:Y:4:U:C6	2.38	0.58
1:A:899:ASN:HB2	1:A:1008:ILE:HG21	1.85	0.58

Continued on next page...

CONFIDENTIAL VALIDATION REPORT

Continued from previous page...

Atom-1	Atom-2	Interatomic distance (Å)	Clash overlap (Å)
1:A:981:CYS:SG	1:A:982:LEU:N	2.77	0.58
2:X:1:C:H2'	2:X:2:A:H8	1.69	0.57
1:A:633:ASP:OD1	1:A:942:ARG:NH2	2.35	0.57
1:A:578:HIS:ND1	2:X:14:G:H4'	2.20	0.56
1:A:926:MET:CE	2:X:5:C:H1'	2.35	0.56
1:A:927:HIS:HE1	2:X:4:G:H1'	1.71	0.56
2:X:11:A:H2'	2:X:12:G:C8	2.40	0.56
3:Y:7:U:O2'	3:Y:8:C:H5'	2.06	0.56
2:X:1:C:H2'	2:X:2:A:C8	2.42	0.55
1:A:843:ARG:NH2	2:X:12:G:H5'	2.22	0.55
3:Y:4:U:H2'	3:Y:5:C:H6	1.71	0.55
1:A:724:PHE:CE1	1:A:792:ALA:HB3	2.40	0.54
1:A:791:VAL:HG23	3:Y:7:U:H5"	1.88	0.54
3:Y:1:C:H2'	3:Y:2:U:C6	2.42	0.54
3:Y:9:G:H2'	3:Y:10:G:C8	2.43	0.54
3:Y:1:C:H2'	3:Y:2:U:H6	1.73	0.54
1:A:742:ALA:O	1:A:746:GLU:HG3	2.08	0.53
2:X:11:A:H2'	2:X:12:G:H8	1.73	0.53
1:A:962:CYS:HB2	1:A:968:TRP:CE2	2.43	0.53
1:A:750:LYS:H	1:A:784:ASN:HD22	1.56	0.53
1:A:750:LYS:O	1:A:784:ASN:N	2.42	0.52
1:A:752:HIS:HB2	1:A:783:ILE:HG12	1.91	0.52
1:A:791:VAL:CG2	3:Y:7:U:H5"	2.38	0.52
1:A:979:LEU:HD23	1:A:979:LEU:H	1.73	0.52
3:Y:8:C:H2'	3:Y:9:G:H8	1.74	0.52
1:A:1015:TYR:O	1:A:1019:CYS:HB3	2.10	0.52
1:A:637:LYS:O	1:A:641:VAL:HG23	2.11	0.51
1:A:728:ARG:NH2	3:Y:7:U:OP2	2.40	0.51
1:A:899:ASN:HB2	1:A:1008:ILE:CG2	2.40	0.51
1:A:425:LEU:HB2	1:A:432:GLY:HA3	1.92	0.51
1:A:568:LYS:HE2	1:A:598:ASP:OD1	2.11	0.51
1:A:544:ASP:OD1	1:A:545:ASP:N	2.43	0.51
3:Y:8:C:H2'	3:Y:9:G:C8	2.46	0.51
1:A:445:GLU:OE1	1:A:447:HIS:NE2	2.43	0.50
2:X:4:G:O2'	2:X:5:C:H5'	2.12	0.50
1:A:307:LEU:HG	1:A:308:GLN:HG2	1.92	0.50
1:A:399:ILE:HG13	1:A:400:SER:N	2.27	0.49
1:A:806:ARG:NH1	1:A:830:TYR:OH	2.46	0.49
1:A:962:CYS:HB2	1:A:968:TRP:NE1	2.28	0.49
1:A:749:VAL:HG13	1:A:785:LEU:HD22	1.94	0.49
3:Y:4:U:H2'	3:Y:5:C:C6	2.47	0.49

Continued on next page...

CONFIDENTIAL VALIDATION REPORT

Continued from previous page...

Atom-1	Atom-2	Interatomic distance (Å)	Clash overlap (Å)
1:A:685:LYS:O	1:A:689:LYS:HG2	2.13	0.48
2:X:5:C:O2’	2:X:6:C:H5’	2.14	0.48
1:A:750:LYS:HD2	1:A:782:GLU:O	2.13	0.48
2:X:8:A:H2’	2:X:9:G:H8	1.78	0.48
1:A:332:THR:O	1:A:824:ARG:NH2	2.46	0.48
1:A:803:ILE:O	1:A:805:ILE:HG23	2.13	0.48
2:X:10:G:H2’	2:X:11:A:H8	1.79	0.48
1:A:389:ILE:HD11	1:A:397:LEU:HD13	1.96	0.47
1:A:824:ARG:N	1:A:824:ARG:HD2	2.28	0.47
1:A:901:SER:C	1:A:902:LEU:HD12	2.34	0.47
1:A:336:LYS:HG2	1:A:488:LEU:HD23	1.95	0.47
3:Y:2:U:H2’	3:Y:3:C:H6	1.76	0.47
1:A:944:ASN:O	1:A:948:GLN:HB2	2.14	0.47
3:Y:10:G:O2’	3:Y:11:C:H5’	2.14	0.47
1:A:573:ASP:OD1	1:A:574:PHE:N	2.48	0.47
1:A:393:GLY:HA2	1:A:398:LYS:CD	2.43	0.46
1:A:327:ILE:HD11	1:A:514:LEU:HD12	1.97	0.46
1:A:572:SER:OG	1:A:573:ASP:N	2.45	0.46
1:A:497:ALA:HB1	1:A:502:GLU:HB2	1.96	0.46
2:X:7:G:O2’	2:X:8:A:H5’	2.16	0.46
1:A:336:LYS:CG	1:A:488:LEU:HD23	2.47	0.45
2:X:8:A:H2’	2:X:9:G:C8	2.52	0.45
1:A:399:ILE:HG13	1:A:400:SER:H	1.82	0.45
1:A:926:MET:HE3	2:X:5:C:H1’	1.98	0.45
1:A:974:HIS:CB	1:A:979:LEU:HD21	2.46	0.44
1:A:731:THR:HG22	1:A:787:ILE:HG22	2.00	0.44
1:A:929:VAL:HG21	1:A:979:LEU:CD1	2.45	0.44
1:A:811:THR:O	2:X:11:A:H4’	2.17	0.44
2:X:13:A:H2’	2:X:14:G:C8	2.52	0.44
2:X:5:C:H2’	2:X:6:C:C6	2.52	0.44
1:A:826:ASP:OD1	1:A:827:GLU:N	2.46	0.44
1:A:711:GLN:OE1	1:A:714:ARG:NH2	2.51	0.44
1:A:511:CYS:SG	1:A:519:ILE:HD11	2.58	0.43
1:A:884:LYS:O	1:A:887:LYS:HB2	2.17	0.43
1:A:544:ASP:O	1:A:699:GLU:HG2	2.17	0.43
1:A:617:THR:O	1:A:807:TYR:OH	2.35	0.43
1:A:826:ASP:O	1:A:827:GLU:HB2	2.18	0.43
1:A:698:ASN:OD1	1:A:699:GLU:N	2.52	0.43
1:A:944:ASN:H	1:A:955:GLN:HG2	1.84	0.43
1:A:338:ARG:NE	1:A:381:TYR:HE2	2.16	0.43
1:A:878:VAL:O	1:A:882:LEU:HD13	2.19	0.43

Continued on next page...

CONFIDENTIAL VALIDATION REPORT

Continued from previous page...

Atom-1	Atom-2	Interatomic distance (Å)	Clash overlap (Å)
1:A:973:VAL:O	3:Y:14:G:H4'	2.19	0.43
1:A:550:PRO:O	1:A:554:LYS:HB2	2.18	0.43
2:X:10:G:H2'	2:X:11:A:C8	2.53	0.43
3:Y:10:G:C2'	3:Y:11:C:H5'	2.49	0.42
1:A:796:LEU:O	1:A:796:LEU:HD12	2.19	0.42
1:A:711:GLN:HE22	1:A:714:ARG:HH21	1.67	0.42
1:A:750:LYS:O	1:A:785:LEU:HD23	2.19	0.42
1:A:614:ILE:O	1:A:618:ILE:N	2.43	0.42
1:A:726:LYS:NZ	3:Y:5:C:O2'	2.50	0.42
1:A:974:HIS:HB3	1:A:979:LEU:CD2	2.48	0.42
1:A:714:ARG:NH2	1:A:827:GLU:O	2.52	0.42
1:A:333:GLY:HA3	1:A:824:ARG:HH12	1.85	0.41
1:A:313:GLN:NE2	1:A:334:SER:O	2.49	0.41
3:Y:5:C:O2'	3:Y:6:C:H5'	2.21	0.41
1:A:793:GLU:O	1:A:794:GLU:HG3	2.19	0.41
1:A:759:HIS:CE1	2:X:5:C:H41	2.38	0.41
1:A:714:ARG:NH2	1:A:827:GLU:HB3	2.36	0.41
1:A:618:ILE:HG22	1:A:619:ARG:N	2.35	0.41
3:Y:6:C:O2'	3:Y:7:U:H5'	2.20	0.40
3:Y:9:G:H2'	3:Y:10:G:H8	1.86	0.40
1:A:393:GLY:HA2	1:A:398:LYS:CE	2.51	0.40
1:A:926:MET:HE2	2:X:5:C:H1'	2.02	0.40

There are no symmetry-related clashes.

5.3 Torsion angles (i)

5.3.1 Protein backbone (i)

In the following table, the Percentiles column shows the percent Ramachandran outliers of the chain as a percentile score with respect to all PDB entries followed by that with respect to all EM entries.

The Analysed column shows the number of residues for which the backbone conformation was analysed, and the total number of residues.

Mol	Chain	Analysed	Favoured	Allowed	Outliers	Percentiles	
1	A	681/1007 (68%)	614 (90%)	66 (10%)	1 (0%)	53	86

All (1) Ramachandran outliers are listed below:

CONFIDENTIAL VALIDATION REPORT

Mol	Chain	Res	Type
1	A	826	ASP

5.3.2 Protein sidechains (i)

In the following table, the Percentiles column shows the percent sidechain outliers of the chain as a percentile score with respect to all PDB entries followed by that with respect to all EM entries.

The Analysed column shows the number of residues for which the sidechain conformation was analysed, and the total number of residues.

Mol	Chain	Analysed	Rotameric	Outliers	Percentiles	
1	A	617/900 (69%)	613 (99%)	4 (1%)	87	94

All (4) residues with a non-rotameric sidechain are listed below:

Mol	Chain	Res	Type
1	A	595	ASN
1	A	599	ARG
1	A	615	ASN
1	A	945	LYS

Some sidechains can be flipped to improve hydrogen bonding and reduce clashes. All (5) such sidechains are listed below:

Mol	Chain	Res	Type
1	A	420	ASN
1	A	595	ASN
1	A	678	ASN
1	A	784	ASN
1	A	957	ASN

5.3.3 RNA (i)

Mol	Chain	Analysed	Backbone Outliers	Pucker Outliers
2	X	13/14 (92%)	0	0
3	Y	13/14 (92%)	1 (7%)	0
All	All	26/28 (92%)	1 (3%)	0

All (1) RNA backbone outliers are listed below:

CONFIDENTIAL VALIDATION REPORT

Mol	Chain	Res	Type
3	Y	14	G

There are no RNA pucker outliers to report.

5.4 Non-standard residues in protein, DNA, RNA chains (i)

There are no non-standard protein/DNA/RNA residues in this entry.

5.5 Carbohydrates (i)

There are no carbohydrates in this entry.

5.6 Ligand geometry (i)

Of 2 ligands modelled in this entry, 1 is monoatomic - leaving 1 for Mogul analysis.

In the following table, the Counts columns list the number of bonds (or angles) for which Mogul statistics could be retrieved, the number of bonds (or angles) that are observed in the model and the number of bonds (or angles) that are defined in the Chemical Component Dictionary. The Link column lists molecule types, if any, to which the group is linked. The Z score for a bond length (or angle) is the number of standard deviations the observed value is removed from the expected value. A bond length (or angle) with $|Z| > 2$ is considered an outlier worth inspection. RMSZ is the root-mean-square of all Z scores of the bond lengths (or angles).

Mol	Type	Chain	Res	Link	Bond lengths			Bond angles		
					Counts	RMSZ	$\#\|Z\| > 2$	Counts	RMSZ	$\#\|Z\| > 2$
5	ATP	A	1102	-	27,33,33	0.97	1 (3%)	27,52,52	2.02	4 (14%)

In the following table, the Chirals column lists the number of chiral outliers, the number of chiral centers analysed, the number of these observed in the model and the number defined in the Chemical Component Dictionary. Similar counts are reported in the Torsion and Rings columns. '-' means no outliers of that kind were identified.

Mol	Type	Chain	Res	Link	Chirals	Torsions	Rings
5	ATP	A	1102	-	-	0/18/38/38	0/3/3/3

All (1) bond length outliers are listed below:

Mol	Chain	Res	Type	Atoms	Z	Observed(Å)	Ideal(Å)
5	A	1102	ATP	C5-C4	2.74	1.46	1.40

CONFIDENTIAL VALIDATION REPORT

All (4) bond angle outliers are listed below:

Mol	Chain	Res	Type	Atoms	Z	Observed(°)	Ideal(°)
5	A	1102	ATP	N3-C2-N1	-6.72	123.12	128.86
5	A	1102	ATP	PA-O3A-PB	-4.09	118.88	132.63
5	A	1102	ATP	PB-O3B-PG	-3.55	120.71	132.63
5	A	1102	ATP	C4-C5-N7	-2.68	106.82	109.41

There are no chirality outliers.

There are no torsion outliers.

There are no ring outliers.

1 monomer is involved in 1 short contact:

Mol	Chain	Res	Type	Clashes	Symm-Clashes
5	A	1102	ATP	1	0

5.7 Other polymers (i)

There are no such residues in this entry.

5.8 Polymer linkage issues (i)

There are no chain breaks in this entry.

CONFIDENTIAL VALIDATION REPORT

Click here to access/download;PDB Validation Report;D_1200011086_val-report-full_P1_6h61.pdf

Full wwPDB/EMDataBank EM Map/Model Validation Report (i)

Jul 30, 2018 – 03:26 PM BST

PDB ID	:	6H61
EMDB ID:	:	4338
Title	:	CryoEM structure of the MDA5-dsRNA filament with 89 degree twist and without nucleotide
Deposited on	:	2018-07-25
Resolution	:	4.02 Å(reported)
Based on PDB ID	:	6G1S

This is a Full wwPDB/EMDataBank EM Map/Model Validation Report.

This report is produced by the wwPDB biocuration pipeline after annotation of the structure.

We welcome your comments at *validation@mail.wwpdb.org*
A user guide is available at
https://www.wwpdb.org/validation/2017/EMValidationReportHelp
with specific help available everywhere you see the (i) symbol.

MolProbity	:	4.02b-467
Percentile statistics	:	20171227.v01 (using entries in the PDB archive December 27th 2017)
Ideal geometry (proteins)	:	Engh & Huber (2001)
Ideal geometry (DNA, RNA)	:	Parkinson et. al. (1996)
Validation Pipeline (wwPDB-VP)	:	rb-20031172

CONFIDENTIAL VALIDATION REPORT

1 Overall quality at a glance ⓘ

The following experimental techniques were used to determine the structure:
ELECTRON MICROSCOPY

The reported resolution of this entry is 4.02 Å.

Percentile scores (ranging between 0-100) for global validation metrics of the entry are shown in the following graphic. The table shows the number of entries on which the scores are based.

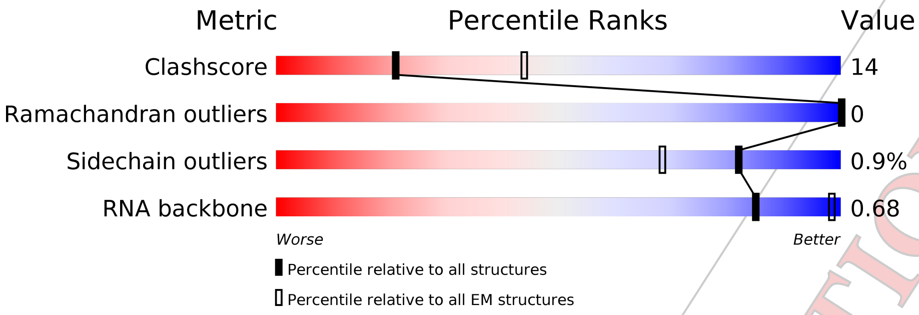

Metric	Whole archive (#Entries)	EM structures (#Entries)
Clashscore	136327	1886
Ramachandran outliers	132723	1663
Sidechain outliers	132532	1531
RNA backbone	3747	458

The table below summarises the geometric issues observed across the polymeric chains. The red, orange, yellow and green segments on the bar indicate the fraction of residues that contain outliers for >=3, 2, 1 and 0 types of geometric quality criteria. A grey segment represents the fraction of residues that are not modelled. The numeric value for each fraction is indicated below the corresponding segment, with a dot representing fractions <=5%

Mol	Chain	Length	Quality of chain
1	A	1007	49% 16% 34%
2	X	15	93% 7%
3	Y	15	7% 93%

CONFIDENTIAL VALIDATION REPORT

2 Entry composition (i)

There are 4 unique types of molecules in this entry. The entry contains 9912 atoms, of which 4057 are hydrogens and 0 are deuteriums.

In the tables below, the AltConf column contains the number of residues with at least one atom in alternate conformation and the Trace column contains the number of residues modelled with at most 2 atoms.

- Molecule 1 is a protein called Interferon-induced helicase C domain-containing protein 1.

Mol	Chain	Residues	Atoms						AltConf	Trace
			Total	C	H	N	O	S		
1	A	660	9272	3312	4057	911	959	33	0	0

There are 18 discrepancies between the modelled and reference sequences:

Chain	Residue	Modelled	Actual	Comment	Reference
A	?	-	ASP	deletion	UNP Q8R5F7
A	?	-	LYS	deletion	UNP Q8R5F7
A	?	-	SER	deletion	UNP Q8R5F7
A	?	-	ASP	deletion	UNP Q8R5F7
A	?	-	ASP	deletion	UNP Q8R5F7
A	?	-	GLU	deletion	UNP Q8R5F7
A	?	-	ALA	deletion	UNP Q8R5F7
A	?	-	SER	deletion	UNP Q8R5F7
A	?	-	SER	deletion	UNP Q8R5F7
A	?	-	CYS	deletion	UNP Q8R5F7
A	?	-	ASN	deletion	UNP Q8R5F7
A	?	-	ASP	deletion	UNP Q8R5F7
A	?	-	GLN	deletion	UNP Q8R5F7
A	?	-	LEU	deletion	UNP Q8R5F7
A	?	-	LYS	deletion	UNP Q8R5F7
A	?	-	GLY	deletion	UNP Q8R5F7
A	?	-	ASP	deletion	UNP Q8R5F7
A	?	-	VAL	deletion	UNP Q8R5F7

- Molecule 2 is a RNA chain called RNA (5'-R(P*UP*CP*CP*AP*UP*GP*CP*GP*CP*AP*UP*GP*AP*CP*G)-3').

Mol	Chain	Residues	Atoms					AltConf	Trace
			Total	C	N	O	P		
2	X	15	318	142	56	105	15	0	0

- Molecule 3 is a RNA chain called RNA (5'-R(P*CP*GP*UP*CP*AP*UP*GP*CP*GP*CP

CONFIDENTIAL VALIDATION REPORT

*AP*UP*GP*GP*A)-3').

Mol	Chain	Residues	Atoms					AltConf	Trace
			Total	C	N	O	P		
3	Y	15	321	143	58	105	15	0	0

- Molecule 4 is ZINC ION (three-letter code: ZN) (formula: Zn).

Mol	Chain	Residues	Atoms		AltConf
			Total	Zn	
4	A	1	1	1	0

CONFIDENTIAL VALIDATION REPORT

3 Residue-property plots ⓘ

These plots are drawn for all protein, RNA and DNA chains in the entry. The first graphic for a chain summarises the proportions of the various outlier classes displayed in the second graphic. The second graphic shows the sequence view annotated by issues in geometry. Residues are color-coded according to the number of geometric quality criteria for which they contain at least one outlier: green = 0, yellow = 1, orange = 2 and red = 3 or more. Stretches of 2 or more consecutive residues without any outlier are shown as a green connector. Residues present in the sample, but not in the model, are shown in grey.

• Molecule 1: Interferon-induced helicase C domain-containing protein 1

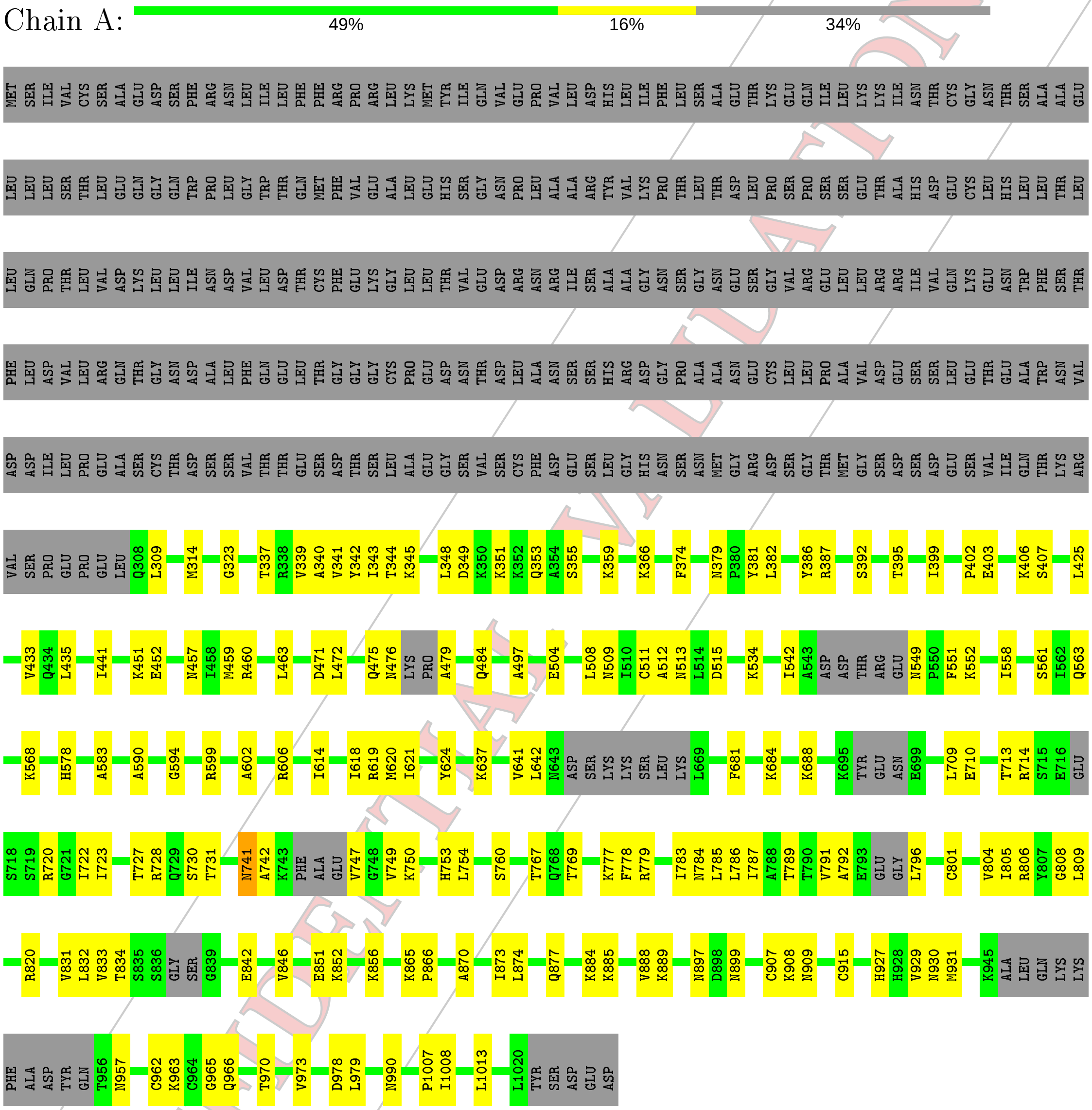

• Molecule 2: RNA (5'-R(P*UP*CP*CP*AP*UP*GP*CP*GP*CP*AP*UP*GP*AP*CP*G)-3')

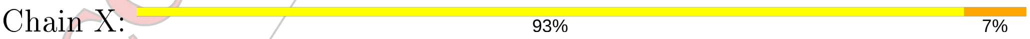

CONFIDENTIAL VALIDATION REPORT

U1 C2 C3 A4 U5 G6 C7 G8 C9 A10 U11 G12 A13 C14 G15

• Molecule 3: RNA (5'-R(P*CP*GP*UP*CP*AP*UP*GP*CP*GP*CP*AP*UP*GP*GP*A)-3')

Chain Y: 7% 93%

C1 G2 U3 C4 A5 U6 G7 C8 G9 C10 A11 U12 G13 G14 A15

CONFIDENTIAL VALIDATION REPORT

4 Experimental information (i)

Property	Value	Source
Reconstruction method	HELICAL	Depositor
Imposed symmetry	HELICAL, twist=89.000°, rise=44.2416 Å, axial sym=C1	Depositor
Number of segments used	26527	Depositor
Resolution determination method	FSC 0.143 CUT-OFF	Depositor
CTF correction method	PHASE FLIPPING AND AMPLITUDE CORRECTION	Depositor
Microscope	FEI TITAN KRIOS	Depositor
Voltage (kV)	300	Depositor
Electron dose ($e^-/\text{Å}^2$)	27.0	Depositor
Minimum defocus (nm)	-1800	Depositor
Maximum defocus (nm)	-2700	Depositor
Magnification	75000	Depositor
Image detector	FEI FALCON III (4k x 4k)	Depositor

CONFIDENTIAL VALIDATION REPORT

5 Model quality (i)

5.1 Standard geometry (i)

Bond lengths and bond angles in the following residue types are not validated in this section: ZN

The Z score for a bond length (or angle) is the number of standard deviations the observed value is removed from the expected value. A bond length (or angle) with $|Z| > 5$ is considered an outlier worth inspection. RMSZ is the root-mean-square of all Z scores of the bond lengths (or angles).

Mol	Chain	Bond lengths		Bond angles	
		RMSZ	#$|Z|$ >2	RMSZ	#$|Z|$ >2
1	A	0.37	0/5293	0.53	1/7121 (0.0%)
2	X	0.58	0/354	0.87	0/549
3	Y	0.61	0/358	0.92	0/556
All	All	0.40	0/6005	0.60	1/8226 (0.0%)

Chiral center outliers are detected by calculating the chiral volume of a chiral center and verifying if the center is modelled as a planar moiety or with the opposite hand.A planarity outlier is detected by checking planarity of atoms in a peptide group, atoms in a mainchain group or atoms of a sidechain that are expected to be planar.

Mol	Chain	#Chirality outliers	#Planarity outliers
1	A	0	1

There are no bond length outliers.

All (1) bond angle outliers are listed below:

Mol	Chain	Res	Type	Atoms	Z	Observed(^{o})	Ideal(^{o})
1	A	497	ALA	C-N-CA	8.18	142.14	121.70

There are no chirality outliers.

All (1) planarity outliers are listed below:

Mol	Chain	Res	Type	Group
1	A	760	SER	Peptide

5.2 Too-close contacts (i)

In the following table, the Non-H and H(model) columns list the number of non-hydrogen atoms and hydrogen atoms in the chain respectively. The H(added) column lists the number of hydrogen

CONFIDENTIAL VALIDATION REPORT

atoms added and optimized by MolProbity. The Clashes column lists the number of clashes within the asymmetric unit, whereas Symm-Clashes lists symmetry related clashes.

Mol	Chain	Non-H	H(model)	H(added)	Clashes	Symm-Clashes
1	A	5215	4057	5282	117	0
2	X	318	0	163	34	0
3	Y	321	0	163	23	0
4	A	1	0	0	0	0
All	All	5855	4057	5608	164	0

The all-atom clashscore is defined as the number of clashes found per 1000 atoms (including hydrogen atoms). The all-atom clashscore for this structure is 14.

All (164) close contacts within the same asymmetric unit are listed below, sorted by their clash magnitude.

Atom-1	Atom-2	Interatomic distance (Å)	Clash overlap (Å)
1:A:720:ARG:NH1	1:A:778:PHE:O	2.16	0.78
1:A:374:PHE:O	1:A:379:ASN:ND2	2.19	0.75
1:A:323:GLY:O	1:A:484:GLN:NE2	2.21	0.74
1:A:750:LYS:H	1:A:784:ASN:HD22	1.36	0.74
1:A:359:LYS:NZ	1:A:407:SER:O	2.21	0.73
1:A:806:ARG:HD2	1:A:809:LEU:HD23	1.70	0.72
1:A:962:CYS:SG	1:A:963:LYS:N	2.63	0.71
3:Y:1:C:H2’	3:Y:2:G:C8	2.28	0.69
1:A:472:LEU:O	1:A:476:ASN:N	2.26	0.69
2:X:3:C:H2’	2:X:4:A:C8	2.29	0.68
1:A:727:THR:O	1:A:730:SER:OG	2.11	0.68
1:A:599:ARG:NH1	1:A:909:ASN:OD1	2.25	0.68
3:Y:3:U:H2’	3:Y:4:C:C6	2.29	0.68
1:A:909:ASN:ND2	1:A:966:GLN:OE1	2.27	0.68
3:Y:1:C:H2’	3:Y:2:G:H8	1.60	0.67
1:A:723:ILE:HD11	1:A:785:LEU:HD21	1.75	0.67
1:A:341:VAL:O	1:A:344:THR:OG1	2.13	0.67
2:X:5:U:H2’	2:X:6:G:H8	1.60	0.66
3:Y:4:C:H2’	3:Y:5:A:C8	2.31	0.65
1:A:351:LYS:O	1:A:355:SER:N	2.29	0.65
1:A:425:LEU:HB2	1:A:433:VAL:H	1.61	0.64
1:A:590:ALA:HB3	1:A:599:ARG:HB3	1.79	0.64
1:A:624:TYR:CZ	1:A:688:LYS:HB2	2.33	0.64
2:X:2:C:H2’	2:X:3:C:H6	1.64	0.63
1:A:747:VAL:HG12	1:A:749:VAL:HG23	1.81	0.63
1:A:563:GLN:NE2	1:A:568:LYS:O	2.32	0.62
2:X:9:C:H2’	2:X:10:A:C8	2.33	0.62

Continued on next page...

CONFIDENTIAL VALIDATION REPORT

Continued from previous page...

Atom-1	Atom-2	Interatomic distance (Å)	Clash overlap (Å)
1:A:753:HIS:HA	1:A:787:ILE:HB	1.81	0.62
3:Y:2:G:O2’	3:Y:3:U:H5’	1.98	0.62
2:X:5:U:H2’	2:X:6:G:C8	2.35	0.62
1:A:399:ILE:HD12	1:A:403:GLU:HB2	1.80	0.62
2:X:1:U:H2’	2:X:2:C:C6	2.35	0.62
1:A:885:LYS:O	1:A:889:LYS:HG2	2.00	0.61
1:A:471:ASP:O	1:A:475:GLN:HB2	1.99	0.61
1:A:509:ASN:O	1:A:513:ASN:ND2	2.34	0.61
2:X:11:U:H2’	2:X:12:G:C8	2.35	0.61
1:A:927:HIS:NE2	2:X:5:U:O2’	2.28	0.60
2:X:3:C:H2’	2:X:4:A:H8	1.64	0.60
1:A:558:ILE:O	1:A:561:SER:OG	2.14	0.60
1:A:710:GLU:O	1:A:714:ARG:HG3	2.01	0.60
1:A:791:VAL:CG2	3:Y:7:G:H5”	2.31	0.60
2:X:9:C:H2’	2:X:10:A:H8	1.66	0.60
1:A:962:CYS:HB3	1:A:966:GLN:H	1.66	0.60
2:X:11:U:H2’	2:X:12:G:H8	1.66	0.59
1:A:399:ILE:HD12	1:A:403:GLU:CB	2.32	0.59
1:A:457:ASN:OD1	1:A:460:ARG:NH2	2.36	0.59
1:A:957:ASN:HB3	1:A:970:THR:CG2	2.31	0.59
1:A:870:ALA:HA	1:A:873:ILE:HG12	1.84	0.59
1:A:459:MET:O	1:A:463:LEU:HG	2.03	0.59
1:A:309:LEU:HD21	1:A:342:TYR:CD2	2.38	0.59
3:Y:9:G:H2’	3:Y:10:C:C6	2.38	0.59
2:X:1:U:H2’	2:X:2:C:H6	1.69	0.58
3:Y:8:C:H2’	3:Y:9:G:C8	2.38	0.58
1:A:842:GLU:O	1:A:846:VAL:HG23	2.04	0.58
1:A:339:VAL:O	1:A:343:ILE:HD12	2.04	0.58
1:A:309:LEU:HD21	1:A:342:TYR:CG	2.37	0.57
1:A:778:PHE:HB2	1:A:786:LEU:HD21	1.86	0.57
2:X:7:C:H2’	2:X:8:G:C8	2.39	0.57
1:A:614:ILE:O	1:A:618:ILE:N	2.37	0.57
3:Y:4:C:H2’	3:Y:5:A:H8	1.70	0.56
3:Y:6:U:H2’	3:Y:7:G:H8	1.70	0.56
1:A:435:LEU:HD12	1:A:441:ILE:HD11	1.88	0.56
1:A:619:ARG:HG3	1:A:621:ILE:HG22	1.86	0.56
2:X:10:A:O2’	2:X:11:U:H5’	2.06	0.56
1:A:865:LYS:HD2	1:A:866:PRO:HD2	1.87	0.56
1:A:511:CYS:O	1:A:515:ASP:N	2.39	0.55
2:X:7:C:H2’	2:X:8:G:H8	1.71	0.55
1:A:806:ARG:HB2	1:A:832:LEU:HD12	1.88	0.55

Continued on next page...

CONFIDENTIAL VALIDATION REPORT

Continued from previous page...

Atom-1	Atom-2	Interatomic distance (Å)	Clash overlap (Å)
3:Y:6:U:H2'	3:Y:7:G:C8	2.42	0.55
2:X:4:A:O2'	2:X:5:U:H5'	2.07	0.55
1:A:808:GLY:HA2	1:A:834:THR:HG22	1.88	0.54
1:A:512:ALA:HB1	1:A:877:GLN:HG3	1.89	0.54
1:A:402:PRO:O	1:A:406:LYS:HG3	2.06	0.54
1:A:805:ILE:HG22	1:A:831:VAL:HB	1.88	0.54
3:Y:5:A:O2'	3:Y:6:U:H5'	2.08	0.54
1:A:767:THR:HG21	2:X:2:C:OP1	2.07	0.54
3:Y:8:C:H2'	3:Y:9:G:H8	1.73	0.54
3:Y:10:C:O2'	3:Y:11:A:H5'	2.07	0.53
1:A:962:CYS:N	1:A:966:GLN:O	2.42	0.53
1:A:754:LEU:N	1:A:787:ILE:O	2.42	0.53
1:A:852:LYS:O	1:A:856:LYS:HG2	2.09	0.53
1:A:791:VAL:HG22	3:Y:7:G:H5"	1.90	0.53
1:A:472:LEU:HA	1:A:475:GLN:HB3	1.91	0.53
1:A:897:ASN:HD22	1:A:1007:PRO:HG2	1.74	0.52
1:A:366:LYS:HE2	3:Y:8:C:H4'	1.91	0.52
1:A:590:ALA:O	1:A:594:GLY:N	2.42	0.52
3:Y:9:G:H2'	3:Y:10:C:H6	1.75	0.52
1:A:777:LYS:HB2	1:A:783:ILE:HD12	1.92	0.52
1:A:551:PHE:CE2	1:A:620:MET:HB2	2.45	0.52
1:A:387:ARG:NH1	1:A:407:SER:OG	2.43	0.51
1:A:723:ILE:HD12	1:A:787:ILE:HG12	1.92	0.51
1:A:962:CYS:HB3	1:A:966:GLN:N	2.25	0.51
2:X:12:G:H2'	2:X:13:A:C8	2.46	0.51
1:A:309:LEU:HD11	1:A:342:TYR:CZ	2.46	0.51
2:X:8:G:O2'	2:X:9:C:H5'	2.10	0.51
3:Y:7:G:O2'	3:Y:8:C:H5'	2.10	0.51
1:A:381:TYR:C	1:A:382:LEU:HD12	2.32	0.50
1:A:392:SER:HB2	1:A:395:THR:HG23	1.94	0.50
2:X:12:G:H2'	2:X:13:A:H8	1.77	0.50
2:X:6:G:O2'	2:X:7:C:H5'	2.11	0.49
1:A:747:VAL:CG1	1:A:749:VAL:HG23	2.41	0.49
1:A:731:THR:HG22	1:A:787:ILE:HG22	1.93	0.49
2:X:2:C:H2'	2:X:3:C:C6	2.46	0.49
3:Y:13:G:O2'	3:Y:14:G:H5'	2.13	0.48
1:A:337:THR:O	1:A:341:VAL:HG23	2.13	0.48
1:A:728:ARG:O	1:A:731:THR:OG1	2.18	0.48
1:A:931:MET:HE1	1:A:1013:LEU:HD11	1.95	0.48
1:A:806:ARG:HB2	1:A:832:LEU:CD1	2.43	0.48
2:X:5:U:O2'	2:X:6:G:H5'	2.13	0.48

Continued on next page...

CONFIDENTIAL VALIDATION REPORT

Continued from previous page...

Atom-1	Atom-2	Interatomic distance (Å)	Clash overlap (Å)
1:A:792:ALA:O	1:A:796:LEU:N	2.47	0.47
1:A:578:HIS:CD2	2:X:15:G:H4'	2.49	0.47
1:A:619:ARG:CG	1:A:621:ILE:HG22	2.45	0.47
2:X:2:C:O2'	2:X:3:C:H5'	2.14	0.47
1:A:899:ASN:HB2	1:A:1008:ILE:CG2	2.44	0.47
1:A:720:ARG:NH2	1:A:779:ARG:O	2.47	0.47
1:A:907:CYS:SG	1:A:908:LYS:N	2.88	0.47
1:A:681:PHE:HA	1:A:684:LYS:HB2	1.97	0.46
1:A:637:LYS:O	1:A:641:VAL:HG23	2.15	0.46
3:Y:3:U:H2'	3:Y:4:C:H6	1.75	0.46
1:A:789:THR:OG1	1:A:791:VAL:HG23	2.16	0.46
1:A:915:CYS:SG	1:A:930:ASN:ND2	2.84	0.46
3:Y:14:G:H2'	3:Y:15:A:C8	2.51	0.46
1:A:452:GLU:O	2:X:9:C:H4'	2.17	0.45
1:A:641:VAL:HG21	1:A:965:GLY:HA2	1.98	0.45
1:A:873:ILE:O	1:A:877:GLN:HG3	2.17	0.45
1:A:425:LEU:HD22	1:A:433:VAL:O	2.16	0.44
1:A:340:ALA:O	1:A:344:THR:HG23	2.17	0.44
1:A:820:ARG:NH1	1:A:851:GLU:OE1	2.43	0.44
1:A:778:PHE:CG	1:A:786:LEU:HD11	2.53	0.44
1:A:309:LEU:HB3	1:A:314:MET:HE2	1.99	0.44
1:A:309:LEU:HD11	1:A:342:TYR:CE1	2.52	0.44
1:A:750:LYS:O	1:A:784:ASN:HB2	2.17	0.44
1:A:973:VAL:HG22	1:A:978:ASP:OD1	2.18	0.44
1:A:808:GLY:N	1:A:833:VAL:O	2.48	0.43
1:A:929:VAL:HG21	1:A:979:LEU:HD22	1.99	0.43
3:Y:14:G:H2'	3:Y:15:A:H8	1.83	0.43
1:A:471:ASP:O	1:A:475:GLN:CB	2.65	0.43
1:A:549:ASN:O	1:A:552:LYS:HB3	2.17	0.43
2:X:9:C:O2'	2:X:10:A:H5'	2.18	0.43
1:A:723:ILE:HG13	1:A:785:LEU:HD11	2.01	0.43
1:A:504:GLU:O	1:A:508:LEU:HD23	2.19	0.43
2:X:6:G:H2'	2:X:7:C:C6	2.54	0.43
1:A:870:ALA:O	1:A:874:LEU:HG	2.18	0.43
2:X:12:G:O2'	2:X:13:A:H5'	2.19	0.43
2:X:13:A:O2'	2:X:14:C:H5'	2.19	0.43
1:A:542:ILE:HD11	1:A:832:LEU:HD23	2.00	0.43
1:A:929:VAL:HG21	1:A:979:LEU:HB3	2.01	0.43
1:A:769:THR:OG1	2:X:1:U:OP1	2.32	0.43
1:A:583:ALA:O	1:A:602:ALA:HB1	2.19	0.42
1:A:344:THR:HG1	1:A:345:LYS:H	1.67	0.42

Continued on next page...

CONFIDENTIAL VALIDATION REPORT

Continued from previous page...

Atom-1	Atom-2	Interatomic distance (Å)	Clash overlap (Å)
1:A:741:ASN:HD22	1:A:742:ALA:N	2.17	0.42
1:A:472:LEU:HD22	1:A:479:ALA:N	2.35	0.41
2:X:11:U:O2'	2:X:12:G:H5'	2.20	0.41
1:A:642:LEU:HD23	1:A:642:LEU:O	2.20	0.41
2:X:6:G:H2'	2:X:7:C:H6	1.85	0.41
1:A:804:VAL:O	1:A:804:VAL:HG13	2.20	0.41
1:A:709:LEU:O	1:A:713:THR:HG23	2.20	0.41
1:A:884:LYS:O	1:A:888:VAL:HG23	2.21	0.41
1:A:791:VAL:HG23	3:Y:7:G:H5"	2.02	0.41
1:A:767:THR:HG21	2:X:2:C:P	2.60	0.41
1:A:349:ASP:O	1:A:353:GLN:HG2	2.20	0.41
1:A:722:ILE:HD13	1:A:801:CYS:SG	2.61	0.40
1:A:348:LEU:HD23	1:A:386:TYR:CD1	2.57	0.40
1:A:750:LYS:H	1:A:784:ASN:ND2	2.13	0.40

There are no symmetry-related clashes.

5.3 Torsion angles (i)

5.3.1 Protein backbone (i)

In the following table, the Percentiles column shows the percent Ramachandran outliers of the chain as a percentile score with respect to all PDB entries followed by that with respect to all EM entries.

The Analysed column shows the number of residues for which the backbone conformation was analysed, and the total number of residues.

Mol	Chain	Analysed	Favoured	Allowed	Outliers	Percentiles	
1	A	640/1007 (64%)	579 (90%)	61 (10%)	0	100	100

There are no Ramachandran outliers to report.

5.3.2 Protein sidechains (i)

In the following table, the Percentiles column shows the percent sidechain outliers of the chain as a percentile score with respect to all PDB entries followed by that with respect to all EM entries.

The Analysed column shows the number of residues for which the sidechain conformation was analysed, and the total number of residues.

CONFIDENTIAL VALIDATION REPORT

Mol	Chain	Analysed	Rotameric	Outliers	Percentiles	
1	A	569/900 (63%)	564 (99%)	5 (1%)	81	91

All (5) residues with a non-rotameric sidechain are listed below:

Mol	Chain	Res	Type
1	A	451	LYS
1	A	534	LYS
1	A	606	ARG
1	A	741	ASN
1	A	990	ASN

Some sidechains can be flipped to improve hydrogen bonding and reduce clashes. All (8) such sidechains are listed below:

Mol	Chain	Res	Type
1	A	563	GLN
1	A	678	ASN
1	A	683	ASN
1	A	706	ASN
1	A	741	ASN
1	A	784	ASN
1	A	897	ASN
1	A	990	ASN

5.3.3 RNA ⓘ

Mol	Chain	Analysed	Backbone Outliers	Pucker Outliers
2	X	14/15 (93%)	1 (7%)	0
3	Y	14/15 (93%)	0	0
All	All	28/30 (93%)	1 (3%)	0

All (1) RNA backbone outliers are listed below:

Mol	Chain	Res	Type
2	X	15	G

There are no RNA pucker outliers to report.

5.4 Non-standard residues in protein, DNA, RNA chains ⓘ

There are no non-standard protein/DNA/RNA residues in this entry.

CONFIDENTIAL VALIDATION REPORT

5.5 Carbohydrates (i)

There are no carbohydrates in this entry.

5.6 Ligand geometry (i)

Of 1 ligands modelled in this entry, 1 is monoatomic - leaving 0 for Mogul analysis.

There are no bond length outliers.

There are no bond angle outliers.

There are no chirality outliers.

There are no torsion outliers.

There are no ring outliers.

No monomer is involved in short contacts.

5.7 Other polymers (i)

There are no such residues in this entry.

5.8 Polymer linkage issues (i)

There are no chain breaks in this entry.

CONFIDENTIAL VALIDATION REPORT

Click here to access/download;PDB Validation Report;D_1200011125_val-report-full_P1_6H66.pdf

Full wwPDB/EMDataBank EM Map/Model Validation Report (i)

Jul 27, 2018 – 07:51 AM BST

PDB ID	:	6H66
EMDB ID:	:	4338
Title	:	CryoEM structure of the MDA5-dsRNA filament with 93 degree twist and without nucleotide
Deposited on	:	2018-07-26
Resolution	:	4.16 Å(reported)
Based on PDB ID	:	6G1X

This is a Full wwPDB/EMDataBank EM Map/Model Validation Report.

This report is produced by the wwPDB biocuration pipeline after annotation of the structure.

We welcome your comments at *validation@mail.wwpdb.org*
A user guide is available at
https://www.wwpdb.org/validation/2017/EMValidationReportHelp
with specific help available everywhere you see the (i) symbol.

MolProbity	:	4.02b-467
Percentile statistics	:	20171227.v01 (using entries in the PDB archive December 27th 2017)
Ideal geometry (proteins)	:	Engh & Huber (2001)
Ideal geometry (DNA, RNA)	:	Parkinson et. al. (1996)
Validation Pipeline (wwPDB-VP)	:	rb-20031172

CONFIDENTIAL VALIDATION REPORT

1 Overall quality at a glance (i)

The following experimental techniques were used to determine the structure:
ELECTRON MICROSCOPY

The reported resolution of this entry is 4.16 Å.

Percentile scores (ranging between 0-100) for global validation metrics of the entry are shown in the following graphic. The table shows the number of entries on which the scores are based.

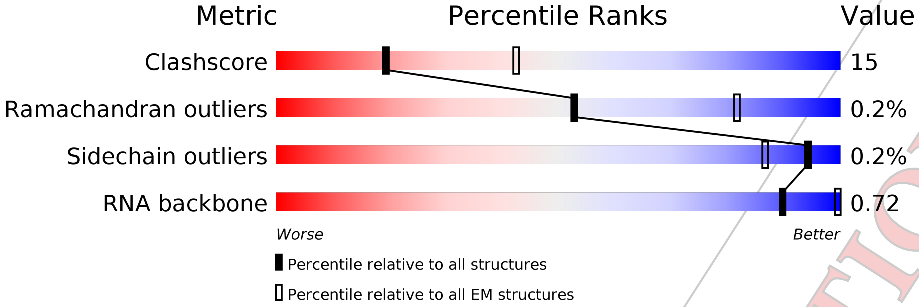

Metric	Whole archive (#Entries)	EM structures (#Entries)
Clashscore	136327	1886
Ramachandran outliers	132723	1663
Sidechain outliers	132532	1531
RNA backbone	3747	458

The table below summarises the geometric issues observed across the polymeric chains. The red, orange, yellow and green segments on the bar indicate the fraction of residues that contain outliers for >=3, 2, 1 and 0 types of geometric quality criteria. A grey segment represents the fraction of residues that are not modelled. The numeric value for each fraction is indicated below the corresponding segment, with a dot representing fractions <=5%

Mol	Chain	Length	Quality of chain
1	A	1007	48% 18% 34%
2	X	15	93% 7%
3	Y	15	7% 93%

CONFIDENTIAL VALIDATION REPORT

2 Entry composition (i)

There are 4 unique types of molecules in this entry. The entry contains 10017 atoms, of which 4120 are hydrogens and 0 are deuteriums.

In the tables below, the AltConf column contains the number of residues with at least one atom in alternate conformation and the Trace column contains the number of residues modelled with at most 2 atoms.

- Molecule 1 is a protein called Interferon-induced helicase C domain-containing protein 1.

Mol	Chain	Residues	Atoms						AltConf	Trace
			Total	C	H	N	O	S		
1	A	667	9377	3339	4120	920	965	33	0	0

There are 18 discrepancies between the modelled and reference sequences:

Chain	Residue	Modelled	Actual	Comment	Reference
A	?	-	ASP	deletion	UNP Q8R5F7
A	?	-	LYS	deletion	UNP Q8R5F7
A	?	-	SER	deletion	UNP Q8R5F7
A	?	-	ASP	deletion	UNP Q8R5F7
A	?	-	ASP	deletion	UNP Q8R5F7
A	?	-	GLU	deletion	UNP Q8R5F7
A	?	-	ALA	deletion	UNP Q8R5F7
A	?	-	SER	deletion	UNP Q8R5F7
A	?	-	SER	deletion	UNP Q8R5F7
A	?	-	CYS	deletion	UNP Q8R5F7
A	?	-	ASN	deletion	UNP Q8R5F7
A	?	-	ASP	deletion	UNP Q8R5F7
A	?	-	GLN	deletion	UNP Q8R5F7
A	?	-	LEU	deletion	UNP Q8R5F7
A	?	-	LYS	deletion	UNP Q8R5F7
A	?	-	GLY	deletion	UNP Q8R5F7
A	?	-	ASP	deletion	UNP Q8R5F7
A	?	-	VAL	deletion	UNP Q8R5F7

- Molecule 2 is a RNA chain called RNA (5'-R(P*UP*CP*CP*AP*UP*GP*CP*GP*CP*AP *UP*GP*AP*CP*G)-3').

Mol	Chain	Residues	Atoms					AltConf	Trace
			Total	C	N	O	P		
2	X	15	318	142	56	105	15	0	0

- Molecule 3 is a RNA chain called RNA (5'-R(P*CP*GP*UP*CP*AP*UP*GP*CP*GP*CP

CONFIDENTIAL VALIDATION REPORT

*AP*UP*GP*GP*A)-3').

Mol	Chain	Residues	Atoms					AltConf	Trace
			Total	C	N	O	P		
3	Y	15	321	143	58	105	15	0	0

- Molecule 4 is ZINC ION (three-letter code: ZN) (formula: Zn).

Mol	Chain	Residues	Atoms		AltConf
			Total	Zn	
4	A	1	1	1	0

CONFIDENTIAL VALIDATION REPORT

3 Residue-property plots (i)

These plots are drawn for all protein, RNA and DNA chains in the entry. The first graphic for a chain summarises the proportions of the various outlier classes displayed in the second graphic. The second graphic shows the sequence view annotated by issues in geometry. Residues are color-coded according to the number of geometric quality criteria for which they contain at least one outlier: green = 0, yellow = 1, orange = 2 and red = 3 or more. Stretches of 2 or more consecutive residues without any outlier are shown as a green connector. Residues present in the sample, but not in the model, are shown in grey.

• Molecule 1: Interferon-induced helicase C domain-containing protein 1

Chain A:

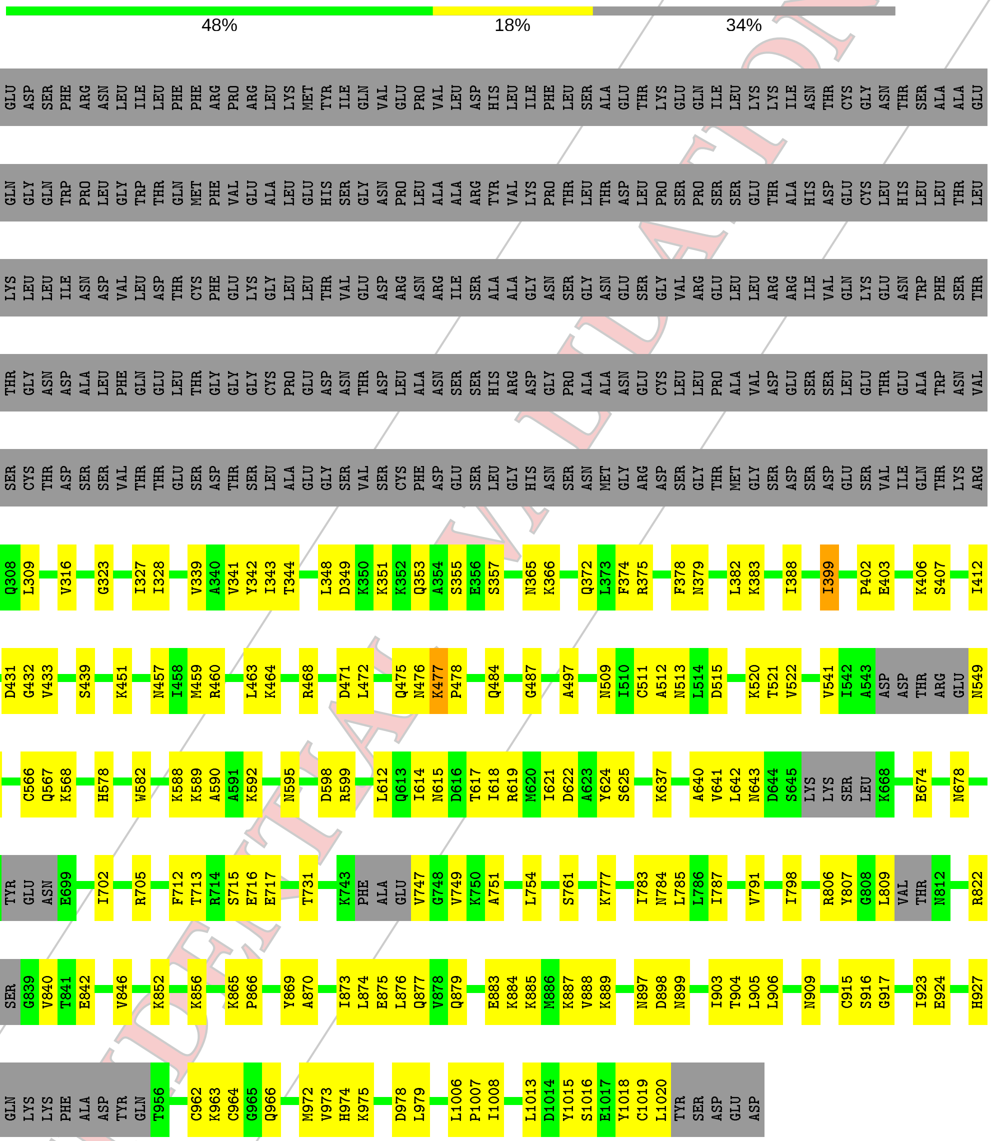

• Molecule 2: RNA (5'-R(P*UP*CP*CP*AP*UP*GP*CP*GP*CP*AP*UP*GP*AP*CP*G)-3')

Chain X:

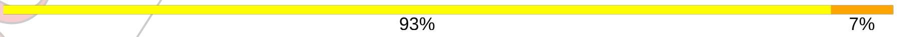

CONFIDENTIAL VALIDATION REPORT

U1 C2 C3 A4 U5 G6 C7 G8 C9 A10 U11 G12 A13 C14 G15

• Molecule 3: RNA (5'-R(P*CP*GP*UP*CP*AP*UP*GP*CP*GP*CP*AP*UP*GP*GP*A)-3')

Chain Y: 7% 93%

C1 G2 U3 C4 A5 U6 G7 C8 G9 C10 A11 U12 G13 G14 A15

CONFIDENTIAL VALIDATION REPORT

4 Experimental information (i)

Property	Value	Source
Reconstruction method	HELICAL	Depositor
Imposed symmetry	HELICAL, twist=93.0596°, rise=44.366 Å, axial sym=C1	Depositor
Number of segments used	19111	Depositor
Resolution determination method	FSC 0.143 CUT-OFF	Depositor
CTF correction method	PHASE FLIPPING AND AMPLITUDE CORRECTION	Depositor
Microscope	FEI TITAN KRIOS	Depositor
Voltage (kV)	300	Depositor
Electron dose $(e^-/\text{Å}^2)$	27.0	Depositor
Minimum defocus (nm)	-1800	Depositor
Maximum defocus (nm)	-2700	Depositor
Magnification	75000	Depositor
Image detector	FEI FALCON III (4k x 4k)	Depositor

CONFIDENTIAL VALIDATION REPORT

5 Model quality (i)

5.1 Standard geometry (i)

Bond lengths and bond angles in the following residue types are not validated in this section: ZN

The Z score for a bond length (or angle) is the number of standard deviations the observed value is removed from the expected value. A bond length (or angle) with $|Z| > 5$ is considered an outlier worth inspection. RMSZ is the root-mean-square of all Z scores of the bond lengths (or angles).

Mol	Chain	Bond lengths		Bond angles	
		RMSZ	#|Z| >2	RMSZ	#|Z| >2
1	A	0.42	0/5338	0.56	2/7184 (0.0%)
2	X	0.66	0/354	0.93	0/549
3	Y	0.65	0/358	0.90	0/556
All	All	0.45	0/6050	0.62	2/8289 (0.0%)

Chiral center outliers are detected by calculating the chiral volume of a chiral center and verifying if the center is modelled as a planar moiety or with the opposite hand.A planarity outlier is detected by checking planarity of atoms in a peptide group, atoms in a mainchain group or atoms of a sidechain that are expected to be planar.

Mol	Chain	#Chirality outliers	#Planarity outliers
1	A	0	1

There are no bond length outliers.

All (2) bond angle outliers are listed below:

Mol	Chain	Res	Type	Atoms	Z	Observed(^{o})	Ideal(^{o})
1	A	497	ALA	C-N-CA	6.51	137.97	121.70
1	A	399	ILE	C-N-CA	6.05	136.83	121.70

There are no chirality outliers.

All (1) planarity outliers are listed below:

Mol	Chain	Res	Type	Group
1	A	477	LYS	Peptide

CONFIDENTIAL VALIDATION REPORT

5.2 Too-close contacts (i)

In the following table, the Non-H and H(model) columns list the number of non-hydrogen atoms and hydrogen atoms in the chain respectively. The H(added) column lists the number of hydrogen atoms added and optimized by MolProbity. The Clashes column lists the number of clashes within the asymmetric unit, whereas Symm-Clashes lists symmetry related clashes.

Mol	Chain	Non-H	H(model)	H(added)	Clashes	Symm-Clashes
1	A	5257	4120	5329	130	0
2	X	318	0	163	25	0
3	Y	321	0	163	26	0
4	A	1	0	0	0	0
All	All	5897	4120	5655	175	0

The all-atom clashscore is defined as the number of clashes found per 1000 atoms (including hydrogen atoms). The all-atom clashscore for this structure is 15.

All (175) close contacts within the same asymmetric unit are listed below, sorted by their clash magnitude.

Atom-1	Atom-2	Interatomic distance (Å)	Clash overlap (Å)
1:A:749:VAL:HA	1:A:784:ASN:HD22	1.40	0.85
3:Y:1:C:H2'	3:Y:2:G:H8	1.49	0.77
1:A:599:ARG:NH1	1:A:909:ASN:OD1	2.18	0.77
1:A:374:PHE:O	1:A:379:ASN:ND2	2.18	0.77
1:A:751:ALA:HB2	1:A:785:LEU:HB3	1.69	0.74
1:A:457:ASN:OD1	1:A:460:ARG:NH2	2.22	0.73
1:A:715:SER:OG	1:A:717:GLU:O	2.07	0.72
2:X:3:C:H2'	2:X:4:A:H8	1.55	0.71
2:X:5:U:H2'	2:X:6:G:H8	1.56	0.70
1:A:372:GLN:OE1	1:A:375:ARG:NH2	2.25	0.70
1:A:924:GLU:OE2	1:A:974:HIS:NE2	2.25	0.70
1:A:379:ASN:O	1:A:383:LYS:N	2.26	0.68
2:X:5:U:H2'	2:X:6:G:C8	2.28	0.68
1:A:339:VAL:O	1:A:343:ILE:HD12	1.94	0.68
1:A:927:HIS:NE2	2:X:5:U:O2'	2.26	0.68
3:Y:4:C:H2'	3:Y:5:A:H8	1.60	0.67
3:Y:4:C:H2'	3:Y:5:A:C8	2.30	0.67
2:X:9:C:H2'	2:X:10:A:H8	1.61	0.66
2:X:9:C:H2'	2:X:10:A:C8	2.31	0.65
3:Y:1:C:H2'	3:Y:2:G:C8	2.31	0.65
1:A:791:VAL:HG23	3:Y:7:G:H5"	1.79	0.65
2:X:3:C:H2'	2:X:4:A:C8	2.31	0.65
1:A:832:LEU:HD21	1:A:840:VAL:HG12	1.80	0.64

Continued on next page...

CONFIDENTIAL VALIDATION REPORT

Continued from previous page...

Atom-1	Atom-2	Interatomic distance (Å)	Clash overlap (Å)
1:A:341:VAL:O	1:A:344:THR:OG1	2.11	0.64
1:A:425:LEU:HB2	1:A:433:VAL:H	1.63	0.63
1:A:349:ASP:O	1:A:353:GLN:HG2	1.97	0.63
1:A:915:CYS:SG	1:A:930:ASN:ND2	2.71	0.63
1:A:351:LYS:O	1:A:355:SER:N	2.32	0.62
1:A:617:THR:O	1:A:807:TYR:OH	2.12	0.62
1:A:962:CYS:HB3	1:A:966:GLN:H	1.62	0.62
1:A:471:ASP:O	1:A:475:GLN:N	2.32	0.62
1:A:614:ILE:O	1:A:618:ILE:N	2.33	0.61
1:A:1018:TYR:O	1:A:1020:LEU:HD12	2.01	0.60
1:A:798:ILE:HB	1:A:822:ARG:HD2	1.82	0.60
3:Y:8:C:H2'	3:Y:9:G:C8	2.37	0.60
1:A:962:CYS:SG	1:A:963:LYS:N	2.75	0.60
3:Y:14:G:H2'	3:Y:15:A:H8	1.66	0.60
1:A:761:SER:OG	3:Y:6:U:OP2	2.19	0.60
1:A:323:GLY:O	1:A:484:GLN:NE2	2.35	0.60
1:A:974:HIS:HB3	1:A:979:LEU:HD12	1.84	0.59
3:Y:14:G:H2'	3:Y:15:A:C8	2.37	0.59
1:A:622:ASP:O	1:A:625:SER:OG	2.19	0.59
3:Y:3:U:H2'	3:Y:4:C:C6	2.38	0.59
2:X:11:U:H2'	2:X:12:G:H8	1.67	0.58
1:A:357:SER:O	1:A:439:SER:OG	2.19	0.58
3:Y:10:C:O2'	3:Y:11:A:H5'	2.03	0.57
3:Y:8:C:H2'	3:Y:9:G:H8	1.69	0.57
2:X:11:U:H2'	2:X:12:G:C8	2.38	0.57
1:A:595:ASN:ND2	1:A:598:ASP:OD2	2.38	0.57
1:A:852:LYS:O	1:A:856:LYS:HG2	2.06	0.56
1:A:509:ASN:O	1:A:513:ASN:ND2	2.38	0.56
1:A:923:ILE:HD12	1:A:924:GLU:HG2	1.87	0.56
1:A:870:ALA:HA	1:A:873:ILE:HG12	1.87	0.56
3:Y:7:G:O2'	3:Y:8:C:H5'	2.06	0.56
1:A:865:LYS:HD2	1:A:866:PRO:HD2	1.87	0.55
3:Y:3:U:H2'	3:Y:4:C:H6	1.71	0.55
3:Y:5:A:O2'	3:Y:6:U:H5'	2.05	0.55
1:A:883:GLU:OE2	1:A:887:LYS:HE2	2.06	0.55
1:A:842:GLU:O	1:A:846:VAL:HG23	2.08	0.54
2:X:10:A:O2'	2:X:11:U:H5'	2.07	0.54
1:A:588:LYS:O	1:A:592:LYS:N	2.40	0.53
1:A:512:ALA:HB1	1:A:877:GLN:HG3	1.88	0.53
1:A:399:ILE:HD11	1:A:403:GLU:CB	2.39	0.53
1:A:566:CYS:HB2	1:A:568:LYS:HG2	1.91	0.53

Continued on next page...

CONFIDENTIAL VALIDATION REPORT

Continued from previous page...

Atom-1	Atom-2	Interatomic distance (Å)	Clash overlap (Å)
2:X:4:A:O2'	2:X:5:U:H5'	2.09	0.53
1:A:806:ARG:HD2	1:A:809:LEU:HD23	1.92	0.52
1:A:962:CYS:SG	1:A:964:CYS:N	2.77	0.52
2:X:7:C:H2'	2:X:8:G:C8	2.45	0.52
1:A:751:ALA:CB	1:A:785:LEU:HB3	2.37	0.52
1:A:962:CYS:HB3	1:A:966:GLN:N	2.24	0.52
2:X:8:G:O2'	2:X:9:C:H5'	2.10	0.52
1:A:464:LYS:HE2	1:A:468:ARG:HH21	1.75	0.52
1:A:749:VAL:HA	1:A:784:ASN:ND2	2.17	0.51
1:A:712:PHE:HE2	1:A:747:VAL:HG22	1.74	0.51
1:A:806:ARG:HG3	1:A:830:TYR:OH	2.10	0.51
1:A:558:ILE:O	1:A:561:SER:OG	2.23	0.51
1:A:590:ALA:HB3	1:A:599:ARG:HB3	1.92	0.51
1:A:897:ASN:HB2	1:A:1007:PRO:HG2	1.92	0.51
1:A:962:CYS:N	1:A:966:GLN:O	2.41	0.51
2:X:12:G:H2'	2:X:13:A:H8	1.76	0.51
2:X:6:G:O2'	2:X:7:C:H5'	2.11	0.50
1:A:511:CYS:O	1:A:515:ASP:N	2.44	0.50
2:X:7:C:H2'	2:X:8:G:H8	1.76	0.50
1:A:566:CYS:CB	1:A:568:LYS:HG2	2.42	0.50
1:A:640:ALA:HA	1:A:643:ASN:OD1	2.11	0.50
1:A:624:TYR:CE2	1:A:688:LYS:HD2	2.47	0.49
1:A:791:VAL:CG2	3:Y:7:G:H5"	2.41	0.49
1:A:731:THR:HG22	1:A:787:ILE:HG22	1.94	0.49
1:A:589:LYS:HD3	1:A:592:LYS:HD2	1.94	0.49
3:Y:3:U:O2'	3:Y:4:C:H5'	2.13	0.49
2:X:13:A:O2'	2:X:14:C:H5'	2.13	0.48
1:A:974:HIS:CE1	1:A:975:LYS:HG2	2.48	0.48
1:A:402:PRO:O	1:A:406:LYS:HG3	2.14	0.48
1:A:422:LEU:O	1:A:425:LEU:HB3	2.14	0.48
3:Y:6:U:H2'	3:Y:7:G:H8	1.78	0.48
1:A:869:TYR:O	1:A:873:ILE:HG23	2.14	0.48
1:A:566:CYS:HB3	1:A:568:LYS:HE2	1.96	0.47
2:X:1:U:H2'	2:X:2:C:C6	2.49	0.47
1:A:316:VAL:HG23	1:A:520:LYS:O	2.14	0.47
1:A:425:LEU:O	1:A:428:GLY:N	2.47	0.47
1:A:425:LEU:HD22	1:A:433:VAL:O	2.15	0.47
1:A:328:ILE:N	1:A:487:GLY:O	2.46	0.47
2:X:2:C:H2'	2:X:3:C:H6	1.79	0.47
1:A:905:LEU:C	1:A:906:LEU:HD12	2.35	0.47
1:A:1006:LEU:HD13	1:A:1008:ILE:HG13	1.96	0.47

Continued on next page...

CONFIDENTIAL VALIDATION REPORT

Continued from previous page...

Atom-1	Atom-2	Interatomic distance (Å)	Clash overlap (Å)
1:A:712:PHE:CE2	1:A:747:VAL:HG22	2.50	0.47
1:A:365:ASN:OD1	1:A:366:LYS:N	2.48	0.47
1:A:1015:TYR:O	1:A:1018:TYR:N	2.48	0.46
1:A:382:LEU:HB2	1:A:388:ILE:HD11	1.97	0.46
2:X:12:G:H2'	2:X:13:A:C8	2.50	0.46
3:Y:9:G:H2'	3:Y:10:C:H6	1.80	0.46
1:A:309:LEU:HD11	1:A:342:TYR:CD1	2.50	0.46
1:A:472:LEU:HA	1:A:475:GLN:HB3	1.97	0.46
1:A:549:ASN:HB2	1:A:550:PRO:HD3	1.98	0.46
1:A:885:LYS:O	1:A:889:LYS:HG2	2.16	0.46
1:A:460:ARG:HE	1:A:884:LYS:HD2	1.81	0.46
1:A:1006:LEU:HD13	1:A:1008:ILE:CG1	2.46	0.46
1:A:637:LYS:O	1:A:641:VAL:HG23	2.15	0.46
1:A:459:MET:O	1:A:463:LEU:HG	2.16	0.46
1:A:476:ASN:HB2	1:A:478:PRO:HD2	1.97	0.46
1:A:716:GLU:O	1:A:717:GLU:HB2	2.15	0.46
1:A:916:SER:OG	1:A:917:GLY:N	2.48	0.45
2:X:12:G:O2'	2:X:13:A:H5'	2.16	0.45
3:Y:2:G:O2'	3:Y:3:U:H5'	2.16	0.45
3:Y:6:U:H2'	3:Y:7:G:C8	2.52	0.45
3:Y:9:G:O2'	3:Y:10:C:H5'	2.17	0.45
1:A:674:GLU:O	1:A:678:ASN:ND2	2.50	0.45
1:A:563:GLN:O	1:A:567:GLN:N	2.50	0.45
1:A:923:ILE:CD1	1:A:924:GLU:HG2	2.47	0.45
1:A:619:ARG:HG3	1:A:621:ILE:HG22	1.99	0.45
2:X:2:C:O2'	2:X:3:C:H5'	2.17	0.44
1:A:974:HIS:ND1	1:A:975:LYS:HG2	2.32	0.44
1:A:378:PHE:CD2	1:A:412:ILE:HD11	2.53	0.44
1:A:589:LYS:HA	1:A:592:LYS:HB2	1.98	0.44
2:X:1:U:H2'	2:X:2:C:H6	1.83	0.44
1:A:702:ILE:HG12	1:A:705:ARG:HH21	1.83	0.43
3:Y:13:G:O2'	3:Y:14:G:H5'	2.17	0.43
3:Y:4:C:O2'	3:Y:5:A:H5'	2.17	0.43
1:A:973:VAL:HG22	1:A:978:ASP:OD1	2.17	0.43
1:A:972:MET:O	1:A:979:LEU:N	2.51	0.43
1:A:568:LYS:HB3	1:A:582:TRP:HH2	1.84	0.43
1:A:903:ILE:HD12	1:A:904:THR:CA	2.48	0.43
1:A:870:ALA:O	1:A:874:LEU:HG	2.19	0.43
1:A:588:LYS:O	1:A:592:LYS:HG3	2.19	0.43
1:A:1018:TYR:CE2	1:A:1020:LEU:HD11	2.54	0.43
1:A:472:LEU:O	1:A:477:LYS:N	2.35	0.43

Continued on next page...

CONFIDENTIAL VALIDATION REPORT

Continued from previous page...

Atom-1	Atom-2	Interatomic distance (Å)	Clash overlap (Å)
2:X:3:C:O2'	2:X:4:A:H5'	2.19	0.43
1:A:898:ASP:HA	1:A:1008:ILE:HA	2.02	0.42
1:A:612:LEU:O	1:A:615:ASN:N	2.52	0.42
3:Y:9:G:H2'	3:Y:10:C:C6	2.54	0.42
1:A:541:VAL:HB	1:A:831:VAL:HG13	2.01	0.42
1:A:578:HIS:CD2	2:X:15:G:H4'	2.55	0.42
1:A:642:LEU:HD23	1:A:642:LEU:O	2.20	0.42
1:A:777:LYS:HB2	1:A:783:ILE:HD12	2.00	0.42
1:A:754:LEU:N	1:A:787:ILE:O	2.53	0.42
1:A:884:LYS:O	1:A:888:VAL:HG23	2.19	0.42
1:A:619:ARG:CG	1:A:621:ILE:HG22	2.49	0.42
1:A:931:MET:CE	1:A:1013:LEU:HD11	2.49	0.42
1:A:683:ASN:O	1:A:686:MET:HG2	2.19	0.41
1:A:590:ALA:HB1	1:A:595:ASN:OD1	2.20	0.41
1:A:899:ASN:H	1:A:1008:ILE:HG22	1.85	0.41
1:A:875:GLU:O	1:A:879:GLN:HG3	2.20	0.41
1:A:521:THR:HG22	1:A:522:VAL:N	2.35	0.41
1:A:713:THR:O	1:A:716:GLU:HG2	2.20	0.41
1:A:975:LYS:HE2	1:A:975:LYS:HA	2.02	0.41
1:A:1016:SER:HA	1:A:1019:CYS:SG	2.61	0.41
1:A:327:ILE:HG22	1:A:328:ILE:N	2.36	0.40
1:A:344:THR:O	1:A:348:LEU:HD13	2.21	0.40
1:A:974:HIS:HB3	1:A:979:LEU:CD1	2.50	0.40
1:A:791:VAL:HG22	3:Y:7:G:O3'	2.21	0.40
1:A:459:MET:O	1:A:463:LEU:N	2.48	0.40
1:A:460:ARG:HA	1:A:463:LEU:HD12	2.03	0.40
1:A:402:PRO:HG2	1:A:431:ASP:HB3	2.03	0.40
1:A:403:GLU:O	1:A:407:SER:N	2.54	0.40
1:A:876:LEU:HD23	1:A:876:LEU:HA	1.86	0.40

There are no symmetry-related clashes.

5.3 Torsion angles (i)

5.3.1 Protein backbone (i)

In the following table, the Percentiles column shows the percent Ramachandran outliers of the chain as a percentile score with respect to all PDB entries followed by that with respect to all EM entries.

The Analysed column shows the number of residues for which the backbone conformation was analysed, and the total number of residues.

CONFIDENTIAL VALIDATION REPORT

Mol	Chain	Analysed	Favoured	Allowed	Outliers	Percentiles	
1	A	651/1007 (65%)	582 (89%)	68 (10%)	1 (0%)	49	83

All (1) Ramachandran outliers are listed below:

Mol	Chain	Res	Type
1	A	432	GLY

5.3.2 Protein sidechains (i)

In the following table, the Percentiles column shows the percent sidechain outliers of the chain as a percentile score with respect to all PDB entries followed by that with respect to all EM entries.

The Analysed column shows the number of residues for which the sidechain conformation was analysed, and the total number of residues.

Mol	Chain	Analysed	Rotameric	Outliers	Percentiles	
1	A	573/900 (64%)	572 (100%)	1 (0%)	94	96

All (1) residues with a non-rotameric sidechain are listed below:

Mol	Chain	Res	Type
1	A	451	LYS

Some sidechains can be flipped to improve hydrogen bonding and reduce clashes. All (1) such sidechains are listed below:

Mol	Chain	Res	Type
1	A	784	ASN

5.3.3 RNA (i)

Mol	Chain	Analysed	Backbone Outliers	Pucker Outliers
2	X	14/15 (93%)	1 (7%)	0
3	Y	14/15 (93%)	0	0
All	All	28/30 (93%)	1 (3%)	0

All (1) RNA backbone outliers are listed below:

Mol	Chain	Res	Type
2	X	15	G

CONFIDENTIAL VALIDATION REPORT

There are no RNA pucker outliers to report.

5.4 Non-standard residues in protein, DNA, RNA chains (i)

There are no non-standard protein/DNA/RNA residues in this entry.

5.5 Carbohydrates (i)

There are no carbohydrates in this entry.

5.6 Ligand geometry (i)

Of 1 ligands modelled in this entry, 1 is monoatomic - leaving 0 for Mogul analysis.

There are no bond length outliers.

There are no bond angle outliers.

There are no chirality outliers.

There are no torsion outliers.

There are no ring outliers.

No monomer is involved in short contacts.

5.7 Other polymers (i)

There are no such residues in this entry.

5.8 Polymer linkage issues (i)

There are no chain breaks in this entry.

CONFIDENTIAL VALIDATION REPORT
